# Resting-state EEG alterations and cognitive impairment in atrial fibrillation: insights into neural biomarkers and functional connectivity

**DOI:** 10.3389/fneur.2025.1583715

**Published:** 2025-06-19

**Authors:** Min-qiang Bao, Li Cao, Yi-nong Chen, Guo-liang Gao, Zhi-yong Lu, Jie Wang, Shuang-shuang Chen, Xiao-ning Sheng, Yu Wang

**Affiliations:** ^1^Department of Neurology, The Affiliated Xuancheng Hospital of Wannan Medical College, Xuancheng, China; ^2^Department of Neurology, The First Affiliated Hospital of Anhui Medical University, Hefei, China; ^3^Department of Electrocardiogram, The First Affiliated Hospital of Anhui Medical University, Hefei, China; ^4^Department of Electrocardiogram, The Affiliated Xuancheng Hospital of Wannan Medical College, Xuancheng, China; ^5^Department of Cardiovascular Medicine, The Affiliated Xuancheng Hospital of Wannan Medical College, Xuancheng, China

**Keywords:** atrial fibrillation, cognitive dysfunction, resting-state EEG, power spectral density, functional connectivity, cross-frequency coupling, sample entropy

## Abstract

**Aims:**

Atrial fibrillation (AF) is associated with cognitive decline, but the role of electroencephalography (EEG) in assessing cognitive dysfunction in AF patients is underexplored.

**Objective:**

This study investigated the relationship between resting-state EEG patterns and cognitive impairment in AF patients.

**Methods:**

We recruited 120 participants from the Affiliated Xuancheng Hospital, China (January 2023 to January 2024), categorizing them into healthy controls and AF patients. Resting-state EEG metrics, including power spectral density (PSD), functional connectivity (FC), cross-frequency coupling (CFC), and sample entropy (EnSA), were analyzed alongside the Montreal Cognitive Assessment (MoCA) scores. Mediation analysis explored EEG's role in the AF-cognitive decline relationship.

**Results:**

AF patients had significantly lower MoCA scores. PSD analysis showed increased δ and θ and decreased α and β activity. FC was reduced in the α and β bands but increased in localized θ and γ bands. CFC analysis revealed elevated θ–β and θ–γ phase-amplitude coupling (PAC), reduced β–γ PAC, and lower EnSA. EEG metrics were significantly correlated with MoCA scores, with θ–β PAC mediating cognitive decline.

**Conclusion:**

AF patients exhibit distinctive EEG changes, with θ–β PAC mediating cognitive impairment, suggesting the potential of resting-state EEG for cognitive assessment in AF patients.

## Introduction

Atrial fibrillation (AF) is one of the most prevalent cardiac arrhythmias, significantly associated with increased risks of stroke, heart failure, and mortality (1, 2). It is also linked to a higher prevalence of chronic disease comorbidities (3). As the population ages, the incidence of cognitive dysfunctions, including mild cognitive impairment and dementia, continues to rise (4). Dementia is characterized by progressive cognitive decline and loss of daily living skills, yet its pathological mechanisms are still not fully understood. There is growing evidence suggesting a critical role of AF in the development of cognitive decline and dementia. For example, the Rotterdam study found that the risk of cognitive dysfunction in AF patients was twice as high as in individuals without AF (5). Although stroke is a well-known cause of cognitive impairment in AF (6), recent studies indicate that AF significantly elevates the risk of cognitive dysfunction. This risk often persists independent of a stroke history (7–11). For instance, a meta-analysis by Kalantarian et al. (12) demonstrated that AF is associated with an increased risk of cognitive dysfunction and dementia, irrespective of prior stroke history. These findings show the urgent need for more in-depth research into the mechanisms connecting AF and cognitive decline.

Electroencephalogram (EEG) is a non-invasive technique with high temporal resolution that has shown potential in the early detection of cognitive impairment. Patients with cognitive impairment often exhibit EEG patterns characterized by increased low-frequency power (δ, θ) and decreased high-frequency power (α, β) (13, 14), accompanied by reduced signal complexity and coherence (15–19). These EEG features are closely related to the transition of brain function from normal to pathological. Consequently, EEG has been widely used in the diagnosis of mild cognitive impairment, Alzheimer's disease, and other neurodegenerative diseases. Furthermore, the sensitivity of EEG to early neurological changes has led to its recommendation as a complementary tool to neuroimaging biomarkers in clinical trials for neurodegenerative diseases.

Although AF and cognitive dysfunction share multiple common risk factors, systematic studies that utilize electroencephalography (EEG) to assess cognitive decline in AF patients are still limited. Resting-state EEG reflects the functional state and network characteristics of the brain. To comprehensively capture the neurological changes associated with AF, we employed four complementary categories of EEG metrics. First, power spectral density (PSD) quantifies the intensity of neural oscillations in all frequency bands and represents linear oscillatory activity. It is the most commonly used spectral measure in studies of cognitive impairment (20). Second, functional connectivity (FC) metrics, such as coherence and the weighted phase lag index (wPLI), measure synchronization between brain regions and reveal network-level disruption (21). Third, cross-frequency coupling (CFC), especially phase-amplitude coupling (PAC), captures non-linear coupling mechanisms between different frequency bands and is related to cognitive processes such as attention and memory (22). Lastly, entropy-based complexity metrics, such as sample entropy (EnSA), quantify the reduction in signal complexity that typically occurs from the early stages of mild cognitive impairment (23). These spectral features, network connectivity patterns, PAC, and non-linear complexity (e.g., entropy) metrics constitute a multidimensional neurophysiological framework for understanding cognitive dysfunction in patients with AF, especially those without a history of stroke.

In this study, we analyzed the resting-state EEG characteristics of AF patients and examined their associations with cognitive function, aiming to provide a theoretical basis for understanding the mechanisms of AF-related cognitive impairment and developing early screening and intervention strategies.

## Methods

### Study population

**Figure 1:**
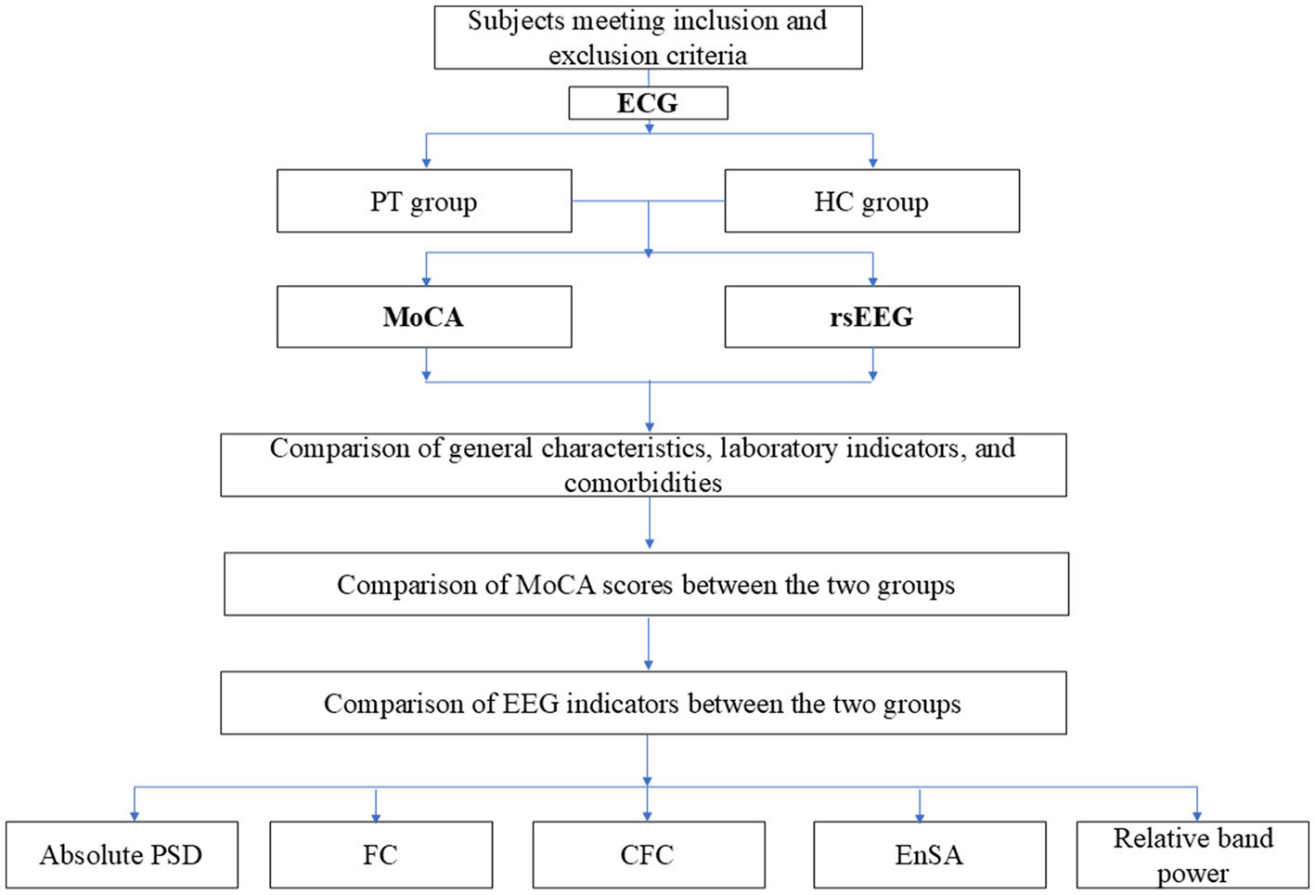
Study flowchart.

This cross-sectional study included 120 patients admitted to the Affiliated Xuancheng Hospital of Wannan Medical College, China, between January 2023 and January 2024. Participants were categorized into an AF patient (PT) group and a healthy control (HC) group based on electrocardiogram (ECG) diagnostic results or ambulatory ECG obtained at the time of admission. Ethical approval was obtained from the Xuancheng People's Hospital Medical Ethics Committee, and all patients provided written informed consent before undergoing EEG examination and cognitive assessment using the Montreal Cognitive Assessment (MoCA) scale. The study flowchart is presented in Figure 1.

### Inclusion and exclusion criteria

PT group: the inclusion criteria for this group were a confirmed diagnosis of AF via electrocardiogram (ECG) or ambulatory ECG, with diagnoses verified by cardiovascular specialists. Exclusion criteria included patients with (1) comorbidities such as acute stroke, severe liver and kidney failure, advanced malignant tumors, valvular heart disease, acute myocardial infarction, (2) inability to cooperate with the examination procedures; or (3) current use of medications known to affect EEG results.

HC group: healthy adults confirmed by a neurologist to be free from neurological disease and showing no pathologic changes on cranial computed tomography or magnetic resonance imaging scans were eligible for enrollment. The exclusion criteria were identical to those of the PT group.

### Clinical data collection

Essential demographic variables (gender, age, height, weight, smoking and drinking history, and education level) were collected along with laboratory test results (serum albumin, fasting glucose, blood creatinine, and blood uric acid) and echocardiographic metrics [ejection fraction (EF), left atrial diameter, aortic root diameter]. Medical histories, including conditions such as hypertension, diabetes mellitus, hyperlipidemia, heart failure, and habits such as smoking and alcohol consumption, were also recorded.

### Assessment of cognitive function

Cognitive function was evaluated for all participants using the MoCA scale. The total score of MoCA is 30, with a score below 22 indicating cognitive impairment. This study used the MoCA^®^ version 8.1, which is based on a sample analysis of 3,097 subjects, establishing a threshold score of ≥22 as normal cognition. This scale covers six cognitive domains: executive function, visuospatial ability, short-term memory, language, attention and numeracy, and orientation.

### EEG data acquisition

EEG recordings were conducted in a quiet environment free from electromagnetic interference using a Nicolet V5.95 system (Natus Medical Inc.). A 21-channel montage was employed according to the international 10–20 system. The electrodes were positioned at the following bilateral sites: frontopolar (Fp1, Fp2), frontal (F3, F4), frontotemporal (F7, F8), central (C3, C4), mid-temporal (T3, T4), posterior-temporal (T5, T6), parietal (P3, P4), occipital (O1, O2), and sphenoidal (F9, F10). Midline electrodes were placed at Fz, Cz, and Pz, whereas A1 and A2 were used as earlobe electrodes. Signals were sampled at 500 Hz with an online bandpass filter of 0.5–70 Hz. Each recording session lasted 30 min, during which patients were instructed to remain awake, perform an eye-open/eye-close task, and avoid physical movement and blinking to reduce artifacts. Data quality was monitored in real-time by trained personnel.

### EEG signal preprocessing

EEG signals were preprocessed using MATLAB and the EEGLAB toolbox. Channel coordinates were mapped using the standard 10–5 system template. The preprocessing steps were as follows: (1) removal of A1, A2, and EKG channels, which were connected during acquisition but excluded from analysis; (2) relabeling of sphenoidal electrodes (ROC, LOC) as F9 and F10 for consistency; (3) band-pass filtering (1–45 Hz) and notch filtering (48–52 Hz); (4) segmentation into 2-s epochs with baseline correction; (5) detection and interpolation of bad channels due to flat signals, high noise, or extreme amplitudes; (6) re-referencing to the common average; (7) manual rejection of heavily contaminated segments; (8) application of independent component analysis to remove ocular and muscular artifacts; and (9) exclusion of epochs exceeding ±100 μV. The resulting preprocessed data were saved as “cleaned_data” for further analysis.

### EEG data analysis

EEG data were analyzed using the following metrics: absolute PSD, relative band power, functional connectivity (coherence and wPLI), PAC, and EnSA. Detailed computational steps and formulas are provided in Supplementary Methods.

### Statistical analysis

In the clinical data analysis, variables adhering to a normal distribution were presented as mean ± standard deviation and compared between the two groups using independent samples *t*-tests. Non-normally distributed continuous variables were expressed as medians with interquartile ranges and analyzed using the Mann–Whitney *U*-test. Categorical variables were expressed as percentages (frequencies) and compared using the χ^2^ test. EEG metrics were also compared between the PT and HC groups using independent samples *t*-tests, with the false discovery rate (FDR) correction applied to adjust for multiple comparison errors. In addition to *p*-values, *t*-values, effect sizes (Cohen's *d*), 95% confidence intervals, and statistical power were also reported for each comparison. Comprehensive statistical details are provided in Supplementary Tables S1–S6. Correlations between MoCA scores and EEG metrics were assessed using Spearman's test. Mediation analysis, considering AF as the independent variable (X), EEG metrics as the mediator (M), and MoCA scores as the dependent variable (Y), was conducted using PROCESS Version 4.2 (Model 4) by Andrew F. Hayes, with adjustments for potential confounders such as EF, gender, and occupation. The Bootstrap method, employing 1,000 samples, was utilized to estimate indirect effects and their confidence intervals. All statistical analyses and graph plotting were performed using SPSS 23.0, GraphPad Prism 9.5.1, and OriginPro 2021, with a threshold for statistical significance set at *p* < 0.05.

## Results

### Basic information

A total of 120 participants were included in this study, and their basic information is summarized in Table 1. There were 60 males and 60 females, with a median age of 71 (68–75.75) years. The PT group included 60 patients (34 males, 56.7%), with a median age of 72 (68.25–76) years, whereas the HC group had 60 participants (26 males, 43.3%), with a median age of 71 (67–74.75) years. The differences in age and gender between the two groups were not statistically significant. The proportion of workers was significantly lower in the PT group than in the HC group (26.7 vs. 45.0%; *p* = 0.036).

**Table 1:** Basic information of the study subjects.

**Variable**	**Group**	**HC**	**PT**	***p*-Value**
Age (years)		71 (67–74.75)	72 (68.25–76)	0.285
Gender	Male	26 (43.3%)	34 (56.7%)	0.144
	Female	34 (56.7%)	26 (43.3%)	
Education	Below junior high school	38 (63.3%)	43 (71.7%)	0.33
	Above junior high school	22 (36.7%)	17 (28.3%)	
Occupation	Farmer	33 (55.0%)	44 (73.3%)	0.036^*^
	Employee	27 (45.0%)	16 (26.7%)	
Hypertension	No	27 (45.0%)	28 (46.7%)	0.855
	Yes	33 (55.0%)	32 (53.3%)	
Diabetes	No	50 (83.3%)	49 (81.7%)	0.81
	Yes	10 (16.7%)	11 (18.3%)	
Hyperlipidemia	No	43 (71.7%)	56 (93.3%)	0.002^**^
	Yes	17 (28.3%)	4 (6.7%)	
Hyperuricemia	No	45 (75.0%)	40 (66.7%)	0.315
	Yes	15 (25.0%)	20 (33.3%)	
Smoking	No	54 (90.0%)	51 (85.0%)	0.408
	Yes	6 (10.0%)	9 (15.0%)	
Drinking	No	51 (85.0%)	57 (95.0%)	0.068
	Yes	9 (15.0%)	3 (5.0%)	
Albumin (g/L)		41.6 (38.7–44.9)	39.35 (36.63–41.38)	0.002^**^
Anticoagulants	No	57 (95.0%)	7 (11.7%)	<0.001^**^
	Yes	3 (5.0%)	53 (88.3%)	
Antiplatelet agents	No	20 (33.3%)	54 (90.0%)	<0.001^**^
	Yes	40 (66.7%)	6 (10.0%)	
Diabetes drugs	No	51 (85.0%)	50 (83.3%)	0.803
	Yes	9 (15.0%)	10 (16.7%)	
Antihypertensive	No	26 (43.3%)	25 (41.7%)	0.853
	Yes	34 (56.7%)	35 (58.3%)	
Statin	No	19 (31.7%)	26 (43.3%)	0.187
	Yes	41 (68.3%)	34 (56.7%)	
NYHA	1	58 (96.7%)	24 (40%)	<0.001^**^
	2	1 (1.7%)	12 (20%)	
	3	0 (0%)	16 (26.7%)	
	4	1 (1.7%)	8 (13.3%)	
MoCA		23 (20–25)	19 (14.25–22)	<0.001^**^
Aortic root diameter (mm)		29 (27–32)	31 (31–34)	0.017^*^
Glomerular filtration rate		94.4 (75.2–123.04)	91.71 (70.74–105.93)	0.17
Left atrial diameter (mm)		36.58 ± 5.37	47.22 ± 8.81	0.01^*^
EF		0.63 ± 0.06	0.52 ± 0.11	<0.001^**^
Body mass index		23.30 ± 3.72	23.33 ± 3.26	0.203

HC, healthy control group; PT, AF patient group; MoCA, montreal cognitive assessment; EF, ejection fraction; NYHA, The New York Heart Association.

* *p* < 0.05;

** *p* < 0.01.

The albumin levels and the prevalence of hyperlipidemia were significantly lower in the PT group than in the HC group (both *p* = 0.002). Although the proportion of patients with a history of alcohol consumption was lower in the PT group, this difference did not reach statistical significance (*p* = 0.068). No significant differences were observed between the two groups regarding history of hypertension, diabetes, hyperuricemia, antihypertensive medication, glucose-lowering medication, smoking, education level, statin use, body mass index, and glomerular filtration rate.

The MoCA scores were significantly lower in the PT group than in the HC group (*p* < 0.001). Anticoagulant use was significantly higher in the PT group compared to the HC group (*p* < 0.001), indicating that standardized anticoagulant therapy was implemented for AF patients. Cardiac decompensation was significantly more prevalent in the PT group than in the HC group (*p* < 0.001). Additionally, the PT group showed significantly lower EF values (*p* < 0.001) and significantly higher left atrial diameter (*p* = 0.01) and aortic root diameter (*p* = 0.017) compared to the HC group. These results indicate structural and functional cardiac abnormalities in AF patients.

### Differential analysis of EEG between the two groups

#### Absolute PSD analysis

**Figure 2:**
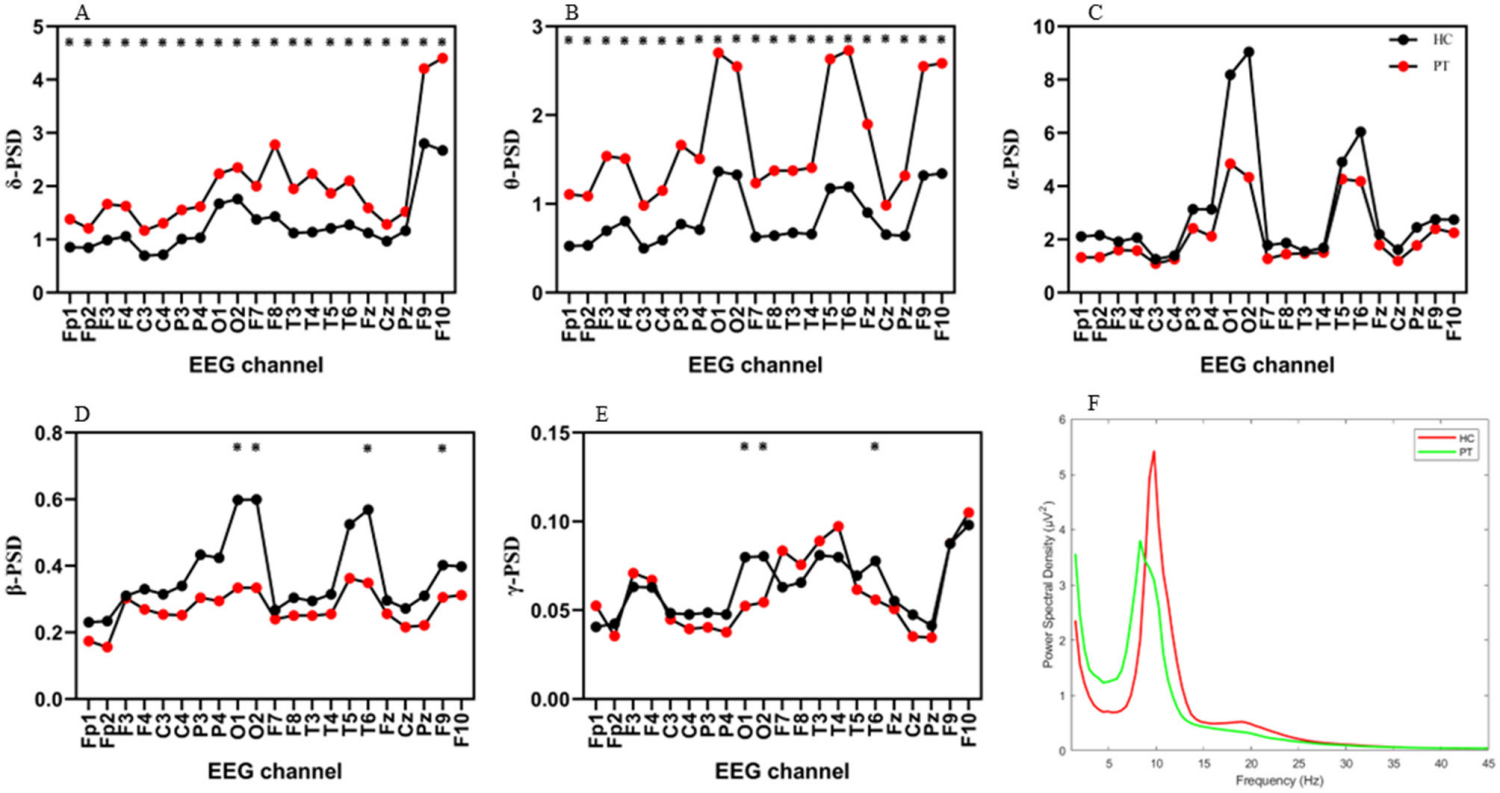
Differences and trends in absolute PSD across EEG channels in the PT and HC groups. **(A)** δ-PSD, **(B)** θ-PSD, **(C)** α-PSD, **(D)** β-PSD, **(E)** γ-PSD, **(F)** Average PSD spectrum. PSD, power spectral density. *p < (FDR-corrected). Detailed statistical analyses are shown in Supplementary Tables S1a–e.

The average PSD spectrogram, illustrated in Figure 2F, showed that the PT group exhibited increased activity in the lower frequency bands (δ and θ waves) and significantly decreased activity in the higher frequency bands (α and β waves). The PSD values in the δ and θ bands were significantly higher in the PT group compared to the HC group, with substantial differences noted in all channels following FDR correction. In contrast, the PSD values in the β and γ bands were notably lower in the PT group than in the HC group, particularly in localized regions such as O1, O2, T6, and F9 (Figure 2).

#### Relative band power analysis

As shown in Supplementary Figure S1, the relative band power in the δ and θ frequency bands was significantly higher in the PT group than in the HC group across all channels, whereas in the α and β frequency bands, the relative band power was significantly lower than in the PT group. No significant difference was observed in the γ band.

#### FC analysis

**Figure 3:**
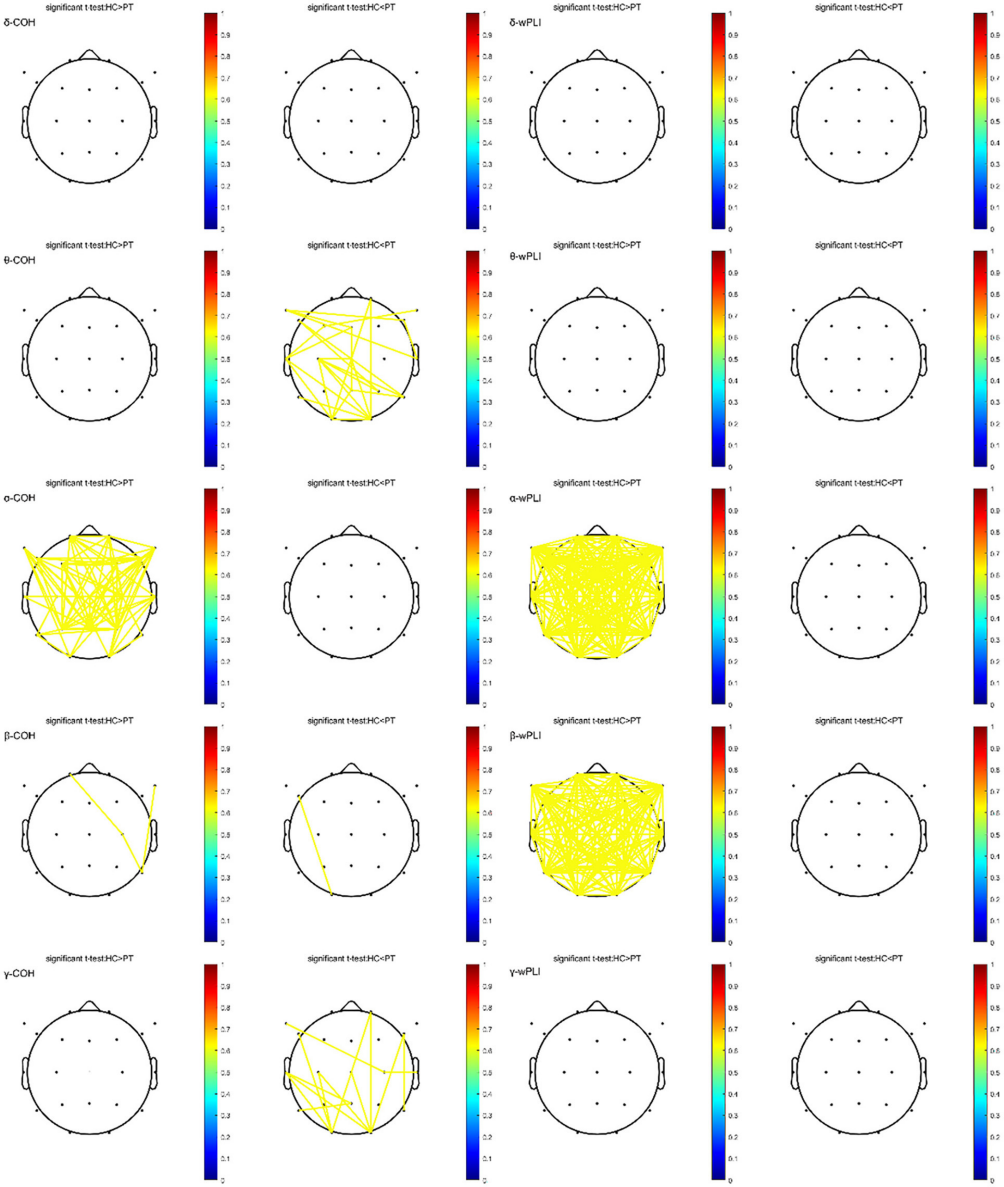
Topographic maps showing the COH (left side) and wPLI (right side) in the δ, θ, α, β, and γ frequency bands in the PT and HC groups after FDR correction. The left side of the figure indicates “HC>PT,” where FC strength in the HC group exceeds that in the PT group. The right side indicates “PT>HC,” where FC strength in the PT group exceeds that in the HC group. The color bar (from blue to red) reflects the significance level (from low to high). Yellow lines represent connections with significant differences, with line intensity corresponding to the significance level. COH, coherence; wPLI, weighted phase lag index. Detailed statistical analyses are shown in Supplementary Tables S3, S4.

In the α frequency band, coherence (COH) and wPLI were significantly lower in the PT group than in the HC group, as was the wPLI in the β frequency band. Conversely, the PT group demonstrated significantly higher COH in the θ-band between local channel pairs than the HC group. The COH in the β-band exhibited inconsistent differences between the two groups depending on the channel pair, whereas in localized regions, the γ-band COH in the PT group was significantly higher than in the HC group for specific channel pairs. However, neither δ-band COH nor wPLI showed significant differences between the groups, as illustrated in Figure 3.

#### CFC analysis

**Figure 4:**
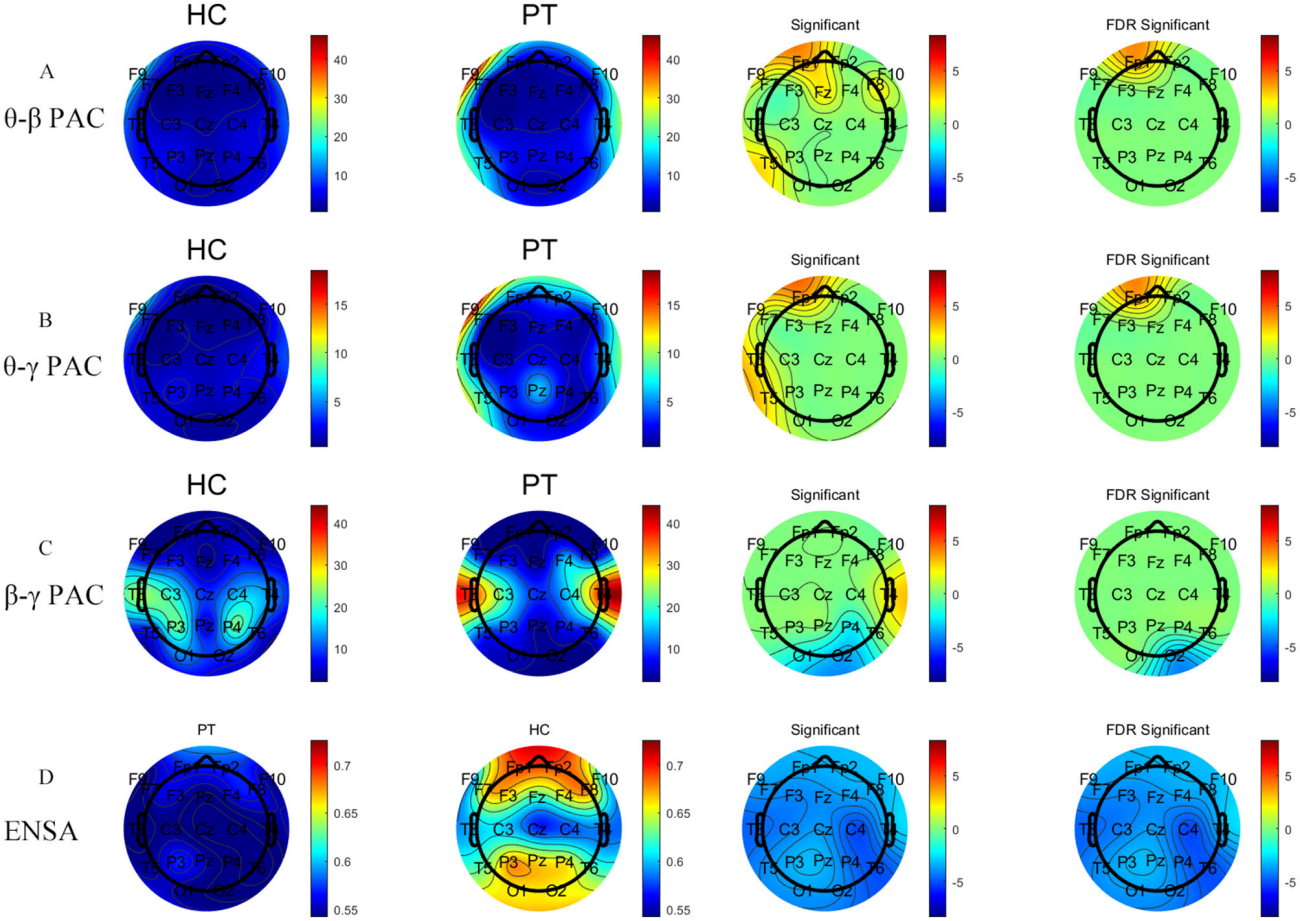
Comparative analysis of PAC metrics and EnSA values between the PT and HC groups. Panels **(A–C)** represent analysis results of θ–β PAC, θ–γ PAC, and β–γ PAC, respectively, whereas Panel **(D)** shows the results of EnSA. The first two columns show the topographical distributions for the HC and PT groups, whereas the last two columns display group comparisons, including statistically significant regions (FDR-corrected). The two left color bars indicate metric values (red: higher, blue: lower). The two right color bars show group differences (red: significantly higher metric values in the PT group vs. the HC group, blue: significantly lower metric values in the PT group vs. the HC group). Detailed statistical analyses are shown in Supplementary Tables S5, S6.

Significant differences were observed in the θ–β, θ–γ, and β–γ phase-amplitude coupling (PAC) patterns between the PT and HC groups. Specifically, the θ–β PAC demonstrated significantly higher coupling strengths in the PT group than in the HC group at the FP1 and F9 channels, and this difference was maintained after FDR correction. Similarly, in the θ–γ PAC, the coupling strength at the FP1 channels was significantly elevated in the PT group. In the β–γ PAC, the coupling strength at the right occipital (O2) channel was significantly lower in the PT group compared to the HC group (Figures 4A–C).

#### EnSA analysis

The HC group exhibited significantly higher EnSA values than the PT group, particularly in the prefrontal and posterior occipital regions (Figure 4D). This significant difference was consistent across all channels following FDR correction, indicating a pronounced reduction in the complexity of brain activity in AF patients.

### Mediating effects of EEG on the risk of cognitive dysfunction

After adjusting for potential confounders, including EF, gender, and occupation, the mediation analysis revealed that θ–β PAC partially mediated the relationship between AF and MoCA scores, with both the direct effect (β = 3.115, *p* < 0.001) and the indirect effect (β = −0.314, *p* = 0.032) being significant. These findings highlight θ–β PAC as a crucial mechanism contributing to AF-related cognitive impairment (Table 2).

**Table 2:** Total, direct, and indirect effects of different groups on MoCA scores via θ–β PAC.

**Effect type**	**β**	**SE**	***p*-Value**	**95%CI LB**	**95%CI UB**
Total	2.801	0.715	<0.001^**^	0.850	4.437
Direct	3.115	0.676	<0.001^**^	1.790	4.441
Indirect	−0.314	0.239	0.032^*^	−0.94	−0.004

θ-β PAC, theta-beta phase-amplitude coupling; β, regression coefficient; SE, standard error; 95% CI LB/UB, 95% confidence interval lower bound/upper bound.

** *p* < 0.01,

* *p* < 0.05.

### Association between EEG indicators and MoCA scores

As shown in Supplementary Figure S2, several EEG indicators were significantly correlated with MoCA scores. Specifically, θ–β PAC, θ–α PAC, δ–α PAC, δ–β PAC, δ–γ PAC, δ-PSD, and θ-PSD were weakly to moderately negatively correlated with MoCA scores. In contrast, β-PSD, α-COH, β-wPLI, α-wPLI, β-γ PAC, and EnSA exhibited significant positive correlations with MoCA scores.

## Discussion

This study systematically analyzed the resting-state EEG characteristics of patients with AF and HCs, exploring the relationship between EEG metrics and cognitive dysfunction. Our findings indicated that AF patients exhibited poorer performance in cognitive function assessments, with MoCA scores significantly lower than HCs. Furthermore, a strong association was observed between EEG features and cognitive dysfunction.

Our analysis identified an increasing trend in δ- and θ-wave PSD in AF patients, whereas α- and β-wave PSD showed a decreasing trend, as analyzed through both absolute PSD and relative band power. These changes align with the EEG patterns observed in patients with cognitive impairment, characterized by elevated low-frequency (δ and θ) power and diminished high-frequency (α and β) power. Correlation analysis further revealed significant negative correlations between δ-PSD and θ-PSD with MoCA scores, suggesting a decline in the brain's information processing efficiency.

Previous studies have reported that increased low-frequency activity (e.g., δ- and θ-waves) is strongly associated with cognitive decline, and in particular, a reduction in α-wave activity is associated with cognitive deterioration mediated by cholinergic deficit (20, 24–26). These findings reinforce the relationship between AF and cognitive decline.

Based on the PSD findings, the complexity of EEG signals was quantified by EnSA. The PT group showed significantly lower EnSA values across all channels than HCs, indicating more regular and less complex brain activity in AF patients. This finding is consistent with previous studies that associated reduced complexity (low entropy values) with cognitive decline (23, 27). Furthermore, EnSA values were weakly to moderately positively correlated with MoCA scores, confirming the relationship between reduced EEG complexity and cognitive dysfunction.

The analysis of FC indicated that FC in the α and β frequency bands was significantly lower, whereas that in the θ and γ frequency bands was significantly higher in the localized brain regions of AF patients compared to HCs, suggesting functional impairment in the brain network of AF patients. These changes, particularly reductions in α and β FC, have been widely reported in various neurodegenerative disorders and are considered an important marker of cognitive impairment (21, 28–37). In the present study, FC in the α and β frequency bands was positively correlated with MoCA scores, suggesting that the stronger the connectivity in these bands, the better the cognitive function. Conversely, θ and γ FC were significantly higher in the PT group, which is consistent with the findings by Iyer et al. (38), who observed similar patterns in patients with cognitive impairment due to Parkinson's disease. Increased connectivity in θ and γ frequency bands has been linked to increased anxiety and cognitive impairment symptoms. Excessive network activity may represent an adaptive cortical response for overall cognitive efficiency (39).

CFC analysis provided further evidence of abnormal network integration in AF patients. The significant increase in θ and γ FC in localized brain regions of AF patients was accompanied by a notable enhancement in θ–γ PAC. Additionally, θ–β PAC was significantly enhanced in localized brain regions. It is often considered a key marker of informational interactions between global and local networks of neurons (40), facilitating interaction and synchronization between these processes (22). Notably, Gong et al. (41) emphasized that the modulation level (slow 5/6) integrates information from higher frequencies and modulates faster spontaneous slow oscillations and that ultraslow oscillations achieve adaptive regulation by modulating higher-frequency oscillations. The pattern of enhanced θ–β/θ–γ PAC and attenuated β–γ PAC observed in our study aligns with this cross-scale modulation framework, suggesting that the changes in PAC and connectivity observed in patients with AF may reflect a disruption in temporal integration in this nested oscillatory framework. These results imply a synergistic role for the θ, β, and γ bands in brain network function. Specifically, increased FC may represent enhanced information transfer between localized brain regions, whereas enhanced PAC suggests greater cross-frequency integration within brain regions. Together, these alterations may act as a compensatory mechanism to address functional impairments of brain networks in AF patients. However, the negative correlation between EEG metrics and MoCA scores indicates that this compensation might reflect “over-mobilization,” a state associated with cognitive impairment. Additionally, the significant reduction in right occipital β–γ PAC may be related to deficits in visual working memory, further supporting the specific impact of AF on cognitive function in localized brain regions.

We utilized EEG metrics as mediating variables to explore the underlying relationship mechanisms between AF and cognitive dysfunction. The results indicated that θ–β PAC partially mediated the relationship between AF and the risk of cognitive impairment, highlighting θ–β PAC as a potential key mechanism underlying AF-associated cognitive dysfunction. In AF patients, enhanced θ–β PAC may indicate abnormal network integration, potentially exacerbating cognitive impairment risk. Although the mediating effect of θ–β PAC was significant, it accounted for only part of it, suggesting that AF may influence cognitive function through additional mechanisms. In particular, AF may contribute to MoCA decline through hemodynamic, embolic, and inflammatory mechanisms. Specifically, chronic cerebral hypoperfusion, silent microemboli, and systemic inflammation lead to endothelial dysfunction, blood-brain barrier disruption, and the development of white-matter hyperintensities (42, 43). These neurovascular insults, combined with EEG-derived disruptions in FC, EnSA, and PAC, contribute to cognitive decline in AF patients, even in the absence of overt stroke.

Despite these promising findings, several limitations should be acknowledged. First, the relatively small sample size limits the reproducibility and generalizability of our findings, echoing ongoing concerns regarding statistical power in neuroimaging research (44). Although FDR correction and confounder adjustment were applied, this cohort was insufficient for robust internal validation. Future studies should confirm our results in larger, independent cohorts. Second, the reliability of EEG-based metrics must be interpreted with caution. Previous studies indicate that metric reliability varies across modalities and preprocessing pipelines (45, 46). Although our data were recorded under consistent conditions, we did not assess test-repetition reliability. Future studies should include repeated measurements to improve the stability and clinical interpretability of EEG biomarkers. Finally, although this study focused on EEG metrics, cognitive dysfunction in AF is multifactorial. Integrating EEG with neuroimaging and biomarker analyses may provide a more comprehensive understanding of the mechanisms.

## Conclusion

The present study analyzed the resting-state EEG characteristics of patients with AF and their relationship with cognitive dysfunction. The PT group exhibited increased activity in low-frequency bands (δ, θ) and decreased activity in high-frequency bands (α, β), along with reduced complexity of brain activity. Furthermore, significant differences were observed in FC and CFC patterns between AF patients and HCs. Mediation analysis revealed a significant mediating role of θ–β PAC in the relationship between AF and cognitive dysfunction. This suggests that EEG metrics may be a critical mechanism through which AF affects cognitive function. These findings provide a novel perspective on cognitive assessment in patients with AF and support the potential application of EEG for evaluating AF-related cognitive dysfunction.

## Data Availability

The original contributions presented in the study are included in the article/Supplementary material, further inquiries can be directed to the corresponding authors.
