# Supplementary material for: Resting-state EEG alterations and cognitive impairment in atrial fibrillation: insights into neural biomarkers and functional connectivity
Source: Front Neurol. 2025 Jun 19;16:1583715. doi: 10.3389/fneur.2025.1583715 (PMC12221916; doi:10.3389/fneur.2025.1583715)
Supplement: Supplementary file 1 [file Supplementary_file_1.docx]

Supplementary Appendix

Supplementary Methods

M1. Power spectral density (PSD): ........................................................................................ p.3

M2. Relative band power : ..................................................................................................... p.3

M3. Functional connectivity (Coherence & wPLI): .............................................................. p.3

M4. Phase–amplitude coupling (PAC): ................................................................................. p.4

M5. Sample entropy (EnSA): ....................................... ........................................................ p.4

M6. References ...................................................................................................................... p.5

Supplementary Figures

Figure S1. Relative band power analysis: .............................................................................. p.6

Figure S2. Association between EEG indicators and MoCA scores ...................................... p.8

Supplementary Tables

Table S1. Absolute PSD: ……………………………............................................................ p.9

Table S1a. Absolute PSD – Delta band ............................................................................ p.10

Table S1b. Absolute PSD – Theta band ........................................................................... p.10

Table S1c. Absolute PSD – Alpha band .......................................................................... p.11

Table S1d. Absolute PSD – Beta band ............................................................................ p.11

Table S1e. Absolute PSD – Gamma band........................................................................ p.12

Table S2. Relative band power: full statistical results by region and frequency .................. p.13

Table S2a. Relative Band Power – Delta band ............................................................... p.13

Table S2b. Relative Band Power – Theta band .............................................................. p.14

Table S2c. Relative Band Power – Alpha band .............................................................. p.15

Table S2d. Relative Band Power – Beta band ................................................................ p.15

Table S2e. Relative Band Power – Gamma band ........................................................... p.16

Table S3. Functional connectivity (coherence): ................................................................... p.17

Table S3a. alpha_coh_HC_PT ..................................................................................... p.17

Table S3b. alpha_coh_PT_HC ......................................................................................... p.22

Table S3c. beta_coh_HC_PT ........................................................................................... p.27

Table S3d. beta_coh_PT_HC ........................................................................................... p.32

Table S3e. delta_coh_HC_PT............................................................................................ p.37

Table S3f. delta_coh_PT_HC ........................................................................................... p.42

Table S3g. theta_coh_HC_PT .......................................................................................... p.47

Table S3h. theta_coh_PT_HC .......................................................................................... p.52

Table S3i. gamma_coh_HC_PT ....................................................................................... p.57

Table S3j. gamma_coh_PT_HC ....................................................................................... p.62

Table S4. Functional connectivity (wPLI): .......................................................................... p.68

Table S4a: alpha_wpli_HC_PT.................................................................................... p.68

Table S4b: alpha_wpli_PT_HC.................................................................................... p.73

Table S4c: beta_wpli_HC_PT...................................................................................... p.78

Table S4d: beta_wpli_PT_HC...................................................................................... p.83

Table S4e: delta_wpli_HC_PT..................................................................................... p.88

Table S4f: delta_wpli_PT_HC..................................................................................... p.93

Table S4g: theta_wpli_HC_PT..................................................................................... p.98

Table S4h: theta_wpli_PT_HC..................................................................................... p.103

Table S4i: gamma_wpli_HC_PT................................................................................. p.108

Table S4j: gamma_wpli_PT_HC.................................................................................. p.113

Table S5. Cross-frequency coupling (CFC): ………………………………......................... p.118

Table S5a. theta_beta PAC ................................................................................................ p.118

Table S5b. theta_gamma PAC ........................................................................................... p.119

Table S5c. beta_gamma PAC ............................................................................................ p.120

Table S6. Sample entropy (EnSA): …………………………………………....................... p.120

**Supplementary Methods:**

**M1: Power Spectral Density (PSD)** ^[1]^

During preprocessing, the data are divided into 2-second segments, with a sampling rate of 500 Hz and the original number of sampling points per segment is 1000; to facilitate the subsequent calculations, each segment is complemented with zero to 1024 time points, a Hanning window is added, the fast Fourier transform is performed for the 1024 time points, and then an average is made between segments, and finally the average of all frequency points within all frequencies of each band is used as the power spectral density of the band (μV ^2). The power spectral density equation is shown as follows.

$$\widehat{\text{P}}\left(\omega\right)=\frac{\Delta\text{t}}{\text{N}}\left|\sum_{\text{n}=0}^{\text{N}-1} \text{x}_{\text{n}}\text{e}^{-\text{j}2\text{πfn}}\right|^{2},-\frac{1}{2\Delta\text{t}}<\text{f}\leq\frac{1}{2\Delta\text{t}}$$

$\text{Xn}$is the EEG data, $\text{N}$ is the length of the original data segment, and $\Delta\text{t}$is the sampling interval. The PSD of each segment is obtained according to the above equation, and averaged between doing segments to obtain the average power spectral density of each frequency point. The power spectral density ($\text{μV}^{2}\text{/Hz}$) of each frequency segment is equal to the average value of all frequency points in its corresponding frequency range.

**M2: Relative band power** ^[2]^

Relative band power is defined by the ratio of the absolute power spectral density in the actual frequency band to the ratio of the total absolute spectral power. The formula is:

$$P_{\text{relative}}=\frac{\sum_{f=f_{1}}^{f_{2}} P\left( f \right)}{\sum_{f=f_{L}}^{f_{H}} P\left( f \right)}$$

$\boldsymbol{f}\boldsymbol{1,f}\boldsymbol{2}$**:** the lower and upper frequency bounds of a specific band (e.g., alpha, beta, or theta band).

$\boldsymbol{fL,fH}$**:** the lower and upper bounds of the full spectrum used for total power calculation (e.g., 1–45 Hz).

$\boldsymbol{P(f)}$**:** the power spectral density at frequency f.

**M3: Functional Connectivity (FC)**

1. Coherence (COH) ^[3]^

Each segment is complemented with zero to 1024 time points, plus the hanning window (hanning), the Fourier transform of 1024 time points, and then do the average between the segments to get the power spectrum of each electrode, as well as the cross-power spectrum of the two electrodes at each frequency point, using the power of the two two electrodes at each frequency point and the corresponding cross-power spectrum, to calculate the coherence value of the two electrodes at each frequency point, the following is the formula for calculating the COH:

$$\text{C}\left(f\right)=\frac{\left|S_{xy}\left(f\right)\right|^{2}}{S_{xx}\left(f\right)S_{yy}\left(f\right)}$$

where signals$x\left(t\right)$and$y\left(t\right)$are the preprocessed data for any two electrodes, and $C\left(f\right)$denotes the coherence coefficients of signals$x\left(t\right)$and$y\left(t\right)$;$\left(f\right)$of SXY is the cross-spectrum of signals$x\left(t\right)$and$y\left(t\right)$; and$Sxx\left(f\right)$and$SYY\left(f\right)$are the autospectra of signals$x\left(t\right)$and$y\left(t\right)$, respectively.

1. Weighted Phase-Lag Index (wPLI) ^[4]^

For each frequency band, all segments are first spliced together, band-pass filtering and Hilbert transform are done to obtain the resolved signal time series of all electrodes, and after removing 10% of the time points before and after, they are re-split into segments, and the cross-power spectra of the phase difference between two and two electrodes are computed, and the wPLI is computed with the following formula:

$$\Phi\equiv\frac{\left|E\left\{\Im\left\{\text{X}\right\}\right\}\right|}{E\left\{\left|\Im\left\{\text{X}\right\}\right|\right\}}=\frac{\left|E\left\{\left|\Im\left\{\text{X}\right\}\right|\text{sgn}\left(\Im\left\{\text{X}\right\}\right)\right\}\right|}{E\left\{\left|\Im\left\{\text{X}\right\}\right|\right\}}$$

$\text{X}$is the cross power spectrum of the phase difference between the two electrodes, $\Im\left\{\text{X}\right\}$is the imaginary part of $\text{X}$, sgn is the sign function, and$\text{E}$is the expected mean value.

**M4: Cross-Frequency Coupling (CFC)** ^[5]^

CFC was assessed using phase–amplitude coupling (PAC), quantified by the Kullback–Leibler Modulation Index (KL-MI). Low-frequency phase and high-frequency amplitude signals were extracted using FIR filters with 2 Hz bandwidth, and KL-MI was computed for all phase–amplitude frequency pairs. Surrogate-based z-score normalization was applied to reduce spurious coupling.PAC was calculated using the following formula:

$$MI=1+\frac{1}{\log\left(n\right)}\sum_{k=1}^{n} P\left(k\right)\log\left(P\left(k\right)\right)$$

**where** *n* is the number of phase bins (typically 18), and *P(k)* is the normalized amplitude within the *k^th^* phase bin.

**M5: Sample Entropy (EnSA)** ^[6]^

The temporal complexity of EEG signals was measured using SampEn with parameters m = 2 and r = 0.2 × SD, computed on each 2-second segment. SampEn was calculated using the following formula:

$$\text{SampEn}\left(m,r,N\right)=-\ln\left[\frac{A^{m}\left(r\right)}{B^{m}\left(r\right)}\right]$$

where *B^m^(r)* is the probability that two sequences match for *m* consecutive points within a tolerance *r*, and *A^m^(r)* is the conditional probability that the same sequences match for *m+1* points.

**M6: References**

1、Di Y, An X, Zhong W, Liu S, Ming D. The Time-Robustness Analysis of Individual Identification Based on Resting-State EEG. Front Hum Neurosci. 2021Sep 13;15:672946. doi: 10.3389/fnhum.2021.672946. PMID: 34588964; PMCID:

PMC8475761.

2、Wang R, Wang J, Yu H, Wei X, Yang C, Deng B. Power spectral density and coherence analysis of Alzheimer's EEG. Cogn Neurodyn. 2015 Jun;9(3):291-304. doi: 10.1007/s11571-014-9325-x. Epub 2014 Dec 16. PMID: 25972978; PMCID: PMC4427585.

3、Guo S, Lu J, Wang Y, Li Y, Huang B, Zhang Y, Gong W, Yao D, Yuan Y, Xia Y. Sad Music Modulates Pain Perception: An EEG Study. J Pain Res. 2020 Aug 7;13:2003-2012. doi: 10.2147/JPR.S264188. PMID: 32848448; PMCID: PMC7429222.

4、Vinck M, Oostenveld R, van Wingerden M, Battaglia F, Pennartz CM. An improved index of phase-synchronization for electrophysiological data in the presence of volume-conduction, noise and sample-size bias. Neuroimage. 2011 Apr 15;55(4):1548-65. doi: 10.1016/j.neuroimage.2011.01.055. Epub 2011 Jan 27. PMID:

21276857.

5、Combrisson E, Nest T, Brovelli A, Ince RAA, Soto JLP, Guillot A, Jerbi K. Tensorpac: An open-source Python toolbox for tensor-based phase-amplitude coupling measurement in electrophysiological brain signals. PLoS Comput Biol. 2020 Oct 29;16(10):e1008302. doi: 10.1371/journal.pcbi.1008302. PMID: 33119593; PMCID: PMC7654762.

6、Ahmadi S, Sepehri N, Wu C, Szturm T. Sample Entropy of Human Gait Center of Pressure Displacement: A Systematic Methodological Analysis. Entropy (Basel). 2018 Aug 6;20(8):579. doi: 10.3390/e20080579. PMID: 33265668; PMCID: PMC7513106.

**Supplementary Figures:**

**Figure S1:** **Relative band power analysis
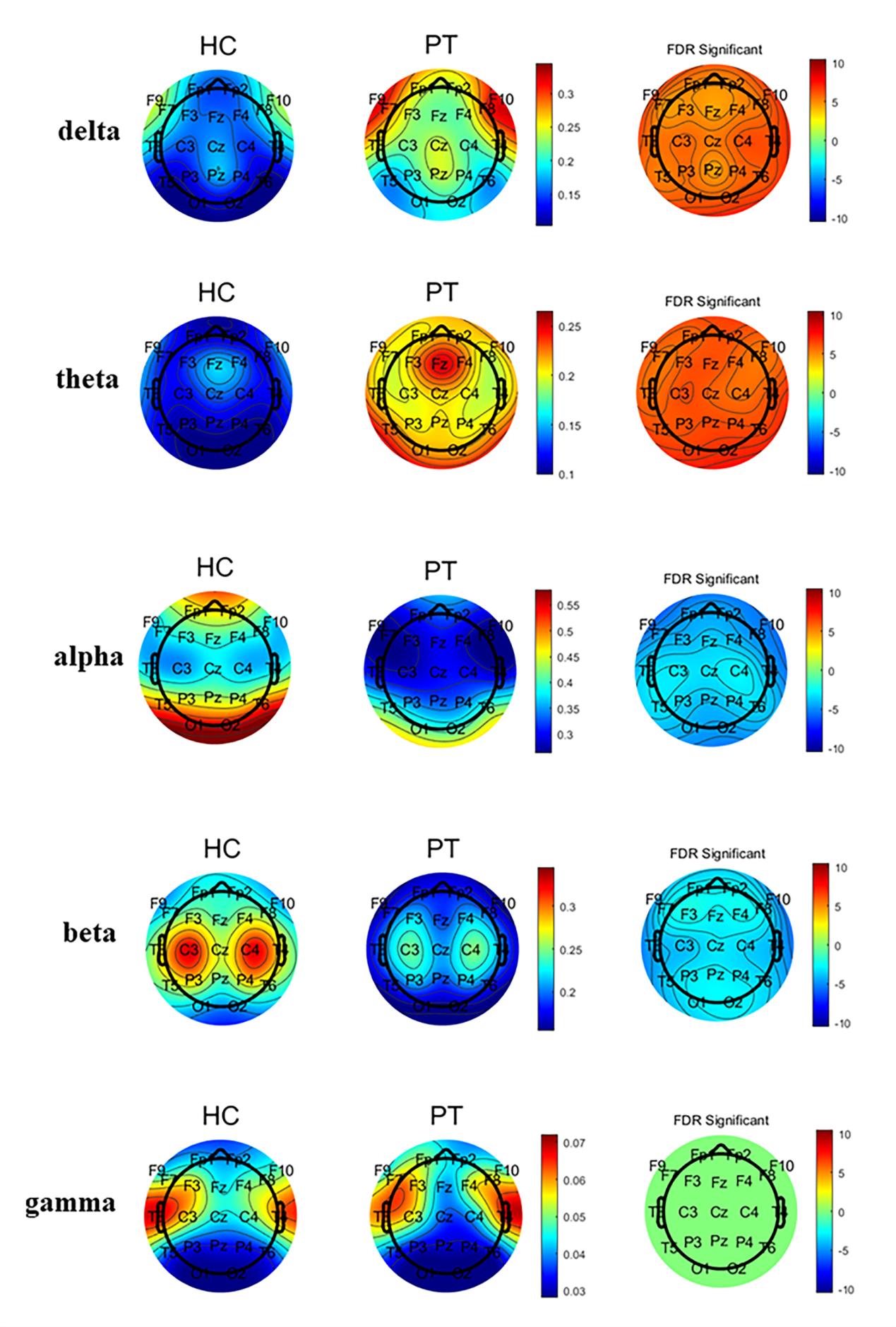
**

**Figure S1:** Topographic maps showing relative band power in different frequency bands in the HC and PT groups. For each frequency band, the topographic maps (from left to right) represent the HC group, the PT group, and the FDR-corrected statistical significance of the PSD differences between the two groups. The left color bar indicates PSD magnitude, with red representing higher values and blue representing lower values. The right color bar indicates the t-value of group differences (FDR-corrected *p<*0.05). A positive t-value suggests higher PSD in the PT group, whereas a negative t-value suggests the opposite. A t-value of 0 represents no significant difference between the two groups. See Supplementary Tables S2a to S2e for full statistical details.

**Figures S2: Association between EEG indicators and MoCA scores**


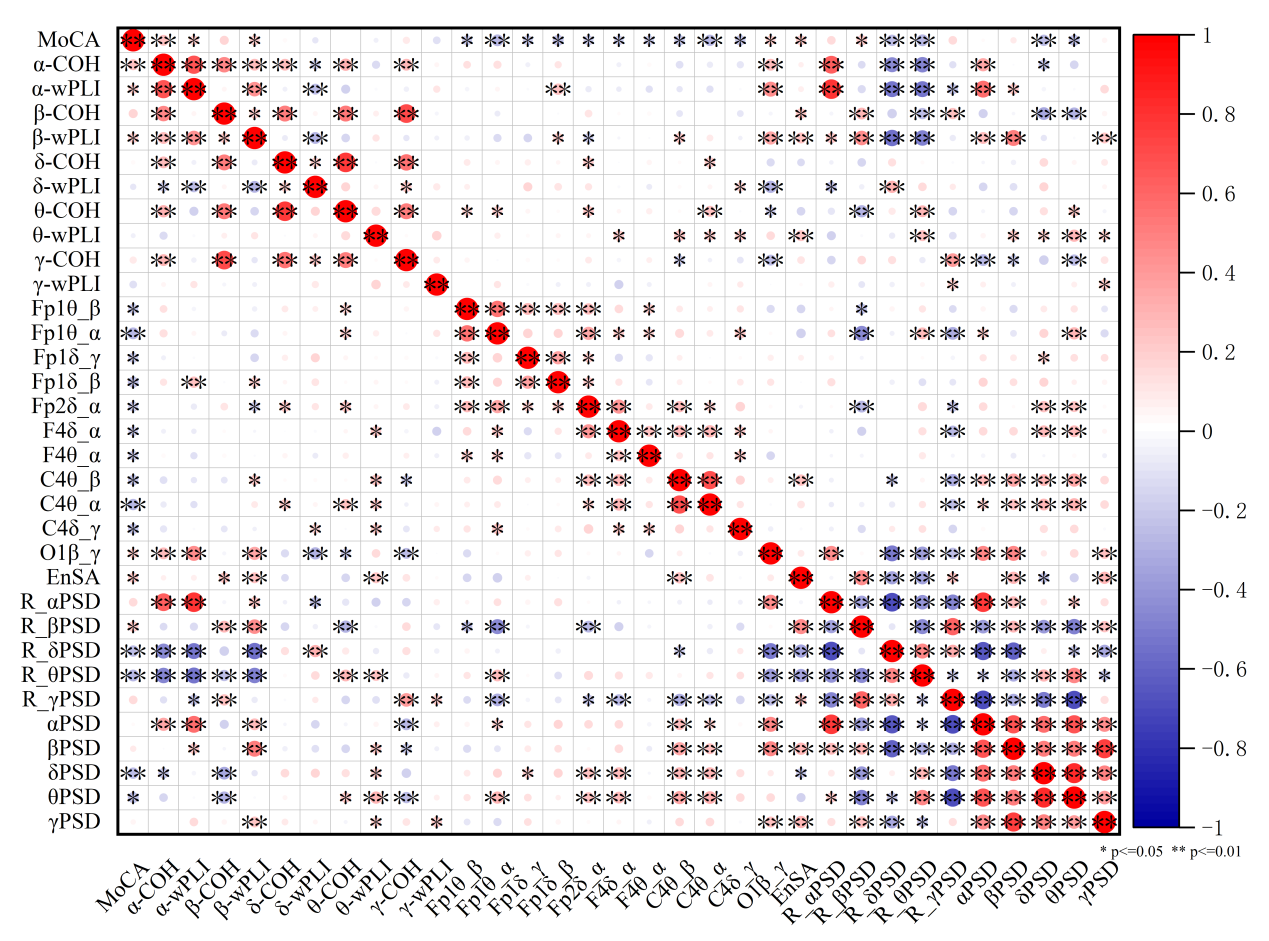


**Figures S2:** Correlation heatmap illustrating the relationships between EEG metrics and MoCA scores. Red circles indicate positive correlations, whereas blue circles represent negative correlations. The size of the circles reflects the strength of the correlation, and the color bar on the right shows the range of correlation coefficients. Statistical significance is marked with asterisks (**p*<0.05, ***p*<0.01). R_αPSD, R_βPSD, R_θPSD, and R_δPSD represent relative band power in the α, β, θ, and δ bands, respectively.

**Supplementary Tables**

Table S1: Absolute PSD

Table S1a. Absolute PSD: Delta Band

| channel | t_value | p_value | fdr_pvalue | CI_low | CI_high | statistical power | effect size |
| --- | --- | --- | --- | --- | --- | --- | --- |
| Fp1 | 2.784423 | 0.006248 | 0.011035 | 0.15104 | 0.894934 | 0.957947 | -0.50836 |
| Fp2 | 2.139503 | 0.034455 | 0.034455 | 0.02711 | 0.701417 | 0.752879 | -0.39062 |
| F3 | 3.416171 | 0.000872 | 0.004577 | 0.283639 | 1.065981 | 0.999998 | -0.6237 |
| F4 | 3.213044 | 0.001694 | 0.005809 | 0.218244 | 0.919401 | 0.999714 | -0.58662 |
| C3 | 3.034737 | 0.002962 | 0.007776 | 0.163394 | 0.777096 | 0.999968 | -0.55406 |
| C4 | 3.170959 | 0.001936 | 0.005809 | 0.221728 | 0.959254 | 1.000001 | -0.57894 |
| P3 | 2.826939 | 0.005522 | 0.011035 | 0.16378 | 0.929912 | 0.999125 | -0.51613 |
| P4 | 2.93921 | 0.003959 | 0.009239 | 0.190605 | 0.977831 | 0.997518 | -0.53662 |
| O1 | 2.149764 | 0.033615 | 0.034455 | 0.044313 | 1.079789 | 0.888824 | -0.39249 |
| O2 | 2.274181 | 0.024762 | 0.028889 | 0.076342 | 1.105089 | 0.820984 | -0.41521 |
| F7 | 2.718194 | 0.007554 | 0.011331 | 0.170311 | 1.084398 | 0.916926 | -0.49627 |
| F8 | 3.612838 | 0.000446 | 0.004473 | 0.610467 | 2.091438 | 0.999996 | -0.65961 |
| T3 | 2.568914 | 0.011448 | 0.015026 | 0.189326 | 1.463167 | 0.999276 | -0.46902 |
| T4 | 3.508365 | 0.000639 | 0.004473 | 0.477837 | 1.716307 | 0.999998 | -0.64054 |
| T5 | 2.753418 | 0.006831 | 0.011035 | 0.184726 | 1.131011 | 0.994055 | -0.5027 |
| T6 | 2.760522 | 0.006693 | 0.011035 | 0.23414 | 1.422631 | 0.997858 | -0.504 |
| Fz | 2.653315 | 0.009068 | 0.012696 | 0.119583 | 0.823276 | 0.984715 | -0.48443 |
| Cz | 2.460138 | 0.015337 | 0.018946 | 0.061791 | 0.571776 | 0.914428 | -0.44916 |
| Pz | 2.141648 | 0.034278 | 0.034455 | 0.027081 | 0.691711 | 0.785416 | -0.39101 |
| F9 | 3.268767 | 0.001415 | 0.005809 | 0.555421 | 2.262659 | 0.999365 | -0.59679 |
| F10 | 3.963884 | 0.000127 | 0.002662 | 0.86834 | 2.602095 | 0.999992 | -0.7237 |

Table S1b. Absolute PSD: Theta Band

| channel | tvalue | pvalue | fdr_pvalue | CI_low | CI_high | statistical power | effect size |
| --- | --- | --- | --- | --- | --- | --- | --- |
| Fp1 | 2.85266 | 0.005121 | 0.008926 | 0.178175 | 0.98707 | 0.999615 | -0.52082 |
| Fp2 | 2.653039 | 0.009075 | 0.012706 | 0.14085 | 0.970024 | 0.997699 | -0.48438 |
| F3 | 3.236676 | 0.00157 | 0.0086 | 0.325948 | 1.353432 | 0.999998 | -0.59093 |
| F4 | 3.025624 | 0.003046 | 0.0086 | 0.244238 | 1.169589 | 0.999974 | -0.5524 |
| C3 | 2.617698 | 0.010012 | 0.013141 | 0.117816 | 0.84985 | 0.999895 | -0.47792 |
| C4 | 2.262523 | 0.025495 | 0.026769 | 0.069656 | 1.047071 | 0.986897 | -0.41308 |
| P3 | 2.383178 | 0.01876 | 0.020735 | 0.150707 | 1.632148 | 0.99905 | -0.43511 |
| P4 | 2.754251 | 0.006815 | 0.010222 | 0.22448 | 1.373173 | 1 | -0.50286 |
| O1 | 2.843568 | 0.005259 | 0.008926 | 0.406241 | 2.269953 | 0.999781 | -0.51916 |
| O2 | 2.965146 | 0.003662 | 0.0086 | 0.405146 | 2.034388 | 0.999977 | -0.54136 |
| F7 | 3.170386 | 0.00194 | 0.0086 | 0.228356 | 0.988297 | 0.999992 | -0.57883 |
| F8 | 3.514085 | 0.000627 | 0.006579 | 0.320651 | 1.148622 | 0.999998 | -0.64158 |
| T3 | 2.826688 | 0.005526 | 0.008926 | 0.209608 | 1.190406 | 0.999908 | -0.51608 |
| T4 | 3.702788 | 0.000326 | 0.006579 | 0.348218 | 1.148869 | 1.000001 | -0.67603 |
| T5 | 2.962979 | 0.003686 | 0.0086 | 0.482961 | 2.429409 | 0.999958 | -0.54096 |
| T6 | 3.073578 | 0.002628 | 0.0086 | 0.546364 | 2.525589 | 1.000001 | -0.56116 |
| Fz | 2.514579 | 0.013264 | 0.016385 | 0.211323 | 1.777751 | 1 | -0.4591 |
| Cz | 2.405536 | 0.017702 | 0.020652 | 0.058711 | 0.605497 | 0.915186 | -0.43919 |
| Pz | 2.890327 | 0.004582 | 0.008926 | 0.213515 | 1.142729 | 0.999999 | -0.5277 |
| F9 | 2.210656 | 0.028988 | 0.028988 | 0.127959 | 2.327716 | 1.000001 | -0.40361 |
| F10 | 2.980315 | 0.003497 | 0.0086 | 0.417098 | 2.068963 | 0.999999 | -0.54413 |

Table S1c. Absolute PSD: Alpha Band

| channel | tvalue | pvalue | fdr_pvalue | CI_low | CI_high | statistical power | effect size |
| --- | --- | --- | --- | --- | --- | --- | --- |
| Fp1 | -2.07721 | 0.039952 | 0.209748 | -1.53572 | -0.03669 | 0.468869 | 0.379245 |
| Fp2 | -2.11721 | 0.036342 | 0.209748 | -1.6103 | -0.05382 | 0.485391 | 0.386548 |
| F3 | -0.83769 | 0.403897 | 0.565456 | -1.07522 | 0.43596 | 0.151137 | 0.152941 |
| F4 | -1.33616 | 0.184068 | 0.351402 | -1.19596 | 0.232278 | 0.253211 | 0.243948 |
| C3 | -0.66973 | 0.50434 | 0.593152 | -0.66187 | 0.327325 | 0.107783 | 0.122275 |
| C4 | -0.54557 | 0.586394 | 0.64812 | -0.59951 | 0.34053 | 0.080273 | 0.099606 |
| P3 | -1.04538 | 0.297985 | 0.481361 | -2.07155 | 0.640089 | 0.212357 | 0.190859 |
| P4 | -1.64554 | 0.10252 | 0.30756 | -2.24918 | 0.207644 | 0.28916 | 0.300433 |
| O1 | -2.2845 | 0.02413 | 0.209748 | -6.23705 | -0.44491 | 0.522479 | 0.41709 |
| O2 | -3.09527 | 0.002456 | 0.051581 | -7.71362 | -1.69453 | 0.672434 | 0.565117 |
| F7 | -1.70596 | 0.090647 | 0.30756 | -1.09733 | 0.081659 | 0.339137 | 0.311464 |
| F8 | -1.25455 | 0.212122 | 0.371213 | -1.07725 | 0.241679 | 0.226708 | 0.229048 |
| T3 | -0.23644 | 0.813504 | 0.813504 | -0.73438 | 0.577718 | 0.057253 | 0.043167 |
| T4 | -0.66333 | 0.508416 | 0.593152 | -0.69659 | 0.347019 | 0.105233 | 0.121106 |
| T5 | -0.49868 | 0.618933 | 0.649879 | -3.19677 | 1.910611 | 0.087248 | 0.091046 |
| T6 | -1.46387 | 0.145887 | 0.340403 | -4.37049 | 0.655289 | 0.243252 | 0.267266 |
| Fz | -0.96364 | 0.337198 | 0.505797 | -1.21208 | 0.418571 | 0.183757 | 0.175936 |
| Cz | -1.75213 | 0.082349 | 0.30756 | -0.90205 | 0.055137 | 0.389677 | 0.319894 |
| Pz | -1.50158 | 0.135876 | 0.340403 | -1.57269 | 0.216214 | 0.329409 | 0.274151 |
| F9 | -0.74878 | 0.455479 | 0.593152 | -1.26806 | 0.572213 | 0.179197 | 0.136708 |
| F10 | -1.36405 | 0.175147 | 0.351402 | -1.21811 | 0.224446 | 0.282532 | 0.249041 |

Table S1d. Absolute PSD: Beta Band

| channel | tvalue | pvalue | fdr_pvalue | CI_low | CI_high | statistical power | effect size |
| --- | --- | --- | --- | --- | --- | --- | --- |
| Fp1 | -1.70813 | 0.090241 | 0.169355 | -0.12191 | 0.008994 | 0.337734 | 0.311861 |
| Fp2 | -2.42499 | 0.016824 | 0.058886 | -0.14121 | -0.01426 | 0.530247 | 0.442741 |
| F3 | -0.11072 | 0.91203 | 0.91203 | -0.14131 | 0.126345 | 0.052284 | 0.020214 |
| F4 | -1.43173 | 0.154866 | 0.232299 | -0.1438 | 0.023118 | 0.246797 | 0.261396 |
| C3 | -0.98496 | 0.326661 | 0.403522 | -0.18416 | 0.061814 | 0.230366 | 0.179828 |
| C4 | -1.67403 | 0.096774 | 0.169355 | -0.19171 | 0.016066 | 0.29083 | 0.305634 |
| P3 | -2.07638 | 0.04003 | 0.084063 | -0.25292 | -0.00599 | 0.489885 | 0.379093 |
| P4 | -2.19974 | 0.029774 | 0.078157 | -0.24589 | -0.01291 | 0.564985 | 0.401615 |
| O1 | -3.91392 | 0.000152 | 0.0016 | -0.3974 | -0.13037 | 0.960382 | 0.71458 |
| O2 | -4.12912 | 6.83E-05 | 0.001434 | -0.39193 | -0.13785 | 0.957942 | 0.75387 |
| F7 | -0.64843 | 0.517968 | 0.543867 | -0.10989 | 0.055678 | 0.108603 | 0.118386 |
| F8 | -1.23912 | 0.21776 | 0.28581 | -0.1379 | 0.031747 | 0.199166 | 0.226231 |
| T3 | -0.92589 | 0.356394 | 0.415793 | -0.13789 | 0.050029 | 0.193182 | 0.169043 |
| T4 | -1.59248 | 0.113953 | 0.184078 | -0.13327 | 0.014465 | 0.32632 | 0.290745 |
| T5 | -2.11906 | 0.036182 | 0.084063 | -0.31384 | -0.01063 | 0.639818 | 0.386885 |
| T6 | -3.20465 | 0.00174 | 0.012177 | -0.35629 | -0.08414 | 0.830927 | 0.585087 |
| Fz | -0.83848 | 0.403456 | 0.445925 | -0.1359 | 0.055049 | 0.186632 | 0.153085 |
| Cz | -1.28455 | 0.201465 | 0.28205 | -0.14238 | 0.030341 | 0.296384 | 0.234526 |
| Pz | -2.27843 | 0.0245 | 0.073499 | -0.16537 | -0.01158 | 0.592201 | 0.415983 |
| F9 | -2.75054 | 0.006888 | 0.036161 | -0.16615 | -0.02705 | 0.77059 | 0.502177 |
| F10 | -2.47587 | 0.01471 | 0.058886 | -0.15513 | -0.01725 | 0.652266 | 0.452029 |

Table S1e. Absolute PSD: Gamma Band

| channel | tvalue | pvalue | fdr_pvalue | CI_low | CI_high | statistical power | effect size |
| --- | --- | --- | --- | --- | --- | --- | --- |
| Fp1 | 1.099119 | 0.273954 | 0.501323 | -0.00952 | 0.033286 | 0.5331 | -0.20067 |
| Fp2 | -1.07074 | 0.28647 | 0.501323 | -0.01963 | 0.005851 | 0.166245 | 0.19549 |
| F3 | 0.61561 | 0.539337 | 0.70788 | -0.01724 | 0.032785 | 0.141564 | -0.11239 |
| F4 | 0.40135 | 0.688889 | 0.761403 | -0.01644 | 0.024801 | 0.081444 | -0.07328 |
| C3 | -0.51427 | 0.608028 | 0.709365 | -0.01682 | 0.009886 | 0.100747 | 0.093892 |
| C4 | -1.46466 | 0.145673 | 0.387195 | -0.01928 | 0.002885 | 0.257789 | 0.267409 |
| P3 | -1.45798 | 0.147503 | 0.387195 | -0.01931 | 0.002934 | 0.327337 | 0.266189 |
| P4 | -1.86752 | 0.06431 | 0.270102 | -0.02067 | 0.000606 | 0.486152 | 0.340961 |
| O1 | -3.80947 | 0.000223 | 0.003951 | -0.04186 | -0.01322 | 0.939993 | 0.695511 |
| O2 | -3.66174 | 0.000376 | 0.003951 | -0.0398 | -0.01186 | 0.956948 | 0.66854 |
| F7 | 1.14441 | 0.25477 | 0.501323 | -0.01494 | 0.055837 | 0.727341 | -0.20894 |
| F8 | 0.954624 | 0.34172 | 0.512579 | -0.01077 | 0.030811 | 0.189965 | -0.17429 |
| T3 | 0.341951 | 0.732996 | 0.769646 | -0.03814 | 0.054059 | 0.091875 | -0.06243 |
| T4 | 1.122223 | 0.264046 | 0.501323 | -0.01328 | 0.048016 | 0.276674 | -0.20489 |
| T5 | -1.00386 | 0.317501 | 0.512579 | -0.0232 | 0.00759 | 0.245373 | 0.183279 |
| T6 | -2.84304 | 0.005268 | 0.036873 | -0.03716 | -0.00665 | 0.840656 | 0.519065 |
| Fz | -0.52215 | 0.602547 | 0.709365 | -0.02152 | 0.01254 | 0.103067 | 0.095331 |
| Cz | -1.95643 | 0.052777 | 0.270102 | -0.02489 | 0.000151 | 0.394266 | 0.357194 |
| Pz | -1.49359 | 0.137951 | 0.387195 | -0.01558 | 0.002183 | 0.368466 | 0.272692 |
| F9 | 0.019848 | 0.984198 | 0.984198 | -0.01421 | 0.014502 | 0.050057 | -0.00362 |
| F10 | 0.649521 | 0.517264 | 0.70788 | -0.01424 | 0.028139 | 0.11215 | -0.11859 |

Table S2. Relative band power

Table S2a. Relative band power: Delta Band

| channel | tvalue | pvalue | fdr_pvalue | CI_low | CI_high | statistical power | effect size |
| --- | --- | --- | --- | --- | --- | --- | --- |
| Fp1 | 5.08067 | 1.42E-06 | 1.76E-06 | 0.053515 | 0.121877 | 0.999996 | -0.9276 |
| Fp2 | 5.10448 | 1.29E-06 | 1.69E-06 | 0.050298 | 0.11406 | 0.999989 | -0.93195 |
| F3 | 5.026064 | 1.8E-06 | 2.1E-06 | 0.040151 | 0.092361 | 0.999708 | -0.91763 |
| F4 | 5.164949 | 9.88E-07 | 1.38E-06 | 0.040874 | 0.091707 | 0.999942 | -0.94299 |
| C3 | 5.781434 | 6.17E-08 | 1.62E-07 | 0.046036 | 0.094002 | 0.999956 | -1.05554 |
| C4 | 6.325407 | 4.7E-09 | 2.47E-08 | 0.046504 | 0.088891 | 0.999996 | -1.15486 |
| P3 | 5.687618 | 9.52E-08 | 2E-07 | 0.046721 | 0.096633 | 0.999973 | -1.03841 |
| P4 | 5.747755 | 7.21E-08 | 1.68E-07 | 0.046306 | 0.094986 | 0.999883 | -1.04939 |
| O1 | 5.925093 | 3.16E-08 | 9.48E-08 | 0.050747 | 0.101696 | 0.999998 | -1.08177 |
| O2 | 6.190164 | 9E-09 | 3.78E-08 | 0.05674 | 0.11012 | 1.000002 | -1.13016 |
| F7 | 4.717128 | 6.62E-06 | 7.32E-06 | 0.045484 | 0.111304 | 0.999447 | -0.86123 |
| F8 | 5.573877 | 1.6E-07 | 2.8E-07 | 0.070549 | 0.148302 | 0.999971 | -1.01765 |
| T3 | 5.634998 | 1.21E-07 | 2.31E-07 | 0.045472 | 0.094749 | 0.999895 | -1.02881 |
| T4 | 6.375623 | 3.68E-09 | 2.47E-08 | 0.059933 | 0.113937 | 0.999998 | -1.16402 |
| T5 | 5.171265 | 9.62E-07 | 1.38E-06 | 0.035778 | 0.080183 | 0.999995 | -0.94414 |
| T6 | 6.006001 | 2.16E-08 | 7.56E-08 | 0.045529 | 0.090321 | 1 | -1.09654 |
| Fz | 4.506864 | 1.56E-05 | 1.56E-05 | 0.032798 | 0.084211 | 0.998944 | -0.82284 |
| Cz | 5.406293 | 3.41E-07 | 5.5E-07 | 0.044504 | 0.095951 | 0.999986 | -0.98705 |
| Pz | 4.593782 | 1.1E-05 | 1.15E-05 | 0.036422 | 0.091615 | 0.991918 | -0.83871 |
| F9 | 6.629813 | 1.06E-09 | 1.11E-08 | 0.061259 | 0.113441 | 1.000001 | -1.21043 |
| F10 | 6.715448 | 6.95E-10 | 1.11E-08 | 0.061855 | 0.113591 | 0.999997 | -1.22607 |

Table S2b. Relative band power: Theta Band

| channel | tvalue | pvalue | fdr_pvalue | CI_low | CI_high | statistical power | effect size |
| --- | --- | --- | --- | --- | --- | --- | --- |
| Fp1 | 5.823728 | 5.07E-08 | 7.1E-08 | 0.064054 | 0.130059 | 1 | -1.06326 |
| Fp2 | 6.265621 | 6.27E-09 | 1.46E-08 | 0.071054 | 0.136722 | 0.999994 | -1.14394 |
| F3 | 6.089922 | 1.45E-08 | 2.35E-08 | 0.060938 | 0.119665 | 0.99999 | -1.11186 |
| F4 | 5.725341 | 8E-08 | 1.05E-07 | 0.05519 | 0.113555 | 1.000004 | -1.0453 |
| C3 | 6.355882 | 4.05E-09 | 1.46E-08 | 0.056277 | 0.107216 | 0.999987 | -1.16042 |
| C4 | 5.550838 | 1.78E-07 | 1.96E-07 | 0.047763 | 0.100743 | 1 | -1.01344 |
| P3 | 6.211613 | 8.12E-09 | 1.71E-08 | 0.066684 | 0.129101 | 1.000005 | -1.13408 |
| P4 | 6.270807 | 6.11E-09 | 1.46E-08 | 0.064731 | 0.124484 | 1.000009 | -1.14489 |
| O1 | 6.614165 | 1.15E-09 | 1.2E-08 | 0.085575 | 0.158714 | 1.000003 | -1.20758 |
| O2 | 6.925133 | 2.44E-10 | 5.12E-09 | 0.086566 | 0.155901 | 0.99999 | -1.26435 |
| F7 | 5.43171 | 3.04E-07 | 3.19E-07 | 0.048905 | 0.105025 | 1.000003 | -0.99169 |
| F8 | 5.363548 | 4.12E-07 | 4.12E-07 | 0.046537 | 0.101013 | 0.999997 | -0.97925 |
| T3 | 5.598256 | 1.43E-07 | 1.67E-07 | 0.048799 | 0.102217 | 1 | -1.0221 |
| T4 | 6.295084 | 5.44E-09 | 1.46E-08 | 0.050552 | 0.096954 | 0.999992 | -1.14932 |
| T5 | 6.191747 | 8.93E-09 | 1.71E-08 | 0.079618 | 0.154492 | 0.999976 | -1.13045 |
| T6 | 6.460977 | 2.43E-09 | 1.46E-08 | 0.080951 | 0.152505 | 0.999974 | -1.17961 |
| Fz | 6.274435 | 6.01E-09 | 1.46E-08 | 0.067584 | 0.129918 | 0.999996 | -1.14555 |
| Cz | 5.867657 | 4.14E-08 | 6.2E-08 | 0.049788 | 0.100513 | 0.999996 | -1.07128 |
| Pz | 6.355981 | 4.05E-09 | 1.46E-08 | 0.066802 | 0.127266 | 0.999975 | -1.16044 |
| F9 | 6.155637 | 1.06E-08 | 1.86E-08 | 0.048013 | 0.093556 | 0.999993 | -1.12386 |
| F10 | 5.623988 | 1.27E-07 | 1.57E-07 | 0.048529 | 0.101277 | 1 | -1.02679 |

Table S2c. Relative band power: Alpha Band

| channel | tvalue | pvalue | fdr_pvalue | CI_low | CI_high | statistical power | effect size |
| --- | --- | --- | --- | --- | --- | --- | --- |
| Fp1 | -4.66777 | 8.11E-06 | 2.4E-05 | -0.20109 | -0.08129 | 0.994165 | 0.852214 |
| Fp2 | -4.69882 | 7.14E-06 | 2.4E-05 | -0.20046 | -0.08159 | 0.994868 | 0.857883 |
| F3 | -4.26281 | 4.09E-05 | 8.04E-05 | -0.16741 | -0.06121 | 0.966972 | 0.778278 |
| F4 | -4.255 | 4.21E-05 | 8.04E-05 | -0.16231 | -0.05921 | 0.97567 | 0.776853 |
| C3 | -3.05071 | 0.00282 | 0.002961 | -0.11095 | -0.02361 | 0.773657 | 0.55698 |
| C4 | -2.75006 | 0.006897 | 0.006897 | -0.10179 | -0.01656 | 0.702957 | 0.502089 |
| P3 | -3.70606 | 0.000322 | 0.000483 | -0.16153 | -0.04903 | 0.924376 | 0.676631 |
| P4 | -3.49546 | 0.000668 | 0.000935 | -0.15083 | -0.04174 | 0.869446 | 0.63818 |
| O1 | -4.37245 | 2.66E-05 | 6.21E-05 | -0.2043 | -0.07693 | 0.988709 | 0.798296 |
| O2 | -4.63911 | 9.12E-06 | 2.4E-05 | -0.21542 | -0.08653 | 0.990724 | 0.846982 |
| F7 | -4.67502 | 7.88E-06 | 2.4E-05 | -0.16974 | -0.06873 | 0.991957 | 0.853539 |
| F8 | -5.21864 | 7.82E-07 | 5.47E-06 | -0.18697 | -0.08411 | 0.996084 | 0.95279 |
| T3 | -3.39134 | 0.000947 | 0.00117 | -0.10779 | -0.02831 | 0.867406 | 0.619171 |
| T4 | -4.71252 | 6.75E-06 | 2.4E-05 | -0.13635 | -0.05566 | 0.986527 | 0.860384 |
| T5 | -3.19326 | 0.001804 | 0.001994 | -0.15444 | -0.03621 | 0.883986 | 0.583008 |
| T6 | -3.456 | 0.000763 | 0.001001 | -0.16633 | -0.04515 | 0.930244 | 0.630976 |
| Fz | -3.71877 | 0.000308 | 0.000483 | -0.15289 | -0.04664 | 0.931294 | 0.678952 |
| Cz | -3.21681 | 0.001673 | 0.001952 | -0.13181 | -0.03136 | 0.844099 | 0.587306 |
| Pz | -3.98283 | 0.000118 | 0.000207 | -0.16317 | -0.05479 | 0.951492 | 0.727161 |
| F9 | -5.78551 | 6.06E-08 | 1.27E-06 | -0.1422 | -0.06968 | 0.999599 | 1.056285 |
| F10 | -5.49943 | 2.24E-07 | 2.35E-06 | -0.14015 | -0.06594 | 0.999272 | 1.004054 |

Table S2d. Relative band power: Beta Band

| channel | tvalue | pvalue | fdr_pvalue | CI_low | CI_high | statistical power | effect size |
| --- | --- | --- | --- | --- | --- | --- | --- |
| Fp1 | -2.73992 | 0.0071 | 0.009319 | -0.08944 | -0.01439 | 0.692287 | 0.500239 |
| Fp2 | -2.66445 | 0.00879 | 0.010256 | -0.08244 | -0.01214 | 0.654354 | 0.486459 |
| F3 | -2.07936 | 0.039751 | 0.039751 | -0.08029 | -0.00196 | 0.483912 | 0.379637 |
| F4 | -2.29827 | 0.023307 | 0.024472 | -0.08364 | -0.00622 | 0.560234 | 0.419606 |
| C3 | -4.03792 | 9.63E-05 | 0.000404 | -0.12313 | -0.0421 | 0.964529 | 0.73722 |
| C4 | -3.63805 | 0.000409 | 0.001226 | -0.11871 | -0.03503 | 0.926047 | 0.664215 |
| P3 | -2.98164 | 0.003483 | 0.006096 | -0.10439 | -0.02107 | 0.792621 | 0.54437 |
| P4 | -3.20232 | 0.001753 | 0.00368 | -0.10671 | -0.02516 | 0.836089 | 0.58466 |
| O1 | -2.95046 | 0.003828 | 0.006183 | -0.09396 | -0.01849 | 0.713512 | 0.538679 |
| O2 | -2.68788 | 0.00823 | 0.010167 | -0.09023 | -0.01368 | 0.62726 | 0.490738 |
| F7 | -2.62681 | 0.009763 | 0.01079 | -0.07632 | -0.01071 | 0.732766 | 0.479588 |
| F8 | -3.21932 | 0.00166 | 0.00368 | -0.08705 | -0.02074 | 0.839703 | 0.587764 |
| T3 | -4.35757 | 2.82E-05 | 0.000148 | -0.1036 | -0.03886 | 0.992993 | 0.795579 |
| T4 | -4.50463 | 1.57E-05 | 0.00011 | -0.10013 | -0.03898 | 0.986833 | 0.822429 |
| T5 | -3.80972 | 0.000222 | 0.000779 | -0.11983 | -0.03786 | 0.924629 | 0.695557 |
| T6 | -3.50566 | 0.000645 | 0.001693 | -0.11727 | -0.03261 | 0.854881 | 0.640042 |
| Fz | -2.78386 | 0.006258 | 0.008761 | -0.09021 | -0.01522 | 0.688263 | 0.508261 |
| Cz | -3.12029 | 0.002272 | 0.004337 | -0.09133 | -0.02041 | 0.810647 | 0.569684 |
| Pz | -2.90863 | 0.004339 | 0.006508 | -0.08323 | -0.0158 | 0.787124 | 0.531041 |
| F9 | -4.52109 | 1.47E-05 | 0.00011 | -0.07675 | -0.02999 | 0.987583 | 0.825434 |
| F10 | -5.42377 | 3.15E-07 | 6.61E-06 | -0.07907 | -0.03678 | 0.999214 | 0.99024 |

Table S2e. Relative band power: Gamma Band

| channel | tvalue | pvalue | fdr_pvalue | CI_low | CI_high | statistical power | effect size |
| --- | --- | --- | --- | --- | --- | --- | --- |
| Fp1 | 1.133503 | 0.259301 | 0.865049 | -0.00624 | 0.022954 | 0.437865 | -0.20695 |
| Fp2 | 0.439747 | 0.660925 | 0.865049 | -0.00788 | 0.012383 | 0.090833 | -0.08029 |
| F3 | -0.14633 | 0.883914 | 0.883914 | -0.01632 | 0.014073 | 0.05237 | 0.026715 |
| F4 | 0.764874 | 0.445873 | 0.865049 | -0.00799 | 0.018051 | 0.19422 | -0.13965 |
| C3 | -0.29043 | 0.771994 | 0.865049 | -0.01464 | 0.010896 | 0.06493 | 0.053026 |
| C4 | -1.14514 | 0.254467 | 0.865049 | -0.01612 | 0.004307 | 0.208164 | 0.209074 |
| P3 | -0.49145 | 0.624025 | 0.865049 | -0.00784 | 0.004725 | 0.077605 | 0.089725 |
| P4 | -0.90252 | 0.368617 | 0.865049 | -0.00969 | 0.003624 | 0.155839 | 0.164778 |
| O1 | -0.48044 | 0.631808 | 0.865049 | -0.00785 | 0.004784 | 0.077354 | 0.087715 |
| O2 | -0.54811 | 0.584654 | 0.865049 | -0.008 | 0.004533 | 0.08415 | 0.10007 |
| F7 | 0.966396 | 0.335823 | 0.865049 | -0.00776 | 0.022544 | 0.315857 | -0.17644 |
| F8 | 0.974444 | 0.331829 | 0.865049 | -0.00643 | 0.018901 | 0.26641 | -0.17791 |
| T3 | -0.74105 | 0.460134 | 0.865049 | -0.02327 | 0.010595 | 0.133066 | 0.135297 |
| T4 | 0.618478 | 0.537453 | 0.865049 | -0.01072 | 0.02046 | 0.1212 | -0.11292 |
| T5 | -0.21177 | 0.832649 | 0.874281 | -0.00897 | 0.007236 | 0.05622 | 0.038664 |
| T6 | -0.82256 | 0.412417 | 0.865049 | -0.01355 | 0.005595 | 0.152388 | 0.150178 |
| Fz | -0.78371 | 0.434779 | 0.865049 | -0.01684 | 0.00729 | 0.122591 | 0.143086 |
| Cz | -1.63801 | 0.104083 | 0.865049 | -0.0175 | 0.001655 | 0.308007 | 0.299058 |
| Pz | -0.78774 | 0.432431 | 0.865049 | -0.00898 | 0.003867 | 0.121122 | 0.14382 |
| F9 | 0.27648 | 0.782663 | 0.865049 | -0.00726 | 0.009616 | 0.063631 | -0.05048 |
| F10 | -0.32327 | 0.747059 | 0.865049 | -0.01181 | 0.008493 | 0.064104 | 0.059022 |

Table S3. Functional connectivity (coherence):

Table S3a: alpha_coh_HC_PT

| channel | tvalue | pvalue | fdr_pvalue | CI_low | CI_high | statistical power | effect size |
| --- | --- | --- | --- | --- | --- | --- | --- |
| Fp1-Fp2 | 2.167209 | 0.016114 | 0.047661 | 0.0254 | 65535 | 0.524034 | 0.395676 |
| Fp1-F3 | 0.079918 | 0.468219 | 0.574952 | -0.04892 | 65535 | 0.050726 | 0.014591 |
| Fp1-F4 | 3.775927 | 0.000126 | 0.001937 | 0.05609 | 65535 | 0.922362 | 0.689387 |
| Fp1-C3 | 0.014478 | 0.494237 | 0.599941 | -0.03704 | 65535 | 0.05004 | 0.002643 |
| Fp1-C4 | 4.402903 | 1.18E-05 | 0.001079 | 0.060291 | 65535 | 0.928639 | 0.803857 |
| Fp1-P3 | 3.303766 | 0.000632 | 0.005098 | 0.033595 | 65535 | 0.80639 | 0.603183 |
| Fp1-P4 | 2.770972 | 0.003248 | 0.014826 | 0.021766 | 65535 | 0.772218 | 0.505908 |
| Fp1-O1 | 0.005397 | 0.497851 | 0.600855 | -0.03664 | 65535 | 0.050003 | 0.000985 |
| Fp1-O2 | 0.840539 | 0.201153 | 0.306102 | -0.01765 | 65535 | 0.183607 | 0.153461 |
| Fp1-F7 | 1.750554 | 0.041311 | 0.095742 | 0.004152 | 65535 | 0.41423 | 0.319607 |
| Fp1-F8 | 3.614349 | 0.000222 | 0.002742 | 0.077195 | 65535 | 0.881701 | 0.659887 |
| Fp1-T3 | 0.493436 | 0.311311 | 0.432484 | -0.05076 | 65535 | 0.075803 | 0.090088 |
| Fp1-T4 | 2.748348 | 0.003466 | 0.015485 | 0.024473 | 65535 | 0.613763 | 0.501778 |
| Fp1-T5 | 1.083173 | 0.14047 | 0.230459 | -0.01704 | 65535 | 0.198404 | 0.197759 |
| Fp1-T6 | 0.494096 | 0.311078 | 0.432484 | -0.02319 | 65535 | 0.084194 | 0.090209 |
| Fp1-Fz | 1.361739 | 0.087937 | 0.166368 | -0.00629 | 65535 | 0.255909 | 0.248619 |
| Fp1-Cz | 1.40966 | 0.080635 | 0.158256 | -0.0088 | 65535 | 0.284742 | 0.257368 |
| Fp1-Pz | 2.966661 | 0.001823 | 0.010344 | 0.027701 | 65535 | 0.726286 | 0.541635 |
| Fp1-F9 | 1.602365 | 0.055874 | 0.118732 | -0.00139 | 65535 | 0.321913 | 0.29255 |
| Fp1-F10 | 1.576388 | 0.058807 | 0.123494 | -0.00259 | 65535 | 0.289815 | 0.287808 |
| Fp2-F3 | 2.125299 | 0.017824 | 0.049249 | 0.011926 | 65535 | 0.446149 | 0.388025 |
| Fp2-F4 | 0.737568 | 0.23112 | 0.334726 | -0.03098 | 65535 | 0.111474 | 0.134661 |
| Fp2-C3 | -0.5575 | 0.71088 | 0.777525 | -0.0535 | 65535 | 0.105418 | -0.10179 |
| Fp2-C4 | 2.803082 | 0.00296 | 0.013811 | 0.025488 | 65535 | 0.675034 | 0.51177 |
| Fp2-P3 | 3.811456 | 0.000111 | 0.001937 | 0.045451 | 65535 | 0.920503 | 0.695874 |
| Fp2-P4 | 2.994777 | 0.001674 | 0.010041 | 0.026492 | 65535 | 0.730284 | 0.546769 |
| Fp2-O1 | -0.25892 | 0.601927 | 0.686982 | -0.04599 | 65535 | 0.058691 | -0.04727 |
| Fp2-O2 | -0.45924 | 0.676546 | 0.751717 | -0.05608 | 65535 | 0.088431 | -0.08384 |
| Fp2-F7 | 1.281435 | 0.101277 | 0.181481 | -0.01541 | 65535 | 0.233466 | 0.233956 |
| Fp2-F8 | 0.324435 | 0.373092 | 0.486641 | -0.05591 | 65535 | 0.060302 | 0.059233 |
| Fp2-T3 | 1.223919 | 0.11171 | 0.192288 | -0.0145 | 65535 | 0.237036 | 0.223456 |
| Fp2-T4 | 0.964186 | 0.168462 | 0.270054 | -0.02183 | 65535 | 0.149689 | 0.176036 |
| Fp2-T5 | 1.14107 | 0.128076 | 0.213459 | -0.01084 | 65535 | 0.17831 | 0.20833 |
| Fp2-T6 | 0.667438 | 0.252898 | 0.363757 | -0.0229 | 65535 | 0.106776 | 0.121857 |
| Fp2-Fz | 0.256669 | 0.398941 | 0.510839 | -0.03724 | 65535 | 0.057145 | 0.046861 |
| Fp2-Cz | 0.883651 | 0.189341 | 0.29453 | -0.02674 | 65535 | 0.142183 | 0.161332 |
| Fp2-Pz | 2.917777 | 0.002111 | 0.010813 | 0.029312 | 65535 | 0.73819 | 0.532711 |
| Fp2-F9 | 1.601466 | 0.055974 | 0.118732 | -0.00149 | 65535 | 0.268675 | 0.292387 |
| Fp2-F10 | 2.900494 | 0.002223 | 0.011113 | 0.027445 | 65535 | 0.665227 | 0.529555 |
| F3-F4 | 4.111762 | 3.65E-05 | 0.001677 | 0.054071 | 65535 | 0.855895 | 0.750702 |
| F3-C3 | 0.864372 | 0.194569 | 0.298244 | -0.01876 | 65535 | 0.146739 | 0.157812 |
| F3-C4 | 3.49602 | 0.000333 | 0.003343 | 0.021646 | 65535 | 0.774595 | 0.638283 |
| F3-P3 | 2.930005 | 0.002035 | 0.010813 | 0.016833 | 65535 | 0.644818 | 0.534943 |
| F3-P4 | 3.045907 | 0.001431 | 0.008839 | 0.018564 | 65535 | 0.686575 | 0.556104 |
| F3-O1 | 1.425372 | 0.078345 | 0.155374 | -0.00257 | 65535 | 0.270721 | 0.260236 |
| F3-O2 | 2.4688 | 0.007494 | 0.028104 | 0.007346 | 65535 | 0.673617 | 0.450739 |
| F3-F7 | 0.259403 | 0.397888 | 0.510839 | -0.0485 | 65535 | 0.059472 | 0.04736 |
| F3-F8 | 3.955302 | 6.54E-05 | 0.001937 | 0.057544 | 65535 | 0.940134 | 0.722136 |
| F3-T3 | -1.27154 | 0.896981 | 0.927545 | -0.06775 | 65535 | 0.286221 | -0.23215 |
| F3-T4 | 0.626137 | 0.266217 | 0.38031 | -0.01491 | 65535 | 0.08413 | 0.114316 |
| F3-T5 | 0.807564 | 0.210483 | 0.313486 | -0.01387 | 65535 | 0.114473 | 0.14744 |
| F3-T6 | -0.17229 | 0.568248 | 0.660042 | -0.01743 | 65535 | 0.053361 | -0.03146 |
| F3-Fz | 3.292421 | 0.000655 | 0.005098 | 0.033382 | 65535 | 0.826408 | 0.601111 |
| F3-Cz | 1.256283 | 0.105747 | 0.186613 | -0.00848 | 65535 | 0.293548 | 0.229365 |
| F3-Pz | 2.336262 | 0.010582 | 0.035844 | 0.008606 | 65535 | 0.576987 | 0.426542 |
| F3-F9 | -0.16419 | 0.565069 | 0.660042 | -0.03694 | 65535 | 0.05251 | -0.02998 |
| F3-F10 | -2.08787 | 0.980519 | 0.980519 | -0.08368 | 65535 | 0.648939 | -0.38119 |
| F4-C3 | 0.809849 | 0.209829 | 0.313486 | -0.01003 | 65535 | 0.118677 | 0.147858 |
| F4-C4 | 2.108144 | 0.018567 | 0.049558 | 0.009297 | 65535 | 0.456405 | 0.384893 |
| F4-P3 | 3.545052 | 0.000282 | 0.003115 | 0.022531 | 65535 | 0.853174 | 0.647235 |
| F4-P4 | 2.971977 | 0.001793 | 0.010344 | 0.016418 | 65535 | 0.759954 | 0.542606 |
| F4-O1 | 2.343659 | 0.010384 | 0.035749 | 0.009156 | 65535 | 0.574305 | 0.427892 |
| F4-O2 | 1.91603 | 0.02889 | 0.072247 | 0.003358 | 65535 | 0.499344 | 0.349818 |
| F4-F7 | 3.16115 | 0.000999 | 0.006991 | 0.035093 | 65535 | 0.865288 | 0.577145 |
| F4-F8 | 2.12733 | 0.017737 | 0.049249 | 0.014804 | 65535 | 0.551982 | 0.388395 |
| F4-T3 | 1.299769 | 0.098107 | 0.178082 | -0.00634 | 65535 | 0.22626 | 0.237304 |
| F4-T4 | 0.452235 | 0.325965 | 0.438799 | -0.02077 | 65535 | 0.077509 | 0.082566 |
| F4-T5 | 2.005967 | 0.023574 | 0.061117 | 0.003896 | 65535 | 0.480767 | 0.366237 |
| F4-T6 | 1.277452 | 0.101975 | 0.181481 | -0.00463 | 65535 | 0.276461 | 0.233229 |
| F4-Fz | 2.642366 | 0.004675 | 0.019699 | 0.026364 | 65535 | 0.633349 | 0.482428 |
| F4-Cz | 3.202744 | 0.000875 | 0.006563 | 0.039901 | 65535 | 0.733256 | 0.584739 |
| F4-Pz | 2.612965 | 0.005072 | 0.020767 | 0.014381 | 65535 | 0.672123 | 0.47706 |
| F4-F9 | 0.117108 | 0.453487 | 0.563504 | -0.02644 | 65535 | 0.051269 | 0.021381 |
| F4-F10 | 1.38226 | 0.084751 | 0.163282 | -0.00368 | 65535 | 0.261209 | 0.252365 |
| C3-C4 | 1.546404 | 0.062342 | 0.129623 | -0.00168 | 65535 | 0.241741 | 0.282333 |
| C3-P3 | 0.259632 | 0.3978 | 0.510839 | -0.0367 | 65535 | 0.056714 | 0.047402 |
| C3-P4 | -0.33146 | 0.629556 | 0.710789 | -0.02238 | 65535 | 0.068468 | -0.06052 |
| C3-O1 | -1.12283 | 0.868106 | 0.906977 | -0.05375 | 65535 | 0.335741 | -0.205 |
| C3-O2 | -0.54343 | 0.706071 | 0.776308 | -0.02693 | 65535 | 0.119649 | -0.09922 |
| C3-F7 | 0.517515 | 0.302883 | 0.426882 | -0.02108 | 65535 | 0.07934 | 0.094485 |
| C3-F8 | 0.328191 | 0.371674 | 0.486641 | -0.02587 | 65535 | 0.062665 | 0.059919 |
| C3-T3 | -0.41905 | 0.662031 | 0.739503 | -0.04168 | 65535 | 0.077313 | -0.07651 |
| C3-T4 | 0.983615 | 0.163659 | 0.264372 | -0.01006 | 65535 | 0.140404 | 0.179583 |
| C3-T5 | 2.157993 | 0.016477 | 0.048059 | 0.006549 | 65535 | 0.513659 | 0.393994 |
| C3-T6 | -0.73907 | 0.769335 | 0.828515 | -0.03024 | 65535 | 0.138856 | -0.13494 |
| C3-Fz | 0.873806 | 0.191999 | 0.29647 | -0.01075 | 65535 | 0.13882 | 0.159534 |
| C3-Cz | -2.08144 | 0.980222 | 0.980519 | -0.10399 | 65535 | 0.810747 | -0.38002 |
| C3-Pz | -0.2557 | 0.600686 | 0.686982 | -0.02191 | 65535 | 0.056738 | -0.04668 |
| C3-F9 | 2.136226 | 0.017363 | 0.049249 | 0.008453 | 65535 | 0.401475 | 0.39002 |
| C3-F10 | -0.23216 | 0.591591 | 0.682605 | -0.02454 | 65535 | 0.057811 | -0.04239 |
| C4-P3 | 3.475736 | 0.000357 | 0.003406 | 0.025379 | 65535 | 0.769022 | 0.63458 |
| C4-P4 | 2.106425 | 0.018643 | 0.049558 | 0.012076 | 65535 | 0.461621 | 0.384579 |
| C4-O1 | 2.406884 | 0.00882 | 0.031934 | 0.007289 | 65535 | 0.571113 | 0.439435 |
| C4-O2 | 2.607971 | 0.005142 | 0.020767 | 0.013054 | 65535 | 0.675731 | 0.476148 |
| C4-F7 | 4.351131 | 1.45E-05 | 0.001079 | 0.047253 | 65535 | 0.931856 | 0.794404 |
| C4-F8 | 2.397295 | 0.009043 | 0.032187 | 0.014365 | 65535 | 0.544295 | 0.437684 |
| C4-T3 | 3.344889 | 0.000552 | 0.004637 | 0.025866 | 65535 | 0.787349 | 0.610691 |
| C4-T4 | -0.05744 | 0.522854 | 0.623859 | -0.02899 | 65535 | 0.050423 | -0.01049 |
| C4-T5 | 1.861699 | 0.032567 | 0.080459 | 0.002283 | 65535 | 0.495024 | 0.339898 |
| C4-T6 | 3.807259 | 0.000112 | 0.001937 | 0.034458 | 65535 | 0.870967 | 0.695108 |
| C4-Fz | 1.711136 | 0.044842 | 0.100691 | 0.000895 | 65535 | 0.314767 | 0.312409 |
| C4-Cz | 2.281794 | 0.012147 | 0.039244 | 0.018191 | 65535 | 0.429459 | 0.416597 |
| C4-Pz | 1.375633 | 0.08577 | 0.163744 | -0.00569 | 65535 | 0.230776 | 0.251155 |
| C4-F9 | 1.233824 | 0.10986 | 0.190666 | -0.00622 | 65535 | 0.220765 | 0.225265 |
| C4-F10 | 4.335236 | 1.54E-05 | 0.001079 | 0.0387 | 65535 | 0.921774 | 0.791502 |
| P3-P4 | 3.74882 | 0.000138 | 0.001937 | 0.037985 | 65535 | 0.884332 | 0.684438 |
| P3-O1 | 0.958486 | 0.169889 | 0.270278 | -0.01777 | 65535 | 0.15162 | 0.174995 |
| P3-O2 | 1.234199 | 0.109791 | 0.190666 | -0.00543 | 65535 | 0.284893 | 0.225333 |
| P3-F7 | 2.950282 | 0.001915 | 0.010582 | 0.023448 | 65535 | 0.828173 | 0.538645 |
| P3-F8 | 3.587542 | 0.000244 | 0.002841 | 0.03299 | 65535 | 0.873084 | 0.654992 |
| P3-T3 | 0.786462 | 0.216587 | 0.318064 | -0.01827 | 65535 | 0.11377 | 0.143588 |
| P3-T4 | 2.706432 | 0.003905 | 0.017084 | 0.016061 | 65535 | 0.626524 | 0.494125 |
| P3-T5 | 2.220745 | 0.014139 | 0.042416 | 0.012261 | 65535 | 0.556646 | 0.405451 |
| P3-T6 | 1.165128 | 0.123159 | 0.208575 | -0.00674 | 65535 | 0.193427 | 0.212722 |
| P3-Fz | 2.541464 | 0.006168 | 0.023986 | 0.010428 | 65535 | 0.665429 | 0.464006 |
| P3-Cz | 1.708664 | 0.045071 | 0.100691 | 0.001026 | 65535 | 0.351136 | 0.311958 |
| P3-Pz | 2.553395 | 0.005971 | 0.02366 | 0.020924 | 65535 | 0.624015 | 0.466184 |
| P3-F9 | 2.918285 | 0.002108 | 0.010813 | 0.020728 | 65535 | 0.704553 | 0.532803 |
| P3-F10 | 3.176188 | 0.000952 | 0.006896 | 0.014487 | 65535 | 0.825865 | 0.579891 |
| P4-O1 | 1.748509 | 0.041488 | 0.095742 | 0.00127 | 65535 | 0.400796 | 0.319232 |
| P4-O2 | 2.641177 | 0.00469 | 0.019699 | 0.017476 | 65535 | 0.711277 | 0.482211 |
| P4-F7 | 2.421737 | 0.008484 | 0.031258 | 0.012847 | 65535 | 0.587144 | 0.442146 |
| P4-F8 | 3.106546 | 0.001186 | 0.007545 | 0.026624 | 65535 | 0.830059 | 0.567175 |
| P4-T3 | 1.498357 | 0.068356 | 0.139366 | -0.00353 | 65535 | 0.290696 | 0.273561 |
| P4-T4 | 2.289984 | 0.011899 | 0.039045 | 0.010834 | 65535 | 0.45497 | 0.418092 |
| P4-T5 | 2.329032 | 0.010779 | 0.035931 | 0.010887 | 65535 | 0.551081 | 0.425221 |
| P4-T6 | 1.676804 | 0.048114 | 0.104165 | 0.000444 | 65535 | 0.39806 | 0.306141 |
| P4-Fz | 2.484458 | 0.007189 | 0.027448 | 0.010582 | 65535 | 0.640238 | 0.453598 |
| P4-Cz | 0.936485 | 0.175468 | 0.277055 | -0.0143 | 65535 | 0.138764 | 0.170978 |
| P4-Pz | 2.365563 | 0.009817 | 0.034358 | 0.017348 | 65535 | 0.615274 | 0.431891 |
| P4-F9 | 3.442499 | 0.000399 | 0.003644 | 0.02046 | 65535 | 0.761957 | 0.628512 |
| P4-F10 | 3.909057 | 7.76E-05 | 0.001937 | 0.034894 | 65535 | 0.877967 | 0.713693 |
| O1-O2 | 0.223902 | 0.41161 | 0.520712 | -0.04507 | 65535 | 0.054895 | 0.040879 |
| O1-F7 | -1.80949 | 0.96354 | 0.980519 | -0.06599 | 65535 | 0.599658 | -0.33037 |
| O1-F8 | -0.0419 | 0.516675 | 0.62001 | -0.02716 | 65535 | 0.050274 | -0.00765 |
| O1-T3 | -1.41224 | 0.919744 | 0.942177 | -0.07727 | 65535 | 0.585606 | -0.25784 |
| O1-T4 | 1.742139 | 0.042045 | 0.095972 | 0.001341 | 65535 | 0.364906 | 0.318069 |
| O1-T5 | 2.261755 | 0.012772 | 0.040004 | 0.011187 | 65535 | 0.597738 | 0.412938 |
| O1-T6 | 0.325943 | 0.372522 | 0.486641 | -0.01922 | 65535 | 0.065168 | 0.059509 |
| O1-Fz | 1.298236 | 0.098369 | 0.178082 | -0.00352 | 65535 | 0.217196 | 0.237024 |
| O1-Cz | -2.05366 | 0.978891 | 0.980519 | -0.07466 | 65535 | 0.777827 | -0.37495 |
| O1-Pz | 1.319946 | 0.094704 | 0.174455 | -0.00618 | 65535 | 0.230286 | 0.240988 |
| O1-F9 | 3.149456 | 0.001036 | 0.007003 | 0.026055 | 65535 | 0.840969 | 0.575009 |
| O1-F10 | 1.149191 | 0.126401 | 0.212353 | -0.00596 | 65535 | 0.198515 | 0.209813 |
| O2-F7 | 0.468889 | 0.320007 | 0.436373 | -0.02055 | 65535 | 0.086054 | 0.085607 |
| O2-F8 | 0.214402 | 0.415302 | 0.522236 | -0.02747 | 65535 | 0.056762 | 0.039144 |
| O2-T3 | -0.11302 | 0.544899 | 0.642858 | -0.02899 | 65535 | 0.052033 | -0.02064 |
| O2-T4 | 3.772889 | 0.000127 | 0.001937 | 0.031676 | 65535 | 0.895261 | 0.688832 |
| O2-T5 | 0.810826 | 0.209549 | 0.313486 | -0.01499 | 65535 | 0.119118 | 0.148036 |
| O2-T6 | 3.939366 | 6.94E-05 | 0.001937 | 0.031219 | 65535 | 0.930663 | 0.719226 |
| O2-Fz | 1.797613 | 0.037398 | 0.089415 | 0.00142 | 65535 | 0.515763 | 0.328198 |
| O2-Cz | -1.14587 | 0.872915 | 0.907486 | -0.06385 | 65535 | 0.587189 | -0.20921 |
| O2-Pz | 1.510885 | 0.066746 | 0.137417 | -0.00304 | 65535 | 0.258846 | 0.275849 |
| O2-F9 | 1.180609 | 0.120066 | 0.204991 | -0.00502 | 65535 | 0.247541 | 0.215548 |
| O2-F10 | 3.821151 | 0.000107 | 0.001937 | 0.041475 | 65535 | 0.846592 | 0.697643 |
| F7-F8 | 2.8766 | 0.002386 | 0.011543 | 0.054745 | 65535 | 0.755569 | 0.525193 |
| F7-T3 | -0.30607 | 0.619955 | 0.703733 | -0.08136 | 65535 | 0.063188 | -0.05588 |
| F7-T4 | 0.407772 | 0.34209 | 0.457573 | -0.03572 | 65535 | 0.070453 | 0.074449 |
| F7-T5 | -1.29481 | 0.901044 | 0.927545 | -0.0779 | 65535 | 0.300983 | -0.2364 |
| F7-T6 | -0.34151 | 0.633336 | 0.711233 | -0.04412 | 65535 | 0.072124 | -0.06235 |
| F7-Fz | 1.440029 | 0.076253 | 0.153973 | -0.00283 | 65535 | 0.33031 | 0.262913 |
| F7-Cz | 1.79672 | 0.037469 | 0.089415 | 0.003791 | 65535 | 0.386485 | 0.328034 |
| F7-Pz | 3.140159 | 0.001067 | 0.007003 | 0.030964 | 65535 | 0.734404 | 0.573312 |
| F7-F9 | -0.17394 | 0.568893 | 0.660042 | -0.06185 | 65535 | 0.05376 | -0.03176 |
| F7-F10 | 1.339689 | 0.09146 | 0.169971 | -0.01021 | 65535 | 0.261429 | 0.244592 |
| F8-T3 | 1.915895 | 0.028899 | 0.072247 | 0.007672 | 65535 | 0.402244 | 0.349793 |
| F8-T4 | -0.82231 | 0.793721 | 0.844142 | -0.08458 | 65535 | 0.123739 | -0.15013 |
| F8-T5 | 1.424803 | 0.078427 | 0.155374 | -0.00451 | 65535 | 0.226002 | 0.260132 |
| F8-T6 | 0.482717 | 0.315096 | 0.432484 | -0.02565 | 65535 | 0.083799 | 0.088131 |
| F8-Fz | 2.128106 | 0.017704 | 0.049249 | 0.007784 | 65535 | 0.510442 | 0.388537 |
| F8-Cz | 2.250216 | 0.013144 | 0.040004 | 0.016114 | 65535 | 0.523505 | 0.410831 |
| F8-Pz | 3.428022 | 0.000419 | 0.003666 | 0.037895 | 65535 | 0.849483 | 0.625868 |
| F8-F9 | -0.77535 | 0.780159 | 0.835884 | -0.07523 | 65535 | 0.119172 | -0.14156 |
| F8-F10 | 3.66024 | 0.000189 | 0.002482 | 0.053666 | 65535 | 0.846007 | 0.668265 |
| T3-T4 | 2.872017 | 0.002418 | 0.011543 | 0.024609 | 65535 | 0.693865 | 0.524356 |
| T3-T5 | 0.485567 | 0.314087 | 0.432484 | -0.03234 | 65535 | 0.076122 | 0.088652 |
| T3-T6 | 0.929039 | 0.177383 | 0.277988 | -0.01394 | 65535 | 0.149393 | 0.169618 |
| T3-Fz | -0.08512 | 0.533843 | 0.633373 | -0.0224 | 65535 | 0.050899 | -0.01554 |
| T3-Cz | 0.084794 | 0.466284 | 0.574952 | -0.04501 | 65535 | 0.051107 | 0.015481 |
| T3-Pz | 2.097981 | 0.01902 | 0.049928 | 0.007785 | 65535 | 0.516624 | 0.383037 |
| T3-F9 | 0.458913 | 0.32357 | 0.438386 | -0.03748 | 65535 | 0.075897 | 0.083786 |
| T3-F10 | 1.761422 | 0.040379 | 0.095275 | 0.003596 | 65535 | 0.515325 | 0.32159 |
| T4-T5 | -0.63931 | 0.738071 | 0.803082 | -0.04177 | 65535 | 0.102382 | -0.11672 |
| T4-T6 | 1.684224 | 0.047391 | 0.103668 | 0.000717 | 65535 | 0.355215 | 0.307496 |
| T4-Fz | 1.836157 | 0.034426 | 0.084063 | 0.001786 | 65535 | 0.375393 | 0.335235 |
| T4-Cz | 0.992011 | 0.161611 | 0.263088 | -0.00923 | 65535 | 0.166546 | 0.181115 |
| T4-Pz | 2.253774 | 0.013028 | 0.040004 | 0.009124 | 65535 | 0.535772 | 0.411481 |
| T4-F9 | -1.04378 | 0.850639 | 0.893886 | -0.07785 | 65535 | 0.15289 | -0.19057 |
| T4-F10 | 0.24063 | 0.40513 | 0.51562 | -0.03627 | 65535 | 0.056231 | 0.043932 |
| T5-T6 | -0.65583 | 0.743395 | 0.804706 | -0.03017 | 65535 | 0.109289 | -0.11974 |
| T5-Fz | 1.932599 | 0.027841 | 0.0713 | 0.002811 | 65535 | 0.450243 | 0.352843 |
| T5-Cz | 1.691453 | 0.046695 | 0.10322 | 0.000486 | 65535 | 0.357796 | 0.308815 |
| T5-Pz | 0.789424 | 0.215724 | 0.318064 | -0.01555 | 65535 | 0.163889 | 0.144128 |
| T5-F9 | 0.07313 | 0.470913 | 0.574952 | -0.04431 | 65535 | 0.050698 | 0.013351 |
| T5-F10 | -1.04674 | 0.85132 | 0.893886 | -0.07217 | 65535 | 0.17385 | -0.19111 |
| T6-Fz | 0.553166 | 0.290598 | 0.412335 | -0.00913 | 65535 | 0.091889 | 0.100994 |
| T6-Cz | 0.141113 | 0.444011 | 0.555013 | -0.0287 | 65535 | 0.05288 | 0.025763 |
| T6-Pz | 0.331641 | 0.370374 | 0.486641 | -0.02343 | 65535 | 0.06871 | 0.060549 |
| T6-F9 | -0.47097 | 0.680735 | 0.752391 | -0.05305 | 65535 | 0.074239 | -0.08599 |
| T6-F10 | 1.099062 | 0.136989 | 0.226518 | -0.01425 | 65535 | 0.197073 | 0.200661 |
| Fz-Cz | -0.83005 | 0.795905 | 0.844142 | -0.05343 | 65535 | 0.163141 | -0.15155 |
| Fz-Pz | 0.748362 | 0.227866 | 0.332304 | -0.01032 | 65535 | 0.123522 | 0.136631 |
| Fz-F9 | 1.348528 | 0.090036 | 0.168817 | -0.00327 | 65535 | 0.237711 | 0.246206 |
| Fz-F10 | 2.264882 | 0.012672 | 0.040004 | 0.005431 | 65535 | 0.544345 | 0.413509 |
| Cz-Pz | 2.116489 | 0.018202 | 0.049558 | 0.013633 | 65535 | 0.443261 | 0.386416 |
| Cz-F9 | 1.382393 | 0.084731 | 0.163282 | -0.00367 | 65535 | 0.273315 | 0.252389 |
| Cz-F10 | 3.765595 | 0.00013 | 0.001937 | 0.026103 | 65535 | 0.858996 | 0.6875 |
| Pz-F9 | 3.495036 | 0.000334 | 0.003343 | 0.023207 | 65535 | 0.811014 | 0.638103 |
| Pz-F10 | 4.087599 | 3.99E-05 | 0.001677 | 0.029595 | 65535 | 0.904003 | 0.74629 |
| F9-F10 | -1.92889 | 0.971927 | 0.980519 | -0.10239 | 65535 | 0.457916 | -0.35217 |

Table S3b: alpha_coh_PT_HC

| channel | tvalue | pvalue | fdr_pvalue | CI_low | CI_high | statistical power | effect size |
| --- | --- | --- | --- | --- | --- | --- | --- |
| Fp1-Fp2 | 2.167209 | 0.983886 | 0.999988 | 65535 | 0.190755 | 0.524034 | 0.395676 |
| Fp1-F3 | 0.079918 | 0.531781 | 0.999988 | 65535 | 0.053873 | 0.050726 | 0.014591 |
| Fp1-F4 | 3.775927 | 0.999874 | 0.999988 | 65535 | 0.143897 | 0.922362 | 0.689387 |
| Fp1-C3 | 0.014478 | 0.505763 | 0.999988 | 65535 | 0.037694 | 0.05004 | 0.002643 |
| Fp1-C4 | 4.402903 | 0.999988 | 0.999988 | 65535 | 0.133116 | 0.928639 | 0.803857 |
| Fp1-P3 | 3.303766 | 0.999368 | 0.999988 | 65535 | 0.101272 | 0.80639 | 0.603183 |
| Fp1-P4 | 2.770972 | 0.996752 | 0.999988 | 65535 | 0.086602 | 0.772218 | 0.505908 |
| Fp1-O1 | 0.005397 | 0.502149 | 0.999988 | 65535 | 0.03688 | 0.050003 | 0.000985 |
| Fp1-O2 | 0.840539 | 0.798847 | 0.999988 | 65535 | 0.053946 | 0.183607 | 0.153461 |
| Fp1-F7 | 1.750554 | 0.958689 | 0.999988 | 65535 | 0.152669 | 0.41423 | 0.319607 |
| Fp1-F8 | 3.614349 | 0.999778 | 0.999988 | 65535 | 0.208022 | 0.881701 | 0.659887 |
| Fp1-T3 | 0.493436 | 0.688689 | 0.999988 | 65535 | 0.093787 | 0.075803 | 0.090088 |
| Fp1-T4 | 2.748348 | 0.996534 | 0.999988 | 65535 | 0.098886 | 0.613763 | 0.501778 |
| Fp1-T5 | 1.083173 | 0.85953 | 0.999988 | 65535 | 0.081252 | 0.198404 | 0.197759 |
| Fp1-T6 | 0.494096 | 0.688922 | 0.999988 | 65535 | 0.042877 | 0.084194 | 0.090209 |
| Fp1-Fz | 1.361739 | 0.912063 | 0.999988 | 65535 | 0.064178 | 0.255909 | 0.248619 |
| Fp1-Cz | 1.40966 | 0.919365 | 0.999988 | 65535 | 0.108724 | 0.284742 | 0.257368 |
| Fp1-Pz | 2.966661 | 0.998177 | 0.999988 | 65535 | 0.097881 | 0.726286 | 0.541635 |
| Fp1-F9 | 1.602365 | 0.944126 | 0.999988 | 65535 | 0.081733 | 0.321913 | 0.29255 |
| Fp1-F10 | 1.576388 | 0.941193 | 0.999988 | 65535 | 0.102674 | 0.289815 | 0.287808 |
| Fp2-F3 | 2.125299 | 0.982176 | 0.999988 | 65535 | 0.096524 | 0.446149 | 0.388025 |
| Fp2-F4 | 0.737568 | 0.76888 | 0.999988 | 65535 | 0.08064 | 0.111474 | 0.134661 |
| Fp2-C3 | -0.5575 | 0.28912 | 0.999988 | 65535 | 0.026574 | 0.105418 | -0.10179 |
| Fp2-C4 | 2.803082 | 0.99704 | 0.999988 | 65535 | 0.099282 | 0.675034 | 0.51177 |
| Fp2-P3 | 3.811456 | 0.999889 | 0.999988 | 65535 | 0.11543 | 0.920503 | 0.695874 |
| Fp2-P4 | 2.994777 | 0.998326 | 0.999988 | 65535 | 0.092198 | 0.730284 | 0.546769 |
| Fp2-O1 | -0.25892 | 0.398073 | 0.999988 | 65535 | 0.033569 | 0.058691 | -0.04727 |
| Fp2-O2 | -0.45924 | 0.323455 | 0.999988 | 65535 | 0.03175 | 0.088431 | -0.08384 |
| Fp2-F7 | 1.281435 | 0.898723 | 0.999988 | 65535 | 0.120348 | 0.233466 | 0.233956 |
| Fp2-F8 | 0.324435 | 0.626908 | 0.999988 | 65535 | 0.083115 | 0.060302 | 0.059233 |
| Fp2-T3 | 1.223919 | 0.88829 | 0.999988 | 65535 | 0.096302 | 0.237036 | 0.223456 |
| Fp2-T4 | 0.964186 | 0.831538 | 0.999988 | 65535 | 0.082507 | 0.149689 | 0.176036 |
| Fp2-T5 | 1.14107 | 0.871924 | 0.999988 | 65535 | 0.058726 | 0.17831 | 0.20833 |
| Fp2-T6 | 0.667438 | 0.747102 | 0.999988 | 65535 | 0.053756 | 0.106776 | 0.121857 |
| Fp2-Fz | 0.256669 | 0.601059 | 0.999988 | 65535 | 0.050879 | 0.057145 | 0.046861 |
| Fp2-Cz | 0.883651 | 0.810659 | 0.999988 | 65535 | 0.087794 | 0.142183 | 0.161332 |
| Fp2-Pz | 2.917777 | 0.997889 | 0.999988 | 65535 | 0.106454 | 0.73819 | 0.532711 |
| Fp2-F9 | 1.601466 | 0.944026 | 0.999988 | 65535 | 0.086386 | 0.268675 | 0.292387 |
| Fp2-F10 | 2.900494 | 0.997777 | 0.999988 | 65535 | 0.100678 | 0.665227 | 0.529555 |
| F3-F4 | 4.111762 | 0.999964 | 0.999988 | 65535 | 0.127132 | 0.855895 | 0.750702 |
| F3-C3 | 0.864372 | 0.805431 | 0.999988 | 65535 | 0.059625 | 0.146739 | 0.157812 |
| F3-C4 | 3.49602 | 0.999667 | 0.999988 | 65535 | 0.060691 | 0.774595 | 0.638283 |
| F3-P3 | 2.930005 | 0.997965 | 0.999988 | 65535 | 0.060708 | 0.644818 | 0.534943 |
| F3-P4 | 3.045907 | 0.998569 | 0.999988 | 65535 | 0.062908 | 0.686575 | 0.556104 |
| F3-O1 | 1.425372 | 0.921655 | 0.999988 | 65535 | 0.034033 | 0.270721 | 0.260236 |
| F3-O2 | 2.4688 | 0.992505 | 0.999988 | 65535 | 0.037383 | 0.673617 | 0.450739 |
| F3-F7 | 0.259403 | 0.602112 | 0.999988 | 65535 | 0.066496 | 0.059472 | 0.04736 |
| F3-F8 | 3.955302 | 0.999935 | 0.999988 | 65535 | 0.140595 | 0.940134 | 0.722136 |
| F3-T3 | -1.27154 | 0.103019 | 0.999988 | 65535 | 0.008935 | 0.286221 | -0.23215 |
| F3-T4 | 0.626137 | 0.733783 | 0.999988 | 65535 | 0.033016 | 0.08413 | 0.114316 |
| F3-T5 | 0.807564 | 0.789517 | 0.999988 | 65535 | 0.040212 | 0.114473 | 0.14744 |
| F3-T6 | -0.17229 | 0.431752 | 0.999988 | 65535 | 0.014148 | 0.053361 | -0.03146 |
| F3-Fz | 3.292421 | 0.999345 | 0.999988 | 65535 | 0.101099 | 0.826408 | 0.601111 |
| F3-Cz | 1.256283 | 0.894253 | 0.999988 | 65535 | 0.06154 | 0.293548 | 0.229365 |
| F3-Pz | 2.336262 | 0.989418 | 0.999988 | 65535 | 0.05067 | 0.576987 | 0.426542 |
| F3-F9 | -0.16419 | 0.434931 | 0.999988 | 65535 | 0.030286 | 0.05251 | -0.02998 |
| F3-F10 | -2.08787 | 0.019481 | 0.999988 | 65535 | -0.00961 | 0.648939 | -0.38119 |
| F4-C3 | 0.809849 | 0.790172 | 0.999988 | 65535 | 0.029201 | 0.118677 | 0.147858 |
| F4-C4 | 2.108144 | 0.981433 | 0.999988 | 65535 | 0.07776 | 0.456405 | 0.384893 |
| F4-P3 | 3.545052 | 0.999718 | 0.999988 | 65535 | 0.062117 | 0.853174 | 0.647235 |
| F4-P4 | 2.971977 | 0.998206 | 0.999988 | 65535 | 0.057845 | 0.759954 | 0.542606 |
| F4-O1 | 2.343659 | 0.989616 | 0.999988 | 65535 | 0.053426 | 0.574305 | 0.427892 |
| F4-O2 | 1.91603 | 0.97111 | 0.999988 | 65535 | 0.046486 | 0.499344 | 0.349818 |
| F4-F7 | 3.16115 | 0.999001 | 0.999988 | 65535 | 0.112497 | 0.865288 | 0.577145 |
| F4-F8 | 2.12733 | 0.982263 | 0.999988 | 65535 | 0.119367 | 0.551982 | 0.388395 |
| F4-T3 | 1.299769 | 0.901893 | 0.999988 | 65535 | 0.052405 | 0.22626 | 0.237304 |
| F4-T4 | 0.452235 | 0.674035 | 0.999988 | 65535 | 0.036356 | 0.077509 | 0.082566 |
| F4-T5 | 2.005967 | 0.976426 | 0.999988 | 65535 | 0.041002 | 0.480767 | 0.366237 |
| F4-T6 | 1.277452 | 0.898025 | 0.999988 | 65535 | 0.035752 | 0.276461 | 0.233229 |
| F4-Fz | 2.642366 | 0.995325 | 0.999988 | 65535 | 0.115158 | 0.633349 | 0.482428 |
| F4-Cz | 3.202744 | 0.999125 | 0.999988 | 65535 | 0.125541 | 0.733256 | 0.584739 |
| F4-Pz | 2.612965 | 0.994928 | 0.999988 | 65535 | 0.064306 | 0.672123 | 0.47706 |
| F4-F9 | 0.117108 | 0.546513 | 0.999988 | 65535 | 0.030462 | 0.051269 | 0.021381 |
| F4-F10 | 1.38226 | 0.915249 | 0.999988 | 65535 | 0.040625 | 0.261209 | 0.252365 |
| C3-C4 | 1.546404 | 0.937658 | 0.999988 | 65535 | 0.048373 | 0.241741 | 0.282333 |
| C3-P3 | 0.259632 | 0.6022 | 0.999988 | 65535 | 0.050335 | 0.056714 | 0.047402 |
| C3-P4 | -0.33146 | 0.370444 | 0.999988 | 65535 | 0.014925 | 0.068468 | -0.06052 |
| C3-O1 | -1.12283 | 0.131894 | 0.999988 | 65535 | 0.010342 | 0.335741 | -0.205 |
| C3-O2 | -0.54343 | 0.293929 | 0.999988 | 65535 | 0.013634 | 0.119649 | -0.09922 |
| C3-F7 | 0.517515 | 0.697117 | 0.999988 | 65535 | 0.040206 | 0.07934 | 0.094485 |
| C3-F8 | 0.328191 | 0.628326 | 0.999988 | 65535 | 0.038641 | 0.062665 | 0.059919 |
| C3-T3 | -0.41905 | 0.337969 | 0.999988 | 65535 | 0.02486 | 0.077313 | -0.07651 |
| C3-T4 | 0.983615 | 0.836341 | 0.999988 | 65535 | 0.03942 | 0.140404 | 0.179583 |
| C3-T5 | 2.157993 | 0.983523 | 0.999988 | 65535 | 0.049971 | 0.513659 | 0.393994 |
| C3-T6 | -0.73907 | 0.230665 | 0.999988 | 65535 | 0.011592 | 0.138856 | -0.13494 |
| C3-Fz | 0.873806 | 0.808001 | 0.999988 | 65535 | 0.034697 | 0.13882 | 0.159534 |
| C3-Cz | -2.08144 | 0.019778 | 0.999988 | 65535 | -0.01178 | 0.810747 | -0.38002 |
| C3-Pz | -0.2557 | 0.399314 | 0.999988 | 65535 | 0.016053 | 0.056738 | -0.04668 |
| C3-F9 | 2.136226 | 0.982637 | 0.999988 | 65535 | 0.067049 | 0.401475 | 0.39002 |
| C3-F10 | -0.23216 | 0.408409 | 0.999988 | 65535 | 0.018508 | 0.057811 | -0.04239 |
| C4-P3 | 3.475736 | 0.999643 | 0.999988 | 65535 | 0.071669 | 0.769022 | 0.63458 |
| C4-P4 | 2.106425 | 0.981357 | 0.999988 | 65535 | 0.101339 | 0.461621 | 0.384579 |
| C4-O1 | 2.406884 | 0.99118 | 0.999988 | 65535 | 0.039557 | 0.571113 | 0.439435 |
| C4-O2 | 2.607971 | 0.994858 | 0.999988 | 65535 | 0.058612 | 0.675731 | 0.476148 |
| C4-F7 | 4.351131 | 0.999986 | 0.999988 | 65535 | 0.105427 | 0.931856 | 0.794404 |
| C4-F8 | 2.397295 | 0.990957 | 0.999988 | 65535 | 0.078782 | 0.544295 | 0.437684 |
| C4-T3 | 3.344889 | 0.999448 | 0.999988 | 65535 | 0.076703 | 0.787349 | 0.610691 |
| C4-T4 | -0.05744 | 0.477146 | 0.999988 | 65535 | 0.027048 | 0.050423 | -0.01049 |
| C4-T5 | 1.861699 | 0.967433 | 0.999988 | 65535 | 0.039423 | 0.495024 | 0.339898 |
| C4-T6 | 3.807259 | 0.999888 | 0.999988 | 65535 | 0.087614 | 0.870967 | 0.695108 |
| C4-Fz | 1.711136 | 0.955158 | 0.999988 | 65535 | 0.056613 | 0.314767 | 0.312409 |
| C4-Cz | 2.281794 | 0.987853 | 0.999988 | 65535 | 0.114867 | 0.429459 | 0.416597 |
| C4-Pz | 1.375633 | 0.91423 | 0.999988 | 65535 | 0.06117 | 0.230776 | 0.251155 |
| C4-F9 | 1.233824 | 0.89014 | 0.999988 | 65535 | 0.042396 | 0.220765 | 0.225265 |
| C4-F10 | 4.335236 | 0.999985 | 0.999988 | 65535 | 0.086627 | 0.921774 | 0.791502 |
| P3-P4 | 3.74882 | 0.999862 | 0.999988 | 65535 | 0.09822 | 0.884332 | 0.684438 |
| P3-O1 | 0.958486 | 0.830111 | 0.999988 | 65535 | 0.066463 | 0.15162 | 0.174995 |
| P3-O2 | 1.234199 | 0.890209 | 0.999988 | 65535 | 0.037055 | 0.284893 | 0.225333 |
| P3-F7 | 2.950282 | 0.998085 | 0.999988 | 65535 | 0.083604 | 0.828173 | 0.538645 |
| P3-F8 | 3.587542 | 0.999756 | 0.999988 | 65535 | 0.089677 | 0.873084 | 0.654992 |
| P3-T3 | 0.786462 | 0.783413 | 0.999988 | 65535 | 0.05126 | 0.11377 | 0.143588 |
| P3-T4 | 2.706432 | 0.996095 | 0.999988 | 65535 | 0.06685 | 0.626524 | 0.494125 |
| P3-T5 | 2.220745 | 0.985861 | 0.999988 | 65535 | 0.084488 | 0.556646 | 0.405451 |
| P3-T6 | 1.165128 | 0.876841 | 0.999988 | 65535 | 0.038623 | 0.193427 | 0.212722 |
| P3-Fz | 2.541464 | 0.993832 | 0.999988 | 65535 | 0.049559 | 0.665429 | 0.464006 |
| P3-Cz | 1.708664 | 0.954929 | 0.999988 | 65535 | 0.068015 | 0.351136 | 0.311958 |
| P3-Pz | 2.553395 | 0.994029 | 0.999988 | 65535 | 0.098397 | 0.624015 | 0.466184 |
| P3-F9 | 2.918285 | 0.997892 | 0.999988 | 65535 | 0.075257 | 0.704553 | 0.532803 |
| P3-F10 | 3.176188 | 0.999048 | 0.999988 | 65535 | 0.046124 | 0.825865 | 0.579891 |
| P4-O1 | 1.748509 | 0.958512 | 0.999988 | 65535 | 0.047745 | 0.400796 | 0.319232 |
| P4-O2 | 2.641177 | 0.99531 | 0.999988 | 65535 | 0.076405 | 0.711277 | 0.482211 |
| P4-F7 | 2.421737 | 0.991516 | 0.999988 | 65535 | 0.068615 | 0.587144 | 0.442146 |
| P4-F8 | 3.106546 | 0.998814 | 0.999988 | 65535 | 0.087561 | 0.830059 | 0.567175 |
| P4-T3 | 1.498357 | 0.931644 | 0.999988 | 65535 | 0.069767 | 0.290696 | 0.273561 |
| P4-T4 | 2.289984 | 0.988101 | 0.999988 | 65535 | 0.067661 | 0.45497 | 0.418092 |
| P4-T5 | 2.329032 | 0.989221 | 0.999988 | 65535 | 0.064673 | 0.551081 | 0.425221 |
| P4-T6 | 1.676804 | 0.951886 | 0.999988 | 65535 | 0.078128 | 0.39806 | 0.306141 |
| P4-Fz | 2.484458 | 0.992811 | 0.999988 | 65535 | 0.053031 | 0.640238 | 0.453598 |
| P4-Cz | 0.936485 | 0.824532 | 0.999988 | 65535 | 0.051422 | 0.138764 | 0.170978 |
| P4-Pz | 2.365563 | 0.990183 | 0.999988 | 65535 | 0.098626 | 0.615274 | 0.431891 |
| P4-F9 | 3.442499 | 0.999601 | 0.999988 | 65535 | 0.058474 | 0.761957 | 0.628512 |
| P4-F10 | 3.909057 | 0.999922 | 0.999988 | 65535 | 0.086289 | 0.877967 | 0.713693 |
| O1-O2 | 0.223902 | 0.58839 | 0.999988 | 65535 | 0.05915 | 0.054895 | 0.040879 |
| O1-F7 | -1.80949 | 0.03646 | 0.999988 | 65535 | -0.00289 | 0.599658 | -0.33037 |
| O1-F8 | -0.0419 | 0.483325 | 0.999988 | 65535 | 0.025817 | 0.050274 | -0.00765 |
| O1-T3 | -1.41224 | 0.080256 | 0.999988 | 65535 | 0.006182 | 0.585606 | -0.25784 |
| O1-T4 | 1.742139 | 0.957955 | 0.999988 | 65535 | 0.054123 | 0.364906 | 0.318069 |
| O1-T5 | 2.261755 | 0.987228 | 0.999988 | 65535 | 0.072611 | 0.597738 | 0.412938 |
| O1-T6 | 0.325943 | 0.627478 | 0.999988 | 65535 | 0.028625 | 0.065168 | 0.059509 |
| O1-Fz | 1.298236 | 0.901631 | 0.999988 | 65535 | 0.028918 | 0.217196 | 0.237024 |
| O1-Cz | -2.05366 | 0.021109 | 0.999988 | 65535 | -0.00796 | 0.777827 | -0.37495 |
| O1-Pz | 1.319946 | 0.905296 | 0.999988 | 65535 | 0.054494 | 0.230286 | 0.240988 |
| O1-F9 | 3.149456 | 0.998964 | 0.999988 | 65535 | 0.083973 | 0.840969 | 0.575009 |
| O1-F10 | 1.149191 | 0.873599 | 0.999988 | 65535 | 0.032864 | 0.198515 | 0.209813 |
| O2-F7 | 0.468889 | 0.679993 | 0.999988 | 65535 | 0.036762 | 0.086054 | 0.085607 |
| O2-F8 | 0.214402 | 0.584698 | 0.999988 | 65535 | 0.035635 | 0.056762 | 0.039144 |
| O2-T3 | -0.11302 | 0.455101 | 0.999988 | 65535 | 0.025292 | 0.052033 | -0.02064 |
| O2-T4 | 3.772889 | 0.999873 | 0.999988 | 65535 | 0.081334 | 0.895261 | 0.688832 |
| O2-T5 | 0.810826 | 0.790451 | 0.999988 | 65535 | 0.043701 | 0.119118 | 0.148036 |
| O2-T6 | 3.939366 | 0.999931 | 0.999988 | 65535 | 0.07659 | 0.930663 | 0.719226 |
| O2-Fz | 1.797613 | 0.962602 | 0.999988 | 65535 | 0.035119 | 0.515763 | 0.328198 |
| O2-Cz | -1.14587 | 0.127085 | 0.999988 | 65535 | 0.01166 | 0.587189 | -0.20921 |
| O2-Pz | 1.510885 | 0.933254 | 0.999988 | 65535 | 0.065435 | 0.258846 | 0.275849 |
| O2-F9 | 1.180609 | 0.879934 | 0.999988 | 65535 | 0.029865 | 0.247541 | 0.215548 |
| O2-F10 | 3.821151 | 0.999893 | 0.999988 | 65535 | 0.105045 | 0.846592 | 0.697643 |
| F7-F8 | 2.8766 | 0.997614 | 0.999988 | 65535 | 0.203689 | 0.755569 | 0.525193 |
| F7-T3 | -0.30607 | 0.380045 | 0.999988 | 65535 | 0.056002 | 0.063188 | -0.05588 |
| F7-T4 | 0.407772 | 0.65791 | 0.999988 | 65535 | 0.059023 | 0.070453 | 0.074449 |
| F7-T5 | -1.29481 | 0.098956 | 0.999988 | 65535 | 0.009579 | 0.300983 | -0.2364 |
| F7-T6 | -0.34151 | 0.366664 | 0.999988 | 65535 | 0.02905 | 0.072124 | -0.06235 |
| F7-Fz | 1.440029 | 0.923747 | 0.999988 | 65535 | 0.04022 | 0.33031 | 0.262913 |
| F7-Cz | 1.79672 | 0.962531 | 0.999988 | 65535 | 0.094332 | 0.386485 | 0.328034 |
| F7-Pz | 3.140159 | 0.998933 | 0.999988 | 65535 | 0.100227 | 0.734404 | 0.573312 |
| F7-F9 | -0.17394 | 0.431107 | 0.999988 | 65535 | 0.050105 | 0.05376 | -0.03176 |
| F7-F10 | 1.339689 | 0.90854 | 0.999988 | 65535 | 0.096168 | 0.261429 | 0.244592 |
| F8-T3 | 1.915895 | 0.971101 | 0.999988 | 65535 | 0.106266 | 0.402244 | 0.349793 |
| F8-T4 | -0.82231 | 0.206279 | 0.999988 | 65535 | 0.028495 | 0.123739 | -0.15013 |
| F8-T5 | 1.424803 | 0.921573 | 0.999988 | 65535 | 0.059695 | 0.226002 | 0.260132 |
| F8-T6 | 0.482717 | 0.684904 | 0.999988 | 65535 | 0.046724 | 0.083799 | 0.088131 |
| F8-Fz | 2.128106 | 0.982296 | 0.999988 | 65535 | 0.062671 | 0.510442 | 0.388537 |
| F8-Cz | 2.250216 | 0.986856 | 0.999988 | 65535 | 0.106311 | 0.523505 | 0.410831 |
| F8-Pz | 3.428022 | 0.999581 | 0.999988 | 65535 | 0.108877 | 0.849483 | 0.625868 |
| F8-F9 | -0.77535 | 0.219841 | 0.999988 | 65535 | 0.027285 | 0.119172 | -0.14156 |
| F8-F10 | 3.66024 | 0.999811 | 0.999988 | 65535 | 0.142531 | 0.846007 | 0.668265 |
| T3-T4 | 2.872017 | 0.997582 | 0.999988 | 65535 | 0.091816 | 0.693865 | 0.524356 |
| T3-T5 | 0.485567 | 0.685913 | 0.999988 | 65535 | 0.059122 | 0.076122 | 0.088652 |
| T3-T6 | 0.929039 | 0.822617 | 0.999988 | 65535 | 0.049471 | 0.149393 | 0.169618 |
| T3-Fz | -0.08512 | 0.466157 | 0.999988 | 65535 | 0.02021 | 0.050899 | -0.01554 |
| T3-Cz | 0.084794 | 0.533716 | 0.999988 | 65535 | 0.049863 | 0.051107 | 0.015481 |
| T3-Pz | 2.097981 | 0.98098 | 0.999988 | 65535 | 0.066438 | 0.516624 | 0.383037 |
| T3-F9 | 0.458913 | 0.67643 | 0.999988 | 65535 | 0.066173 | 0.075897 | 0.083786 |
| T3-F10 | 1.761422 | 0.959621 | 0.999988 | 65535 | 0.118737 | 0.515325 | 0.32159 |
| T4-T5 | -0.63931 | 0.261929 | 0.999988 | 65535 | 0.018522 | 0.102382 | -0.11672 |
| T4-T6 | 1.684224 | 0.952609 | 0.999988 | 65535 | 0.090884 | 0.355215 | 0.307496 |
| T4-Fz | 1.836157 | 0.965574 | 0.999988 | 65535 | 0.035001 | 0.375393 | 0.335235 |
| T4-Cz | 0.992011 | 0.838389 | 0.999988 | 65535 | 0.036737 | 0.166546 | 0.181115 |
| T4-Pz | 2.253774 | 0.986972 | 0.999988 | 65535 | 0.059889 | 0.535772 | 0.411481 |
| T4-F9 | -1.04378 | 0.149361 | 0.999988 | 65535 | 0.017697 | 0.15289 | -0.19057 |
| T4-F10 | 0.24063 | 0.59487 | 0.999988 | 65535 | 0.048583 | 0.056231 | 0.043932 |
| T5-T6 | -0.65583 | 0.256605 | 0.999988 | 65535 | 0.013067 | 0.109289 | -0.11974 |
| T5-Fz | 1.932599 | 0.972159 | 0.999988 | 65535 | 0.036737 | 0.450243 | 0.352843 |
| T5-Cz | 1.691453 | 0.953305 | 0.999988 | 65535 | 0.048502 | 0.357796 | 0.308815 |
| T5-Pz | 0.789424 | 0.784276 | 0.999988 | 65535 | 0.04383 | 0.163889 | 0.144128 |
| T5-F9 | 0.07313 | 0.529087 | 0.999988 | 65535 | 0.048399 | 0.050698 | 0.013351 |
| T5-F10 | -1.04674 | 0.14868 | 0.999988 | 65535 | 0.016308 | 0.17385 | -0.19111 |
| T6-Fz | 0.553166 | 0.709402 | 0.999988 | 65535 | 0.018267 | 0.091889 | 0.100994 |
| T6-Cz | 0.141113 | 0.555989 | 0.999988 | 65535 | 0.034041 | 0.05288 | 0.025763 |
| T6-Pz | 0.331641 | 0.629626 | 0.999988 | 65535 | 0.035146 | 0.06871 | 0.060549 |
| T6-F9 | -0.47097 | 0.319266 | 0.999988 | 65535 | 0.029576 | 0.074239 | -0.08599 |
| T6-F10 | 1.099062 | 0.863011 | 0.999988 | 65535 | 0.070279 | 0.197073 | 0.200661 |
| Fz-Cz | -0.83005 | 0.204095 | 0.999988 | 65535 | 0.017777 | 0.163141 | -0.15155 |
| Fz-Pz | 0.748362 | 0.772134 | 0.999988 | 65535 | 0.027297 | 0.123522 | 0.136631 |
| Fz-F9 | 1.348528 | 0.909964 | 0.999988 | 65535 | 0.031828 | 0.237711 | 0.246206 |
| Fz-F10 | 2.264882 | 0.987328 | 0.999988 | 65535 | 0.035099 | 0.544345 | 0.413509 |
| Cz-Pz | 2.116489 | 0.981798 | 0.999988 | 65535 | 0.1122 | 0.443261 | 0.386416 |
| Cz-F9 | 1.382393 | 0.915269 | 0.999988 | 65535 | 0.040552 | 0.273315 | 0.252389 |
| Cz-F10 | 3.765595 | 0.99987 | 0.999988 | 65535 | 0.067166 | 0.858996 | 0.6875 |
| Pz-F9 | 3.495036 | 0.999666 | 0.999988 | 65535 | 0.06509 | 0.811014 | 0.638103 |
| Pz-F10 | 4.087599 | 0.99996 | 0.999988 | 65535 | 0.069982 | 0.904003 | 0.74629 |
| F9-F10 | -1.92889 | 0.028073 | 0.999988 | 65535 | -0.00774 | 0.457916 | -0.35217 |

Table S3c: beta_coh_HC_PT

| channel | tvalue | pvalue | fdr_pvalue | CI_low | CI_high | statistical power | effect size |
| --- | --- | --- | --- | --- | --- | --- | --- |
| Fp1-Fp2 | 1.187395 | 0.118728 | 0.336932 | -0.02464 | 65535 | 0.193338 | 0.216788 |
| Fp1-F3 | 0.011218 | 0.495534 | 0.723985 | -0.05019 | 65535 | 0.050015 | 0.002048 |
| Fp1-F4 | 2.70906 | 0.003876 | 0.101746 | 0.023235 | 65535 | 0.614854 | 0.494604 |
| Fp1-C3 | -0.42542 | 0.664348 | 0.895587 | -0.03818 | 65535 | 0.07316 | -0.07767 |
| Fp1-C4 | 3.747606 | 0.000139 | 0.021537 | 0.027723 | 65535 | 0.798361 | 0.684216 |
| Fp1-P3 | 1.919554 | 0.028664 | 0.184079 | 0.002663 | 65535 | 0.31597 | 0.350461 |
| Fp1-P4 | 0.59853 | 0.275317 | 0.512464 | -0.00697 | 65535 | 0.096736 | 0.109276 |
| Fp1-O1 | -2.42157 | 0.991512 | 0.999922 | -0.06823 | 65535 | 0.797314 | -0.44212 |
| Fp1-O2 | -1.83417 | 0.965426 | 0.999922 | -0.06003 | 65535 | 0.602523 | -0.33487 |
| Fp1-F7 | 1.627428 | 0.053157 | 0.232561 | -0.00134 | 65535 | 0.367826 | 0.297126 |
| Fp1-F8 | 2.965225 | 0.00183 | 0.076881 | 0.046865 | 65535 | 0.70904 | 0.541374 |
| Fp1-T3 | 0.56995 | 0.284898 | 0.512464 | -0.04126 | 65535 | 0.082553 | 0.104058 |
| Fp1-T4 | 1.85569 | 0.032996 | 0.19448 | 0.003787 | 65535 | 0.301278 | 0.338801 |
| Fp1-T5 | -0.79023 | 0.784511 | 0.980729 | -0.05971 | 65535 | 0.129556 | -0.14428 |
| Fp1-T6 | -0.14026 | 0.555653 | 0.799227 | -0.02229 | 65535 | 0.052126 | -0.02561 |
| Fp1-Fz | 2.016944 | 0.022986 | 0.184079 | 0.005585 | 65535 | 0.437258 | 0.368242 |
| Fp1-Cz | 1.127353 | 0.13094 | 0.352531 | -0.01333 | 65535 | 0.209 | 0.205825 |
| Fp1-Pz | 0.670314 | 0.251984 | 0.512464 | -0.01057 | 65535 | 0.096189 | 0.122382 |
| Fp1-F9 | 1.085523 | 0.139951 | 0.361558 | -0.01038 | 65535 | 0.160447 | 0.198189 |
| Fp1-F10 | 1.746667 | 0.041649 | 0.213322 | 0.002005 | 65535 | 0.35315 | 0.318896 |
| Fp2-F3 | 0.904278 | 0.183845 | 0.419647 | -0.01657 | 65535 | 0.129761 | 0.165098 |
| Fp2-F4 | 0.394079 | 0.347117 | 0.583639 | -0.04193 | 65535 | 0.06616 | 0.071949 |
| Fp2-C3 | -0.68426 | 0.752425 | 0.959325 | -0.03929 | 65535 | 0.125147 | -0.12493 |
| Fp2-C4 | 0.699678 | 0.242753 | 0.509781 | -0.01478 | 65535 | 0.10071 | 0.127743 |
| Fp2-P3 | 2.085957 | 0.019569 | 0.184079 | 0.004286 | 65535 | 0.355586 | 0.380842 |
| Fp2-P4 | 2.373718 | 0.009612 | 0.155276 | 0.004686 | 65535 | 0.458266 | 0.43338 |
| Fp2-O1 | -2.54013 | 0.99381 | 0.999922 | -0.07113 | 65535 | 0.8738 | -0.46376 |
| Fp2-O2 | -2.87522 | 0.997604 | 0.999922 | -0.10101 | 65535 | 0.973084 | -0.52494 |
| Fp2-F7 | 0.942305 | 0.173981 | 0.419647 | -0.02699 | 65535 | 0.137128 | 0.17204 |
| Fp2-F8 | -0.13556 | 0.553799 | 0.799227 | -0.07717 | 65535 | 0.051893 | -0.02475 |
| Fp2-T3 | 0.249179 | 0.401828 | 0.62973 | -0.03758 | 65535 | 0.057581 | 0.045494 |
| Fp2-T4 | 0.552914 | 0.290684 | 0.512972 | -0.02924 | 65535 | 0.08227 | 0.100948 |
| Fp2-T5 | 0.288037 | 0.386912 | 0.620241 | -0.01931 | 65535 | 0.056667 | 0.052588 |
| Fp2-T6 | -1.23478 | 0.890317 | 0.999922 | -0.05105 | 65535 | 0.283482 | -0.22544 |
| Fp2-Fz | 0.342602 | 0.366254 | 0.601414 | -0.02865 | 65535 | 0.061741 | 0.06255 |
| Fp2-Cz | 0.569747 | 0.284966 | 0.512464 | -0.02861 | 65535 | 0.092975 | 0.104021 |
| Fp2-Pz | 0.651161 | 0.258104 | 0.512464 | -0.01139 | 65535 | 0.100358 | 0.118885 |
| Fp2-F9 | 0.334122 | 0.36944 | 0.601414 | -0.02971 | 65535 | 0.059737 | 0.061002 |
| Fp2-F10 | 2.065463 | 0.020535 | 0.184079 | 0.007564 | 65535 | 0.404501 | 0.3771 |
| F3-F4 | 1.415696 | 0.079749 | 0.266223 | -0.00486 | 65535 | 0.181357 | 0.258469 |
| F3-C3 | 0.585013 | 0.279828 | 0.512464 | -0.02737 | 65535 | 0.083278 | 0.106808 |
| F3-C4 | 1.604883 | 0.055596 | 0.23827 | -0.00043 | 65535 | 0.383456 | 0.29301 |
| F3-P3 | 0.814576 | 0.208478 | 0.465188 | -0.00658 | 65535 | 0.11487 | 0.148721 |
| F3-P4 | 1.4476 | 0.07519 | 0.261369 | -0.00117 | 65535 | 0.197075 | 0.264294 |
| F3-O1 | -0.85385 | 0.802541 | 0.980729 | -0.01943 | 65535 | 0.15939 | -0.15589 |
| F3-O2 | 0.583739 | 0.280255 | 0.512464 | -0.00719 | 65535 | 0.087651 | 0.106576 |
| F3-F7 | -0.29963 | 0.617506 | 0.864508 | -0.06741 | 65535 | 0.062739 | -0.0547 |
| F3-F8 | 2.518337 | 0.006565 | 0.134617 | 0.018182 | 65535 | 0.564507 | 0.459783 |
| F3-T3 | -0.85647 | 0.803263 | 0.980729 | -0.04236 | 65535 | 0.187315 | -0.15637 |
| F3-T4 | 0.745453 | 0.22874 | 0.490158 | -0.00726 | 65535 | 0.089601 | 0.1361 |
| F3-T5 | -2.01987 | 0.977169 | 0.999922 | -0.04586 | 65535 | 0.949725 | -0.36878 |
| F3-T6 | 1.172544 | 0.12167 | 0.340677 | -0.00299 | 65535 | 0.145153 | 0.214076 |
| F3-Fz | 2.318928 | 0.01106 | 0.165898 | 0.013352 | 65535 | 0.544218 | 0.423377 |
| F3-Cz | -1.07391 | 0.857472 | 0.999922 | -0.04122 | 65535 | 0.385098 | -0.19607 |
| F3-Pz | 0.084302 | 0.46648 | 0.694757 | -0.01032 | 65535 | 0.051137 | 0.015391 |
| F3-F9 | -0.87464 | 0.808226 | 0.981084 | -0.03999 | 65535 | 0.129653 | -0.15969 |
| F3-F10 | -3.1073 | 0.998817 | 0.999922 | -0.07441 | 65535 | 0.960652 | -0.56731 |
| F4-C3 | 1.638284 | 0.052013 | 0.232561 | -0.00015 | 65535 | 0.299565 | 0.299109 |
| F4-C4 | 0.593144 | 0.27711 | 0.512464 | -0.01866 | 65535 | 0.088118 | 0.108293 |
| F4-P3 | 1.850941 | 0.033339 | 0.19448 | 0.000943 | 65535 | 0.341705 | 0.337934 |
| F4-P4 | 1.288785 | 0.099997 | 0.313114 | -0.00215 | 65535 | 0.204774 | 0.235299 |
| F4-O1 | 1.356159 | 0.088819 | 0.283063 | -0.00182 | 65535 | 0.19723 | 0.2476 |
| F4-O2 | -0.22759 | 0.589822 | 0.83691 | -0.0197 | 65535 | 0.056727 | -0.04155 |
| F4-F7 | 1.928726 | 0.028083 | 0.184079 | 0.005525 | 65535 | 0.379415 | 0.352136 |
| F4-F8 | 1.496223 | 0.068633 | 0.248498 | -0.00524 | 65535 | 0.300726 | 0.273172 |
| F4-T3 | 0.914347 | 0.1812 | 0.419647 | -0.00851 | 65535 | 0.122266 | 0.166936 |
| F4-T4 | 1.414891 | 0.079867 | 0.266223 | -0.00297 | 65535 | 0.220842 | 0.258323 |
| F4-T5 | 0.684607 | 0.247467 | 0.511865 | -0.00463 | 65535 | 0.112529 | 0.124991 |
| F4-T6 | -1.41347 | 0.919925 | 0.999922 | -0.02199 | 65535 | 0.4744 | -0.25806 |
| F4-Fz | 2.423557 | 0.008444 | 0.14777 | 0.020659 | 65535 | 0.536626 | 0.442479 |
| F4-Cz | 2.973936 | 0.001783 | 0.076881 | 0.022145 | 65535 | 0.599535 | 0.542964 |
| F4-Pz | 1.164072 | 0.123371 | 0.340895 | -0.00328 | 65535 | 0.193021 | 0.21253 |
| F4-F9 | -0.95256 | 0.828619 | 0.994736 | -0.03216 | 65535 | 0.152921 | -0.17391 |
| F4-F10 | 0.3933 | 0.347404 | 0.583639 | -0.01279 | 65535 | 0.066691 | 0.071806 |
| C3-C4 | 1.271343 | 0.103053 | 0.313641 | -0.00555 | 65535 | 0.151271 | 0.232114 |
| C3-P3 | 1.36299 | 0.087741 | 0.283063 | -0.00652 | 65535 | 0.197226 | 0.248846 |
| C3-P4 | -1.2089 | 0.885441 | 0.999922 | -0.01562 | 65535 | 0.81332 | -0.22071 |
| C3-O1 | -2.6292 | 0.995151 | 0.999922 | -0.05175 | 65535 | 0.999046 | -0.48002 |
| C3-O2 | -2.35357 | 0.989876 | 0.999922 | -0.02326 | 65535 | 0.995844 | -0.4297 |
| C3-F7 | -0.50315 | 0.692101 | 0.919881 | -0.03214 | 65535 | 0.078846 | -0.09186 |
| C3-F8 | 0.167601 | 0.433592 | 0.655067 | -0.01856 | 65535 | 0.053292 | 0.0306 |
| C3-T3 | -1.44211 | 0.92404 | 0.999922 | -0.05636 | 65535 | 0.37068 | -0.26329 |
| C3-T4 | 0.805405 | 0.211103 | 0.465188 | -0.00697 | 65535 | 0.09499 | 0.147046 |
| C3-T5 | 1.713152 | 0.044655 | 0.223277 | 0.000528 | 65535 | 0.350486 | 0.312777 |
| C3-T6 | -1.52633 | 0.935198 | 0.999922 | -0.0229 | 65535 | 0.661976 | -0.27867 |
| C3-Fz | 2.713679 | 0.003826 | 0.101746 | 0.0126 | 65535 | 0.592341 | 0.495447 |
| C3-Cz | -2.02459 | 0.977416 | 0.999922 | -0.06351 | 65535 | 0.922746 | -0.36964 |
| C3-Pz | -1.79979 | 0.962775 | 0.999922 | -0.03382 | 65535 | 0.439611 | -0.32859 |
| C3-F9 | 1.577533 | 0.058675 | 0.240896 | -0.00142 | 65535 | 0.24748 | 0.288017 |
| C3-F10 | -2.43991 | 0.991911 | 0.999922 | -0.02995 | 65535 | 0.859889 | -0.44546 |
| C4-P3 | 1.875948 | 0.031567 | 0.19448 | 0.000773 | 65535 | 0.324202 | 0.3425 |
| C4-P4 | 0.95701 | 0.170259 | 0.41575 | -0.01344 | 65535 | 0.135967 | 0.174725 |
| C4-O1 | 0.463307 | 0.321999 | 0.549754 | -0.00409 | 65535 | 0.075442 | 0.084588 |
| C4-O2 | 0.201288 | 0.42041 | 0.64413 | -0.01237 | 65535 | 0.053685 | 0.03675 |
| C4-F7 | 2.268219 | 0.012567 | 0.175941 | 0.006075 | 65535 | 0.417662 | 0.414118 |
| C4-F8 | -1.65414 | 0.949621 | 0.999922 | -0.04794 | 65535 | 0.348897 | -0.302 |
| C4-T3 | 2.023497 | 0.022641 | 0.184079 | 0.003125 | 65535 | 0.34953 | 0.369438 |
| C4-T4 | -3.26132 | 0.999275 | 0.999922 | -0.07155 | 65535 | 0.995037 | -0.59543 |
| C4-T5 | 0.493428 | 0.311314 | 0.535868 | -0.00523 | 65535 | 0.082879 | 0.090087 |
| C4-T6 | 3.50869 | 0.000319 | 0.022338 | 0.019253 | 65535 | 0.752522 | 0.640596 |
| C4-Fz | 1.652036 | 0.050593 | 0.232561 | -7.8E-05 | 65535 | 0.281255 | 0.301619 |
| C4-Cz | 2.491691 | 0.007051 | 0.134617 | 0.015889 | 65535 | 0.503123 | 0.454918 |
| C4-Pz | 0.594536 | 0.276646 | 0.512464 | -0.01425 | 65535 | 0.085481 | 0.108547 |
| C4-F9 | -2.19312 | 0.98487 | 0.999922 | -0.0291 | 65535 | 0.824236 | -0.40041 |
| C4-F10 | 1.56695 | 0.059902 | 0.240896 | -0.00096 | 65535 | 0.261875 | 0.286085 |
| P3-P4 | 1.665543 | 0.049229 | 0.232561 | 3.66E-05 | 65535 | 0.283984 | 0.304085 |
| P3-O1 | 0.792353 | 0.214873 | 0.465188 | -0.01373 | 65535 | 0.126758 | 0.144663 |
| P3-O2 | 0.559771 | 0.288348 | 0.512972 | -0.00566 | 65535 | 0.081742 | 0.1022 |
| P3-F7 | 1.189871 | 0.118243 | 0.336932 | -0.00506 | 65535 | 0.182928 | 0.21724 |
| P3-F8 | 1.960617 | 0.02614 | 0.184079 | 0.002396 | 65535 | 0.345227 | 0.357958 |
| P3-T3 | -1.28983 | 0.900184 | 0.999922 | -0.04786 | 65535 | 0.438788 | -0.23549 |
| P3-T4 | 1.513637 | 0.066396 | 0.248498 | -0.00091 | 65535 | 0.22512 | 0.276351 |
| P3-T5 | 1.990693 | 0.024413 | 0.184079 | 0.006368 | 65535 | 0.467226 | 0.363448 |
| P3-T6 | 0.568123 | 0.285515 | 0.512464 | -0.00651 | 65535 | 0.07984 | 0.103725 |
| P3-Fz | 2.566012 | 0.00577 | 0.134617 | 0.004667 | 65535 | 0.560904 | 0.468487 |
| P3-Cz | 1.786725 | 0.038275 | 0.207771 | 0.001204 | 65535 | 0.410025 | 0.32621 |
| P3-Pz | 2.155585 | 0.016573 | 0.180394 | 0.006831 | 65535 | 0.513855 | 0.393554 |
| P3-F9 | 1.981071 | 0.024955 | 0.184079 | 0.002211 | 65535 | 0.443466 | 0.361692 |
| P3-F10 | 0.505002 | 0.30725 | 0.533243 | -0.00636 | 65535 | 0.071959 | 0.0922 |
| P4-O1 | 2.121885 | 0.017969 | 0.180394 | 0.003007 | 65535 | 0.417907 | 0.387401 |
| P4-O2 | 1.280791 | 0.101389 | 0.313114 | -0.00481 | 65535 | 0.264358 | 0.233839 |
| P4-F7 | 1.772094 | 0.03948 | 0.207771 | 0.000572 | 65535 | 0.280964 | 0.323539 |
| P4-F8 | 1.555771 | 0.06122 | 0.240896 | -0.00051 | 65535 | 0.31317 | 0.284044 |
| P4-T3 | 0.620305 | 0.268127 | 0.512464 | -0.00957 | 65535 | 0.092564 | 0.113252 |
| P4-T4 | 0.012617 | 0.494977 | 0.723985 | -0.01876 | 65535 | 0.050017 | 0.002303 |
| P4-T5 | 0.537853 | 0.295846 | 0.51773 | -0.0069 | 65535 | 0.077093 | 0.098198 |
| P4-T6 | 1.80847 | 0.03654 | 0.207389 | 0.002961 | 65535 | 0.413763 | 0.33018 |
| P4-Fz | 0.272352 | 0.392914 | 0.62509 | -0.00665 | 65535 | 0.06467 | 0.049724 |
| P4-Cz | 1.004043 | 0.158706 | 0.396765 | -0.00748 | 65535 | 0.144821 | 0.183312 |
| P4-Pz | 0.905804 | 0.183443 | 0.419647 | -0.00998 | 65535 | 0.131559 | 0.165376 |
| P4-F9 | 2.884473 | 0.002331 | 0.081583 | 0.00477 | 65535 | 0.655518 | 0.526631 |
| P4-F10 | 0.989862 | 0.162133 | 0.400565 | -0.00471 | 65535 | 0.12612 | 0.180723 |
| O1-O2 | 0.921858 | 0.179242 | 0.419647 | -0.01729 | 65535 | 0.140083 | 0.168308 |
| O1-F7 | -3.90848 | 0.999922 | 0.999922 | -0.10174 | 65535 | 0.999412 | -0.71359 |
| O1-F8 | -1.32635 | 0.906357 | 0.999922 | -0.04342 | 65535 | 0.49811 | -0.24216 |
| O1-T3 | -2.83744 | 0.997323 | 0.999922 | -0.09792 | 65535 | 0.999952 | -0.51804 |
| O1-T4 | -0.61884 | 0.731393 | 0.953991 | -0.01885 | 65535 | 0.110613 | -0.11298 |
| O1-T5 | 1.072234 | 0.142902 | 0.361558 | -0.00801 | 65535 | 0.214497 | 0.195762 |
| O1-T6 | 1.959686 | 0.026195 | 0.184079 | 0.003292 | 65535 | 0.54428 | 0.357788 |
| O1-Fz | 1.562585 | 0.060414 | 0.240896 | -0.00032 | 65535 | 0.254852 | 0.285288 |
| O1-Cz | -2.90736 | 0.997822 | 0.999922 | -0.07637 | 65535 | 1 | -0.53081 |
| O1-Pz | 1.549708 | 0.061945 | 0.240896 | -0.00084 | 65535 | 0.321485 | 0.282937 |
| O1-F9 | 0.220496 | 0.412933 | 0.64234 | -0.01701 | 65535 | 0.062438 | 0.040257 |
| O1-F10 | 0.19739 | 0.421931 | 0.64413 | -0.01412 | 65535 | 0.055203 | 0.036038 |
| O2-F7 | -1.33324 | 0.907489 | 0.999922 | -0.04129 | 65535 | 0.290568 | -0.24341 |
| O2-F8 | -3.0267 | 0.998482 | 0.999922 | -0.08598 | 65535 | 0.921735 | -0.5526 |
| O2-T3 | -2.91566 | 0.997876 | 0.999922 | -0.05484 | 65535 | 0.99993 | -0.53232 |
| O2-T4 | 1.533329 | 0.063936 | 0.244118 | -0.00112 | 65535 | 0.275211 | 0.279947 |
| O2-T5 | 1.635392 | 0.052316 | 0.232561 | -0.00026 | 65535 | 0.349891 | 0.29858 |
| O2-T6 | 1.442386 | 0.075921 | 0.261369 | -0.00259 | 65535 | 0.277092 | 0.263342 |
| O2-Fz | 1.104634 | 0.135783 | 0.360942 | -0.00234 | 65535 | 0.157264 | 0.201677 |
| O2-Cz | -2.57636 | 0.994391 | 0.999922 | -0.07641 | 65535 | 1 | -0.47038 |
| O2-Pz | 0.854698 | 0.197225 | 0.445347 | -0.00943 | 65535 | 0.106236 | 0.156046 |
| O2-F9 | -0.45096 | 0.673576 | 0.900961 | -0.01814 | 65535 | 0.07155 | -0.08233 |
| O2-F10 | 1.639653 | 0.05187 | 0.232561 | -0.00019 | 65535 | 0.376526 | 0.299358 |
| F7-F8 | 2.120258 | 0.018039 | 0.180394 | 0.02153 | 65535 | 0.47382 | 0.387104 |
| F7-T3 | -0.85017 | 0.801523 | 0.980729 | -0.11333 | 65535 | 0.164614 | -0.15522 |
| F7-T4 | 0.128095 | 0.449146 | 0.673719 | -0.03669 | 65535 | 0.051818 | 0.023387 |
| F7-T5 | -3.03846 | 0.998536 | 0.999922 | -0.09437 | 65535 | 0.888374 | -0.55474 |
| F7-T6 | -1.8159 | 0.964038 | 0.999922 | -0.06179 | 65535 | 0.879405 | -0.33154 |
| F7-Fz | -0.54727 | 0.707388 | 0.934286 | -0.01958 | 65535 | 0.088965 | -0.09992 |
| F7-Cz | 0.378297 | 0.352945 | 0.588241 | -0.02566 | 65535 | 0.066597 | 0.069067 |
| F7-Pz | 0.730002 | 0.233418 | 0.495129 | -0.00885 | 65535 | 0.09957 | 0.133279 |
| F7-F9 | -0.42802 | 0.665293 | 0.895587 | -0.06713 | 65535 | 0.074116 | -0.07815 |
| F7-F10 | 0.568251 | 0.285472 | 0.512464 | -0.03104 | 65535 | 0.090754 | 0.103749 |
| F8-T3 | 0.578808 | 0.281911 | 0.512464 | -0.02843 | 65535 | 0.084416 | 0.105676 |
| F8-T4 | -0.34266 | 0.633767 | 0.881397 | -0.06236 | 65535 | 0.061935 | -0.06256 |
| F8-T5 | 0.630043 | 0.264942 | 0.512464 | -0.01303 | 65535 | 0.085834 | 0.11503 |
| F8-T6 | -3.03826 | 0.998535 | 0.999922 | -0.08074 | 65535 | 0.94404 | -0.55471 |
| F8-Fz | 0.906835 | 0.183171 | 0.419647 | -0.00648 | 65535 | 0.149258 | 0.165565 |
| F8-Cz | 1.476524 | 0.071234 | 0.253543 | -0.00365 | 65535 | 0.264837 | 0.269575 |
| F8-Pz | 1.225266 | 0.111458 | 0.325085 | -0.00359 | 65535 | 0.243989 | 0.223702 |
| F8-F9 | -1.26369 | 0.895584 | 0.999922 | -0.0824 | 65535 | 0.237572 | -0.23072 |
| F8-F10 | 2.133959 | 0.017458 | 0.180394 | 0.012452 | 65535 | 0.451743 | 0.389606 |
| T3-T4 | 2.162991 | 0.016279 | 0.180394 | 0.008573 | 65535 | 0.490453 | 0.394906 |
| T3-T5 | -0.56108 | 0.712095 | 0.934625 | -0.05866 | 65535 | 0.092848 | -0.10244 |
| T3-T6 | 0.793458 | 0.214552 | 0.465188 | -0.01189 | 65535 | 0.130418 | 0.144865 |
| T3-Fz | -0.39328 | 0.65259 | 0.889896 | -0.01155 | 65535 | 0.069836 | -0.0718 |
| T3-Cz | -0.66315 | 0.745735 | 0.959325 | -0.05431 | 65535 | 0.14747 | -0.12107 |
| T3-Pz | -1.67018 | 0.951233 | 0.999922 | -0.03123 | 65535 | 0.979202 | -0.30493 |
| T3-F9 | 1.09416 | 0.138057 | 0.361558 | -0.0158 | 65535 | 0.200181 | 0.199765 |
| T3-F10 | 1.930344 | 0.027982 | 0.184079 | 0.009 | 65535 | 0.497552 | 0.352431 |
| T4-T5 | -0.95385 | 0.828946 | 0.994736 | -0.03611 | 65535 | 0.19077 | -0.17415 |
| T4-T6 | 0.60082 | 0.274556 | 0.512464 | -0.02562 | 65535 | 0.088798 | 0.109694 |
| T4-Fz | 1.915464 | 0.028927 | 0.184079 | 0.00112 | 65535 | 0.324587 | 0.349714 |
| T4-Cz | 1.502266 | 0.06785 | 0.248498 | -0.00067 | 65535 | 0.257251 | 0.274275 |
| T4-Pz | 1.07584 | 0.142097 | 0.361558 | -0.00392 | 65535 | 0.177818 | 0.19642 |
| T4-F9 | -0.36349 | 0.641555 | 0.886358 | -0.05943 | 65535 | 0.064604 | -0.06636 |
| T4-F10 | 0.008926 | 0.496447 | 0.723985 | -0.03868 | 65535 | 0.050008 | 0.001629 |
| T5-T6 | -0.84024 | 0.798764 | 0.980729 | -0.02037 | 65535 | 0.12999 | -0.15341 |
| T5-Fz | 1.230289 | 0.110518 | 0.325085 | -0.00189 | 65535 | 0.166178 | 0.224619 |
| T5-Cz | -0.65643 | 0.743587 | 0.959325 | -0.01979 | 65535 | 0.185422 | -0.11985 |
| T5-Pz | -0.20274 | 0.580156 | 0.828795 | -0.02174 | 65535 | 0.06057 | -0.03702 |
| T5-F9 | 0.257361 | 0.398674 | 0.629486 | -0.03278 | 65535 | 0.057337 | 0.046987 |
| T5-F10 | -0.76866 | 0.778185 | 0.980729 | -0.05556 | 65535 | 0.113769 | -0.14034 |
| T6-Fz | 1.155567 | 0.125096 | 0.341172 | -0.00212 | 65535 | 0.202733 | 0.210977 |
| T6-Cz | -1.19605 | 0.882963 | 0.999922 | -0.0425 | 65535 | 0.690058 | -0.21837 |
| T6-Pz | -0.6885 | 0.753755 | 0.959325 | -0.02583 | 65535 | 0.189188 | -0.1257 |
| T6-F9 | -0.83822 | 0.798199 | 0.980729 | -0.05335 | 65535 | 0.131548 | -0.15304 |
| T6-F10 | 3.637015 | 0.000205 | 0.021537 | 0.038699 | 65535 | 0.911021 | 0.664025 |
| Fz-Cz | 0.301062 | 0.381949 | 0.616994 | -0.02424 | 65535 | 0.059787 | 0.054966 |
| Fz-Pz | -0.24006 | 0.59465 | 0.838097 | -0.01249 | 65535 | 0.057663 | -0.04383 |
| Fz-F9 | 1.770951 | 0.039575 | 0.207771 | 0.000459 | 65535 | 0.38631 | 0.32333 |
| Fz-F10 | 0.33658 | 0.368516 | 0.601414 | -0.00626 | 65535 | 0.062356 | 0.061451 |
| Cz-Pz | 2.129792 | 0.017633 | 0.180394 | 0.009314 | 65535 | 0.418745 | 0.388845 |
| Cz-F9 | -0.38321 | 0.648874 | 0.889896 | -0.01944 | 65535 | 0.110514 | -0.06996 |
| Cz-F10 | 0.680945 | 0.24862 | 0.511865 | -0.00808 | 65535 | 0.130713 | 0.124323 |
| Pz-F9 | 1.244405 | 0.107908 | 0.323725 | -0.00173 | 65535 | 0.22392 | 0.227196 |
| Pz-F10 | 1.355253 | 0.088963 | 0.283063 | -0.00118 | 65535 | 0.256011 | 0.247434 |
| F9-F10 | 0.193921 | 0.423286 | 0.64413 | -0.04227 | 65535 | 0.053485 | 0.035405 |

Table S3d: beta_coh_PT_HC

| channel | tvalue | pvalue | fdr_pvalue | CI_low | CI_high | statistical power | effect size |
| --- | --- | --- | --- | --- | --- | --- | --- |
| Fp1-Fp2 | 1.187395 | 0.881272 | 0.999861 | 65535 | 0.149039 | 0.193338 | 0.216788 |
| Fp1-F3 | 0.011218 | 0.504466 | 0.999861 | 65535 | 0.050877 | 0.050015 | 0.002048 |
| Fp1-F4 | 2.70906 | 0.996124 | 0.999861 | 65535 | 0.096523 | 0.614854 | 0.494604 |
| Fp1-C3 | -0.42542 | 0.335652 | 0.999861 | 65535 | 0.022584 | 0.07316 | -0.07767 |
| Fp1-C4 | 3.747606 | 0.999861 | 0.999861 | 65535 | 0.071711 | 0.798361 | 0.684216 |
| Fp1-P3 | 1.919554 | 0.971336 | 0.999861 | 65535 | 0.036399 | 0.31597 | 0.350461 |
| Fp1-P4 | 0.59853 | 0.724683 | 0.999861 | 65535 | 0.014849 | 0.096736 | 0.109276 |
| Fp1-O1 | -2.42157 | 0.008488 | 0.118831 | 65535 | -0.01277 | 0.797314 | -0.44212 |
| Fp1-O2 | -1.83417 | 0.034574 | 0.355329 | 65535 | -0.00303 | 0.602523 | -0.33487 |
| Fp1-F7 | 1.627428 | 0.946843 | 0.999861 | 65535 | 0.145113 | 0.367826 | 0.297126 |
| Fp1-F8 | 2.965225 | 0.998169 | 0.999861 | 65535 | 0.165726 | 0.70904 | 0.541374 |
| Fp1-T3 | 0.56995 | 0.715102 | 0.999861 | 65535 | 0.084494 | 0.082553 | 0.104058 |
| Fp1-T4 | 1.85569 | 0.967004 | 0.999861 | 65535 | 0.067265 | 0.301278 | 0.338801 |
| Fp1-T5 | -0.79023 | 0.215489 | 0.999861 | 65535 | 0.021162 | 0.129556 | -0.14428 |
| Fp1-T6 | -0.14026 | 0.444347 | 0.999861 | 65535 | 0.018814 | 0.052126 | -0.02561 |
| Fp1-Fz | 2.016944 | 0.977014 | 0.999861 | 65535 | 0.057154 | 0.437258 | 0.368242 |
| Fp1-Cz | 1.127353 | 0.86906 | 0.999861 | 65535 | 0.069979 | 0.209 | 0.205825 |
| Fp1-Pz | 0.670314 | 0.748016 | 0.999861 | 65535 | 0.02492 | 0.096189 | 0.122382 |
| Fp1-F9 | 1.085523 | 0.860049 | 0.999861 | 65535 | 0.049766 | 0.160447 | 0.198189 |
| Fp1-F10 | 1.746667 | 0.958351 | 0.999861 | 65535 | 0.076867 | 0.35315 | 0.318896 |
| Fp2-F3 | 0.904278 | 0.816155 | 0.999861 | 65535 | 0.056346 | 0.129761 | 0.165098 |
| Fp2-F4 | 0.394079 | 0.652883 | 0.999861 | 65535 | 0.068083 | 0.06616 | 0.071949 |
| Fp2-C3 | -0.68426 | 0.247575 | 0.999861 | 65535 | 0.016333 | 0.125147 | -0.12493 |
| Fp2-C4 | 0.699678 | 0.757247 | 0.999861 | 65535 | 0.036372 | 0.10071 | 0.127743 |
| Fp2-P3 | 2.085957 | 0.980431 | 0.999861 | 65535 | 0.037483 | 0.355586 | 0.380842 |
| Fp2-P4 | 2.373718 | 0.990388 | 0.999861 | 65535 | 0.026389 | 0.458266 | 0.43338 |
| Fp2-O1 | -2.54013 | 0.00619 | 0.099994 | 65535 | -0.01495 | 0.8738 | -0.46376 |
| Fp2-O2 | -2.87522 | 0.002396 | 0.055899 | 65535 | -0.02713 | 0.973084 | -0.52494 |
| Fp2-F7 | 0.942305 | 0.826019 | 0.999861 | 65535 | 0.098086 | 0.137128 | 0.17204 |
| Fp2-F8 | -0.13556 | 0.446201 | 0.999861 | 65535 | 0.0655 | 0.051893 | -0.02475 |
| Fp2-T3 | 0.249179 | 0.598172 | 0.999861 | 65535 | 0.050874 | 0.057581 | 0.045494 |
| Fp2-T4 | 0.552914 | 0.709316 | 0.999861 | 65535 | 0.058495 | 0.08227 | 0.100948 |
| Fp2-T5 | 0.288037 | 0.613088 | 0.999861 | 65535 | 0.027429 | 0.056667 | 0.052588 |
| Fp2-T6 | -1.23478 | 0.109683 | 0.719795 | 65535 | 0.007466 | 0.283482 | -0.22544 |
| Fp2-Fz | 0.342602 | 0.633746 | 0.999861 | 65535 | 0.043571 | 0.061741 | 0.06255 |
| Fp2-Cz | 0.569747 | 0.715034 | 0.999861 | 65535 | 0.058563 | 0.092975 | 0.104021 |
| Fp2-Pz | 0.651161 | 0.741896 | 0.999861 | 65535 | 0.026132 | 0.100358 | 0.118885 |
| Fp2-F9 | 0.334122 | 0.63056 | 0.999861 | 65535 | 0.044708 | 0.059737 | 0.061002 |
| Fp2-F10 | 2.065463 | 0.979465 | 0.999861 | 65535 | 0.069098 | 0.404501 | 0.3771 |
| F3-F4 | 1.415696 | 0.920251 | 0.999861 | 65535 | 0.061626 | 0.181357 | 0.258469 |
| F3-C3 | 0.585013 | 0.720172 | 0.999861 | 65535 | 0.057213 | 0.083278 | 0.106808 |
| F3-C4 | 1.604883 | 0.944404 | 0.999861 | 65535 | 0.026677 | 0.383456 | 0.29301 |
| F3-P3 | 0.814576 | 0.791522 | 0.999861 | 65535 | 0.019291 | 0.11487 | 0.148721 |
| F3-P4 | 1.4476 | 0.92481 | 0.999861 | 65535 | 0.017265 | 0.197075 | 0.264294 |
| F3-O1 | -0.85385 | 0.197459 | 0.985537 | 65535 | 0.006221 | 0.15939 | -0.15589 |
| F3-O2 | 0.583739 | 0.719745 | 0.999861 | 65535 | 0.015015 | 0.087651 | 0.106576 |
| F3-F7 | -0.29963 | 0.382494 | 0.999861 | 65535 | 0.046772 | 0.062739 | -0.0547 |
| F3-F8 | 2.518337 | 0.993435 | 0.999861 | 65535 | 0.088243 | 0.564507 | 0.459783 |
| F3-T3 | -0.85647 | 0.196737 | 0.985537 | 65535 | 0.0135 | 0.187315 | -0.15637 |
| F3-T4 | 0.745453 | 0.77126 | 0.999861 | 65535 | 0.019119 | 0.089601 | 0.1361 |
| F3-T5 | -2.01987 | 0.022831 | 0.252346 | 65535 | -0.00451 | 0.949725 | -0.36878 |
| F3-T6 | 1.172544 | 0.87833 | 0.999861 | 65535 | 0.01745 | 0.145153 | 0.214076 |
| F3-Fz | 2.318928 | 0.98894 | 0.999861 | 65535 | 0.080323 | 0.544218 | 0.423377 |
| F3-Cz | -1.07391 | 0.142528 | 0.855166 | 65535 | 0.008811 | 0.385098 | -0.19607 |
| F3-Pz | 0.084302 | 0.53352 | 0.999861 | 65535 | 0.011431 | 0.051137 | 0.015391 |
| F3-F9 | -0.87464 | 0.191774 | 0.985537 | 65535 | 0.012367 | 0.129653 | -0.15969 |
| F3-F10 | -3.1073 | 0.001183 | 0.053131 | 65535 | -0.02263 | 0.960652 | -0.56731 |
| F4-C3 | 1.638284 | 0.947987 | 0.999861 | 65535 | 0.025876 | 0.299565 | 0.299109 |
| F4-C4 | 0.593144 | 0.72289 | 0.999861 | 65535 | 0.039457 | 0.088118 | 0.108293 |
| F4-P3 | 1.850941 | 0.966661 | 0.999861 | 65535 | 0.017135 | 0.341705 | 0.337934 |
| F4-P4 | 1.288785 | 0.900003 | 0.999861 | 65535 | 0.017136 | 0.204774 | 0.235299 |
| F4-O1 | 1.356159 | 0.911181 | 0.999861 | 65535 | 0.018197 | 0.19723 | 0.2476 |
| F4-O2 | -0.22759 | 0.410178 | 0.999861 | 65535 | 0.014945 | 0.056727 | -0.04155 |
| F4-F7 | 1.928726 | 0.971917 | 0.999861 | 65535 | 0.073164 | 0.379415 | 0.352136 |
| F4-F8 | 1.496223 | 0.931367 | 0.999861 | 65535 | 0.102307 | 0.300726 | 0.273172 |
| F4-T3 | 0.914347 | 0.8188 | 0.999861 | 65535 | 0.029432 | 0.122266 | 0.166936 |
| F4-T4 | 1.414891 | 0.920133 | 0.999861 | 65535 | 0.037564 | 0.220842 | 0.258323 |
| F4-T5 | 0.684607 | 0.752533 | 0.999861 | 65535 | 0.011152 | 0.112529 | 0.124991 |
| F4-T6 | -1.41347 | 0.080075 | 0.622806 | 65535 | 0.00175 | 0.4744 | -0.25806 |
| F4-Fz | 2.423557 | 0.991556 | 0.999861 | 65535 | 0.110122 | 0.536626 | 0.442479 |
| F4-Cz | 2.973936 | 0.998217 | 0.999861 | 65535 | 0.077937 | 0.599535 | 0.542964 |
| F4-Pz | 1.164072 | 0.876629 | 0.999861 | 65535 | 0.01875 | 0.193021 | 0.21253 |
| F4-F9 | -0.95256 | 0.171381 | 0.9727 | 65535 | 0.008691 | 0.152921 | -0.17391 |
| F4-F10 | 0.3933 | 0.652596 | 0.999861 | 65535 | 0.020745 | 0.066691 | 0.071806 |
| C3-C4 | 1.271343 | 0.896947 | 0.999861 | 65535 | 0.042077 | 0.151271 | 0.232114 |
| C3-P3 | 1.36299 | 0.912259 | 0.999861 | 65535 | 0.066785 | 0.197226 | 0.248846 |
| C3-P4 | -1.2089 | 0.114559 | 0.722877 | 65535 | 0.002447 | 0.81332 | -0.22071 |
| C3-O1 | -2.6292 | 0.004849 | 0.092572 | 65535 | -0.01173 | 0.999046 | -0.48002 |
| C3-O2 | -2.35357 | 0.010124 | 0.132876 | 65535 | -0.00403 | 0.995844 | -0.4297 |
| C3-F7 | -0.50315 | 0.307899 | 0.999861 | 65535 | 0.017175 | 0.078846 | -0.09186 |
| C3-F8 | 0.167601 | 0.566408 | 0.999861 | 65535 | 0.022735 | 0.053292 | 0.0306 |
| C3-T3 | -1.44211 | 0.07596 | 0.613521 | 65535 | 0.003923 | 0.37068 | -0.26329 |
| C3-T4 | 0.805405 | 0.788897 | 0.999861 | 65535 | 0.020129 | 0.09499 | 0.147046 |
| C3-T5 | 1.713152 | 0.955345 | 0.999861 | 65535 | 0.032186 | 0.350486 | 0.312777 |
| C3-T6 | -1.52633 | 0.064802 | 0.544337 | 65535 | 0.000946 | 0.661976 | -0.27867 |
| C3-Fz | 2.713679 | 0.996174 | 0.999861 | 65535 | 0.052171 | 0.592341 | 0.495447 |
| C3-Cz | -2.02459 | 0.022584 | 0.252346 | 65535 | -0.00632 | 0.922746 | -0.36964 |
| C3-Pz | -1.79979 | 0.037225 | 0.355329 | 65535 | -0.00139 | 0.439611 | -0.32859 |
| C3-F9 | 1.577533 | 0.941325 | 0.999861 | 65535 | 0.057008 | 0.24748 | 0.288017 |
| C3-F10 | -2.43991 | 0.008089 | 0.118831 | 65535 | -0.00572 | 0.859889 | -0.44546 |
| C4-P3 | 1.875948 | 0.968433 | 0.999861 | 65535 | 0.012526 | 0.324202 | 0.3425 |
| C4-P4 | 0.95701 | 0.829741 | 0.999861 | 65535 | 0.05013 | 0.135967 | 0.174725 |
| C4-O1 | 0.463307 | 0.678001 | 0.999861 | 65535 | 0.007268 | 0.075442 | 0.084588 |
| C4-O2 | 0.201288 | 0.57959 | 0.999861 | 65535 | 0.015795 | 0.053685 | 0.03675 |
| C4-F7 | 2.268219 | 0.987433 | 0.999861 | 65535 | 0.039077 | 0.417662 | 0.414118 |
| C4-F8 | -1.65414 | 0.050379 | 0.440814 | 65535 | 5.4E-05 | 0.348897 | -0.302 |
| C4-T3 | 2.023497 | 0.977359 | 0.999861 | 65535 | 0.03146 | 0.34953 | 0.369438 |
| C4-T4 | -3.26132 | 0.000725 | 0.053131 | 65535 | -0.02332 | 0.995037 | -0.59543 |
| C4-T5 | 0.493428 | 0.688686 | 0.999861 | 65535 | 0.009671 | 0.082879 | 0.090087 |
| C4-T6 | 3.50869 | 0.999681 | 0.999861 | 65535 | 0.053746 | 0.752522 | 0.640596 |
| C4-Fz | 1.652036 | 0.949407 | 0.999861 | 65535 | 0.044124 | 0.281255 | 0.301619 |
| C4-Cz | 2.491691 | 0.992949 | 0.999861 | 65535 | 0.079074 | 0.503123 | 0.454918 |
| C4-Pz | 0.594536 | 0.723354 | 0.999861 | 65535 | 0.03018 | 0.085481 | 0.108547 |
| C4-F9 | -2.19312 | 0.01513 | 0.186899 | 65535 | -0.00404 | 0.824236 | -0.40041 |
| C4-F10 | 1.56695 | 0.940098 | 0.999861 | 65535 | 0.034218 | 0.261875 | 0.286085 |
| P3-P4 | 1.665543 | 0.950771 | 0.999861 | 65535 | 0.015838 | 0.283984 | 0.304085 |
| P3-O1 | 0.792353 | 0.785127 | 0.999861 | 65535 | 0.038856 | 0.126758 | 0.144663 |
| P3-O2 | 0.559771 | 0.711652 | 0.999861 | 65535 | 0.011422 | 0.081742 | 0.1022 |
| P3-F7 | 1.189871 | 0.881757 | 0.999861 | 65535 | 0.030817 | 0.182928 | 0.21724 |
| P3-F8 | 1.960617 | 0.97386 | 0.999861 | 65535 | 0.028639 | 0.345227 | 0.357958 |
| P3-T3 | -1.28983 | 0.099816 | 0.69871 | 65535 | 0.005975 | 0.438788 | -0.23549 |
| P3-T4 | 1.513637 | 0.933604 | 0.999861 | 65535 | 0.020097 | 0.22512 | 0.276351 |
| P3-T5 | 1.990693 | 0.975587 | 0.999861 | 65535 | 0.069804 | 0.467226 | 0.363448 |
| P3-T6 | 0.568123 | 0.714485 | 0.999861 | 65535 | 0.013288 | 0.07984 | 0.103725 |
| P3-Fz | 2.566012 | 0.99423 | 0.999861 | 65535 | 0.021706 | 0.560904 | 0.468487 |
| P3-Cz | 1.786725 | 0.961725 | 0.999861 | 65535 | 0.032178 | 0.410025 | 0.32621 |
| P3-Pz | 2.155585 | 0.983427 | 0.999861 | 65535 | 0.05234 | 0.513855 | 0.393554 |
| P3-F9 | 1.981071 | 0.975045 | 0.999861 | 65535 | 0.024896 | 0.443466 | 0.361692 |
| P3-F10 | 0.505002 | 0.69275 | 0.999861 | 65535 | 0.011924 | 0.071959 | 0.0922 |
| P4-O1 | 2.121885 | 0.982031 | 0.999861 | 65535 | 0.024491 | 0.417907 | 0.387401 |
| P4-O2 | 1.280791 | 0.898611 | 0.999861 | 65535 | 0.037465 | 0.264358 | 0.233839 |
| P4-F7 | 1.772094 | 0.96052 | 0.999861 | 65535 | 0.017177 | 0.280964 | 0.323539 |
| P4-F8 | 1.555771 | 0.93878 | 0.999861 | 65535 | 0.016075 | 0.31317 | 0.284044 |
| P4-T3 | 0.620305 | 0.731873 | 0.999861 | 65535 | 0.021004 | 0.092564 | 0.113252 |
| P4-T4 | 0.012617 | 0.505023 | 0.999861 | 65535 | 0.01905 | 0.050017 | 0.002303 |
| P4-T5 | 0.537853 | 0.704154 | 0.999861 | 65535 | 0.013519 | 0.077093 | 0.098198 |
| P4-T6 | 1.80847 | 0.96346 | 0.999861 | 65535 | 0.068159 | 0.413763 | 0.33018 |
| P4-Fz | 0.272352 | 0.607086 | 0.999861 | 65535 | 0.009266 | 0.06467 | 0.049724 |
| P4-Cz | 1.004043 | 0.841294 | 0.999861 | 65535 | 0.030473 | 0.144821 | 0.183312 |
| P4-Pz | 0.905804 | 0.816557 | 0.999861 | 65535 | 0.034005 | 0.131559 | 0.165376 |
| P4-F9 | 2.884473 | 0.997669 | 0.999861 | 65535 | 0.017665 | 0.655518 | 0.526631 |
| P4-F10 | 0.989862 | 0.837866 | 0.999861 | 65535 | 0.018671 | 0.12612 | 0.180723 |
| O1-O2 | 0.921858 | 0.820758 | 0.999861 | 65535 | 0.060617 | 0.140083 | 0.168308 |
| O1-F7 | -3.90848 | 7.77E-05 | 0.016322 | 65535 | -0.04114 | 0.999412 | -0.71359 |
| O1-F8 | -1.32635 | 0.093643 | 0.678101 | 65535 | 0.004823 | 0.49811 | -0.24216 |
| O1-T3 | -2.83744 | 0.002677 | 0.056225 | 65535 | -0.02569 | 0.999952 | -0.51804 |
| O1-T4 | -0.61884 | 0.268607 | 0.999861 | 65535 | 0.0086 | 0.110613 | -0.11298 |
| O1-T5 | 1.072234 | 0.857098 | 0.999861 | 65535 | 0.037352 | 0.214497 | 0.195762 |
| O1-T6 | 1.959686 | 0.973805 | 0.999861 | 65535 | 0.039457 | 0.54428 | 0.357788 |
| O1-Fz | 1.562585 | 0.939586 | 0.999861 | 65535 | 0.01086 | 0.254852 | 0.285288 |
| O1-Cz | -2.90736 | 0.002178 | 0.055899 | 65535 | -0.0209 | 1 | -0.53081 |
| O1-Pz | 1.549708 | 0.938055 | 0.999861 | 65535 | 0.024901 | 0.321485 | 0.282937 |
| O1-F9 | 0.220496 | 0.587067 | 0.999861 | 65535 | 0.02223 | 0.062438 | 0.040257 |
| O1-F10 | 0.19739 | 0.578069 | 0.999861 | 65535 | 0.017931 | 0.055203 | 0.036038 |
| O2-F7 | -1.33324 | 0.092511 | 0.678101 | 65535 | 0.004481 | 0.290568 | -0.24341 |
| O2-F8 | -3.0267 | 0.001518 | 0.053131 | 65535 | -0.02512 | 0.921735 | -0.5526 |
| O2-T3 | -2.91566 | 0.002124 | 0.055899 | 65535 | -0.01508 | 0.99993 | -0.53232 |
| O2-T4 | 1.533329 | 0.936064 | 0.999861 | 65535 | 0.028768 | 0.275211 | 0.279947 |
| O2-T5 | 1.635392 | 0.947684 | 0.999861 | 65535 | 0.037736 | 0.349891 | 0.29858 |
| O2-T6 | 1.442386 | 0.924079 | 0.999861 | 65535 | 0.037192 | 0.277092 | 0.263342 |
| O2-Fz | 1.104634 | 0.864217 | 0.999861 | 65535 | 0.011699 | 0.157264 | 0.201677 |
| O2-Cz | -2.57636 | 0.005609 | 0.098157 | 65535 | -0.01658 | 1 | -0.47038 |
| O2-Pz | 0.854698 | 0.802775 | 0.999861 | 65535 | 0.029513 | 0.106236 | 0.156046 |
| O2-F9 | -0.45096 | 0.326424 | 0.999861 | 65535 | 0.01038 | 0.07155 | -0.08233 |
| O2-F10 | 1.639653 | 0.94813 | 0.999861 | 65535 | 0.03483 | 0.376526 | 0.299358 |
| F7-F8 | 2.120258 | 0.981961 | 0.999861 | 65535 | 0.175919 | 0.47382 | 0.387104 |
| F7-T3 | -0.85017 | 0.198477 | 0.985537 | 65535 | 0.036498 | 0.164614 | -0.15522 |
| F7-T4 | 0.128095 | 0.550854 | 0.999861 | 65535 | 0.042832 | 0.051818 | 0.023387 |
| F7-T5 | -3.03846 | 0.001464 | 0.053131 | 65535 | -0.02774 | 0.888374 | -0.55474 |
| F7-T6 | -1.8159 | 0.035962 | 0.355329 | 65535 | -0.00281 | 0.879405 | -0.33154 |
| F7-Fz | -0.54727 | 0.292612 | 0.999861 | 65535 | 0.009861 | 0.088965 | -0.09992 |
| F7-Cz | 0.378297 | 0.647055 | 0.999861 | 65535 | 0.040832 | 0.066597 | 0.069067 |
| F7-Pz | 0.730002 | 0.766582 | 0.999861 | 65535 | 0.022788 | 0.09957 | 0.133279 |
| F7-F9 | -0.42802 | 0.334707 | 0.999861 | 65535 | 0.03958 | 0.074116 | -0.07815 |
| F7-F10 | 0.568251 | 0.714528 | 0.999861 | 65535 | 0.06342 | 0.090754 | 0.103749 |
| F8-T3 | 0.578808 | 0.718089 | 0.999861 | 65535 | 0.058934 | 0.084416 | 0.105676 |
| F8-T4 | -0.34266 | 0.366233 | 0.999861 | 65535 | 0.040997 | 0.061935 | -0.06256 |
| F8-T5 | 0.630043 | 0.735058 | 0.999861 | 65535 | 0.029005 | 0.085834 | 0.11503 |
| F8-T6 | -3.03826 | 0.001465 | 0.053131 | 65535 | -0.02373 | 0.94404 | -0.55471 |
| F8-Fz | 0.906835 | 0.816829 | 0.999861 | 65535 | 0.022121 | 0.149258 | 0.165565 |
| F8-Cz | 1.476524 | 0.928766 | 0.999861 | 65535 | 0.063128 | 0.264837 | 0.269575 |
| F8-Pz | 1.225266 | 0.888542 | 0.999861 | 65535 | 0.023953 | 0.243989 | 0.223702 |
| F8-F9 | -1.26369 | 0.104416 | 0.707336 | 65535 | 0.011117 | 0.237572 | -0.23072 |
| F8-F10 | 2.133959 | 0.982542 | 0.999861 | 65535 | 0.099172 | 0.451743 | 0.389606 |
| T3-T4 | 2.162991 | 0.983721 | 0.999861 | 65535 | 0.064849 | 0.490453 | 0.394906 |
| T3-T5 | -0.56108 | 0.287905 | 0.999861 | 65535 | 0.028993 | 0.092848 | -0.10244 |
| T3-T6 | 0.793458 | 0.785448 | 0.999861 | 65535 | 0.033715 | 0.130418 | 0.144865 |
| T3-Fz | -0.39328 | 0.34741 | 0.999861 | 65535 | 0.00712 | 0.069836 | -0.0718 |
| T3-Cz | -0.66315 | 0.254265 | 0.999861 | 65535 | 0.023276 | 0.14747 | -0.12107 |
| T3-Pz | -1.67018 | 0.048767 | 0.440814 | 65535 | -0.00012 | 0.979202 | -0.30493 |
| T3-F9 | 1.09416 | 0.861943 | 0.999861 | 65535 | 0.077121 | 0.200181 | 0.199765 |
| T3-F10 | 1.930344 | 0.972018 | 0.999861 | 65535 | 0.118519 | 0.497552 | 0.352431 |
| T4-T5 | -0.95385 | 0.171054 | 0.9727 | 65535 | 0.009735 | 0.19077 | -0.17415 |
| T4-T6 | 0.60082 | 0.725444 | 0.999861 | 65535 | 0.054747 | 0.088798 | 0.109694 |
| T4-Fz | 1.915464 | 0.971073 | 0.999861 | 65535 | 0.015535 | 0.324587 | 0.349714 |
| T4-Cz | 1.502266 | 0.93215 | 0.999861 | 65535 | 0.013533 | 0.257251 | 0.274275 |
| T4-Pz | 1.07584 | 0.857903 | 0.999861 | 65535 | 0.018407 | 0.177818 | 0.19642 |
| T4-F9 | -0.36349 | 0.358445 | 0.999861 | 65535 | 0.038059 | 0.064604 | -0.06636 |
| T4-F10 | 0.008926 | 0.503553 | 0.999861 | 65535 | 0.039097 | 0.050008 | 0.001629 |
| T5-T6 | -0.84024 | 0.201236 | 0.985537 | 65535 | 0.006667 | 0.12999 | -0.15341 |
| T5-Fz | 1.230289 | 0.889482 | 0.999861 | 65535 | 0.012789 | 0.166178 | 0.224619 |
| T5-Cz | -0.65643 | 0.256413 | 0.999861 | 65535 | 0.008563 | 0.185422 | -0.11985 |
| T5-Pz | -0.20274 | 0.419844 | 0.999861 | 65535 | 0.017005 | 0.06057 | -0.03702 |
| T5-F9 | 0.257361 | 0.601326 | 0.999861 | 65535 | 0.044831 | 0.057337 | 0.046987 |
| T5-F10 | -0.76866 | 0.221815 | 0.999861 | 65535 | 0.02036 | 0.113769 | -0.14034 |
| T6-Fz | 1.155567 | 0.874904 | 0.999861 | 65535 | 0.011858 | 0.202733 | 0.210977 |
| T6-Cz | -1.19605 | 0.117037 | 0.722877 | 65535 | 0.006877 | 0.690058 | -0.21837 |
| T6-Pz | -0.6885 | 0.246245 | 0.999861 | 65535 | 0.010672 | 0.189188 | -0.1257 |
| T6-F9 | -0.83822 | 0.201801 | 0.985537 | 65535 | 0.017518 | 0.131548 | -0.15304 |
| T6-F10 | 3.637015 | 0.999795 | 0.999861 | 65535 | 0.103533 | 0.911021 | 0.664025 |
| Fz-Cz | 0.301062 | 0.618051 | 0.999861 | 65535 | 0.034992 | 0.059787 | 0.054966 |
| Fz-Pz | -0.24006 | 0.40535 | 0.999861 | 65535 | 0.00933 | 0.057663 | -0.04383 |
| Fz-F9 | 1.770951 | 0.960425 | 0.999861 | 65535 | 0.013923 | 0.38631 | 0.32333 |
| Fz-F10 | 0.33658 | 0.631485 | 0.999861 | 65535 | 0.009451 | 0.062356 | 0.061451 |
| Cz-Pz | 2.129792 | 0.982367 | 0.999861 | 65535 | 0.074752 | 0.418745 | 0.388845 |
| Cz-F9 | -0.38321 | 0.351126 | 0.999861 | 65535 | 0.012138 | 0.110514 | -0.06996 |
| Cz-F10 | 0.680945 | 0.75138 | 0.999861 | 65535 | 0.019334 | 0.130713 | 0.124323 |
| Pz-F9 | 1.244405 | 0.892092 | 0.999861 | 65535 | 0.012111 | 0.22392 | 0.227196 |
| Pz-F10 | 1.355253 | 0.911037 | 0.999861 | 65535 | 0.011714 | 0.256011 | 0.247434 |
| F9-F10 | 0.193921 | 0.576714 | 0.999861 | 65535 | 0.053464 | 0.053485 | 0.035405 |

Table S3e: delta_coh_HC_PT

| channel | tvalue | pvalue | fdr_pvalue | CI_low | CI_high | statistical power | effect size |
| --- | --- | --- | --- | --- | --- | --- | --- |
| Fp1-Fp2 | 1.243939 | 0.107994 | 0.999617 | -0.02087 | 65535 | 0.229996 | 0.227111 |
| Fp1-F3 | -0.2436 | 0.59602 | 0.999617 | -0.06336 | 65535 | 0.056626 | -0.04448 |
| Fp1-F4 | 1.709323 | 0.04501 | 0.945207 | 0.001123 | 65535 | 0.458641 | 0.312078 |
| Fp1-C3 | 0.456507 | 0.324432 | 0.999617 | -0.02463 | 65535 | 0.074601 | 0.083346 |
| Fp1-C4 | 2.403565 | 0.008897 | 0.622759 | 0.013028 | 65535 | 0.476502 | 0.438829 |
| Fp1-P3 | 0.505683 | 0.307011 | 0.999617 | -0.01392 | 65535 | 0.078405 | 0.092325 |
| Fp1-P4 | -0.68614 | 0.753015 | 0.999617 | -0.02165 | 65535 | 0.11833 | -0.12527 |
| Fp1-O1 | -0.83687 | 0.797821 | 0.999617 | -0.05677 | 65535 | 0.149244 | -0.15279 |
| Fp1-O2 | -1.53298 | 0.936021 | 0.999617 | -0.06586 | 65535 | 0.503256 | -0.27988 |
| Fp1-F7 | 0.997531 | 0.160274 | 0.999617 | -0.03215 | 65535 | 0.159733 | 0.182123 |
| Fp1-F8 | 1.175739 | 0.121033 | 0.999617 | -0.01869 | 65535 | 0.180764 | 0.214659 |
| Fp1-T3 | -0.14779 | 0.558618 | 0.999617 | -0.0741 | 65535 | 0.052331 | -0.02698 |
| Fp1-T4 | 0.161484 | 0.435994 | 0.999617 | -0.03614 | 65535 | 0.052667 | 0.029483 |
| Fp1-T5 | -0.29281 | 0.614907 | 0.999617 | -0.06225 | 65535 | 0.059266 | -0.05346 |
| Fp1-T6 | -1.00039 | 0.840416 | 0.999617 | -0.0515 | 65535 | 0.234882 | -0.18265 |
| Fp1-Fz | 1.063037 | 0.144968 | 0.999617 | -0.00951 | 65535 | 0.164998 | 0.194083 |
| Fp1-Cz | 0.482196 | 0.31528 | 0.999617 | -0.03631 | 65535 | 0.07934 | 0.088037 |
| Fp1-Pz | 0.151978 | 0.439732 | 0.999617 | -0.01309 | 65535 | 0.051991 | 0.027747 |
| Fp1-F9 | -0.46178 | 0.677456 | 0.999617 | -0.05236 | 65535 | 0.077345 | -0.08431 |
| Fp1-F10 | 0.451647 | 0.326176 | 0.999617 | -0.03531 | 65535 | 0.070225 | 0.082459 |
| Fp2-F3 | 0.923994 | 0.178688 | 0.999617 | -0.01661 | 65535 | 0.11778 | 0.168697 |
| Fp2-F4 | 0.298403 | 0.38296 | 0.999617 | -0.04545 | 65535 | 0.059423 | 0.054481 |
| Fp2-C3 | -0.49037 | 0.687607 | 0.999617 | -0.04304 | 65535 | 0.085903 | -0.08953 |
| Fp2-C4 | 0.249678 | 0.401635 | 0.999617 | -0.02686 | 65535 | 0.05674 | 0.045584 |
| Fp2-P3 | 0.424221 | 0.336088 | 0.999617 | -0.01356 | 65535 | 0.070057 | 0.077452 |
| Fp2-P4 | 0.801716 | 0.212164 | 0.999617 | -0.00823 | 65535 | 0.110395 | 0.146372 |
| Fp2-O1 | -1.25708 | 0.894397 | 0.999617 | -0.06968 | 65535 | 0.302795 | -0.22951 |
| Fp2-O2 | -2.60815 | 0.99486 | 0.999617 | -0.10698 | 65535 | 0.944607 | -0.47618 |
| Fp2-F7 | 0.664667 | 0.25378 | 0.999617 | -0.04087 | 65535 | 0.093007 | 0.121351 |
| Fp2-F8 | -1.14956 | 0.873675 | 0.999617 | -0.13136 | 65535 | 0.197927 | -0.20988 |
| Fp2-T3 | 0.278788 | 0.390448 | 0.999617 | -0.04225 | 65535 | 0.059071 | 0.050899 |
| Fp2-T4 | 0.105195 | 0.4582 | 0.999617 | -0.05335 | 65535 | 0.051072 | 0.019206 |
| Fp2-T5 | 0.392532 | 0.347687 | 0.999617 | -0.02647 | 65535 | 0.063904 | 0.071666 |
| Fp2-T6 | -0.8336 | 0.796905 | 0.999617 | -0.07084 | 65535 | 0.130885 | -0.15219 |
| Fp2-Fz | 0.494888 | 0.3108 | 0.999617 | -0.02471 | 65535 | 0.071807 | 0.090354 |
| Fp2-Cz | -0.6277 | 0.734294 | 0.999617 | -0.07344 | 65535 | 0.104383 | -0.1146 |
| Fp2-Pz | 0.44044 | 0.330212 | 0.999617 | -0.01405 | 65535 | 0.068072 | 0.080413 |
| Fp2-F9 | -0.05518 | 0.521955 | 0.999617 | -0.05239 | 65535 | 0.050276 | -0.01007 |
| Fp2-F10 | 1.461491 | 0.073269 | 0.999617 | -0.00514 | 65535 | 0.248324 | 0.266831 |
| F3-F4 | 1.924845 | 0.028328 | 0.849839 | 0.006163 | 65535 | 0.297336 | 0.351427 |
| F3-C3 | -0.66994 | 0.747897 | 0.999617 | -0.05113 | 65535 | 0.115675 | -0.12231 |
| F3-C4 | 1.584739 | 0.057851 | 0.958237 | -0.00088 | 65535 | 0.238592 | 0.289332 |
| F3-P3 | 0.213305 | 0.415729 | 0.999617 | -0.01113 | 65535 | 0.053331 | 0.038944 |
| F3-P4 | 1.033768 | 0.151679 | 0.999617 | -0.0034 | 65535 | 0.144585 | 0.188739 |
| F3-O1 | 0.817817 | 0.207555 | 0.999617 | -0.00944 | 65535 | 0.102778 | 0.149312 |
| F3-O2 | 0.437578 | 0.331246 | 0.999617 | -0.01374 | 65535 | 0.065096 | 0.07989 |
| F3-F7 | -0.07389 | 0.52939 | 0.999617 | -0.06686 | 65535 | 0.050695 | -0.01349 |
| F3-F8 | 1.403244 | 0.081585 | 0.999617 | -0.00625 | 65535 | 0.212784 | 0.256196 |
| F3-T3 | -2.51153 | 0.993314 | 0.999617 | -0.08106 | 65535 | 0.911199 | -0.45854 |
| F3-T4 | -1.43876 | 0.923567 | 0.999617 | -0.05989 | 65535 | 0.244941 | -0.26268 |
| F3-T5 | -2.83484 | 0.997302 | 0.999617 | -0.07912 | 65535 | 0.877531 | -0.51757 |
| F3-T6 | -1.0514 | 0.852389 | 0.999617 | -0.03744 | 65535 | 0.143084 | -0.19196 |
| F3-Fz | 1.803134 | 0.03696 | 0.945207 | 0.003085 | 65535 | 0.286359 | 0.329206 |
| F3-Cz | -0.3411 | 0.633184 | 0.999617 | -0.03775 | 65535 | 0.07022 | -0.06228 |
| F3-Pz | -1.44671 | 0.924686 | 0.999617 | -0.02112 | 65535 | 0.759158 | -0.26413 |
| F3-F9 | -2.21818 | 0.985772 | 0.999617 | -0.09722 | 65535 | 0.562723 | -0.40498 |
| F3-F10 | -3.4547 | 0.999617 | 0.999617 | -0.1039 | 65535 | 0.999103 | -0.63074 |
| F4-C3 | 0.273106 | 0.392624 | 0.999617 | -0.01749 | 65535 | 0.056021 | 0.049862 |
| F4-C4 | 0.500231 | 0.308922 | 0.999617 | -0.02348 | 65535 | 0.073363 | 0.09133 |
| F4-P3 | 0.483391 | 0.314857 | 0.999617 | -0.00616 | 65535 | 0.079234 | 0.088255 |
| F4-P4 | 0.793139 | 0.214644 | 0.999617 | -0.00382 | 65535 | 0.107605 | 0.144807 |
| F4-O1 | 1.018446 | 0.155275 | 0.999617 | -0.00883 | 65535 | 0.132843 | 0.185942 |
| F4-O2 | -0.25729 | 0.601297 | 0.999617 | -0.02573 | 65535 | 0.056654 | -0.04697 |
| F4-F7 | 0.699462 | 0.24282 | 0.999617 | -0.02439 | 65535 | 0.092431 | 0.127704 |
| F4-F8 | 0.686727 | 0.246801 | 0.999617 | -0.03565 | 65535 | 0.10577 | 0.125379 |
| F4-T3 | -0.02526 | 0.510055 | 0.999617 | -0.02947 | 65535 | 0.050062 | -0.00461 |
| F4-T4 | 0.045821 | 0.481765 | 0.999617 | -0.0337 | 65535 | 0.050185 | 0.008366 |
| F4-T5 | -0.61404 | 0.729816 | 0.999617 | -0.02108 | 65535 | 0.088061 | -0.11211 |
| F4-T6 | -0.26955 | 0.60601 | 0.999617 | -0.03245 | 65535 | 0.056535 | -0.04921 |
| F4-Fz | 1.96673 | 0.025781 | 0.849839 | 0.007942 | 65535 | 0.380762 | 0.359074 |
| F4-Cz | 2.513529 | 0.006651 | 0.622759 | 0.015054 | 65535 | 0.465803 | 0.458905 |
| F4-Pz | 0.150113 | 0.440466 | 0.999617 | -0.00985 | 65535 | 0.053993 | 0.027407 |
| F4-F9 | -1.17177 | 0.878175 | 0.999617 | -0.06128 | 65535 | 0.213399 | -0.21393 |
| F4-F10 | -1.8452 | 0.966242 | 0.999617 | -0.06013 | 65535 | 0.432874 | -0.33689 |
| C3-C4 | 1.521474 | 0.065408 | 0.981121 | -0.00166 | 65535 | 0.208419 | 0.277782 |
| C3-P3 | 0.224278 | 0.411464 | 0.999617 | -0.02421 | 65535 | 0.054565 | 0.040948 |
| C3-P4 | -1.77515 | 0.960774 | 0.999617 | -0.02703 | 65535 | 0.66477 | -0.3241 |
| C3-O1 | -2.06483 | 0.979435 | 0.999617 | -0.0487 | 65535 | 0.783184 | -0.37699 |
| C3-O2 | -2.11404 | 0.981691 | 0.999617 | -0.04057 | 65535 | 0.821407 | -0.38597 |
| C3-F7 | 0.991956 | 0.161624 | 0.999617 | -0.0142 | 65535 | 0.177494 | 0.181106 |
| C3-F8 | -0.0695 | 0.527647 | 0.999617 | -0.02932 | 65535 | 0.050413 | -0.01269 |
| C3-T3 | -0.38473 | 0.649437 | 0.999617 | -0.04359 | 65535 | 0.065402 | -0.07024 |
| C3-T4 | 0.625083 | 0.266562 | 0.999617 | -0.01441 | 65535 | 0.079645 | 0.114124 |
| C3-T5 | 0.410585 | 0.341061 | 0.999617 | -0.01369 | 65535 | 0.067996 | 0.074962 |
| C3-T6 | -1.29764 | 0.901529 | 0.999617 | -0.03629 | 65535 | 0.267856 | -0.23692 |
| C3-Fz | 0.302378 | 0.381448 | 0.999617 | -0.015 | 65535 | 0.058956 | 0.055206 |
| C3-Cz | -1.95419 | 0.973478 | 0.999617 | -0.0654 | 65535 | 0.891786 | -0.35679 |
| C3-Pz | -2.60578 | 0.994826 | 0.999617 | -0.03326 | 65535 | 0.86142 | -0.47575 |
| C3-F9 | 0.731384 | 0.232997 | 0.999617 | -0.01818 | 65535 | 0.089763 | 0.133532 |
| C3-F10 | -0.12657 | 0.550251 | 0.999617 | -0.02962 | 65535 | 0.051857 | -0.02311 |
| C4-P3 | 0.177381 | 0.429757 | 0.999617 | -0.01176 | 65535 | 0.054568 | 0.032385 |
| C4-P4 | 0.159377 | 0.436822 | 0.999617 | -0.01928 | 65535 | 0.052174 | 0.029098 |
| C4-O1 | 0.767108 | 0.222275 | 0.999617 | -0.00673 | 65535 | 0.099731 | 0.140054 |
| C4-O2 | -0.28114 | 0.610452 | 0.999617 | -0.01602 | 65535 | 0.0573 | -0.05133 |
| C4-F7 | 0.282436 | 0.389052 | 0.999617 | -0.01898 | 65535 | 0.056744 | 0.051566 |
| C4-F8 | -2.0327 | 0.977837 | 0.999617 | -0.08284 | 65535 | 0.564414 | -0.37112 |
| C4-T3 | 1.595274 | 0.056663 | 0.958237 | -0.00079 | 65535 | 0.248294 | 0.291256 |
| C4-T4 | -1.94966 | 0.973206 | 0.999617 | -0.06942 | 65535 | 0.492655 | -0.35596 |
| C4-T5 | 1.716051 | 0.044388 | 0.945207 | 0.000563 | 65535 | 0.279033 | 0.313307 |
| C4-T6 | -0.20482 | 0.580969 | 0.999617 | -0.02336 | 65535 | 0.054238 | -0.0374 |
| C4-Fz | 2.128274 | 0.017697 | 0.849839 | 0.005792 | 65535 | 0.39268 | 0.388568 |
| C4-Cz | 2.662401 | 0.00442 | 0.622759 | 0.021135 | 65535 | 0.505733 | 0.486086 |
| C4-Pz | 0.113625 | 0.454864 | 0.999617 | -0.019 | 65535 | 0.051137 | 0.020745 |
| C4-F9 | -1.71072 | 0.95512 | 0.999617 | -0.0601 | 65535 | 0.403807 | -0.31233 |
| C4-F10 | 0.270345 | 0.393683 | 0.999617 | -0.0104 | 65535 | 0.055792 | 0.049358 |
| P3-P4 | 1.571953 | 0.059319 | 0.958237 | -0.00068 | 65535 | 0.249729 | 0.286998 |
| P3-O1 | -0.84601 | 0.800371 | 0.999617 | -0.04061 | 65535 | 0.183139 | -0.15446 |
| P3-O2 | -0.54732 | 0.707405 | 0.999617 | -0.01542 | 65535 | 0.091912 | -0.09993 |
| P3-F7 | 0.035788 | 0.485756 | 0.999617 | -0.01743 | 65535 | 0.050156 | 0.006534 |
| P3-F8 | -0.04725 | 0.518802 | 0.999617 | -0.01188 | 65535 | 0.050258 | -0.00863 |
| P3-T3 | -1.62057 | 0.946111 | 0.999617 | -0.05258 | 65535 | 0.502008 | -0.29587 |
| P3-T4 | -0.89451 | 0.813565 | 0.999617 | -0.01607 | 65535 | 0.234946 | -0.16331 |
| P3-T5 | -0.87395 | 0.808041 | 0.999617 | -0.02484 | 65535 | 0.165836 | -0.15956 |
| P3-T6 | 1.360908 | 0.088068 | 0.999617 | -0.00301 | 65535 | 0.285835 | 0.248467 |
| P3-Fz | -1.21893 | 0.887349 | 0.999617 | -0.02429 | 65535 | 0.309894 | -0.22255 |
| P3-Cz | -0.72547 | 0.765201 | 0.999617 | -0.02278 | 65535 | 0.149731 | -0.13245 |
| P3-Pz | 0.134736 | 0.446525 | 0.999617 | -0.02447 | 65535 | 0.052192 | 0.024599 |
| P3-F9 | -0.05878 | 0.523386 | 0.999617 | -0.01379 | 65535 | 0.050494 | -0.01073 |
| P3-F10 | 0.639468 | 0.261879 | 0.999617 | -0.00803 | 65535 | 0.08659 | 0.11675 |
| P4-O1 | -1.49316 | 0.930968 | 0.999617 | -0.02748 | 65535 | 0.749213 | -0.27261 |
| P4-O2 | -1.4623 | 0.926841 | 0.999617 | -0.03395 | 65535 | 0.823084 | -0.26698 |
| P4-F7 | -1.03039 | 0.847532 | 0.999617 | -0.01529 | 65535 | 0.15886 | -0.18812 |
| P4-F8 | -0.22302 | 0.588049 | 0.999617 | -0.01603 | 65535 | 0.056156 | -0.04072 |
| P4-T3 | -1.13928 | 0.871552 | 0.999617 | -0.03122 | 65535 | 0.274334 | -0.208 |
| P4-T4 | -0.14338 | 0.556883 | 0.999617 | -0.01757 | 65535 | 0.052209 | -0.02618 |
| P4-T5 | 0.248818 | 0.401967 | 0.999617 | -0.01334 | 65535 | 0.059293 | 0.045428 |
| P4-T6 | 0.148173 | 0.44123 | 0.999617 | -0.02053 | 65535 | 0.052473 | 0.027053 |
| P4-Fz | -0.44768 | 0.672396 | 0.999617 | -0.01567 | 65535 | 0.076721 | -0.08173 |
| P4-Cz | -0.4271 | 0.664959 | 0.999617 | -0.02844 | 65535 | 0.064937 | -0.07798 |
| P4-Pz | -0.46375 | 0.67816 | 0.999617 | -0.03677 | 65535 | 0.086286 | -0.08467 |
| P4-F9 | -0.39393 | 0.652829 | 0.999617 | -0.01207 | 65535 | 0.071465 | -0.07192 |
| P4-F10 | -0.31717 | 0.624161 | 0.999617 | -0.0142 | 65535 | 0.059405 | -0.05791 |
| O1-O2 | -2.10258 | 0.981186 | 0.999617 | -0.08927 | 65535 | 0.660939 | -0.38388 |
| O1-F7 | -0.85321 | 0.802363 | 0.999617 | -0.0505 | 65535 | 0.161446 | -0.15577 |
| O1-F8 | -0.88516 | 0.811065 | 0.999617 | -0.03851 | 65535 | 0.145191 | -0.16161 |
| O1-T3 | -2.49559 | 0.993022 | 0.999617 | -0.102 | 65535 | 0.974941 | -0.45563 |
| O1-T4 | -0.60745 | 0.727639 | 0.999617 | -0.03238 | 65535 | 0.104209 | -0.1109 |
| O1-T5 | 0.230626 | 0.409002 | 0.999617 | -0.0225 | 65535 | 0.058848 | 0.042106 |
| O1-T6 | -2.07845 | 0.980082 | 0.999617 | -0.0552 | 65535 | 0.653229 | -0.37947 |
| O1-Fz | 0.672798 | 0.251196 | 0.999617 | -0.00778 | 65535 | 0.090321 | 0.122835 |
| O1-Cz | -3.11006 | 0.998827 | 0.999617 | -0.09689 | 65535 | 0.99965 | -0.56782 |
| O1-Pz | -0.58432 | 0.71994 | 0.999617 | -0.0218 | 65535 | 0.086762 | -0.10668 |
| O1-F9 | 0.702754 | 0.241796 | 0.999617 | -0.00985 | 65535 | 0.089112 | 0.128305 |
| O1-F10 | 0.202651 | 0.419878 | 0.999617 | -0.01486 | 65535 | 0.053848 | 0.036999 |
| O2-F7 | -0.95955 | 0.830379 | 0.999617 | -0.04526 | 65535 | 0.171847 | -0.17519 |
| O2-F8 | -2.09861 | 0.981008 | 0.999617 | -0.07831 | 65535 | 0.588111 | -0.38315 |
| O2-T3 | -2.35068 | 0.989801 | 0.999617 | -0.05839 | 65535 | 0.848453 | -0.42917 |
| O2-T4 | 0.83701 | 0.202139 | 0.999617 | -0.01164 | 65535 | 0.11118 | 0.152816 |
| O2-T5 | -2.45622 | 0.992252 | 0.999617 | -0.03441 | 65535 | 0.965934 | -0.44844 |
| O2-T6 | -1.24867 | 0.892871 | 0.999617 | -0.04085 | 65535 | 0.26474 | -0.22797 |
| O2-Fz | -0.10931 | 0.543429 | 0.999617 | -0.01209 | 65535 | 0.051168 | -0.01996 |
| O2-Cz | -2.69217 | 0.995934 | 0.999617 | -0.08619 | 65535 | 0.998941 | -0.49152 |
| O2-Pz | -0.16354 | 0.564815 | 0.999617 | -0.01893 | 65535 | 0.052101 | -0.02986 |
| O2-F9 | -0.45551 | 0.67521 | 0.999617 | -0.03035 | 65535 | 0.068309 | -0.08316 |
| O2-F10 | -0.79782 | 0.786712 | 0.999617 | -0.02128 | 65535 | 0.1744 | -0.14566 |
| F7-F8 | 1.027144 | 0.153227 | 0.999617 | -0.03221 | 65535 | 0.158536 | 0.18753 |
| F7-T3 | -1.36092 | 0.911933 | 0.999617 | -0.13756 | 65535 | 0.298678 | -0.24847 |
| F7-T4 | -1.61398 | 0.945399 | 0.999617 | -0.1075 | 65535 | 0.349009 | -0.29467 |
| F7-T5 | -0.60043 | 0.725315 | 0.999617 | -0.06809 | 65535 | 0.089045 | -0.10962 |
| F7-T6 | -1.84139 | 0.965962 | 0.999617 | -0.09985 | 65535 | 0.516843 | -0.33619 |
| F7-Fz | 0.101974 | 0.459475 | 0.999617 | -0.01935 | 65535 | 0.050893 | 0.018618 |
| F7-Cz | 0.946905 | 0.172811 | 0.999617 | -0.01478 | 65535 | 0.129062 | 0.17288 |
| F7-Pz | -1.29258 | 0.90066 | 0.999617 | -0.01731 | 65535 | 0.302788 | -0.23599 |
| F7-F9 | 0.139911 | 0.444485 | 0.999617 | -0.05578 | 65535 | 0.05205 | 0.025544 |
| F7-F10 | 0.892007 | 0.187102 | 0.999617 | -0.0265 | 65535 | 0.15217 | 0.162858 |
| F8-T3 | 0.480356 | 0.315932 | 0.999617 | -0.03479 | 65535 | 0.07003 | 0.087701 |
| F8-T4 | -2.05442 | 0.978928 | 0.999617 | -0.1462 | 65535 | 0.468197 | -0.37508 |
| F8-T5 | -0.82243 | 0.793754 | 0.999617 | -0.05282 | 65535 | 0.119183 | -0.15015 |
| F8-T6 | -1.85733 | 0.967121 | 0.999617 | -0.09997 | 65535 | 0.473091 | -0.3391 |
| F8-Fz | -0.14218 | 0.556411 | 0.999617 | -0.02175 | 65535 | 0.051795 | -0.02596 |
| F8-Cz | 0.778343 | 0.218962 | 0.999617 | -0.01656 | 65535 | 0.108522 | 0.142106 |
| F8-Pz | -0.878 | 0.809135 | 0.999617 | -0.01509 | 65535 | 0.194524 | -0.1603 |
| F8-F9 | -2.42379 | 0.991561 | 0.999617 | -0.15271 | 65535 | 0.624369 | -0.44252 |
| F8-F10 | 2.064091 | 0.020601 | 0.849839 | 0.011622 | 65535 | 0.409946 | 0.37685 |
| T3-T4 | 0.347906 | 0.364265 | 0.999617 | -0.03731 | 65535 | 0.060968 | 0.063519 |
| T3-T5 | -0.65151 | 0.742008 | 0.999617 | -0.07986 | 65535 | 0.094328 | -0.11895 |
| T3-T6 | -0.85499 | 0.802854 | 0.999617 | -0.05741 | 65535 | 0.143552 | -0.1561 |
| T3-Fz | -1.52325 | 0.934815 | 0.999617 | -0.02567 | 65535 | 0.557041 | -0.27811 |
| T3-Cz | -0.81854 | 0.792652 | 0.999617 | -0.05864 | 65535 | 0.182881 | -0.14944 |
| T3-Pz | -1.92668 | 0.971788 | 0.999617 | -0.03624 | 65535 | 0.999999 | -0.35176 |
| T3-F9 | 0.918828 | 0.18003 | 0.999617 | -0.02636 | 65535 | 0.147894 | 0.167755 |
| T3-F10 | 0.620234 | 0.26815 | 0.999617 | -0.04095 | 65535 | 0.105697 | 0.113239 |
| T4-T5 | -2.12156 | 0.982017 | 0.999617 | -0.08889 | 65535 | 0.676844 | -0.38734 |
| T4-T6 | -0.49168 | 0.688069 | 0.999617 | -0.06769 | 65535 | 0.079332 | -0.08977 |
| T4-Fz | 0.677247 | 0.249787 | 0.999617 | -0.00979 | 65535 | 0.081704 | 0.123648 |
| T4-Cz | -0.20751 | 0.582014 | 0.999617 | -0.02093 | 65535 | 0.057737 | -0.03789 |
| T4-Pz | -1.18449 | 0.8807 | 0.999617 | -0.01466 | 65535 | 0.375283 | -0.21626 |
| T4-F9 | -3.28151 | 0.999321 | 0.999617 | -0.17389 | 65535 | 0.860022 | -0.59912 |
| T4-F10 | -0.30782 | 0.620619 | 0.999617 | -0.05309 | 65535 | 0.060829 | -0.0562 |
| T5-T6 | -0.91464 | 0.818878 | 0.999617 | -0.03451 | 65535 | 0.199817 | -0.16699 |
| T5-Fz | 0.194841 | 0.422926 | 0.999617 | -0.0079 | 65535 | 0.053656 | 0.035573 |
| T5-Cz | 0.082858 | 0.467053 | 0.999617 | -0.01678 | 65535 | 0.050621 | 0.015127 |
| T5-Pz | -1.83262 | 0.96531 | 0.999617 | -0.04631 | 65535 | 0.937278 | -0.33459 |
| T5-F9 | -1.65881 | 0.950095 | 0.999617 | -0.09711 | 65535 | 0.445917 | -0.30285 |
| T5-F10 | -0.70186 | 0.757927 | 0.999617 | -0.07339 | 65535 | 0.103587 | -0.12814 |
| T6-Fz | 0.102746 | 0.45917 | 0.999617 | -0.01383 | 65535 | 0.051104 | 0.018759 |
| T6-Cz | -1.66581 | 0.950798 | 0.999617 | -0.06156 | 65535 | 0.554224 | -0.30413 |
| T6-Pz | -1.94032 | 0.972636 | 0.999617 | -0.03199 | 65535 | 0.96317 | -0.35425 |
| T6-F9 | -2.30449 | 0.988528 | 0.999617 | -0.10878 | 65535 | 0.599141 | -0.42074 |
| T6-F10 | -0.99415 | 0.838909 | 0.999617 | -0.07078 | 65535 | 0.176063 | -0.18151 |
| Fz-Cz | -2.00596 | 0.976426 | 0.999617 | -0.06255 | 65535 | 0.880454 | -0.36624 |
| Fz-Pz | -1.49983 | 0.931835 | 0.999617 | -0.01845 | 65535 | 0.490383 | -0.27383 |
| Fz-F9 | -0.011 | 0.50438 | 0.999617 | -0.01811 | 65535 | 0.05001 | -0.00201 |
| Fz-F10 | -0.11499 | 0.545674 | 0.999617 | -0.01492 | 65535 | 0.051293 | -0.02099 |
| Cz-Pz | 1.090345 | 0.138891 | 0.999617 | -0.00868 | 65535 | 0.158725 | 0.199069 |
| Cz-F9 | -0.28037 | 0.610159 | 0.999617 | -0.0149 | 65535 | 0.057013 | -0.05119 |
| Cz-F10 | -0.7144 | 0.761806 | 0.999617 | -0.0118 | 65535 | 0.106119 | -0.13043 |
| Pz-F9 | -0.82444 | 0.794324 | 0.999617 | -0.01171 | 65535 | 0.273455 | -0.15052 |
| Pz-F10 | -0.57286 | 0.716084 | 0.999617 | -0.00801 | 65535 | 0.101991 | -0.10459 |
| F9-F10 | -0.36603 | 0.642501 | 0.999617 | -0.06883 | 65535 | 0.064271 | -0.06683 |

Table S3f: delta_coh_PT_HC

| channel | tvalue | pvalue | fdr_pvalue | CI_low | CI_high | statistical power | effect size |
| --- | --- | --- | --- | --- | --- | --- | --- |
| Fp1-Fp2 | 1.243939 | 0.892006 | 0.970577 | 65535 | 0.146294 | 0.229996 | 0.227111 |
| Fp1-F3 | -0.2436 | 0.40398 | 0.807961 | 65535 | 0.047123 | 0.056626 | -0.04448 |
| Fp1-F4 | 1.709323 | 0.95499 | 0.993458 | 65535 | 0.073514 | 0.458641 | 0.312078 |
| Fp1-C3 | 0.456507 | 0.675568 | 0.887364 | 65535 | 0.04334 | 0.074601 | 0.083346 |
| Fp1-C4 | 2.403565 | 0.991103 | 0.99558 | 65535 | 0.070958 | 0.476502 | 0.438829 |
| Fp1-P3 | 0.505683 | 0.692989 | 0.887364 | 65535 | 0.026141 | 0.078405 | 0.092325 |
| Fp1-P4 | -0.68614 | 0.246985 | 0.673597 | 65535 | 0.008977 | 0.11833 | -0.12527 |
| Fp1-O1 | -0.83687 | 0.202179 | 0.604766 | 65535 | 0.018684 | 0.149244 | -0.15279 |
| Fp1-O2 | -1.53298 | 0.063979 | 0.350999 | 65535 | 0.002578 | 0.503256 | -0.27988 |
| Fp1-F7 | 0.997531 | 0.839726 | 0.942578 | 65535 | 0.129298 | 0.159733 | 0.182123 |
| Fp1-F8 | 1.175739 | 0.878967 | 0.96137 | 65535 | 0.109841 | 0.180764 | 0.214659 |
| Fp1-T3 | -0.14779 | 0.441382 | 0.831729 | 65535 | 0.061971 | 0.052331 | -0.02698 |
| Fp1-T4 | 0.161484 | 0.564006 | 0.865972 | 65535 | 0.043938 | 0.052667 | 0.029483 |
| Fp1-T5 | -0.29281 | 0.385093 | 0.802613 | 65535 | 0.043565 | 0.059266 | -0.05346 |
| Fp1-T6 | -1.00039 | 0.159584 | 0.583262 | 65535 | 0.012738 | 0.234882 | -0.18265 |
| Fp1-Fz | 1.063037 | 0.855032 | 0.945036 | 65535 | 0.043481 | 0.164998 | 0.194083 |
| Fp1-Cz | 0.482196 | 0.68472 | 0.887364 | 65535 | 0.066103 | 0.07934 | 0.088037 |
| Fp1-Pz | 0.151978 | 0.560268 | 0.865972 | 65535 | 0.015726 | 0.051991 | 0.027747 |
| Fp1-F9 | -0.46178 | 0.322544 | 0.747792 | 65535 | 0.029548 | 0.077345 | -0.08431 |
| Fp1-F10 | 0.451647 | 0.673824 | 0.887364 | 65535 | 0.061758 | 0.070225 | 0.082459 |
| Fp2-F3 | 0.923994 | 0.821312 | 0.94249 | 65535 | 0.058426 | 0.11778 | 0.168697 |
| Fp2-F4 | 0.298403 | 0.61704 | 0.865972 | 65535 | 0.065397 | 0.059423 | 0.054481 |
| Fp2-C3 | -0.49037 | 0.312393 | 0.745482 | 65535 | 0.023391 | 0.085903 | -0.08953 |
| Fp2-C4 | 0.249678 | 0.598365 | 0.865972 | 65535 | 0.036381 | 0.05674 | 0.045584 |
| Fp2-P3 | 0.424221 | 0.663912 | 0.887364 | 65535 | 0.022881 | 0.070057 | 0.077452 |
| Fp2-P4 | 0.801716 | 0.787836 | 0.929469 | 65535 | 0.023649 | 0.110395 | 0.146372 |
| Fp2-O1 | -1.25708 | 0.105603 | 0.459126 | 65535 | 0.009581 | 0.302795 | -0.22951 |
| Fp2-O2 | -2.60815 | 0.00514 | 0.155206 | 65535 | -0.02383 | 0.944607 | -0.47618 |
| Fp2-F7 | 0.664667 | 0.74622 | 0.920363 | 65535 | 0.095573 | 0.093007 | 0.121351 |
| Fp2-F8 | -1.14956 | 0.126325 | 0.499518 | 65535 | 0.023783 | 0.197927 | -0.20988 |
| Fp2-T3 | 0.278788 | 0.609552 | 0.865972 | 65535 | 0.059335 | 0.059071 | 0.050899 |
| Fp2-T4 | 0.105195 | 0.5418 | 0.865972 | 65535 | 0.060577 | 0.051072 | 0.019206 |
| Fp2-T5 | 0.392532 | 0.652313 | 0.887364 | 65535 | 0.042897 | 0.063904 | 0.071666 |
| Fp2-T6 | -0.8336 | 0.203095 | 0.604766 | 65535 | 0.023437 | 0.130885 | -0.15219 |
| Fp2-Fz | 0.494888 | 0.6892 | 0.887364 | 65535 | 0.04574 | 0.071807 | 0.090354 |
| Fp2-Cz | -0.6277 | 0.265706 | 0.694986 | 65535 | 0.0331 | 0.104383 | -0.1146 |
| Fp2-Pz | 0.44044 | 0.669788 | 0.887364 | 65535 | 0.024211 | 0.068072 | 0.080413 |
| Fp2-F9 | -0.05518 | 0.478045 | 0.842097 | 65535 | 0.049016 | 0.050276 | -0.01007 |
| Fp2-F10 | 1.461491 | 0.926731 | 0.990504 | 65535 | 0.081713 | 0.248324 | 0.266831 |
| F3-F4 | 1.924845 | 0.971672 | 0.99558 | 65535 | 0.082712 | 0.297336 | 0.351427 |
| F3-C3 | -0.66994 | 0.252103 | 0.678738 | 65535 | 0.021698 | 0.115675 | -0.12231 |
| F3-C4 | 1.584739 | 0.942149 | 0.990504 | 65535 | 0.039182 | 0.238592 | 0.289332 |
| F3-P3 | 0.213305 | 0.584271 | 0.865972 | 65535 | 0.014419 | 0.053331 | 0.038944 |
| F3-P4 | 1.033768 | 0.848321 | 0.942578 | 65535 | 0.014671 | 0.144585 | 0.188739 |
| F3-O1 | 0.817817 | 0.792445 | 0.929684 | 65535 | 0.027822 | 0.102778 | 0.149312 |
| F3-O2 | 0.437578 | 0.668754 | 0.887364 | 65535 | 0.02359 | 0.065096 | 0.07989 |
| F3-F7 | -0.07389 | 0.47061 | 0.842097 | 65535 | 0.061152 | 0.050695 | -0.01349 |
| F3-F8 | 1.403244 | 0.918415 | 0.989062 | 65535 | 0.07511 | 0.212784 | 0.256196 |
| F3-T3 | -2.51153 | 0.006686 | 0.161103 | 65535 | -0.0166 | 0.911199 | -0.45854 |
| F3-T4 | -1.43876 | 0.076433 | 0.364794 | 65535 | 0.004238 | 0.244941 | -0.26268 |
| F3-T5 | -2.83484 | 0.002698 | 0.141636 | 65535 | -0.02073 | 0.877531 | -0.51757 |
| F3-T6 | -1.0514 | 0.147611 | 0.563607 | 65535 | 0.008381 | 0.143084 | -0.19196 |
| F3-Fz | 1.803134 | 0.96304 | 0.99558 | 65535 | 0.073493 | 0.286359 | 0.329206 |
| F3-Cz | -0.3411 | 0.366816 | 0.794138 | 65535 | 0.024868 | 0.07022 | -0.06228 |
| F3-Pz | -1.44671 | 0.075314 | 0.364794 | 65535 | 0.001436 | 0.759158 | -0.26413 |
| F3-F9 | -2.21818 | 0.014228 | 0.210718 | 65535 | -0.01405 | 0.562723 | -0.40498 |
| F3-F10 | -3.4547 | 0.000383 | 0.071307 | 65535 | -0.03652 | 0.999103 | -0.63074 |
| F4-C3 | 0.273106 | 0.607376 | 0.865972 | 65535 | 0.024385 | 0.056021 | 0.049862 |
| F4-C4 | 0.500231 | 0.691078 | 0.887364 | 65535 | 0.043766 | 0.073363 | 0.09133 |
| F4-P3 | 0.483391 | 0.685143 | 0.887364 | 65535 | 0.011231 | 0.079234 | 0.088255 |
| F4-P4 | 0.793139 | 0.785356 | 0.929469 | 65535 | 0.010829 | 0.107605 | 0.144807 |
| F4-O1 | 1.018446 | 0.844725 | 0.942578 | 65535 | 0.036961 | 0.132843 | 0.185942 |
| F4-O2 | -0.25729 | 0.398703 | 0.805074 | 65535 | 0.018819 | 0.056654 | -0.04697 |
| F4-F7 | 0.699462 | 0.75718 | 0.920363 | 65535 | 0.059999 | 0.092431 | 0.127704 |
| F4-F8 | 0.686727 | 0.753199 | 0.920363 | 65535 | 0.086078 | 0.10577 | 0.125379 |
| F4-T3 | -0.02526 | 0.489945 | 0.850318 | 65535 | 0.028588 | 0.050062 | -0.00461 |
| F4-T4 | 0.045821 | 0.518235 | 0.865972 | 65535 | 0.035614 | 0.050185 | 0.008366 |
| F4-T5 | -0.61404 | 0.270184 | 0.694986 | 65535 | 0.009685 | 0.088061 | -0.11211 |
| F4-T6 | -0.26955 | 0.39399 | 0.803281 | 65535 | 0.023371 | 0.056535 | -0.04921 |
| F4-Fz | 1.96673 | 0.974219 | 0.99558 | 65535 | 0.093207 | 0.380762 | 0.359074 |
| F4-Cz | 2.513529 | 0.993349 | 0.99558 | 65535 | 0.073389 | 0.465803 | 0.458905 |
| F4-Pz | 0.150113 | 0.559534 | 0.865972 | 65535 | 0.011812 | 0.053993 | 0.027407 |
| F4-F9 | -1.17177 | 0.121825 | 0.491987 | 65535 | 0.010527 | 0.213399 | -0.21393 |
| F4-F10 | -1.8452 | 0.033758 | 0.234996 | 65535 | -0.00322 | 0.432874 | -0.33689 |
| C3-C4 | 1.521474 | 0.934592 | 0.990504 | 65535 | 0.038672 | 0.208419 | 0.277782 |
| C3-P3 | 0.224278 | 0.588536 | 0.865972 | 65535 | 0.031786 | 0.054565 | 0.040948 |
| C3-P4 | -1.77515 | 0.039226 | 0.25742 | 65535 | -0.00092 | 0.66477 | -0.3241 |
| C3-O1 | -2.06483 | 0.020565 | 0.210718 | 65535 | -0.00532 | 0.783184 | -0.37699 |
| C3-O2 | -2.11404 | 0.018309 | 0.210718 | 65535 | -0.00491 | 0.821407 | -0.38597 |
| C3-F7 | 0.991956 | 0.838376 | 0.942578 | 65535 | 0.056498 | 0.177494 | 0.181106 |
| C3-F8 | -0.0695 | 0.472353 | 0.842097 | 65535 | 0.026959 | 0.050413 | -0.01269 |
| C3-T3 | -0.38473 | 0.350563 | 0.77493 | 65535 | 0.027169 | 0.065402 | -0.07024 |
| C3-T4 | 0.625083 | 0.733438 | 0.920363 | 65535 | 0.031858 | 0.079645 | 0.114124 |
| C3-T5 | 0.410585 | 0.658939 | 0.887364 | 65535 | 0.022702 | 0.067996 | 0.074962 |
| C3-T6 | -1.29764 | 0.098471 | 0.443861 | 65535 | 0.004423 | 0.267856 | -0.23692 |
| C3-Fz | 0.302378 | 0.618552 | 0.865972 | 65535 | 0.021686 | 0.058956 | 0.055206 |
| C3-Cz | -1.95419 | 0.026522 | 0.219429 | 65535 | -0.00537 | 0.891786 | -0.35679 |
| C3-Pz | -2.60578 | 0.005174 | 0.155206 | 65535 | -0.00739 | 0.86142 | -0.47575 |
| C3-F9 | 0.731384 | 0.767003 | 0.925693 | 65535 | 0.046881 | 0.089763 | 0.133532 |
| C3-F10 | -0.12657 | 0.449749 | 0.833738 | 65535 | 0.025417 | 0.051857 | -0.02311 |
| C4-P3 | 0.177381 | 0.570243 | 0.865972 | 65535 | 0.014572 | 0.054568 | 0.032385 |
| C4-P4 | 0.159377 | 0.563178 | 0.865972 | 65535 | 0.023376 | 0.052174 | 0.029098 |
| C4-O1 | 0.767108 | 0.777725 | 0.929469 | 65535 | 0.018327 | 0.099731 | 0.140054 |
| C4-O2 | -0.28114 | 0.389548 | 0.802613 | 65535 | 0.011378 | 0.0573 | -0.05133 |
| C4-F7 | 0.282436 | 0.610948 | 0.865972 | 65535 | 0.026775 | 0.056744 | 0.051566 |
| C4-F8 | -2.0327 | 0.022163 | 0.21156 | 65535 | -0.00841 | 0.564414 | -0.37112 |
| C4-T3 | 1.595274 | 0.943337 | 0.990504 | 65535 | 0.040847 | 0.248294 | 0.291256 |
| C4-T4 | -1.94966 | 0.026794 | 0.219429 | 65535 | -0.00561 | 0.492655 | -0.35596 |
| C4-T5 | 1.716051 | 0.955612 | 0.993458 | 65535 | 0.032621 | 0.279033 | 0.313307 |
| C4-T6 | -0.20482 | 0.419031 | 0.814783 | 65535 | 0.018221 | 0.054238 | -0.0374 |
| C4-Fz | 2.128274 | 0.982303 | 0.99558 | 65535 | 0.046615 | 0.39268 | 0.388568 |
| C4-Cz | 2.662401 | 0.99558 | 0.99558 | 65535 | 0.090898 | 0.505733 | 0.486086 |
| C4-Pz | 0.113625 | 0.545136 | 0.865972 | 65535 | 0.021794 | 0.051137 | 0.020745 |
| C4-F9 | -1.71072 | 0.04488 | 0.2856 | 65535 | -0.00094 | 0.403807 | -0.31233 |
| C4-F10 | 0.270345 | 0.606317 | 0.865972 | 65535 | 0.014455 | 0.055792 | 0.049358 |
| P3-P4 | 1.571953 | 0.940681 | 0.990504 | 65535 | 0.025689 | 0.249729 | 0.286998 |
| P3-O1 | -0.84601 | 0.199629 | 0.604766 | 65535 | 0.013167 | 0.183139 | -0.15446 |
| P3-O2 | -0.54732 | 0.292595 | 0.714476 | 65535 | 0.007767 | 0.091912 | -0.09993 |
| P3-F7 | 0.035788 | 0.514244 | 0.865972 | 65535 | 0.018194 | 0.050156 | 0.006534 |
| P3-F8 | -0.04725 | 0.481198 | 0.842097 | 65535 | 0.011217 | 0.050258 | -0.00863 |
| P3-T3 | -1.62057 | 0.053889 | 0.309899 | 65535 | 0.000598 | 0.502008 | -0.29587 |
| P3-T4 | -0.89451 | 0.186435 | 0.604766 | 65535 | 0.004807 | 0.234946 | -0.16331 |
| P3-T5 | -0.87395 | 0.191959 | 0.604766 | 65535 | 0.007693 | 0.165836 | -0.15956 |
| P3-T6 | 1.360908 | 0.911932 | 0.987143 | 65535 | 0.030595 | 0.285835 | 0.248467 |
| P3-Fz | -1.21893 | 0.11265 | 0.473132 | 65535 | 0.003706 | 0.309894 | -0.22255 |
| P3-Cz | -0.72547 | 0.234799 | 0.666323 | 65535 | 0.008912 | 0.149731 | -0.13245 |
| P3-Pz | 0.134736 | 0.553475 | 0.865972 | 65535 | 0.028804 | 0.052192 | 0.024599 |
| P3-F9 | -0.05878 | 0.476614 | 0.842097 | 65535 | 0.012849 | 0.050494 | -0.01073 |
| P3-F10 | 0.639468 | 0.738121 | 0.920363 | 65535 | 0.018115 | 0.08659 | 0.11675 |
| P4-O1 | -1.49316 | 0.069032 | 0.353577 | 65535 | 0.001437 | 0.749213 | -0.27261 |
| P4-O2 | -1.4623 | 0.073159 | 0.364794 | 65535 | 0.002128 | 0.823084 | -0.26698 |
| P4-F7 | -1.03039 | 0.152468 | 0.571754 | 65535 | 0.003569 | 0.15886 | -0.18812 |
| P4-F8 | -0.22302 | 0.411951 | 0.814783 | 65535 | 0.012229 | 0.056156 | -0.04072 |
| P4-T3 | -1.13928 | 0.128448 | 0.499518 | 65535 | 0.005788 | 0.274334 | -0.208 |
| P4-T4 | -0.14338 | 0.443117 | 0.831729 | 65535 | 0.014774 | 0.052209 | -0.02618 |
| P4-T5 | 0.248818 | 0.598033 | 0.865972 | 65535 | 0.018051 | 0.059293 | 0.045428 |
| P4-T6 | 0.148173 | 0.55877 | 0.865972 | 65535 | 0.024565 | 0.052473 | 0.027053 |
| P4-Fz | -0.44768 | 0.327604 | 0.747792 | 65535 | 0.009008 | 0.076721 | -0.08173 |
| P4-Cz | -0.4271 | 0.335041 | 0.756544 | 65535 | 0.016787 | 0.064937 | -0.07798 |
| P4-Pz | -0.46375 | 0.32184 | 0.747792 | 65535 | 0.020696 | 0.086286 | -0.08467 |
| P4-F9 | -0.39393 | 0.347171 | 0.77493 | 65535 | 0.007433 | 0.071465 | -0.07192 |
| P4-F10 | -0.31717 | 0.375839 | 0.802613 | 65535 | 0.009642 | 0.059405 | -0.05791 |
| O1-O2 | -2.10258 | 0.018814 | 0.210718 | 65535 | -0.01056 | 0.660939 | -0.38388 |
| O1-F7 | -0.85321 | 0.197637 | 0.604766 | 65535 | 0.016182 | 0.161446 | -0.15577 |
| O1-F8 | -0.88516 | 0.188935 | 0.604766 | 65535 | 0.011701 | 0.145191 | -0.16161 |
| O1-T3 | -2.49559 | 0.006978 | 0.161103 | 65535 | -0.02057 | 0.974941 | -0.45563 |
| O1-T4 | -0.60745 | 0.272361 | 0.694986 | 65535 | 0.015013 | 0.104209 | -0.1109 |
| O1-T5 | 0.230626 | 0.590998 | 0.865972 | 65535 | 0.029773 | 0.058848 | 0.042106 |
| O1-T6 | -2.07845 | 0.019918 | 0.210718 | 65535 | -0.00621 | 0.653229 | -0.37947 |
| O1-Fz | 0.672798 | 0.748804 | 0.920363 | 65535 | 0.018397 | 0.090321 | 0.122835 |
| O1-Cz | -3.11006 | 0.001173 | 0.082091 | 65535 | -0.02951 | 0.99965 | -0.56782 |
| O1-Pz | -0.58432 | 0.28006 | 0.70015 | 65535 | 0.010437 | 0.086762 | -0.10668 |
| O1-F9 | 0.702754 | 0.758204 | 0.920363 | 65535 | 0.024348 | 0.089112 | 0.128305 |
| O1-F10 | 0.202651 | 0.580122 | 0.865972 | 65535 | 0.019001 | 0.053848 | 0.036999 |
| O2-F7 | -0.95955 | 0.169621 | 0.603735 | 65535 | 0.012074 | 0.171847 | -0.17519 |
| O2-F8 | -2.09861 | 0.018992 | 0.210718 | 65535 | -0.00919 | 0.588111 | -0.38315 |
| O2-T3 | -2.35068 | 0.010199 | 0.178486 | 65535 | -0.01009 | 0.848453 | -0.42917 |
| O2-T4 | 0.83701 | 0.797861 | 0.930837 | 65535 | 0.035372 | 0.11118 | 0.152816 |
| O2-T5 | -2.45622 | 0.007748 | 0.161103 | 65535 | -0.00668 | 0.965934 | -0.44844 |
| O2-T6 | -1.24867 | 0.107129 | 0.459126 | 65535 | 0.005752 | 0.26474 | -0.22797 |
| O2-Fz | -0.10931 | 0.456571 | 0.833738 | 65535 | 0.010597 | 0.051168 | -0.01996 |
| O2-Cz | -2.69217 | 0.004066 | 0.155206 | 65535 | -0.02049 | 0.998941 | -0.49152 |
| O2-Pz | -0.16354 | 0.435185 | 0.831729 | 65535 | 0.015528 | 0.052101 | -0.02986 |
| O2-F9 | -0.45551 | 0.32479 | 0.747792 | 65535 | 0.017266 | 0.068309 | -0.08316 |
| O2-F10 | -0.79782 | 0.213288 | 0.613569 | 65535 | 0.007453 | 0.1744 | -0.14566 |
| F7-F8 | 1.027144 | 0.846773 | 0.942578 | 65535 | 0.137118 | 0.158536 | 0.18753 |
| F7-T3 | -1.36092 | 0.088067 | 0.410978 | 65535 | 0.013531 | 0.298678 | -0.24847 |
| F7-T4 | -1.61398 | 0.054601 | 0.309899 | 65535 | 0.001442 | 0.349009 | -0.29467 |
| F7-T5 | -0.60043 | 0.274685 | 0.694986 | 65535 | 0.031881 | 0.089045 | -0.10962 |
| F7-T6 | -1.84139 | 0.034038 | 0.234996 | 65535 | -0.00524 | 0.516843 | -0.33619 |
| F7-Fz | 0.101974 | 0.540525 | 0.865972 | 65535 | 0.021883 | 0.050893 | 0.018618 |
| F7-Cz | 0.946905 | 0.827189 | 0.942578 | 65535 | 0.054162 | 0.129062 | 0.17288 |
| F7-Pz | -1.29258 | 0.09934 | 0.443861 | 65535 | 0.002143 | 0.302788 | -0.23599 |
| F7-F9 | 0.139911 | 0.555515 | 0.865972 | 65535 | 0.066062 | 0.05205 | 0.025544 |
| F7-F10 | 0.892007 | 0.812898 | 0.94249 | 65535 | 0.088243 | 0.15217 | 0.162858 |
| F8-T3 | 0.480356 | 0.684068 | 0.887364 | 65535 | 0.063178 | 0.07003 | 0.087701 |
| F8-T4 | -2.05442 | 0.021072 | 0.210718 | 65535 | -0.01562 | 0.468197 | -0.37508 |
| F8-T5 | -0.82243 | 0.206246 | 0.604766 | 65535 | 0.017791 | 0.119183 | -0.15015 |
| F8-T6 | -1.85733 | 0.032879 | 0.234996 | 65535 | -0.00567 | 0.473091 | -0.3391 |
| F8-Fz | -0.14218 | 0.443589 | 0.831729 | 65535 | 0.018313 | 0.051795 | -0.02596 |
| F8-Cz | 0.778343 | 0.781038 | 0.929469 | 65535 | 0.045856 | 0.108522 | 0.142106 |
| F8-Pz | -0.878 | 0.190865 | 0.604766 | 65535 | 0.004639 | 0.194524 | -0.1603 |
| F8-F9 | -2.42379 | 0.008439 | 0.161103 | 65535 | -0.02866 | 0.624369 | -0.44252 |
| F8-F10 | 2.064091 | 0.979399 | 0.99558 | 65535 | 0.106481 | 0.409946 | 0.37685 |
| T3-T4 | 0.347906 | 0.635735 | 0.884134 | 65535 | 0.057132 | 0.060968 | 0.063519 |
| T3-T5 | -0.65151 | 0.257992 | 0.685803 | 65535 | 0.034802 | 0.094328 | -0.11895 |
| T3-T6 | -0.85499 | 0.197146 | 0.604766 | 65535 | 0.018342 | 0.143552 | -0.1561 |
| T3-Fz | -1.52325 | 0.065186 | 0.350999 | 65535 | 0.001086 | 0.557041 | -0.27811 |
| T3-Cz | -0.81854 | 0.207348 | 0.604766 | 65535 | 0.019876 | 0.182881 | -0.14944 |
| T3-Pz | -1.92668 | 0.028212 | 0.219429 | 65535 | -0.00272 | 0.999999 | -0.35176 |
| T3-F9 | 0.918828 | 0.81997 | 0.94249 | 65535 | 0.091914 | 0.147894 | 0.167755 |
| T3-F10 | 0.620234 | 0.73185 | 0.920363 | 65535 | 0.089905 | 0.105697 | 0.113239 |
| T4-T5 | -2.12156 | 0.017983 | 0.210718 | 65535 | -0.01091 | 0.676844 | -0.38734 |
| T4-T6 | -0.49168 | 0.311931 | 0.745482 | 65535 | 0.036722 | 0.079332 | -0.08977 |
| T4-Fz | 0.677247 | 0.750213 | 0.920363 | 65535 | 0.023301 | 0.081704 | 0.123648 |
| T4-Cz | -0.20751 | 0.417986 | 0.814783 | 65535 | 0.016272 | 0.057737 | -0.03789 |
| T4-Pz | -1.18449 | 0.1193 | 0.491234 | 65535 | 0.002441 | 0.375283 | -0.21626 |
| T4-F9 | -3.28151 | 0.000679 | 0.071307 | 65535 | -0.05716 | 0.860022 | -0.59912 |
| T4-F10 | -0.30782 | 0.379381 | 0.802613 | 65535 | 0.03646 | 0.060829 | -0.0562 |
| T5-T6 | -0.91464 | 0.181122 | 0.604766 | 65535 | 0.009972 | 0.199817 | -0.16699 |
| T5-Fz | 0.194841 | 0.577074 | 0.865972 | 65535 | 0.010008 | 0.053656 | 0.035573 |
| T5-Cz | 0.082858 | 0.532947 | 0.865972 | 65535 | 0.018541 | 0.050621 | 0.015127 |
| T5-Pz | -1.83262 | 0.03469 | 0.234996 | 65535 | -0.00232 | 0.937278 | -0.33459 |
| T5-F9 | -1.65881 | 0.049905 | 0.299432 | 65535 | -2.7E-05 | 0.445917 | -0.30285 |
| T5-F10 | -0.70186 | 0.242073 | 0.668887 | 65535 | 0.029731 | 0.103587 | -0.12814 |
| T6-Fz | 0.102746 | 0.54083 | 0.865972 | 65535 | 0.015655 | 0.051104 | 0.018759 |
| T6-Cz | -1.66581 | 0.049202 | 0.299432 | 65535 | -0.00015 | 0.554224 | -0.30413 |
| T6-Pz | -1.94032 | 0.027364 | 0.219429 | 65535 | -0.00251 | 0.96317 | -0.35425 |
| T6-F9 | -2.30449 | 0.011472 | 0.185314 | 65535 | -0.01775 | 0.599141 | -0.42074 |
| T6-F10 | -0.99415 | 0.161091 | 0.583262 | 65535 | 0.017713 | 0.176063 | -0.18151 |
| Fz-Cz | -2.00596 | 0.023574 | 0.215242 | 65535 | -0.00594 | 0.880454 | -0.36624 |
| Fz-Pz | -1.49983 | 0.068165 | 0.353577 | 65535 | 0.000924 | 0.490383 | -0.27383 |
| Fz-F9 | -0.011 | 0.49562 | 0.853117 | 65535 | 0.017867 | 0.05001 | -0.00201 |
| Fz-F10 | -0.11499 | 0.454326 | 0.833738 | 65535 | 0.012982 | 0.051293 | -0.02099 |
| Cz-Pz | 1.090345 | 0.861109 | 0.946769 | 65535 | 0.042038 | 0.158725 | 0.199069 |
| Cz-F9 | -0.28037 | 0.389841 | 0.802613 | 65535 | 0.010588 | 0.057013 | -0.05119 |
| Cz-F10 | -0.7144 | 0.238194 | 0.666943 | 65535 | 0.004693 | 0.106119 | -0.13043 |
| Pz-F9 | -0.82444 | 0.205677 | 0.604766 | 65535 | 0.00393 | 0.273455 | -0.15052 |
| Pz-F10 | -0.57286 | 0.283916 | 0.701439 | 65535 | 0.003898 | 0.101991 | -0.10459 |
| F9-F10 | -0.36603 | 0.357499 | 0.782029 | 65535 | 0.043935 | 0.064271 | -0.06683 |

Table S3g: theta_coh_HC_PT

| channel | tvalue | pvalue | fdr_pvalue | CI_low | CI_high | statistical power | effect size |
| --- | --- | --- | --- | --- | --- | --- | --- |
| Fp1-Fp2 | 0.780191 | 0.21842 | 0.999992 | -0.04511 | 65535 | 0.11562 | 0.142442 |
| Fp1-F3 | -0.57699 | 0.717476 | 0.999992 | -0.07679 | 65535 | 0.084742 | -0.10534 |
| Fp1-F4 | 2.061338 | 0.020734 | 0.77992 | 0.010487 | 65535 | 0.473814 | 0.376347 |
| Fp1-C3 | -0.6864 | 0.753097 | 0.999992 | -0.05236 | 65535 | 0.161936 | -0.12532 |
| Fp1-C4 | 2.932199 | 0.002022 | 0.424584 | 0.024795 | 65535 | 0.590094 | 0.535344 |
| Fp1-P3 | -0.31894 | 0.624831 | 0.999992 | -0.02678 | 65535 | 0.058384 | -0.05823 |
| Fp1-P4 | -2.41217 | 0.991301 | 0.999992 | -0.05956 | 65535 | 0.897518 | -0.4404 |
| Fp1-O1 | -1.86137 | 0.967409 | 0.999992 | -0.07522 | 65535 | 0.522371 | -0.33984 |
| Fp1-O2 | -2.44489 | 0.992016 | 0.999992 | -0.08911 | 65535 | 0.877245 | -0.44637 |
| Fp1-F7 | 0.504603 | 0.307389 | 0.999992 | -0.05411 | 65535 | 0.078432 | 0.092128 |
| Fp1-F8 | 1.636042 | 0.052248 | 0.999992 | -0.00085 | 65535 | 0.302616 | 0.298699 |
| Fp1-T3 | -0.96704 | 0.832249 | 0.999992 | -0.10981 | 65535 | 0.156092 | -0.17656 |
| Fp1-T4 | 0.218866 | 0.413566 | 0.999992 | -0.03284 | 65535 | 0.054339 | 0.039959 |
| Fp1-T5 | 0.155797 | 0.43823 | 0.999992 | -0.04594 | 65535 | 0.053034 | 0.028445 |
| Fp1-T6 | -1.6418 | 0.948353 | 0.999992 | -0.05569 | 65535 | 0.579021 | -0.29975 |
| Fp1-Fz | -0.26001 | 0.602344 | 0.999992 | -0.04122 | 65535 | 0.057934 | -0.04747 |
| Fp1-Cz | -0.55065 | 0.708543 | 0.999992 | -0.07582 | 65535 | 0.090399 | -0.10053 |
| Fp1-Pz | -1.03916 | 0.849572 | 0.999992 | -0.03898 | 65535 | 0.200471 | -0.18972 |
| Fp1-F9 | -0.87043 | 0.807082 | 0.999992 | -0.05785 | 65535 | 0.143099 | -0.15892 |
| Fp1-F10 | -0.44202 | 0.670359 | 0.999992 | -0.0545 | 65535 | 0.069417 | -0.0807 |
| Fp2-F3 | 0.518264 | 0.302622 | 0.999992 | -0.02779 | 65535 | 0.072172 | 0.094622 |
| Fp2-F4 | -0.19985 | 0.579029 | 0.999992 | -0.06796 | 65535 | 0.054394 | -0.03649 |
| Fp2-C3 | -2.35843 | 0.990002 | 0.999992 | -0.09366 | 65535 | 0.976837 | -0.43059 |
| Fp2-C4 | 1.29545 | 0.098847 | 0.999992 | -0.00673 | 65535 | 0.192223 | 0.236515 |
| Fp2-P3 | -0.61703 | 0.730797 | 0.999992 | -0.03356 | 65535 | 0.092454 | -0.11265 |
| Fp2-P4 | -1.05492 | 0.853193 | 0.999992 | -0.03772 | 65535 | 0.183155 | -0.1926 |
| Fp2-O1 | -2.71545 | 0.996194 | 0.999992 | -0.10352 | 65535 | 0.893858 | -0.49577 |
| Fp2-O2 | -3.35819 | 0.999472 | 0.999992 | -0.12325 | 65535 | 0.998774 | -0.61312 |
| Fp2-F7 | -0.16346 | 0.564781 | 0.999992 | -0.07434 | 65535 | 0.052651 | -0.02984 |
| Fp2-F8 | -1.33077 | 0.907084 | 0.999992 | -0.13089 | 65535 | 0.247827 | -0.24296 |
| Fp2-T3 | -0.67414 | 0.74923 | 0.999992 | -0.07095 | 65535 | 0.114687 | -0.12308 |
| Fp2-T4 | -0.43162 | 0.666598 | 0.999992 | -0.06674 | 65535 | 0.068917 | -0.0788 |
| Fp2-T5 | -0.80989 | 0.790182 | 0.999992 | -0.04461 | 65535 | 0.110559 | -0.14786 |
| Fp2-T6 | -0.82484 | 0.794436 | 0.999992 | -0.06483 | 65535 | 0.133938 | -0.15059 |
| Fp2-Fz | -0.84973 | 0.801402 | 0.999992 | -0.0717 | 65535 | 0.128836 | -0.15514 |
| Fp2-Cz | -1.59632 | 0.943454 | 0.999992 | -0.11209 | 65535 | 0.430802 | -0.29145 |
| Fp2-Pz | -0.74663 | 0.771613 | 0.999992 | -0.04389 | 65535 | 0.130865 | -0.13631 |
| Fp2-F9 | -0.81968 | 0.792974 | 0.999992 | -0.06481 | 65535 | 0.116626 | -0.14965 |
| Fp2-F10 | 1.138983 | 0.128509 | 0.999992 | -0.01169 | 65535 | 0.16328 | 0.207949 |
| F3-F4 | 2.479589 | 0.007283 | 0.714212 | 0.017954 | 65535 | 0.450247 | 0.452709 |
| F3-C3 | -0.10143 | 0.540308 | 0.999992 | -0.04318 | 65535 | 0.051297 | -0.01852 |
| F3-C4 | 1.8099 | 0.036428 | 0.999992 | 0.001593 | 65535 | 0.300133 | 0.330441 |
| F3-P3 | -1.0478 | 0.851563 | 0.999992 | -0.02199 | 65535 | 0.164448 | -0.1913 |
| F3-P4 | -0.38498 | 0.649528 | 0.999992 | -0.01458 | 65535 | 0.06429 | -0.07029 |
| F3-O1 | 0.124308 | 0.450641 | 0.999992 | -0.01572 | 65535 | 0.051543 | 0.022696 |
| F3-O2 | -0.02203 | 0.50877 | 0.999992 | -0.01596 | 65535 | 0.050058 | -0.00402 |
| F3-F7 | -0.47246 | 0.681266 | 0.999992 | -0.07934 | 65535 | 0.079787 | -0.08626 |
| F3-F8 | 2.240027 | 0.013481 | 0.714212 | 0.01381 | 65535 | 0.482433 | 0.408971 |
| F3-T3 | -3.35356 | 0.999463 | 0.999992 | -0.11179 | 65535 | 0.99749 | -0.61227 |
| F3-T4 | -1.4007 | 0.918036 | 0.999992 | -0.04454 | 65535 | 0.257939 | -0.25573 |
| F3-T5 | -1.99142 | 0.975628 | 0.999992 | -0.05984 | 65535 | 0.64047 | -0.36358 |
| F3-T6 | -2.04289 | 0.978355 | 0.999992 | -0.03522 | 65535 | 0.487853 | -0.37298 |
| F3-Fz | 2.236354 | 0.013604 | 0.714212 | 0.013591 | 65535 | 0.483314 | 0.408301 |
| F3-Cz | -0.47577 | 0.68244 | 0.999992 | -0.04382 | 65535 | 0.083713 | -0.08686 |
| F3-Pz | -2.13596 | 0.982625 | 0.999992 | -0.03602 | 65535 | 0.93312 | -0.38997 |
| F3-F9 | -2.31902 | 0.988943 | 0.999992 | -0.08242 | 65535 | 0.631232 | -0.42339 |
| F3-F10 | -4.51102 | 0.999992 | 0.999992 | -0.12524 | 65535 | 0.999997 | -0.8236 |
| F4-C3 | -0.55366 | 0.70957 | 0.999992 | -0.02957 | 65535 | 0.080779 | -0.10108 |
| F4-C4 | 1.007301 | 0.157926 | 0.999992 | -0.01506 | 65535 | 0.13891 | 0.183907 |
| F4-P3 | -1.78205 | 0.961344 | 0.999992 | -0.0212 | 65535 | 0.672444 | -0.32536 |
| F4-P4 | -1.51128 | 0.933305 | 0.999992 | -0.0208 | 65535 | 0.357262 | -0.27592 |
| F4-O1 | 0.421891 | 0.336936 | 0.999992 | -0.01597 | 65535 | 0.064982 | 0.077026 |
| F4-O2 | -0.70332 | 0.75838 | 0.999992 | -0.02943 | 65535 | 0.101609 | -0.12841 |
| F4-F7 | 0.790672 | 0.215361 | 0.999992 | -0.02245 | 65535 | 0.109384 | 0.144356 |
| F4-F8 | 0.972749 | 0.166334 | 0.999992 | -0.02402 | 65535 | 0.15687 | 0.177599 |
| F4-T3 | -0.66409 | 0.746036 | 0.999992 | -0.04022 | 65535 | 0.097297 | -0.12125 |
| F4-T4 | -0.90753 | 0.817013 | 0.999992 | -0.04858 | 65535 | 0.143666 | -0.16569 |
| F4-T5 | -2.3797 | 0.990535 | 0.999992 | -0.03579 | 65535 | 0.969139 | -0.43447 |
| F4-T6 | -0.80448 | 0.788632 | 0.999992 | -0.03201 | 65535 | 0.12673 | -0.14688 |
| F4-Fz | 1.540383 | 0.063072 | 0.999992 | -0.00353 | 65535 | 0.28066 | 0.281234 |
| F4-Cz | 1.597688 | 0.056393 | 0.999992 | -0.00146 | 65535 | 0.238018 | 0.291697 |
| F4-Pz | -1.62892 | 0.947002 | 0.999992 | -0.03478 | 65535 | 0.609779 | -0.2974 |
| F4-F9 | -2.36595 | 0.990193 | 0.999992 | -0.06704 | 65535 | 0.759263 | -0.43196 |
| F4-F10 | -2.48039 | 0.992733 | 0.999992 | -0.0539 | 65535 | 0.803771 | -0.45286 |
| C3-C4 | 0.205701 | 0.418689 | 0.999992 | -0.0181 | 65535 | 0.05326 | 0.037556 |
| C3-P3 | -0.85199 | 0.802028 | 0.999992 | -0.05382 | 65535 | 0.114169 | -0.15555 |
| C3-P4 | -3.06989 | 0.998671 | 0.999992 | -0.04742 | 65535 | 1 | -0.56048 |
| C3-O1 | -3.48104 | 0.99965 | 0.999992 | -0.08492 | 65535 | 0.999995 | -0.63555 |
| C3-O2 | -3.69804 | 0.999834 | 0.999992 | -0.05523 | 65535 | 1 | -0.67517 |
| C3-F7 | -0.3759 | 0.646166 | 0.999992 | -0.03804 | 65535 | 0.068496 | -0.06863 |
| C3-F8 | -1.42354 | 0.921391 | 0.999992 | -0.05607 | 65535 | 0.386645 | -0.2599 |
| C3-T3 | -1.75911 | 0.959425 | 0.999992 | -0.0673 | 65535 | 0.534551 | -0.32117 |
| C3-T4 | -1.4807 | 0.929324 | 0.999992 | -0.03942 | 65535 | 0.358468 | -0.27034 |
| C3-T5 | -0.34849 | 0.635954 | 0.999992 | -0.01883 | 65535 | 0.061814 | -0.06363 |
| C3-T6 | -2.88372 | 0.997664 | 0.999992 | -0.05227 | 65535 | 0.997507 | -0.52649 |
| C3-Fz | 0.479177 | 0.31635 | 0.999992 | -0.01788 | 65535 | 0.073857 | 0.087486 |
| C3-Cz | -3.06289 | 0.998642 | 0.999992 | -0.11751 | 65535 | 0.999524 | -0.55921 |
| C3-Pz | -3.99418 | 0.999943 | 0.999992 | -0.05406 | 65535 | 0.999982 | -0.72923 |
| C3-F9 | -0.09547 | 0.537949 | 0.999992 | -0.03058 | 65535 | 0.050764 | -0.01743 |
| C3-F10 | -1.72733 | 0.956638 | 0.999992 | -0.04667 | 65535 | 0.650268 | -0.31537 |
| C4-P3 | -0.22957 | 0.590587 | 0.999992 | -0.01328 | 65535 | 0.054729 | -0.04191 |
| C4-P4 | -0.70461 | 0.758779 | 0.999992 | -0.04684 | 65535 | 0.096042 | -0.12864 |
| C4-O1 | -0.50843 | 0.69395 | 0.999992 | -0.01439 | 65535 | 0.083841 | -0.09283 |
| C4-O2 | -1.00729 | 0.842071 | 0.999992 | -0.01974 | 65535 | 0.205646 | -0.1839 |
| C4-F7 | 1.725402 | 0.043536 | 0.999992 | 0.000801 | 65535 | 0.265002 | 0.315014 |
| C4-F8 | -0.681 | 0.751398 | 0.999992 | -0.04076 | 65535 | 0.094116 | -0.12433 |
| C4-T3 | 1.430478 | 0.077611 | 0.999992 | -0.00283 | 65535 | 0.187881 | 0.261168 |
| C4-T4 | -1.99283 | 0.975706 | 0.999992 | -0.0543 | 65535 | 0.62742 | -0.36384 |
| C4-T5 | 0.643273 | 0.260647 | 0.999992 | -0.00804 | 65535 | 0.103363 | 0.117445 |
| C4-T6 | 1.598554 | 0.056297 | 0.999992 | -0.00064 | 65535 | 0.251241 | 0.291855 |
| C4-Fz | 1.132689 | 0.129821 | 0.999992 | -0.00812 | 65535 | 0.171509 | 0.2068 |
| C4-Cz | 2.030373 | 0.022283 | 0.77992 | 0.009938 | 65535 | 0.329593 | 0.370694 |
| C4-Pz | -0.53487 | 0.703129 | 0.999992 | -0.03254 | 65535 | 0.084834 | -0.09765 |
| C4-F9 | -1.93522 | 0.972322 | 0.999992 | -0.04007 | 65535 | 0.61763 | -0.35332 |
| C4-F10 | 0.883599 | 0.189355 | 0.999992 | -0.00589 | 65535 | 0.112208 | 0.161322 |
| P3-P4 | 0.613786 | 0.270269 | 0.999992 | -0.01029 | 65535 | 0.078993 | 0.112061 |
| P3-O1 | -1.64393 | 0.948574 | 0.999992 | -0.05862 | 65535 | 0.596026 | -0.30014 |
| P3-O2 | -1.62968 | 0.947082 | 0.999992 | -0.0294 | 65535 | 0.567432 | -0.29754 |
| P3-F7 | -1.11124 | 0.865637 | 0.999992 | -0.03104 | 65535 | 0.21983 | -0.20288 |
| P3-F8 | -0.89673 | 0.814156 | 0.999992 | -0.02485 | 65535 | 0.137085 | -0.16372 |
| P3-T3 | -1.40679 | 0.91894 | 0.999992 | -0.04794 | 65535 | 0.34594 | -0.25684 |
| P3-T4 | -1.26769 | 0.896297 | 0.999992 | -0.02111 | 65535 | 0.329096 | -0.23145 |
| P3-T5 | -0.19334 | 0.576486 | 0.999992 | -0.02738 | 65535 | 0.054052 | -0.0353 |
| P3-T6 | 0.58759 | 0.278965 | 0.999992 | -0.01157 | 65535 | 0.089929 | 0.107279 |
| P3-Fz | -1.63621 | 0.94777 | 0.999992 | -0.03189 | 65535 | 0.607571 | -0.29873 |
| P3-Cz | -1.06664 | 0.855845 | 0.999992 | -0.035 | 65535 | 0.276769 | -0.19474 |
| P3-Pz | -0.57963 | 0.718365 | 0.999992 | -0.03879 | 65535 | 0.093582 | -0.10583 |
| P3-F9 | -1.44644 | 0.924648 | 0.999992 | -0.02615 | 65535 | 0.475243 | -0.26408 |
| P3-F10 | -0.34772 | 0.635665 | 0.999992 | -0.01434 | 65535 | 0.061553 | -0.06348 |
| P4-O1 | -2.21046 | 0.985499 | 0.999992 | -0.03979 | 65535 | 0.959004 | -0.40357 |
| P4-O2 | -1.40015 | 0.917953 | 0.999992 | -0.03638 | 65535 | 0.48185 | -0.25563 |
| P4-F7 | -2.64428 | 0.99535 | 0.999992 | -0.0439 | 65535 | 0.873972 | -0.48278 |
| P4-F8 | -1.50782 | 0.932863 | 0.999992 | -0.03957 | 65535 | 0.382518 | -0.27529 |
| P4-T3 | -1.72371 | 0.956311 | 0.999992 | -0.04636 | 65535 | 0.468605 | -0.31471 |
| P4-T4 | -0.62217 | 0.732484 | 0.999992 | -0.02467 | 65535 | 0.089401 | -0.11359 |
| P4-T5 | 0.238449 | 0.405973 | 0.999992 | -0.01458 | 65535 | 0.055403 | 0.043535 |
| P4-T6 | -0.28618 | 0.612378 | 0.999992 | -0.03341 | 65535 | 0.059766 | -0.05225 |
| P4-Fz | -1.85578 | 0.96701 | 0.999992 | -0.02639 | 65535 | 0.696319 | -0.33882 |
| P4-Cz | -1.77853 | 0.961054 | 0.999992 | -0.05608 | 65535 | 0.409987 | -0.32471 |
| P4-Pz | -1.15426 | 0.874638 | 0.999992 | -0.04927 | 65535 | 0.255594 | -0.21074 |
| P4-F9 | -1.9736 | 0.974618 | 0.999992 | -0.02115 | 65535 | 0.641571 | -0.36033 |
| P4-F10 | -0.66241 | 0.7455 | 0.999992 | -0.02366 | 65535 | 0.094368 | -0.12094 |
| O1-O2 | -2.57551 | 0.994378 | 0.999992 | -0.11996 | 65535 | 0.833477 | -0.47022 |
| O1-F7 | -1.8947 | 0.96971 | 0.999992 | -0.0686 | 65535 | 0.606508 | -0.34592 |
| O1-F8 | -1.88499 | 0.969054 | 0.999992 | -0.05665 | 65535 | 0.640436 | -0.34415 |
| O1-T3 | -3.30107 | 0.999363 | 0.999992 | -0.11674 | 65535 | 0.999923 | -0.60269 |
| O1-T4 | -1.75047 | 0.958682 | 0.999992 | -0.0467 | 65535 | 0.713807 | -0.31959 |
| O1-T5 | -0.69847 | 0.756873 | 0.999992 | -0.03189 | 65535 | 0.166623 | -0.12752 |
| O1-T6 | -2.4858 | 0.992837 | 0.999992 | -0.04954 | 65535 | 0.857867 | -0.45384 |
| O1-Fz | -1.19597 | 0.882947 | 0.999992 | -0.02449 | 65535 | 0.204193 | -0.21835 |
| O1-Cz | -4.28183 | 0.999981 | 0.999992 | -0.11828 | 65535 | 1 | -0.78175 |
| O1-Pz | -2.91611 | 0.997878 | 0.999992 | -0.05855 | 65535 | 0.987132 | -0.53241 |
| O1-F9 | -0.31565 | 0.623587 | 0.999992 | -0.0207 | 65535 | 0.06166 | -0.05763 |
| O1-F10 | -0.49093 | 0.687806 | 0.999992 | -0.02023 | 65535 | 0.07584 | -0.08963 |
| O2-F7 | -1.5346 | 0.936221 | 0.999992 | -0.05157 | 65535 | 0.377098 | -0.28018 |
| O2-F8 | -2.12869 | 0.98232 | 0.999992 | -0.07552 | 65535 | 0.586971 | -0.38864 |
| O2-T3 | -3.09044 | 0.998753 | 0.999992 | -0.0637 | 65535 | 0.998815 | -0.56423 |
| O2-T4 | 0.227554 | 0.410194 | 0.999992 | -0.01758 | 65535 | 0.055546 | 0.041545 |
| O2-T5 | -3.01793 | 0.998441 | 0.999992 | -0.05042 | 65535 | 0.975503 | -0.551 |
| O2-T6 | -0.52955 | 0.701289 | 0.999992 | -0.02566 | 65535 | 0.083713 | -0.09668 |
| O2-Fz | -1.31044 | 0.903704 | 0.999992 | -0.02426 | 65535 | 0.280229 | -0.23925 |
| O2-Cz | -3.75147 | 0.999863 | 0.999992 | -0.11066 | 65535 | 1 | -0.68492 |
| O2-Pz | -1.79036 | 0.96202 | 0.999992 | -0.04964 | 65535 | 0.334734 | -0.32687 |
| O2-F9 | -0.84982 | 0.801426 | 0.999992 | -0.02828 | 65535 | 0.154972 | -0.15515 |
| O2-F10 | -0.38738 | 0.650415 | 0.999992 | -0.01977 | 65535 | 0.066107 | -0.07073 |
| F7-F8 | 1.25413 | 0.106137 | 0.999992 | -0.01995 | 65535 | 0.208212 | 0.228972 |
| F7-T3 | -1.81093 | 0.963652 | 0.999992 | -0.15071 | 65535 | 0.49753 | -0.33063 |
| F7-T4 | -1.13894 | 0.871482 | 0.999992 | -0.07957 | 65535 | 0.2181 | -0.20794 |
| F7-T5 | -1.24947 | 0.893017 | 0.999992 | -0.07636 | 65535 | 0.260598 | -0.22812 |
| F7-T6 | -2.57372 | 0.994351 | 0.999992 | -0.10038 | 65535 | 0.891781 | -0.4699 |
| F7-Fz | -0.40314 | 0.656212 | 0.999992 | -0.03087 | 65535 | 0.066041 | -0.0736 |
| F7-Cz | -0.7672 | 0.777751 | 0.999992 | -0.05631 | 65535 | 0.112252 | -0.14007 |
| F7-Pz | -2.31668 | 0.988877 | 0.999992 | -0.04169 | 65535 | 0.756543 | -0.42297 |
| F7-F9 | -0.32455 | 0.626952 | 0.999992 | -0.06181 | 65535 | 0.061564 | -0.05925 |
| F7-F10 | 0.122557 | 0.451333 | 0.999992 | -0.0489 | 65535 | 0.051875 | 0.022376 |
| F8-T3 | 0.0606 | 0.47589 | 0.999992 | -0.04238 | 65535 | 0.050319 | 0.011064 |
| F8-T4 | -2.74559 | 0.996507 | 0.999992 | -0.1631 | 65535 | 0.771449 | -0.50127 |
| F8-T5 | -1.46818 | 0.927642 | 0.999992 | -0.05226 | 65535 | 0.285749 | -0.26805 |
| F8-T6 | -1.95542 | 0.973551 | 0.999992 | -0.08604 | 65535 | 0.55485 | -0.35701 |
| F8-Fz | -0.26974 | 0.606085 | 0.999992 | -0.0262 | 65535 | 0.057635 | -0.04925 |
| F8-Cz | -0.47523 | 0.682249 | 0.999992 | -0.05103 | 65535 | 0.074783 | -0.08676 |
| F8-Pz | -1.0991 | 0.863019 | 0.999992 | -0.03457 | 65535 | 0.308934 | -0.20067 |
| F8-F9 | -2.5408 | 0.993821 | 0.999992 | -0.13647 | 65535 | 0.702231 | -0.46389 |
| F8-F10 | 1.458848 | 0.073632 | 0.999992 | -0.00464 | 65535 | 0.237936 | 0.266348 |
| T3-T4 | 1.359988 | 0.088213 | 0.999992 | -0.00796 | 65535 | 0.227424 | 0.248298 |
| T3-T5 | 0.0136 | 0.494586 | 0.999992 | -0.05103 | 65535 | 0.050021 | 0.002483 |
| T3-T6 | -0.68705 | 0.753302 | 0.999992 | -0.04768 | 65535 | 0.114397 | -0.12544 |
| T3-Fz | -3.58532 | 0.999755 | 0.999992 | -0.0551 | 65535 | 0.999881 | -0.65459 |
| T3-Cz | -1.55164 | 0.938287 | 0.999992 | -0.08095 | 65535 | 0.562856 | -0.28329 |
| T3-Pz | -2.4536 | 0.992198 | 0.999992 | -0.05589 | 65535 | 0.999854 | -0.44796 |
| T3-F9 | 1.133312 | 0.12969 | 0.999992 | -0.01798 | 65535 | 0.200896 | 0.206913 |
| T3-F10 | 0.958168 | 0.169969 | 0.999992 | -0.02627 | 65535 | 0.193314 | 0.174937 |
| T4-T5 | -2.1416 | 0.982859 | 0.999992 | -0.08114 | 65535 | 0.610343 | -0.391 |
| T4-T6 | -0.30215 | 0.618463 | 0.999992 | -0.05921 | 65535 | 0.060499 | -0.05516 |
| T4-Fz | -0.30086 | 0.617973 | 0.999992 | -0.01888 | 65535 | 0.057471 | -0.05493 |
| T4-Cz | -1.50049 | 0.93192 | 0.999992 | -0.0368 | 65535 | 0.758879 | -0.27395 |
| T4-Pz | -2.34691 | 0.989702 | 0.999992 | -0.03169 | 65535 | 0.95076 | -0.42849 |
| T4-F9 | -3.25903 | 0.99927 | 0.999992 | -0.15625 | 65535 | 0.822276 | -0.59502 |
| T4-F10 | -0.28906 | 0.613481 | 0.999992 | -0.05551 | 65535 | 0.058168 | -0.05278 |
| T5-T6 | -1.71125 | 0.955169 | 0.999992 | -0.0434 | 65535 | 0.485156 | -0.31243 |
| T5-Fz | -1.53525 | 0.936301 | 0.999992 | -0.01769 | 65535 | 0.512154 | -0.2803 |
| T5-Cz | -1.46233 | 0.926846 | 0.999992 | -0.02775 | 65535 | 0.30803 | -0.26698 |
| T5-Pz | -2.34305 | 0.9896 | 0.999992 | -0.06083 | 65535 | 0.999829 | -0.42778 |
| T5-F9 | -2.03539 | 0.977974 | 0.999992 | -0.10025 | 65535 | 0.579533 | -0.37161 |
| T5-F10 | -0.73105 | 0.7669 | 0.999992 | -0.06793 | 65535 | 0.10912 | -0.13347 |
| T6-Fz | -0.57712 | 0.717521 | 0.999992 | -0.01633 | 65535 | 0.091749 | -0.10537 |
| T6-Cz | -2.46079 | 0.992345 | 0.999992 | -0.07185 | 65535 | 0.957953 | -0.44928 |
| T6-Pz | -2.55521 | 0.994058 | 0.999992 | -0.04347 | 65535 | 0.996836 | -0.46652 |
| T6-F9 | -1.48673 | 0.930123 | 0.999992 | -0.07997 | 65535 | 0.302168 | -0.27144 |
| T6-F10 | -1.52709 | 0.935292 | 0.999992 | -0.0833 | 65535 | 0.358263 | -0.27881 |
| Fz-Cz | -3.22553 | 0.999186 | 0.999992 | -0.10843 | 65535 | 0.997512 | -0.5889 |
| Fz-Pz | -3.08583 | 0.998735 | 0.999992 | -0.03575 | 65535 | 0.996001 | -0.56339 |
| Fz-F9 | -2.54675 | 0.99392 | 0.999992 | -0.02674 | 65535 | 0.937668 | -0.46497 |
| Fz-F10 | -1.56121 | 0.939424 | 0.999992 | -0.0218 | 65535 | 0.406945 | -0.28504 |
| Cz-Pz | -0.14467 | 0.557393 | 0.999992 | -0.03808 | 65535 | 0.052108 | -0.02641 |
| Cz-F9 | -2.09616 | 0.980898 | 0.999992 | -0.03976 | 65535 | 0.993537 | -0.38271 |
| Cz-F10 | -0.81726 | 0.792288 | 0.999992 | -0.01859 | 65535 | 0.20639 | -0.14921 |
| Pz-F9 | -1.78562 | 0.961635 | 0.999992 | -0.02123 | 65535 | 0.749026 | -0.32601 |
| Pz-F10 | -1.75153 | 0.958774 | 0.999992 | -0.02051 | 65535 | 0.804248 | -0.31978 |
| F9-F10 | -1.21483 | 0.886571 | 0.999992 | -0.09149 | 65535 | 0.216022 | -0.2218 |

Table S3h: theta_coh_PT_HC

| channel | tvalue | pvalue | fdr_pvalue | CI_low | CI_high | statistical power | effect size |
| --- | --- | --- | --- | --- | --- | --- | --- |
| Fp1-Fp2 | 0.780191 | 0.78158 | 0.885883 | 65535 | 0.125299 | 0.11562 | 0.142442 |
| Fp1-F3 | -0.57699 | 0.282524 | 0.442761 | 65535 | 0.037139 | 0.084742 | -0.10534 |
| Fp1-F4 | 2.061338 | 0.979266 | 0.996005 | 65535 | 0.096674 | 0.473814 | 0.376347 |
| Fp1-C3 | -0.6864 | 0.246903 | 0.414302 | 65535 | 0.021697 | 0.161936 | -0.12532 |
| Fp1-C4 | 2.932199 | 0.997978 | 0.997978 | 65535 | 0.089309 | 0.590094 | 0.535344 |
| Fp1-P3 | -0.31894 | 0.375169 | 0.51329 | 65535 | 0.01814 | 0.058384 | -0.05823 |
| Fp1-P4 | -2.41217 | 0.008699 | 0.055358 | 65535 | -0.01104 | 0.897518 | -0.4404 |
| Fp1-O1 | -1.86137 | 0.03259 | 0.123712 | 65535 | -0.00435 | 0.522371 | -0.33984 |
| Fp1-O2 | -2.44489 | 0.007984 | 0.052392 | 65535 | -0.01709 | 0.877245 | -0.44637 |
| Fp1-F7 | 0.504603 | 0.692611 | 0.808046 | 65535 | 0.10146 | 0.078432 | 0.092128 |
| Fp1-F8 | 1.636042 | 0.947752 | 0.985287 | 65535 | 0.128898 | 0.302616 | 0.298699 |
| Fp1-T3 | -0.96704 | 0.167751 | 0.332337 | 65535 | 0.028901 | 0.156092 | -0.17656 |
| Fp1-T4 | 0.218866 | 0.586434 | 0.703721 | 65535 | 0.042825 | 0.054339 | 0.039959 |
| Fp1-T5 | 0.155797 | 0.56177 | 0.681918 | 65535 | 0.055464 | 0.053034 | 0.028445 |
| Fp1-T6 | -1.6418 | 0.051647 | 0.154578 | 65535 | 0.000271 | 0.579021 | -0.29975 |
| Fp1-Fz | -0.26001 | 0.397656 | 0.521923 | 65535 | 0.030047 | 0.057934 | -0.04747 |
| Fp1-Cz | -0.55065 | 0.291457 | 0.450045 | 65535 | 0.038011 | 0.090399 | -0.10053 |
| Fp1-Pz | -1.03916 | 0.150428 | 0.303748 | 65535 | 0.008942 | 0.200471 | -0.18972 |
| Fp1-F9 | -0.87043 | 0.192918 | 0.371676 | 65535 | 0.018018 | 0.143099 | -0.15892 |
| Fp1-F10 | -0.44202 | 0.329641 | 0.480727 | 65535 | 0.031555 | 0.069417 | -0.0807 |
| Fp2-F3 | 0.518264 | 0.697378 | 0.809112 | 65535 | 0.053073 | 0.072172 | 0.094622 |
| Fp2-F4 | -0.19985 | 0.420971 | 0.545632 | 65535 | 0.053334 | 0.054394 | -0.03649 |
| Fp2-C3 | -2.35843 | 0.009998 | 0.057476 | 65535 | -0.01634 | 0.976837 | -0.43059 |
| Fp2-C4 | 1.29545 | 0.901153 | 0.970473 | 65535 | 0.054867 | 0.192223 | 0.236515 |
| Fp2-P3 | -0.61703 | 0.269203 | 0.431547 | 65535 | 0.015354 | 0.092454 | -0.11265 |
| Fp2-P4 | -1.05492 | 0.146807 | 0.302251 | 65535 | 0.008383 | 0.183155 | -0.1926 |
| Fp2-O1 | -2.71545 | 0.003806 | 0.038064 | 65535 | -0.02503 | 0.893858 | -0.49577 |
| Fp2-O2 | -3.35819 | 0.000528 | 0.012518 | 65535 | -0.04178 | 0.998774 | -0.61312 |
| Fp2-F7 | -0.16346 | 0.435219 | 0.557292 | 65535 | 0.060992 | 0.052651 | -0.02984 |
| Fp2-F8 | -1.33077 | 0.092916 | 0.214422 | 65535 | 0.014326 | 0.247827 | -0.24296 |
| Fp2-T3 | -0.67414 | 0.25077 | 0.414302 | 65535 | 0.029928 | 0.114687 | -0.12308 |
| Fp2-T4 | -0.43162 | 0.333402 | 0.482859 | 65535 | 0.039167 | 0.068917 | -0.0788 |
| Fp2-T5 | -0.80989 | 0.209818 | 0.379378 | 65535 | 0.015329 | 0.110559 | -0.14786 |
| Fp2-T6 | -0.82484 | 0.205564 | 0.379301 | 65535 | 0.021753 | 0.133938 | -0.15059 |
| Fp2-Fz | -0.84973 | 0.198598 | 0.372372 | 65535 | 0.023108 | 0.128836 | -0.15514 |
| Fp2-Cz | -1.59632 | 0.056546 | 0.162666 | 65535 | 0.00212 | 0.430802 | -0.29145 |
| Fp2-Pz | -0.74663 | 0.228387 | 0.403035 | 65535 | 0.016634 | 0.130865 | -0.13631 |
| Fp2-F9 | -0.81968 | 0.207026 | 0.379301 | 65535 | 0.021927 | 0.116626 | -0.14965 |
| Fp2-F10 | 1.138983 | 0.871491 | 0.948255 | 65535 | 0.062989 | 0.16328 | 0.207949 |
| F3-F4 | 2.479589 | 0.992717 | 0.997467 | 65535 | 0.0904 | 0.450247 | 0.452709 |
| F3-C3 | -0.10143 | 0.459692 | 0.581023 | 65535 | 0.038204 | 0.051297 | -0.01852 |
| F3-C4 | 1.8099 | 0.963572 | 0.991912 | 65535 | 0.036347 | 0.300133 | 0.330441 |
| F3-P3 | -1.0478 | 0.148437 | 0.302638 | 65535 | 0.004958 | 0.164448 | -0.1913 |
| F3-P4 | -0.38498 | 0.350472 | 0.497292 | 65535 | 0.009083 | 0.06429 | -0.07029 |
| F3-O1 | 0.124308 | 0.549359 | 0.670729 | 65535 | 0.018265 | 0.051543 | 0.022696 |
| F3-O2 | -0.02203 | 0.49123 | 0.614038 | 65535 | 0.015543 | 0.050058 | -0.00402 |
| F3-F7 | -0.47246 | 0.318734 | 0.468072 | 65535 | 0.044149 | 0.079787 | -0.08626 |
| F3-F8 | 2.240027 | 0.986519 | 0.996005 | 65535 | 0.092468 | 0.482433 | 0.408971 |
| F3-T3 | -3.35356 | 0.000536 | 0.012518 | 65535 | -0.03783 | 0.99749 | -0.61227 |
| F3-T4 | -1.4007 | 0.081964 | 0.191442 | 65535 | 0.003745 | 0.257939 | -0.25573 |
| F3-T5 | -1.99142 | 0.024372 | 0.104453 | 65535 | -0.00547 | 0.64047 | -0.36358 |
| F3-T6 | -2.04289 | 0.021645 | 0.098412 | 65535 | -0.00366 | 0.487853 | -0.37298 |
| F3-Fz | 2.236354 | 0.986396 | 0.996005 | 65535 | 0.091494 | 0.483314 | 0.408301 |
| F3-Cz | -0.47577 | 0.31756 | 0.468072 | 65535 | 0.02428 | 0.083713 | -0.08686 |
| F3-Pz | -2.13596 | 0.017375 | 0.08438 | 65535 | -0.00454 | 0.93312 | -0.38997 |
| F3-F9 | -2.31902 | 0.011057 | 0.058397 | 65535 | -0.0137 | 0.631232 | -0.42339 |
| F3-F10 | -4.51102 | 7.67E-06 | 0.00161 | 65535 | -0.05793 | 0.999997 | -0.8236 |
| F4-C3 | -0.55366 | 0.29043 | 0.450045 | 65535 | 0.014762 | 0.080779 | -0.10108 |
| F4-C4 | 1.007301 | 0.842074 | 0.930714 | 65535 | 0.061695 | 0.13891 | 0.183907 |
| F4-P3 | -1.78205 | 0.038656 | 0.134077 | 65535 | -0.00077 | 0.672444 | -0.32536 |
| F4-P4 | -1.51128 | 0.066695 | 0.176235 | 65535 | 0.000962 | 0.357262 | -0.27592 |
| F4-O1 | 0.421891 | 0.663064 | 0.782266 | 65535 | 0.026868 | 0.064982 | 0.077026 |
| F4-O2 | -0.70332 | 0.24162 | 0.414302 | 65535 | 0.011898 | 0.101609 | -0.12841 |
| F4-F7 | 0.790672 | 0.784639 | 0.885883 | 65535 | 0.063377 | 0.109384 | 0.144356 |
| F4-F8 | 0.972749 | 0.833666 | 0.926296 | 65535 | 0.092235 | 0.15687 | 0.177599 |
| F4-T3 | -0.66409 | 0.253964 | 0.414302 | 65535 | 0.017216 | 0.097297 | -0.12125 |
| F4-T4 | -0.90753 | 0.182987 | 0.359134 | 65535 | 0.01421 | 0.143666 | -0.16569 |
| F4-T5 | -2.3797 | 0.009465 | 0.057476 | 65535 | -0.0064 | 0.969139 | -0.43447 |
| F4-T6 | -0.80448 | 0.211368 | 0.379378 | 65535 | 0.011094 | 0.12673 | -0.14688 |
| F4-Fz | 1.540383 | 0.936928 | 0.985287 | 65535 | 0.096123 | 0.28066 | 0.281234 |
| F4-Cz | 1.597688 | 0.943607 | 0.985287 | 65535 | 0.079046 | 0.238018 | 0.291697 |
| F4-Pz | -1.62892 | 0.052998 | 0.154578 | 65535 | 0.000306 | 0.609779 | -0.2974 |
| F4-F9 | -2.36595 | 0.009807 | 0.057476 | 65535 | -0.0118 | 0.759263 | -0.43196 |
| F4-F10 | -2.48039 | 0.007267 | 0.052392 | 65535 | -0.01071 | 0.803771 | -0.45286 |
| C3-C4 | 0.205701 | 0.581311 | 0.701582 | 65535 | 0.023225 | 0.05326 | 0.037556 |
| C3-P3 | -0.85199 | 0.197972 | 0.372372 | 65535 | 0.017282 | 0.114169 | -0.15555 |
| C3-P4 | -3.06989 | 0.001329 | 0.017823 | 65535 | -0.01416 | 1 | -0.56048 |
| C3-O1 | -3.48104 | 0.00035 | 0.010515 | 65535 | -0.03013 | 0.999995 | -0.63555 |
| C3-O2 | -3.69804 | 0.000166 | 0.006955 | 65535 | -0.02104 | 1 | -0.67517 |
| C3-F7 | -0.3759 | 0.353834 | 0.498692 | 65535 | 0.023978 | 0.068496 | -0.06863 |
| C3-F8 | -1.42354 | 0.078609 | 0.189745 | 65535 | 0.004264 | 0.386645 | -0.2599 |
| C3-T3 | -1.75911 | 0.040575 | 0.135576 | 65535 | -0.00199 | 0.534551 | -0.32117 |
| C3-T4 | -1.4807 | 0.070676 | 0.17882 | 65535 | 0.002225 | 0.358468 | -0.27034 |
| C3-T5 | -0.34849 | 0.364046 | 0.506691 | 65535 | 0.012288 | 0.061814 | -0.06363 |
| C3-T6 | -2.88372 | 0.002336 | 0.025821 | 65535 | -0.01411 | 0.997507 | -0.52649 |
| C3-Fz | 0.479177 | 0.68365 | 0.802048 | 65535 | 0.032419 | 0.073857 | 0.087486 |
| C3-Cz | -3.06289 | 0.001358 | 0.017823 | 65535 | -0.03497 | 0.999524 | -0.55921 |
| C3-Pz | -3.99418 | 5.67E-05 | 0.003966 | 65535 | -0.02235 | 0.999982 | -0.72923 |
| C3-F9 | -0.09547 | 0.462051 | 0.581023 | 65535 | 0.027249 | 0.050764 | -0.01743 |
| C3-F10 | -1.72733 | 0.043362 | 0.139012 | 65535 | -0.00096 | 0.650268 | -0.31537 |
| C4-P3 | -0.22957 | 0.409413 | 0.534016 | 65535 | 0.010053 | 0.054729 | -0.04191 |
| C4-P4 | -0.70461 | 0.241221 | 0.414302 | 65535 | 0.018898 | 0.096042 | -0.12864 |
| C4-O1 | -0.50843 | 0.30605 | 0.462378 | 65535 | 0.007638 | 0.083841 | -0.09283 |
| C4-O2 | -1.00729 | 0.157929 | 0.315858 | 65535 | 0.004819 | 0.205646 | -0.1839 |
| C4-F7 | 1.725402 | 0.956464 | 0.989445 | 65535 | 0.040153 | 0.265002 | 0.315014 |
| C4-F8 | -0.681 | 0.248602 | 0.414302 | 65535 | 0.017025 | 0.094116 | -0.12433 |
| C4-T3 | 1.430478 | 0.922389 | 0.982512 | 65535 | 0.038442 | 0.187881 | 0.261168 |
| C4-T4 | -1.99283 | 0.024294 | 0.104453 | 65535 | -0.00498 | 0.62742 | -0.36384 |
| C4-T5 | 0.643273 | 0.739353 | 0.843826 | 65535 | 0.018225 | 0.103363 | 0.117445 |
| C4-T6 | 1.598554 | 0.943703 | 0.985287 | 65535 | 0.035231 | 0.251241 | 0.291855 |
| C4-Fz | 1.132689 | 0.870179 | 0.948255 | 65535 | 0.043136 | 0.171509 | 0.2068 |
| C4-Cz | 2.030373 | 0.977717 | 0.996005 | 65535 | 0.098394 | 0.329593 | 0.370694 |
| C4-Pz | -0.53487 | 0.296871 | 0.45456 | 65535 | 0.016667 | 0.084834 | -0.09765 |
| C4-F9 | -1.93522 | 0.027678 | 0.111776 | 65535 | -0.00309 | 0.61763 | -0.35332 |
| C4-F10 | 0.883599 | 0.810645 | 0.91035 | 65535 | 0.019345 | 0.112208 | 0.161322 |
| P3-P4 | 0.613786 | 0.729731 | 0.837396 | 65535 | 0.022383 | 0.078993 | 0.112061 |
| P3-O1 | -1.64393 | 0.051426 | 0.154578 | 65535 | 0.000247 | 0.596026 | -0.30014 |
| P3-O2 | -1.62968 | 0.052918 | 0.154578 | 65535 | 0.000252 | 0.567432 | -0.29754 |
| P3-F7 | -1.11124 | 0.134363 | 0.285012 | 65535 | 0.006128 | 0.21983 | -0.20288 |
| P3-F8 | -0.89673 | 0.185844 | 0.361363 | 65535 | 0.007405 | 0.137085 | -0.16372 |
| P3-T3 | -1.40679 | 0.08106 | 0.191442 | 65535 | 0.003928 | 0.34594 | -0.25684 |
| P3-T4 | -1.26769 | 0.103703 | 0.234168 | 65535 | 0.002816 | 0.329096 | -0.23145 |
| P3-T5 | -0.19334 | 0.423514 | 0.545632 | 65535 | 0.021665 | 0.054052 | -0.0353 |
| P3-T6 | 0.58759 | 0.721035 | 0.831963 | 65535 | 0.024281 | 0.089929 | 0.107279 |
| P3-Fz | -1.63621 | 0.05223 | 0.154578 | 65535 | 0.00021 | 0.607571 | -0.29873 |
| P3-Cz | -1.06664 | 0.144155 | 0.299729 | 65535 | 0.007594 | 0.276769 | -0.19474 |
| P3-Pz | -0.57963 | 0.281635 | 0.442761 | 65535 | 0.018694 | 0.093582 | -0.10583 |
| P3-F9 | -1.44644 | 0.075352 | 0.183999 | 65535 | 0.001781 | 0.475243 | -0.26408 |
| P3-F10 | -0.34772 | 0.364335 | 0.506691 | 65535 | 0.009368 | 0.061553 | -0.06348 |
| P4-O1 | -2.21046 | 0.014501 | 0.074273 | 65535 | -0.00568 | 0.959004 | -0.40357 |
| P4-O2 | -1.40015 | 0.082047 | 0.191442 | 65535 | 0.003066 | 0.48185 | -0.25563 |
| P4-F7 | -2.64428 | 0.00465 | 0.044386 | 65535 | -0.01007 | 0.873972 | -0.48278 |
| P4-F8 | -1.50782 | 0.067137 | 0.176235 | 65535 | 0.001876 | 0.382518 | -0.27529 |
| P4-T3 | -1.72371 | 0.043689 | 0.139012 | 65535 | -0.0009 | 0.468605 | -0.31471 |
| P4-T4 | -0.62217 | 0.267516 | 0.431547 | 65535 | 0.011205 | 0.089401 | -0.11359 |
| P4-T5 | 0.238449 | 0.594027 | 0.704778 | 65535 | 0.019478 | 0.055403 | 0.043535 |
| P4-T6 | -0.28618 | 0.387622 | 0.515194 | 65535 | 0.023573 | 0.059766 | -0.05225 |
| P4-Fz | -1.85578 | 0.03299 | 0.123712 | 65535 | -0.00149 | 0.696319 | -0.33882 |
| P4-Cz | -1.77853 | 0.038946 | 0.134077 | 65535 | -0.00197 | 0.409987 | -0.32471 |
| P4-Pz | -1.15426 | 0.125362 | 0.271403 | 65535 | 0.008823 | 0.255594 | -0.21074 |
| P4-F9 | -1.9736 | 0.025382 | 0.106604 | 65535 | -0.00184 | 0.641571 | -0.36033 |
| P4-F10 | -0.66241 | 0.2545 | 0.414302 | 65535 | 0.010152 | 0.094368 | -0.12094 |
| O1-O2 | -2.57551 | 0.005622 | 0.048057 | 65535 | -0.026 | 0.833477 | -0.47022 |
| O1-F7 | -1.8947 | 0.03029 | 0.120015 | 65535 | -0.00457 | 0.606508 | -0.34592 |
| O1-F8 | -1.88499 | 0.030946 | 0.120344 | 65535 | -0.00363 | 0.640436 | -0.34415 |
| O1-T3 | -3.30107 | 0.000637 | 0.013382 | 65535 | -0.03868 | 0.999923 | -0.60269 |
| O1-T4 | -1.75047 | 0.041318 | 0.135576 | 65535 | -0.00127 | 0.713807 | -0.31959 |
| O1-T5 | -0.69847 | 0.243127 | 0.414302 | 65535 | 0.012986 | 0.166623 | -0.12752 |
| O1-T6 | -2.4858 | 0.007163 | 0.052392 | 65535 | -0.0099 | 0.857867 | -0.45384 |
| O1-Fz | -1.19597 | 0.117053 | 0.256054 | 65535 | 0.003964 | 0.204193 | -0.21835 |
| O1-Cz | -4.28183 | 1.9E-05 | 0.001993 | 65535 | -0.05225 | 1 | -0.78175 |
| O1-Pz | -2.91611 | 0.002122 | 0.024752 | 65535 | -0.01611 | 0.987132 | -0.53241 |
| O1-F9 | -0.31565 | 0.376413 | 0.51329 | 65535 | 0.01408 | 0.06166 | -0.05763 |
| O1-F10 | -0.49093 | 0.312194 | 0.468072 | 65535 | 0.010984 | 0.07584 | -0.08963 |
| O2-F7 | -1.5346 | 0.063779 | 0.173944 | 65535 | 0.001991 | 0.377098 | -0.28018 |
| O2-F8 | -2.12869 | 0.01768 | 0.08438 | 65535 | -0.00939 | 0.586971 | -0.38864 |
| O2-T3 | -3.09044 | 0.001247 | 0.017823 | 65535 | -0.01922 | 0.998815 | -0.56423 |
| O2-T4 | 0.227554 | 0.589806 | 0.703746 | 65535 | 0.023173 | 0.055546 | 0.041545 |
| O2-T5 | -3.01793 | 0.001559 | 0.019263 | 65535 | -0.01467 | 0.975503 | -0.551 |
| O2-T6 | -0.52955 | 0.298711 | 0.45456 | 65535 | 0.013235 | 0.083713 | -0.09668 |
| O2-Fz | -1.31044 | 0.096296 | 0.219806 | 65535 | 0.002839 | 0.280229 | -0.23925 |
| O2-Cz | -3.75147 | 0.000137 | 0.006955 | 65535 | -0.04283 | 1 | -0.68492 |
| O2-Pz | -1.79036 | 0.03798 | 0.134077 | 65535 | -0.00191 | 0.334734 | -0.32687 |
| O2-F9 | -0.84982 | 0.198574 | 0.372372 | 65535 | 0.009113 | 0.154972 | -0.15515 |
| O2-F10 | -0.38738 | 0.349585 | 0.497292 | 65535 | 0.012279 | 0.066107 | -0.07073 |
| F7-F8 | 1.25413 | 0.893863 | 0.967584 | 65535 | 0.143921 | 0.208212 | 0.228972 |
| F7-T3 | -1.81093 | 0.036348 | 0.133915 | 65535 | -0.00665 | 0.49753 | -0.33063 |
| F7-T4 | -1.13894 | 0.128518 | 0.275396 | 65535 | 0.014764 | 0.2181 | -0.20794 |
| F7-T5 | -1.24947 | 0.106983 | 0.239005 | 65535 | 0.010727 | 0.260598 | -0.22812 |
| F7-T6 | -2.57372 | 0.005649 | 0.048057 | 65535 | -0.02173 | 0.891781 | -0.4699 |
| F7-Fz | -0.40314 | 0.343788 | 0.494489 | 65535 | 0.018793 | 0.066041 | -0.0736 |
| F7-Cz | -0.7672 | 0.222249 | 0.395527 | 65535 | 0.020682 | 0.112252 | -0.14007 |
| F7-Pz | -2.31668 | 0.011123 | 0.058397 | 65535 | -0.00691 | 0.756543 | -0.42297 |
| F7-F9 | -0.32455 | 0.373048 | 0.51329 | 65535 | 0.04157 | 0.061564 | -0.05925 |
| F7-F10 | 0.122557 | 0.548667 | 0.670729 | 65535 | 0.056705 | 0.051875 | 0.022376 |
| F8-T3 | 0.0606 | 0.52411 | 0.64743 | 65535 | 0.045601 | 0.050319 | 0.011064 |
| F8-T4 | -2.74559 | 0.003493 | 0.036678 | 65535 | -0.04029 | 0.771449 | -0.50127 |
| F8-T5 | -1.46818 | 0.072358 | 0.180734 | 65535 | 0.003171 | 0.285749 | -0.26805 |
| F8-T6 | -1.95542 | 0.026449 | 0.108908 | 65535 | -0.00709 | 0.55485 | -0.35701 |
| F8-Fz | -0.26974 | 0.393915 | 0.520265 | 65535 | 0.018868 | 0.057635 | -0.04925 |
| F8-Cz | -0.47523 | 0.317751 | 0.468072 | 65535 | 0.028293 | 0.074783 | -0.08676 |
| F8-Pz | -1.0991 | 0.136981 | 0.287661 | 65535 | 0.007006 | 0.308934 | -0.20067 |
| F8-F9 | -2.5408 | 0.006179 | 0.048057 | 65535 | -0.0287 | 0.702231 | -0.46389 |
| F8-F10 | 1.458848 | 0.926368 | 0.982512 | 65535 | 0.072692 | 0.237936 | 0.266348 |
| T3-T4 | 1.359988 | 0.911787 | 0.976914 | 65535 | 0.080676 | 0.227424 | 0.248298 |
| T3-T5 | 0.0136 | 0.505414 | 0.628029 | 65535 | 0.051875 | 0.050021 | 0.002483 |
| T3-T6 | -0.68705 | 0.246698 | 0.414302 | 65535 | 0.019741 | 0.114397 | -0.12544 |
| T3-Fz | -3.58532 | 0.000245 | 0.008589 | 65535 | -0.02025 | 0.999881 | -0.65459 |
| T3-Cz | -1.55164 | 0.061713 | 0.172795 | 65535 | 0.002679 | 0.562856 | -0.28329 |
| T3-Pz | -2.4536 | 0.007802 | 0.052392 | 65535 | -0.01082 | 0.999854 | -0.44796 |
| T3-F9 | 1.133312 | 0.87031 | 0.948255 | 65535 | 0.095646 | 0.200896 | 0.206913 |
| T3-F10 | 0.958168 | 0.830031 | 0.926296 | 65535 | 0.098214 | 0.193314 | 0.174937 |
| T4-T5 | -2.1416 | 0.017141 | 0.08438 | 65535 | -0.01033 | 0.610343 | -0.391 |
| T4-T6 | -0.30215 | 0.381537 | 0.514267 | 65535 | 0.040954 | 0.060499 | -0.05516 |
| T4-Fz | -0.30086 | 0.382027 | 0.514267 | 65535 | 0.013078 | 0.057471 | -0.05493 |
| T4-Cz | -1.50049 | 0.06808 | 0.176503 | 65535 | 0.001834 | 0.758879 | -0.27395 |
| T4-Pz | -2.34691 | 0.010298 | 0.057476 | 65535 | -0.00545 | 0.95076 | -0.42849 |
| T4-F9 | -3.25903 | 0.00073 | 0.013944 | 65535 | -0.05088 | 0.822276 | -0.59502 |
| T4-F10 | -0.28906 | 0.386519 | 0.515194 | 65535 | 0.039025 | 0.058168 | -0.05278 |
| T5-T6 | -1.71125 | 0.044831 | 0.140515 | 65535 | -0.00069 | 0.485156 | -0.31243 |
| T5-Fz | -1.53525 | 0.063699 | 0.173944 | 65535 | 0.000679 | 0.512154 | -0.2803 |
| T5-Cz | -1.46233 | 0.073154 | 0.180734 | 65535 | 0.001739 | 0.30803 | -0.26698 |
| T5-Pz | -2.34305 | 0.0104 | 0.057476 | 65535 | -0.01042 | 0.999829 | -0.42778 |
| T5-F9 | -2.03539 | 0.022026 | 0.098412 | 65535 | -0.01025 | 0.579533 | -0.37161 |
| T5-F10 | -0.73105 | 0.2331 | 0.407924 | 65535 | 0.026355 | 0.10912 | -0.13347 |
| T6-Fz | -0.57712 | 0.282479 | 0.442761 | 65535 | 0.007899 | 0.091749 | -0.10537 |
| T6-Cz | -2.46079 | 0.007655 | 0.052392 | 65535 | -0.01401 | 0.957953 | -0.44928 |
| T6-Pz | -2.55521 | 0.005942 | 0.048057 | 65535 | -0.00926 | 0.996836 | -0.46652 |
| T6-F9 | -1.48673 | 0.069877 | 0.17882 | 65535 | 0.004352 | 0.302168 | -0.27144 |
| T6-F10 | -1.52709 | 0.064708 | 0.174213 | 65535 | 0.00342 | 0.358263 | -0.27881 |
| Fz-Cz | -3.22553 | 0.000814 | 0.014237 | 65535 | -0.03481 | 0.997512 | -0.5889 |
| Fz-Pz | -3.08583 | 0.001265 | 0.017823 | 65535 | -0.01076 | 0.996001 | -0.56339 |
| Fz-F9 | -2.54675 | 0.00608 | 0.048057 | 65535 | -0.00565 | 0.937668 | -0.46497 |
| Fz-F10 | -1.56121 | 0.060576 | 0.171906 | 65535 | 0.000655 | 0.406945 | -0.28504 |
| Cz-Pz | -0.14467 | 0.442607 | 0.563319 | 65535 | 0.031966 | 0.052108 | -0.02641 |
| Cz-F9 | -2.09616 | 0.019102 | 0.089144 | 65535 | -0.00464 | 0.993537 | -0.38271 |
| Cz-F10 | -0.81726 | 0.207712 | 0.379301 | 65535 | 0.006312 | 0.20639 | -0.14921 |
| Pz-F9 | -1.78562 | 0.038365 | 0.134077 | 65535 | -0.00079 | 0.749026 | -0.32601 |
| Pz-F10 | -1.75153 | 0.041226 | 0.135576 | 65535 | -0.00056 | 0.804248 | -0.31978 |
| F9-F10 | -1.21483 | 0.113429 | 0.250738 | 65535 | 0.014111 | 0.216022 | -0.2218 |

Table S3i: gamma_coh_HC_PT

| channel | tvalue | pvalue | fdr_pvalue | CI_low | CI_high | statistical power | effect size |
| --- | --- | --- | --- | --- | --- | --- | --- |
| Fp1-Fp2 | 0.772029 | 0.22082 | 0.99999 | -0.04335 | 65535 | 0.112248 | 0.140952 |
| Fp1-F3 | 0.70096 | 0.242354 | 0.99999 | -0.0304 | 65535 | 0.108048 | 0.127977 |
| Fp1-F4 | 1.695166 | 0.046341 | 0.593725 | 0.000667 | 65535 | 0.298917 | 0.309494 |
| Fp1-C3 | 0.260365 | 0.397518 | 0.99999 | -0.03159 | 65535 | 0.059099 | 0.047536 |
| Fp1-C4 | 1.334411 | 0.092319 | 0.842916 | -0.00471 | 65535 | 0.221466 | 0.243629 |
| Fp1-P3 | 1.124516 | 0.131538 | 0.850337 | -0.00544 | 65535 | 0.176329 | 0.205308 |
| Fp1-P4 | -1.06757 | 0.856052 | 0.99999 | -0.02235 | 65535 | 0.234735 | -0.19491 |
| Fp1-O1 | -2.66543 | 0.995617 | 0.99999 | -0.09511 | 65535 | 0.912738 | -0.48664 |
| Fp1-O2 | -2.30449 | 0.988528 | 0.99999 | -0.08126 | 65535 | 0.890676 | -0.42074 |
| Fp1-F7 | 1.214918 | 0.113411 | 0.850337 | -0.02031 | 65535 | 0.228063 | 0.221813 |
| Fp1-F8 | 1.692854 | 0.046561 | 0.593725 | 0.00127 | 65535 | 0.319878 | 0.309071 |
| Fp1-T3 | 1.089362 | 0.139107 | 0.850337 | -0.02326 | 65535 | 0.173716 | 0.198889 |
| Fp1-T4 | -0.24142 | 0.595175 | 0.99999 | -0.0476 | 65535 | 0.055242 | -0.04408 |
| Fp1-T5 | -0.83729 | 0.797938 | 0.99999 | -0.06163 | 65535 | 0.142682 | -0.15287 |
| Fp1-T6 | -0.49503 | 0.689251 | 0.99999 | -0.03947 | 65535 | 0.081558 | -0.09038 |
| Fp1-Fz | 0.868258 | 0.193508 | 0.99999 | -0.01373 | 65535 | 0.123165 | 0.158521 |
| Fp1-Cz | 0.178105 | 0.429473 | 0.99999 | -0.03869 | 65535 | 0.054642 | 0.032517 |
| Fp1-Pz | -1.38858 | 0.916213 | 0.99999 | -0.03136 | 65535 | 0.499772 | -0.25352 |
| Fp1-F9 | 0.901467 | 0.184588 | 0.99999 | -0.01668 | 65535 | 0.125665 | 0.164584 |
| Fp1-F10 | 2.136794 | 0.01734 | 0.556582 | 0.011613 | 65535 | 0.482095 | 0.390123 |
| Fp2-F3 | 1.427052 | 0.078103 | 0.745528 | -0.00495 | 65535 | 0.261212 | 0.260543 |
| Fp2-F4 | 0.794786 | 0.214167 | 0.99999 | -0.02835 | 65535 | 0.116491 | 0.145107 |
| Fp2-C3 | -0.31609 | 0.623755 | 0.99999 | -0.03648 | 65535 | 0.064091 | -0.05771 |
| Fp2-C4 | -1.02907 | 0.847224 | 0.99999 | -0.04925 | 65535 | 0.20378 | -0.18788 |
| Fp2-P3 | 0.747723 | 0.228057 | 0.99999 | -0.00874 | 65535 | 0.104101 | 0.136515 |
| Fp2-P4 | 0.142909 | 0.443303 | 0.99999 | -0.01034 | 65535 | 0.051965 | 0.026091 |
| Fp2-O1 | -2.94387 | 0.998048 | 0.99999 | -0.09614 | 65535 | 0.989654 | -0.53747 |
| Fp2-O2 | -3.59993 | 0.999767 | 0.99999 | -0.13235 | 65535 | 0.999927 | -0.65725 |
| Fp2-F7 | 1.217628 | 0.112897 | 0.850337 | -0.01647 | 65535 | 0.192821 | 0.222307 |
| Fp2-F8 | -0.2532 | 0.599725 | 0.99999 | -0.08734 | 65535 | 0.056881 | -0.04623 |
| Fp2-T3 | 0.50817 | 0.306141 | 0.99999 | -0.03395 | 65535 | 0.083023 | 0.092779 |
| Fp2-T4 | 0.215663 | 0.414812 | 0.99999 | -0.04438 | 65535 | 0.054849 | 0.039375 |
| Fp2-T5 | 0.055191 | 0.47804 | 0.99999 | -0.02547 | 65535 | 0.050262 | 0.010076 |
| Fp2-T6 | -1.6457 | 0.948757 | 0.99999 | -0.07637 | 65535 | 0.494171 | -0.30046 |
| Fp2-Fz | 0.507822 | 0.306263 | 0.99999 | -0.02463 | 65535 | 0.07684 | 0.092715 |
| Fp2-Cz | -0.39712 | 0.654002 | 0.99999 | -0.05696 | 65535 | 0.078145 | -0.0725 |
| Fp2-Pz | -1.66438 | 0.950655 | 0.99999 | -0.03508 | 65535 | 0.693109 | -0.30387 |
| Fp2-F9 | 0.149134 | 0.440851 | 0.99999 | -0.04015 | 65535 | 0.052205 | 0.027228 |
| Fp2-F10 | 1.925418 | 0.028292 | 0.556582 | 0.006152 | 65535 | 0.414588 | 0.351532 |
| F3-F4 | 1.872523 | 0.031805 | 0.556582 | 0.004731 | 65535 | 0.270766 | 0.341874 |
| F3-C3 | -0.11864 | 0.547118 | 0.99999 | -0.05343 | 65535 | 0.051332 | -0.02166 |
| F3-C4 | 0.819068 | 0.207199 | 0.99999 | -0.00853 | 65535 | 0.114445 | 0.149541 |
| F3-P3 | -0.13724 | 0.554461 | 0.99999 | -0.01624 | 65535 | 0.052456 | -0.02506 |
| F3-P4 | 0.768532 | 0.221853 | 0.99999 | -0.00295 | 65535 | 0.095167 | 0.140314 |
| F3-O1 | -0.92409 | 0.821337 | 0.99999 | -0.0297 | 65535 | 0.184193 | -0.16871 |
| F3-O2 | -0.12095 | 0.548031 | 0.99999 | -0.01599 | 65535 | 0.051602 | -0.02208 |
| F3-F7 | 0.223966 | 0.411586 | 0.99999 | -0.04971 | 65535 | 0.056775 | 0.04089 |
| F3-F8 | 2.304444 | 0.011473 | 0.556582 | 0.014591 | 65535 | 0.485462 | 0.420732 |
| F3-T3 | 0.171575 | 0.432033 | 0.99999 | -0.02629 | 65535 | 0.053239 | 0.031325 |
| F3-T4 | 1.082238 | 0.140677 | 0.850337 | -0.00594 | 65535 | 0.130092 | 0.197589 |
| F3-T5 | -2.31216 | 0.988749 | 0.99999 | -0.06134 | 65535 | 0.951064 | -0.42214 |
| F3-T6 | 1.433108 | 0.077236 | 0.745528 | -0.00274 | 65535 | 0.177774 | 0.261649 |
| F3-Fz | 0.978011 | 0.165035 | 0.962704 | -0.01581 | 65535 | 0.141963 | 0.17856 |
| F3-Cz | -0.93334 | 0.823724 | 0.99999 | -0.04225 | 65535 | 0.256602 | -0.1704 |
| F3-Pz | -0.6171 | 0.730823 | 0.99999 | -0.01396 | 65535 | 0.121272 | -0.11267 |
| F3-F9 | -0.22718 | 0.589662 | 0.99999 | -0.03593 | 65535 | 0.05455 | -0.04148 |
| F3-F10 | -1.37141 | 0.913576 | 0.99999 | -0.04517 | 65535 | 0.314097 | -0.25038 |
| F4-C3 | 0.248829 | 0.401963 | 0.99999 | -0.01463 | 65535 | 0.055488 | 0.04543 |
| F4-C4 | -0.05472 | 0.521772 | 0.99999 | -0.03201 | 65535 | 0.0504 | -0.00999 |
| F4-P3 | 0.724589 | 0.235069 | 0.99999 | -0.00548 | 65535 | 0.098879 | 0.132291 |
| F4-P4 | 0.29712 | 0.383449 | 0.99999 | -0.00487 | 65535 | 0.059036 | 0.054246 |
| F4-O1 | 0.474015 | 0.318183 | 0.99999 | -0.00801 | 65535 | 0.067851 | 0.086543 |
| F4-O2 | -0.7592 | 0.775376 | 0.99999 | -0.031 | 65535 | 0.184375 | -0.13861 |
| F4-F7 | 1.884816 | 0.030957 | 0.556582 | 0.004265 | 65535 | 0.360107 | 0.344119 |
| F4-F8 | 2.069355 | 0.020348 | 0.556582 | 0.014048 | 65535 | 0.540342 | 0.377811 |
| F4-T3 | 1.11635 | 0.13327 | 0.850337 | -0.00748 | 65535 | 0.146345 | 0.203817 |
| F4-T4 | 1.15115 | 0.125999 | 0.850337 | -0.00831 | 65535 | 0.150971 | 0.21017 |
| F4-T5 | 0.315878 | 0.376327 | 0.99999 | -0.00497 | 65535 | 0.059728 | 0.057671 |
| F4-T6 | -1.91335 | 0.970937 | 0.99999 | -0.04203 | 65535 | 0.609799 | -0.34933 |
| F4-Fz | 1.576151 | 0.058834 | 0.624107 | -0.00193 | 65535 | 0.314426 | 0.287765 |
| F4-Cz | 1.756526 | 0.040796 | 0.593725 | 0.00139 | 65535 | 0.292689 | 0.320696 |
| F4-Pz | 0.16407 | 0.434978 | 0.99999 | -0.01024 | 65535 | 0.052505 | 0.029955 |
| F4-F9 | 0.023484 | 0.490652 | 0.99999 | -0.02213 | 65535 | 0.050064 | 0.004288 |
| F4-F10 | 0.045476 | 0.481902 | 0.99999 | -0.02026 | 65535 | 0.05018 | 0.008303 |
| C3-C4 | 0.848217 | 0.199017 | 0.99999 | -0.01235 | 65535 | 0.102671 | 0.154863 |
| C3-P3 | 0.524491 | 0.300461 | 0.99999 | -0.02125 | 65535 | 0.078731 | 0.095759 |
| C3-P4 | -2.35558 | 0.989928 | 0.99999 | -0.01771 | 65535 | 0.99966 | -0.43007 |
| C3-O1 | -3.16733 | 0.999021 | 0.99999 | -0.06404 | 65535 | 0.999989 | -0.57827 |
| C3-O2 | -3.92192 | 0.999926 | 0.99999 | -0.03913 | 65535 | 1 | -0.71604 |
| C3-F7 | 0.014181 | 0.494355 | 0.99999 | -0.0349 | 65535 | 0.050029 | 0.002589 |
| C3-F8 | 0.026292 | 0.489534 | 0.99999 | -0.02378 | 65535 | 0.050071 | 0.0048 |
| C3-T3 | -0.81941 | 0.792897 | 0.99999 | -0.06719 | 65535 | 0.147235 | -0.1496 |
| C3-T4 | 0.302576 | 0.381373 | 0.99999 | -0.01386 | 65535 | 0.058304 | 0.055242 |
| C3-T5 | -0.94394 | 0.826434 | 0.99999 | -0.0296 | 65535 | 0.231068 | -0.17234 |
| C3-T6 | -1.41188 | 0.919691 | 0.99999 | -0.01852 | 65535 | 0.399437 | -0.25777 |
| C3-Fz | 1.987181 | 0.024609 | 0.556582 | 0.004366 | 65535 | 0.373374 | 0.362808 |
| C3-Cz | -1.82688 | 0.964877 | 0.99999 | -0.06044 | 65535 | 0.843404 | -0.33354 |
| C3-Pz | -2.67846 | 0.995774 | 0.99999 | -0.04304 | 65535 | 0.981685 | -0.48902 |
| C3-F9 | 0.637516 | 0.262512 | 0.99999 | -0.02087 | 65535 | 0.083844 | 0.116394 |
| C3-F10 | -0.92817 | 0.822394 | 0.99999 | -0.03022 | 65535 | 0.188306 | -0.16946 |
| C4-P3 | -0.05022 | 0.519986 | 0.99999 | -0.00513 | 65535 | 0.050303 | -0.00917 |
| C4-P4 | -1.12787 | 0.869168 | 0.99999 | -0.03774 | 65535 | 0.264551 | -0.20592 |
| C4-O1 | -2.48826 | 0.992884 | 0.99999 | -0.02644 | 65535 | 0.891648 | -0.45429 |
| C4-O2 | -1.61409 | 0.945411 | 0.99999 | -0.02754 | 65535 | 0.392903 | -0.29469 |
| C4-F7 | 0.686057 | 0.247012 | 0.99999 | -0.01225 | 65535 | 0.092357 | 0.125256 |
| C4-F8 | -2.00056 | 0.976132 | 0.99999 | -0.07112 | 65535 | 0.658616 | -0.36525 |
| C4-T3 | 1.077521 | 0.141723 | 0.850337 | -0.00517 | 65535 | 0.144118 | 0.196727 |
| C4-T4 | -3.81124 | 0.999889 | 0.99999 | -0.14647 | 65535 | 0.997249 | -0.69583 |
| C4-T5 | 0.033209 | 0.486782 | 0.99999 | -0.00975 | 65535 | 0.050084 | 0.006063 |
| C4-T6 | -0.71195 | 0.761049 | 0.99999 | -0.02756 | 65535 | 0.100572 | -0.12998 |
| C4-Fz | 2.253465 | 0.013038 | 0.556582 | 0.005365 | 65535 | 0.429004 | 0.411425 |
| C4-Cz | 1.970887 | 0.025539 | 0.556582 | 0.00469 | 65535 | 0.40754 | 0.359833 |
| C4-Pz | -1.00862 | 0.84239 | 0.99999 | -0.03159 | 65535 | 0.187626 | -0.18415 |
| C4-F9 | -3.05865 | 0.998624 | 0.99999 | -0.06068 | 65535 | 0.958293 | -0.55843 |
| C4-F10 | 0.660611 | 0.255075 | 0.99999 | -0.01163 | 65535 | 0.088967 | 0.120611 |
| P3-P4 | -0.40299 | 0.656156 | 0.99999 | -0.00959 | 65535 | 0.075736 | -0.07357 |
| P3-O1 | -0.04369 | 0.517386 | 0.99999 | -0.02249 | 65535 | 0.050256 | -0.00798 |
| P3-O2 | 0.126745 | 0.449679 | 0.99999 | -0.01148 | 65535 | 0.05276 | 0.02314 |
| P3-F7 | 0.15461 | 0.438696 | 0.99999 | -0.01654 | 65535 | 0.05238 | 0.028228 |
| P3-F8 | 0.928513 | 0.177518 | 0.99999 | -0.00637 | 65535 | 0.1165 | 0.169522 |
| P3-T3 | -1.30764 | 0.903231 | 0.99999 | -0.07548 | 65535 | 0.43925 | -0.23874 |
| P3-T4 | 0.192143 | 0.42398 | 0.99999 | -0.01278 | 65535 | 0.05406 | 0.03508 |
| P3-T5 | -0.28904 | 0.613472 | 0.99999 | -0.03297 | 65535 | 0.071406 | -0.05277 |
| P3-T6 | 1.67732 | 0.048063 | 0.593725 | 0.000158 | 65535 | 0.27024 | 0.306235 |
| P3-Fz | 0.655069 | 0.256849 | 0.99999 | -0.00368 | 65535 | 0.088511 | 0.119599 |
| P3-Cz | -0.21809 | 0.586131 | 0.99999 | -0.01741 | 65535 | 0.061822 | -0.03982 |
| P3-Pz | 0.131851 | 0.447664 | 0.99999 | -0.01468 | 65535 | 0.052396 | 0.024072 |
| P3-F9 | 0.167345 | 0.433692 | 0.99999 | -0.01903 | 65535 | 0.053232 | 0.030553 |
| P3-F10 | 1.169722 | 0.122235 | 0.850337 | -0.00346 | 65535 | 0.162895 | 0.213561 |
| P4-O1 | 0.671793 | 0.251514 | 0.99999 | -0.0082 | 65535 | 0.087246 | 0.122652 |
| P4-O2 | -0.22608 | 0.589235 | 0.99999 | -0.01636 | 65535 | 0.061042 | -0.04128 |
| P4-F7 | 0.476606 | 0.317262 | 0.99999 | -0.0051 | 65535 | 0.068896 | 0.087016 |
| P4-F8 | -0.0016 | 0.500637 | 0.99999 | -0.01 | 65535 | 0.05 | -0.00029 |
| P4-T3 | 0.446072 | 0.328181 | 0.99999 | -0.01014 | 65535 | 0.068087 | 0.081441 |
| P4-T4 | -1.8452 | 0.966242 | 0.99999 | -0.07147 | 65535 | 0.658513 | -0.33689 |
| P4-T5 | -0.92652 | 0.821966 | 0.99999 | -0.01429 | 65535 | 0.247341 | -0.16916 |
| P4-T6 | 0.414389 | 0.339671 | 0.99999 | -0.01893 | 65535 | 0.067181 | 0.075656 |
| P4-Fz | -1.05098 | 0.852292 | 0.99999 | -0.01559 | 65535 | 0.345865 | -0.19188 |
| P4-Cz | 0.020023 | 0.492029 | 0.99999 | -0.01747 | 65535 | 0.050041 | 0.003656 |
| P4-Pz | -1.55068 | 0.938172 | 0.99999 | -0.02718 | 65535 | 0.319274 | -0.28311 |
| P4-F9 | 0.360987 | 0.359377 | 0.99999 | -0.00645 | 65535 | 0.066861 | 0.065907 |
| P4-F10 | -1.56008 | 0.939291 | 0.99999 | -0.03584 | 65535 | 0.381271 | -0.28483 |
| O1-O2 | -1.83828 | 0.965732 | 0.99999 | -0.08304 | 65535 | 0.608073 | -0.33562 |
| O1-F7 | -4.19222 | 0.999973 | 0.99999 | -0.14773 | 65535 | 0.999946 | -0.76539 |
| O1-F8 | -1.88821 | 0.969273 | 0.99999 | -0.07237 | 65535 | 0.775906 | -0.34474 |
| O1-T3 | -2.7855 | 0.996886 | 0.99999 | -0.12378 | 65535 | 0.998288 | -0.50856 |
| O1-T4 | -2.25847 | 0.987123 | 0.99999 | -0.04932 | 65535 | 0.796303 | -0.41234 |
| O1-T5 | -0.96071 | 0.83067 | 0.99999 | -0.03973 | 65535 | 0.286645 | -0.1754 |
| O1-T6 | -1.11608 | 0.866671 | 0.99999 | -0.03833 | 65535 | 0.368178 | -0.20377 |
| O1-Fz | 0.211671 | 0.416364 | 0.99999 | -0.00858 | 65535 | 0.055162 | 0.038646 |
| O1-Cz | -2.92313 | 0.997922 | 0.99999 | -0.10658 | 65535 | 0.999998 | -0.53369 |
| O1-Pz | -2.4027 | 0.991083 | 0.99999 | -0.03019 | 65535 | 0.740252 | -0.43867 |
| O1-F9 | -2.02031 | 0.977192 | 0.99999 | -0.06234 | 65535 | 0.901941 | -0.36886 |
| O1-F10 | -0.14734 | 0.558441 | 0.99999 | -0.02599 | 65535 | 0.053405 | -0.0269 |
| O2-F7 | -2.37657 | 0.990458 | 0.99999 | -0.08253 | 65535 | 0.783648 | -0.4339 |
| O2-F8 | -3.6232 | 0.999785 | 0.99999 | -0.13377 | 65535 | 0.988758 | -0.6615 |
| O2-T3 | -3.1729 | 0.999038 | 0.99999 | -0.09399 | 65535 | 0.999799 | -0.57929 |
| O2-T4 | -0.7498 | 0.772566 | 0.99999 | -0.03061 | 65535 | 0.120499 | -0.13689 |
| O2-T5 | -1.72295 | 0.956241 | 0.99999 | -0.03318 | 65535 | 0.657532 | -0.31457 |
| O2-T6 | -1.38944 | 0.916343 | 0.99999 | -0.05183 | 65535 | 0.401804 | -0.25368 |
| O2-Fz | -1.68565 | 0.952747 | 0.99999 | -0.01911 | 65535 | 0.810673 | -0.30776 |
| O2-Cz | -3.19785 | 0.999111 | 0.99999 | -0.10164 | 65535 | 1 | -0.58384 |
| O2-Pz | -1.06526 | 0.855533 | 0.99999 | -0.02824 | 65535 | 0.171852 | -0.19449 |
| O2-F9 | -1.61608 | 0.945626 | 0.99999 | -0.04763 | 65535 | 0.451231 | -0.29505 |
| O2-F10 | -0.7449 | 0.771095 | 0.99999 | -0.03529 | 65535 | 0.129209 | -0.136 |
| F7-F8 | 1.570925 | 0.059439 | 0.624107 | -0.0043 | 65535 | 0.302395 | 0.28681 |
| F7-T3 | -0.52799 | 0.70075 | 0.99999 | -0.11808 | 65535 | 0.092231 | -0.0964 |
| F7-T4 | -0.07293 | 0.529009 | 0.99999 | -0.04411 | 65535 | 0.050643 | -0.01332 |
| F7-T5 | -1.81211 | 0.963744 | 0.99999 | -0.08071 | 65535 | 0.47505 | -0.33085 |
| F7-T6 | -2.25581 | 0.987038 | 0.99999 | -0.08879 | 65535 | 0.867398 | -0.41185 |
| F7-Fz | -1.12891 | 0.869387 | 0.99999 | -0.03919 | 65535 | 0.206034 | -0.20611 |
| F7-Cz | -0.74326 | 0.770599 | 0.99999 | -0.05935 | 65535 | 0.129843 | -0.1357 |
| F7-Pz | -1.50045 | 0.931916 | 0.99999 | -0.02433 | 65535 | 0.445597 | -0.27394 |
| F7-F9 | -0.23275 | 0.591821 | 0.99999 | -0.066 | 65535 | 0.056967 | -0.04249 |
| F7-F10 | 1.29434 | 0.099038 | 0.850337 | -0.01126 | 65535 | 0.271982 | 0.236313 |
| F8-T3 | 0.593164 | 0.277103 | 0.99999 | -0.03234 | 65535 | 0.088084 | 0.108297 |
| F8-T4 | -0.00629 | 0.502505 | 0.99999 | -0.06014 | 65535 | 0.050004 | -0.00115 |
| F8-T5 | 0.428202 | 0.334642 | 0.99999 | -0.01823 | 65535 | 0.066747 | 0.078179 |
| F8-T6 | -4.45634 | 0.99999 | 0.99999 | -0.14332 | 65535 | 0.999492 | -0.81361 |
| F8-Fz | -0.94937 | 0.827814 | 0.99999 | -0.0269 | 65535 | 0.216667 | -0.17333 |
| F8-Cz | 0.192438 | 0.423865 | 0.99999 | -0.03297 | 65535 | 0.054183 | 0.035134 |
| F8-Pz | -0.72302 | 0.76445 | 0.99999 | -0.01668 | 65535 | 0.156071 | -0.132 |
| F8-F9 | -0.46884 | 0.679976 | 0.99999 | -0.06869 | 65535 | 0.073815 | -0.0856 |
| F8-F10 | 1.600393 | 0.056093 | 0.624107 | -0.00182 | 65535 | 0.280548 | 0.29219 |
| T3-T4 | 1.895888 | 0.03021 | 0.556582 | 0.00502 | 65535 | 0.387431 | 0.34614 |
| T3-T5 | -1.16082 | 0.87597 | 0.99999 | -0.083 | 65535 | 0.247392 | -0.21194 |
| T3-T6 | 0.395233 | 0.346692 | 0.99999 | -0.02381 | 65535 | 0.069011 | 0.072159 |
| T3-Fz | -0.51886 | 0.697586 | 0.99999 | -0.01889 | 65535 | 0.07797 | -0.09473 |
| T3-Cz | -0.60769 | 0.72772 | 0.99999 | -0.05823 | 65535 | 0.12544 | -0.11095 |
| T3-Pz | -2.93959 | 0.998022 | 0.99999 | -0.03627 | 65535 | 0.999974 | -0.53669 |
| T3-F9 | 1.797038 | 0.037444 | 0.593725 | 0.004454 | 65535 | 0.400245 | 0.328093 |
| T3-F10 | 2.79797 | 0.003004 | 0.556582 | 0.041247 | 65535 | 0.747203 | 0.510837 |
| T4-T5 | -1.44075 | 0.923849 | 0.99999 | -0.0426 | 65535 | 0.527503 | -0.26304 |
| T4-T6 | -2.0192 | 0.977134 | 0.99999 | -0.09003 | 65535 | 0.603194 | -0.36865 |
| T4-Fz | 0.423836 | 0.336229 | 0.99999 | -0.00623 | 65535 | 0.071131 | 0.077382 |
| T4-Cz | -1.60299 | 0.944194 | 0.99999 | -0.01404 | 65535 | 0.462739 | -0.29266 |
| T4-Pz | -1.8409 | 0.965926 | 0.99999 | -0.01768 | 65535 | 0.725104 | -0.3361 |
| T4-F9 | -0.17981 | 0.571196 | 0.99999 | -0.06274 | 65535 | 0.053948 | -0.03283 |
| T4-F10 | 0.024735 | 0.490154 | 0.99999 | -0.04351 | 65535 | 0.050065 | 0.004516 |
| T5-T6 | -0.89691 | 0.814205 | 0.99999 | -0.02033 | 65535 | 0.134402 | -0.16375 |
| T5-Fz | 0.209014 | 0.417399 | 0.99999 | -0.0066 | 65535 | 0.055422 | 0.03816 |
| T5-Cz | -2.26849 | 0.987441 | 0.99999 | -0.04539 | 65535 | 0.996576 | -0.41417 |
| T5-Pz | -2.77161 | 0.996758 | 0.99999 | -0.03667 | 65535 | 0.998878 | -0.50602 |
| T5-F9 | -1.11962 | 0.867426 | 0.99999 | -0.07191 | 65535 | 0.2176 | -0.20441 |
| T5-F10 | -0.35722 | 0.639217 | 0.99999 | -0.04995 | 65535 | 0.062975 | -0.06522 |
| T6-Fz | -0.22674 | 0.589489 | 0.99999 | -0.01016 | 65535 | 0.05646 | -0.0414 |
| T6-Cz | -2.08239 | 0.980266 | 0.99999 | -0.05459 | 65535 | 0.95689 | -0.38019 |
| T6-Pz | -2.54474 | 0.993887 | 0.99999 | -0.03439 | 65535 | 0.962308 | -0.4646 |
| T6-F9 | -2.13058 | 0.9824 | 0.99999 | -0.0924 | 65535 | 0.729746 | -0.38899 |
| T6-F10 | 2.20126 | 0.014832 | 0.556582 | 0.011802 | 65535 | 0.468909 | 0.401893 |
| Fz-Cz | -0.025 | 0.509953 | 0.99999 | -0.02928 | 65535 | 0.050095 | -0.00456 |
| Fz-Pz | -2.13775 | 0.9827 | 0.99999 | -0.02368 | 65535 | 0.96104 | -0.3903 |
| Fz-F9 | 1.116747 | 0.133186 | 0.850337 | -0.00356 | 65535 | 0.18691 | 0.203889 |
| Fz-F10 | -0.55019 | 0.708385 | 0.99999 | -0.01464 | 65535 | 0.095039 | -0.10045 |
| Cz-Pz | 0.213811 | 0.415532 | 0.99999 | -0.02013 | 65535 | 0.054029 | 0.039037 |
| Cz-F9 | -1.08806 | 0.860607 | 0.99999 | -0.03943 | 65535 | 0.383567 | -0.19865 |
| Cz-F10 | -0.59483 | 0.723451 | 0.99999 | -0.02701 | 65535 | 0.120456 | -0.1086 |
| Pz-F9 | -0.52739 | 0.700545 | 0.99999 | -0.01076 | 65535 | 0.109928 | -0.09629 |
| Pz-F10 | -1.51751 | 0.934093 | 0.99999 | -0.01447 | 65535 | 0.815758 | -0.27706 |
| F9-F10 | 1.254124 | 0.106138 | 0.850337 | -0.0137 | 65535 | 0.193108 | 0.228971 |

Table S3j: gamma_coh_PT_HC

| channel | tvalue | pvalue | fdr_pvalue | CI_low | CI_high | statistical power | effect size |
| --- | --- | --- | --- | --- | --- | --- | --- |
| Fp1-Fp2 | 0.772029 | 0.77918 | 0.973974 | 65535 | 0.118919 | 0.112248 | 0.140952 |
| Fp1-F3 | 0.70096 | 0.757646 | 0.970157 | 65535 | 0.07493 | 0.108048 | 0.127977 |
| Fp1-F4 | 1.695166 | 0.953659 | 0.993257 | 65535 | 0.059992 | 0.298917 | 0.309494 |
| Fp1-C3 | 0.260365 | 0.602482 | 0.884764 | 65535 | 0.043359 | 0.059099 | 0.047536 |
| Fp1-C4 | 1.334411 | 0.907681 | 0.993257 | 65535 | 0.043582 | 0.221466 | 0.243629 |
| Fp1-P3 | 1.124516 | 0.868462 | 0.993257 | 65535 | 0.028388 | 0.176329 | 0.205308 |
| Fp1-P4 | -1.06757 | 0.143948 | 0.46674 | 65535 | 0.00484 | 0.234735 | -0.19491 |
| Fp1-O1 | -2.66543 | 0.004383 | 0.054145 | 65535 | -0.02217 | 0.912738 | -0.48664 |
| Fp1-O2 | -2.30449 | 0.011472 | 0.10038 | 65535 | -0.01326 | 0.890676 | -0.42074 |
| Fp1-F7 | 1.214918 | 0.886589 | 0.993257 | 65535 | 0.131718 | 0.228063 | 0.221813 |
| Fp1-F8 | 1.692854 | 0.953439 | 0.993257 | 65535 | 0.121641 | 0.319878 | 0.309071 |
| Fp1-T3 | 1.089362 | 0.860893 | 0.993257 | 65535 | 0.112423 | 0.173716 | 0.198889 |
| Fp1-T4 | -0.24142 | 0.404825 | 0.827739 | 65535 | 0.0355 | 0.055242 | -0.04408 |
| Fp1-T5 | -0.83729 | 0.202062 | 0.551077 | 65535 | 0.020268 | 0.142682 | -0.15287 |
| Fp1-T6 | -0.49503 | 0.310749 | 0.709318 | 65535 | 0.021321 | 0.081558 | -0.09038 |
| Fp1-Fz | 0.868258 | 0.806492 | 0.984671 | 65535 | 0.043924 | 0.123165 | 0.158521 |
| Fp1-Cz | 0.178105 | 0.570527 | 0.876362 | 65535 | 0.047999 | 0.054642 | 0.032517 |
| Fp1-Pz | -1.38858 | 0.083787 | 0.319915 | 65535 | 0.002772 | 0.499772 | -0.25352 |
| Fp1-F9 | 0.901467 | 0.815412 | 0.989806 | 65535 | 0.056449 | 0.125665 | 0.164584 |
| Fp1-F10 | 2.136794 | 0.98266 | 0.993257 | 65535 | 0.092016 | 0.482095 | 0.390123 |
| Fp2-F3 | 1.427052 | 0.921897 | 0.993257 | 65535 | 0.066192 | 0.261212 | 0.260543 |
| Fp2-F4 | 0.794786 | 0.785833 | 0.976479 | 65535 | 0.080551 | 0.116491 | 0.145107 |
| Fp2-C3 | -0.31609 | 0.376245 | 0.81455 | 65535 | 0.024794 | 0.064091 | -0.05771 |
| Fp2-C4 | -1.02907 | 0.152776 | 0.478851 | 65535 | 0.011527 | 0.20378 | -0.18788 |
| Fp2-P3 | 0.747723 | 0.771943 | 0.973974 | 65535 | 0.023087 | 0.104101 | 0.136515 |
| Fp2-P4 | 0.142909 | 0.556697 | 0.876362 | 65535 | 0.012294 | 0.051965 | 0.026091 |
| Fp2-O1 | -2.94387 | 0.001952 | 0.033559 | 65535 | -0.02687 | 0.989654 | -0.53747 |
| Fp2-O2 | -3.59993 | 0.000233 | 0.008166 | 65535 | -0.04889 | 0.999927 | -0.65725 |
| Fp2-F7 | 1.217628 | 0.887103 | 0.993257 | 65535 | 0.107576 | 0.192821 | 0.222307 |
| Fp2-F8 | -0.2532 | 0.400275 | 0.827739 | 65535 | 0.064194 | 0.056881 | -0.04623 |
| Fp2-T3 | 0.50817 | 0.693859 | 0.93404 | 65535 | 0.06396 | 0.083023 | 0.092779 |
| Fp2-T4 | 0.215663 | 0.585188 | 0.876362 | 65535 | 0.05765 | 0.054849 | 0.039375 |
| Fp2-T5 | 0.055191 | 0.52196 | 0.876362 | 65535 | 0.027223 | 0.050262 | 0.010076 |
| Fp2-T6 | -1.6457 | 0.051243 | 0.24457 | 65535 | 0.000281 | 0.494171 | -0.30046 |
| Fp2-Fz | 0.507822 | 0.693737 | 0.93404 | 65535 | 0.046378 | 0.07684 | 0.092715 |
| Fp2-Cz | -0.39712 | 0.345998 | 0.764837 | 65535 | 0.034943 | 0.078145 | -0.0725 |
| Fp2-Pz | -1.66438 | 0.049345 | 0.240989 | 65535 | -6.9E-05 | 0.693109 | -0.30387 |
| Fp2-F9 | 0.149134 | 0.559149 | 0.876362 | 65535 | 0.048082 | 0.052205 | 0.027228 |
| Fp2-F10 | 1.925418 | 0.971708 | 0.993257 | 65535 | 0.082394 | 0.414588 | 0.351532 |
| F3-F4 | 1.872523 | 0.968195 | 0.993257 | 65535 | 0.077808 | 0.270766 | 0.341874 |
| F3-C3 | -0.11864 | 0.452882 | 0.864593 | 65535 | 0.046295 | 0.051332 | -0.02166 |
| F3-C4 | 0.819068 | 0.792801 | 0.979342 | 65535 | 0.025196 | 0.114445 | 0.149541 |
| F3-P3 | -0.13724 | 0.445539 | 0.864593 | 65535 | 0.013759 | 0.052456 | -0.02506 |
| F3-P4 | 0.768532 | 0.778147 | 0.973974 | 65535 | 0.008037 | 0.095167 | 0.140314 |
| F3-O1 | -0.92409 | 0.178663 | 0.500256 | 65535 | 0.008441 | 0.184193 | -0.16871 |
| F3-O2 | -0.12095 | 0.451969 | 0.864593 | 65535 | 0.013818 | 0.051602 | -0.02208 |
| F3-F7 | 0.223966 | 0.588414 | 0.876362 | 65535 | 0.065237 | 0.056775 | 0.04089 |
| F3-F8 | 2.304444 | 0.988527 | 0.993257 | 65535 | 0.089415 | 0.485462 | 0.420732 |
| F3-T3 | 0.171575 | 0.567967 | 0.876362 | 65535 | 0.032354 | 0.053239 | 0.031325 |
| F3-T4 | 1.082238 | 0.859323 | 0.993257 | 65535 | 0.02826 | 0.130092 | 0.197589 |
| F3-T5 | -2.31216 | 0.011251 | 0.10038 | 65535 | -0.01011 | 0.951064 | -0.42214 |
| F3-T6 | 1.433108 | 0.922764 | 0.993257 | 65535 | 0.037664 | 0.177774 | 0.261649 |
| F3-Fz | 0.978011 | 0.834965 | 0.993257 | 65535 | 0.061294 | 0.141963 | 0.17856 |
| F3-Cz | -0.93334 | 0.176276 | 0.500256 | 65535 | 0.011814 | 0.256602 | -0.1704 |
| F3-Pz | -0.6171 | 0.269177 | 0.664868 | 65535 | 0.006387 | 0.121272 | -0.11267 |
| F3-F9 | -0.22718 | 0.410338 | 0.827739 | 65535 | 0.027266 | 0.05455 | -0.04148 |
| F3-F10 | -1.37141 | 0.086424 | 0.324091 | 65535 | 0.004271 | 0.314097 | -0.25038 |
| F4-C3 | 0.248829 | 0.598037 | 0.884421 | 65535 | 0.019801 | 0.055488 | 0.04543 |
| F4-C4 | -0.05472 | 0.478228 | 0.876226 | 65535 | 0.029961 | 0.0504 | -0.00999 |
| F4-P3 | 0.724589 | 0.764931 | 0.973548 | 65535 | 0.01398 | 0.098879 | 0.132291 |
| F4-P4 | 0.29712 | 0.616551 | 0.895943 | 65535 | 0.006997 | 0.059036 | 0.054246 |
| F4-O1 | 0.474015 | 0.681817 | 0.931006 | 65535 | 0.014417 | 0.067851 | 0.086543 |
| F4-O2 | -0.7592 | 0.224624 | 0.587491 | 65535 | 0.011526 | 0.184375 | -0.13861 |
| F4-F7 | 1.884816 | 0.969043 | 0.993257 | 65535 | 0.066581 | 0.360107 | 0.344119 |
| F4-F8 | 2.069355 | 0.979652 | 0.993257 | 65535 | 0.127243 | 0.540342 | 0.377811 |
| F4-T3 | 1.11635 | 0.86673 | 0.993257 | 65535 | 0.038313 | 0.146345 | 0.203817 |
| F4-T4 | 1.15115 | 0.874001 | 0.993257 | 65535 | 0.046069 | 0.150971 | 0.21017 |
| F4-T5 | 0.315878 | 0.623673 | 0.897065 | 65535 | 0.00731 | 0.059728 | 0.057671 |
| F4-T6 | -1.91335 | 0.029063 | 0.179505 | 65535 | -0.00301 | 0.609799 | -0.34933 |
| F4-Fz | 1.576151 | 0.941166 | 0.993257 | 65535 | 0.076452 | 0.314426 | 0.287765 |
| F4-Cz | 1.756526 | 0.959204 | 0.993257 | 65535 | 0.048117 | 0.292689 | 0.320696 |
| F4-Pz | 0.16407 | 0.565022 | 0.876362 | 65535 | 0.012491 | 0.052505 | 0.029955 |
| F4-F9 | 0.023484 | 0.509348 | 0.876226 | 65535 | 0.022765 | 0.050064 | 0.004288 |
| F4-F10 | 0.045476 | 0.518098 | 0.876362 | 65535 | 0.021407 | 0.05018 | 0.008303 |
| C3-C4 | 0.848217 | 0.800983 | 0.983663 | 65535 | 0.038223 | 0.102671 | 0.154863 |
| C3-P3 | 0.524491 | 0.699539 | 0.93569 | 65535 | 0.040914 | 0.078731 | 0.095759 |
| C3-P4 | -2.35558 | 0.010072 | 0.096139 | 65535 | -0.00308 | 0.99966 | -0.43007 |
| C3-O1 | -3.16733 | 0.000979 | 0.022852 | 65535 | -0.02003 | 0.999989 | -0.57827 |
| C3-O2 | -3.92192 | 7.4E-05 | 0.005179 | 65535 | -0.01588 | 1 | -0.71604 |
| C3-F7 | 0.014181 | 0.505645 | 0.876226 | 65535 | 0.035506 | 0.050029 | 0.002589 |
| C3-F8 | 0.026292 | 0.510466 | 0.876226 | 65535 | 0.02455 | 0.050071 | 0.0048 |
| C3-T3 | -0.81941 | 0.207103 | 0.557585 | 65535 | 0.02274 | 0.147235 | -0.1496 |
| C3-T4 | 0.302576 | 0.618627 | 0.895943 | 65535 | 0.020044 | 0.058304 | 0.055242 |
| C3-T5 | -0.94394 | 0.173566 | 0.500256 | 65535 | 0.008122 | 0.231068 | -0.17234 |
| C3-T6 | -1.41188 | 0.080309 | 0.318204 | 65535 | 0.001484 | 0.399437 | -0.25777 |
| C3-Fz | 1.987181 | 0.975391 | 0.993257 | 65535 | 0.048325 | 0.373374 | 0.362808 |
| C3-Cz | -1.82688 | 0.035123 | 0.189124 | 65535 | -0.00293 | 0.843404 | -0.33354 |
| C3-Pz | -2.67846 | 0.004226 | 0.054145 | 65535 | -0.01013 | 0.981685 | -0.48902 |
| C3-F9 | 0.637516 | 0.737488 | 0.970108 | 65535 | 0.046948 | 0.083844 | 0.116394 |
| C3-F10 | -0.92817 | 0.177606 | 0.500256 | 65535 | 0.008528 | 0.188306 | -0.16946 |
| C4-P3 | -0.05022 | 0.480014 | 0.876226 | 65535 | 0.004824 | 0.050303 | -0.00917 |
| C4-P4 | -1.12787 | 0.130832 | 0.451597 | 65535 | 0.00718 | 0.264551 | -0.20592 |
| C4-O1 | -2.48826 | 0.007116 | 0.078653 | 65535 | -0.0053 | 0.891648 | -0.45429 |
| C4-O2 | -1.61409 | 0.054589 | 0.249211 | 65535 | 0.000369 | 0.392903 | -0.29469 |
| C4-F7 | 0.686057 | 0.752988 | 0.970108 | 65535 | 0.029538 | 0.092357 | 0.125256 |
| C4-F8 | -2.00056 | 0.023868 | 0.151886 | 65535 | -0.00666 | 0.658616 | -0.36525 |
| C4-T3 | 1.077521 | 0.858277 | 0.993257 | 65535 | 0.024355 | 0.144118 | 0.196727 |
| C4-T4 | -3.81124 | 0.000111 | 0.005808 | 65535 | -0.05767 | 0.997249 | -0.69583 |
| C4-T5 | 0.033209 | 0.513218 | 0.876226 | 65535 | 0.010151 | 0.050084 | 0.006063 |
| C4-T6 | -0.71195 | 0.238951 | 0.597378 | 65535 | 0.011 | 0.100572 | -0.12998 |
| C4-Fz | 2.253465 | 0.986962 | 0.993257 | 65535 | 0.035234 | 0.429004 | 0.411425 |
| C4-Cz | 1.970887 | 0.974461 | 0.993257 | 65535 | 0.054373 | 0.40754 | 0.359833 |
| C4-Pz | -1.00862 | 0.15761 | 0.486736 | 65535 | 0.007692 | 0.187626 | -0.18415 |
| C4-F9 | -3.05865 | 0.001376 | 0.028894 | 65535 | -0.01802 | 0.958293 | -0.55843 |
| C4-F10 | 0.660611 | 0.744925 | 0.970108 | 65535 | 0.027039 | 0.088967 | 0.120611 |
| P3-P4 | -0.40299 | 0.343844 | 0.764837 | 65535 | 0.005837 | 0.075736 | -0.07357 |
| P3-O1 | -0.04369 | 0.482614 | 0.876226 | 65535 | 0.021333 | 0.050256 | -0.00798 |
| P3-O2 | 0.126745 | 0.550321 | 0.876362 | 65535 | 0.01338 | 0.05276 | 0.02314 |
| P3-F7 | 0.15461 | 0.561304 | 0.876362 | 65535 | 0.019944 | 0.05238 | 0.028228 |
| P3-F8 | 0.928513 | 0.822482 | 0.99265 | 65535 | 0.022602 | 0.1165 | 0.169522 |
| P3-T3 | -1.30764 | 0.096769 | 0.356516 | 65535 | 0.008914 | 0.43925 | -0.23874 |
| P3-T4 | 0.192143 | 0.57602 | 0.876362 | 65535 | 0.016129 | 0.05406 | 0.03508 |
| P3-T5 | -0.28904 | 0.386528 | 0.827739 | 65535 | 0.023177 | 0.071406 | -0.05277 |
| P3-T6 | 1.67732 | 0.951937 | 0.993257 | 65535 | 0.027114 | 0.27024 | 0.306235 |
| P3-Fz | 0.655069 | 0.743151 | 0.970108 | 65535 | 0.008492 | 0.088511 | 0.119599 |
| P3-Cz | -0.21809 | 0.413869 | 0.827739 | 65535 | 0.013361 | 0.061822 | -0.03982 |
| P3-Pz | 0.131851 | 0.552336 | 0.876362 | 65535 | 0.017219 | 0.052396 | 0.024072 |
| P3-F9 | 0.167345 | 0.566308 | 0.876362 | 65535 | 0.023309 | 0.053232 | 0.030553 |
| P3-F10 | 1.169722 | 0.877765 | 0.993257 | 65535 | 0.020046 | 0.162895 | 0.213561 |
| P4-O1 | 0.671793 | 0.748486 | 0.970108 | 65535 | 0.019373 | 0.087246 | 0.122652 |
| P4-O2 | -0.22608 | 0.410765 | 0.827739 | 65535 | 0.012433 | 0.061042 | -0.04128 |
| P4-F7 | 0.476606 | 0.682738 | 0.931006 | 65535 | 0.009217 | 0.068896 | 0.087016 |
| P4-F8 | -0.0016 | 0.499363 | 0.876226 | 65535 | 0.00998 | 0.05 | -0.00029 |
| P4-T3 | 0.446072 | 0.671819 | 0.92817 | 65535 | 0.017611 | 0.068087 | 0.081441 |
| P4-T4 | -1.8452 | 0.033758 | 0.189124 | 65535 | -0.00382 | 0.658513 | -0.33689 |
| P4-T5 | -0.92652 | 0.178034 | 0.500256 | 65535 | 0.004044 | 0.247341 | -0.16916 |
| P4-T6 | 0.414389 | 0.660329 | 0.925332 | 65535 | 0.031546 | 0.067181 | 0.075656 |
| P4-Fz | -1.05098 | 0.147708 | 0.469981 | 65535 | 0.003493 | 0.345865 | -0.19188 |
| P4-Cz | 0.020023 | 0.507971 | 0.876226 | 65535 | 0.0179 | 0.050041 | 0.003656 |
| P4-Pz | -1.55068 | 0.061828 | 0.264977 | 65535 | 0.000908 | 0.319274 | -0.28311 |
| P4-F9 | 0.360987 | 0.640623 | 0.915175 | 65535 | 0.010046 | 0.066861 | 0.065907 |
| P4-F10 | -1.56008 | 0.060709 | 0.264977 | 65535 | 0.001089 | 0.381271 | -0.28483 |
| O1-O2 | -1.83828 | 0.034268 | 0.189124 | 65535 | -0.00428 | 0.608073 | -0.33562 |
| O1-F7 | -4.19222 | 2.68E-05 | 0.002817 | 65535 | -0.064 | 0.999946 | -0.76539 |
| O1-F8 | -1.88821 | 0.030727 | 0.184359 | 65535 | -0.0047 | 0.775906 | -0.34474 |
| O1-T3 | -2.7855 | 0.003114 | 0.045383 | 65535 | -0.03141 | 0.998288 | -0.50856 |
| O1-T4 | -2.25847 | 0.012877 | 0.100819 | 65535 | -0.00756 | 0.796303 | -0.41234 |
| O1-T5 | -0.96071 | 0.16933 | 0.500256 | 65535 | 0.010578 | 0.286645 | -0.1754 |
| O1-T6 | -1.11608 | 0.133329 | 0.451597 | 65535 | 0.007486 | 0.368178 | -0.20377 |
| O1-Fz | 0.211671 | 0.583636 | 0.876362 | 65535 | 0.011087 | 0.055162 | 0.038646 |
| O1-Cz | -2.92313 | 0.002077 | 0.033559 | 65535 | -0.02944 | 0.999998 | -0.53369 |
| O1-Pz | -2.4027 | 0.008917 | 0.093625 | 65535 | -0.00554 | 0.740252 | -0.43867 |
| O1-F9 | -2.02031 | 0.022808 | 0.15006 | 65535 | -0.00614 | 0.901941 | -0.36886 |
| O1-F10 | -0.14734 | 0.441559 | 0.864593 | 65535 | 0.021747 | 0.053405 | -0.0269 |
| O2-F7 | -2.37657 | 0.009542 | 0.095417 | 65535 | -0.0147 | 0.783648 | -0.4339 |
| O2-F8 | -3.6232 | 0.000215 | 0.008166 | 65535 | -0.04978 | 0.988758 | -0.6615 |
| O2-T3 | -3.1729 | 0.000962 | 0.022852 | 65535 | -0.02948 | 0.999799 | -0.57929 |
| O2-T4 | -0.7498 | 0.227434 | 0.587491 | 65535 | 0.011546 | 0.120499 | -0.13689 |
| O2-T5 | -1.72295 | 0.043758 | 0.224129 | 65535 | -0.00064 | 0.657532 | -0.31457 |
| O2-T6 | -1.38944 | 0.083657 | 0.319915 | 65535 | 0.004566 | 0.401804 | -0.25368 |
| O2-Fz | -1.68565 | 0.047253 | 0.236264 | 65535 | -0.00016 | 0.810673 | -0.30776 |
| O2-Cz | -3.19785 | 0.000889 | 0.022852 | 65535 | -0.03224 | 1 | -0.58384 |
| O2-Pz | -1.06526 | 0.144467 | 0.46674 | 65535 | 0.006146 | 0.171852 | -0.19449 |
| O2-F9 | -1.61608 | 0.054374 | 0.249211 | 65535 | 0.000608 | 0.451231 | -0.29505 |
| O2-F10 | -0.7449 | 0.228905 | 0.587491 | 65535 | 0.013407 | 0.129209 | -0.136 |
| F7-F8 | 1.570925 | 0.940561 | 0.993257 | 65535 | 0.159872 | 0.302395 | 0.28681 |
| F7-T3 | -0.52799 | 0.29925 | 0.697878 | 65535 | 0.061035 | 0.092231 | -0.0964 |
| F7-T4 | -0.07293 | 0.470991 | 0.876226 | 65535 | 0.040395 | 0.050643 | -0.01332 |
| F7-T5 | -1.81211 | 0.036256 | 0.190342 | 65535 | -0.00359 | 0.47505 | -0.33085 |
| F7-T6 | -2.25581 | 0.012962 | 0.100819 | 65535 | -0.01357 | 0.867398 | -0.41185 |
| F7-Fz | -1.12891 | 0.130613 | 0.451597 | 65535 | 0.007438 | 0.206034 | -0.20611 |
| F7-Cz | -0.74326 | 0.229401 | 0.587491 | 65535 | 0.022608 | 0.129843 | -0.1357 |
| F7-Pz | -1.50045 | 0.068084 | 0.280348 | 65535 | 0.001213 | 0.445597 | -0.27394 |
| F7-F9 | -0.23275 | 0.408179 | 0.827739 | 65535 | 0.049747 | 0.056967 | -0.04249 |
| F7-F10 | 1.29434 | 0.900962 | 0.993257 | 65535 | 0.091476 | 0.271982 | 0.236313 |
| F8-T3 | 0.593164 | 0.722897 | 0.960812 | 65535 | 0.068371 | 0.088084 | 0.108297 |
| F8-T4 | -0.00629 | 0.497495 | 0.876226 | 65535 | 0.059682 | 0.050004 | -0.00115 |
| F8-T5 | 0.428202 | 0.665358 | 0.925332 | 65535 | 0.030933 | 0.066747 | 0.078179 |
| F8-T6 | -4.45634 | 9.55E-06 | 0.002005 | 65535 | -0.0656 | 0.999492 | -0.81361 |
| F8-Fz | -0.94937 | 0.172186 | 0.500256 | 65535 | 0.007311 | 0.216667 | -0.17333 |
| F8-Cz | 0.192438 | 0.576135 | 0.876362 | 65535 | 0.041628 | 0.054183 | 0.035134 |
| F8-Pz | -0.72302 | 0.23555 | 0.59597 | 65535 | 0.00655 | 0.156071 | -0.132 |
| F8-F9 | -0.46884 | 0.320024 | 0.722636 | 65535 | 0.038404 | 0.073815 | -0.0856 |
| F8-F10 | 1.600393 | 0.943907 | 0.993257 | 65535 | 0.103144 | 0.280548 | 0.29219 |
| T3-T4 | 1.895888 | 0.96979 | 0.993257 | 65535 | 0.074947 | 0.387431 | 0.34614 |
| T3-T5 | -1.16082 | 0.12403 | 0.449073 | 65535 | 0.014637 | 0.247392 | -0.21194 |
| T3-T6 | 0.395233 | 0.653308 | 0.925332 | 65535 | 0.03872 | 0.069011 | 0.072159 |
| T3-Fz | -0.51886 | 0.302414 | 0.697878 | 65535 | 0.009883 | 0.07797 | -0.09473 |
| T3-Cz | -0.60769 | 0.272279 | 0.664868 | 65535 | 0.026992 | 0.12544 | -0.11095 |
| T3-Pz | -2.93959 | 0.001977 | 0.033559 | 65535 | -0.01011 | 0.999974 | -0.53669 |
| T3-F9 | 1.797038 | 0.962556 | 0.993257 | 65535 | 0.110578 | 0.400245 | 0.328093 |
| T3-F10 | 2.79797 | 0.996996 | 0.996996 | 65535 | 0.161206 | 0.747203 | 0.510837 |
| T4-T5 | -1.44075 | 0.076151 | 0.307534 | 65535 | 0.002985 | 0.527503 | -0.26304 |
| T4-T6 | -2.0192 | 0.022866 | 0.15006 | 65535 | -0.00885 | 0.603194 | -0.36865 |
| T4-Fz | 0.423836 | 0.663771 | 0.925332 | 65535 | 0.010503 | 0.071131 | 0.077382 |
| T4-Cz | -1.60299 | 0.055806 | 0.249344 | 65535 | 0.000236 | 0.462739 | -0.29266 |
| T4-Pz | -1.8409 | 0.034074 | 0.189124 | 65535 | -0.00093 | 0.725104 | -0.3361 |
| T4-F9 | -0.17981 | 0.428804 | 0.849518 | 65535 | 0.050465 | 0.053948 | -0.03283 |
| T4-F10 | 0.024735 | 0.509846 | 0.876226 | 65535 | 0.04483 | 0.050065 | 0.004516 |
| T5-T6 | -0.89691 | 0.185795 | 0.513382 | 65535 | 0.006054 | 0.134402 | -0.16375 |
| T5-Fz | 0.209014 | 0.582601 | 0.876362 | 65535 | 0.008507 | 0.055422 | 0.03816 |
| T5-Cz | -2.26849 | 0.012559 | 0.100819 | 65535 | -0.00706 | 0.996576 | -0.41417 |
| T5-Pz | -2.77161 | 0.003242 | 0.045383 | 65535 | -0.00922 | 0.998878 | -0.50602 |
| T5-F9 | -1.11962 | 0.132574 | 0.451597 | 65535 | 0.013936 | 0.2176 | -0.20441 |
| T5-F10 | -0.35722 | 0.360783 | 0.789212 | 65535 | 0.032239 | 0.062975 | -0.06522 |
| T6-Fz | -0.22674 | 0.410511 | 0.827739 | 65535 | 0.007714 | 0.05646 | -0.0414 |
| T6-Cz | -2.08239 | 0.019734 | 0.138136 | 65535 | -0.0062 | 0.95689 | -0.38019 |
| T6-Pz | -2.54474 | 0.006113 | 0.071321 | 65535 | -0.00726 | 0.962308 | -0.4646 |
| T6-F9 | -2.13058 | 0.0176 | 0.127447 | 65535 | -0.01153 | 0.729746 | -0.38899 |
| T6-F10 | 2.20126 | 0.985168 | 0.993257 | 65535 | 0.083817 | 0.468909 | 0.401893 |
| Fz-Cz | -0.025 | 0.490047 | 0.876226 | 65535 | 0.028409 | 0.050095 | -0.00456 |
| Fz-Pz | -2.13775 | 0.0173 | 0.127447 | 65535 | -0.00299 | 0.96104 | -0.3903 |
| Fz-F9 | 1.116747 | 0.866814 | 0.993257 | 65535 | 0.018278 | 0.18691 | 0.203889 |
| Fz-F10 | -0.55019 | 0.291615 | 0.695899 | 65535 | 0.007346 | 0.095039 | -0.10045 |
| Cz-Pz | 0.213811 | 0.584468 | 0.876362 | 65535 | 0.026096 | 0.054029 | 0.039037 |
| Cz-F9 | -1.08806 | 0.139393 | 0.464645 | 65535 | 0.008182 | 0.383567 | -0.19865 |
| Cz-F10 | -0.59483 | 0.276549 | 0.667532 | 65535 | 0.012746 | 0.120456 | -0.1086 |
| Pz-F9 | -0.52739 | 0.299455 | 0.697878 | 65535 | 0.005564 | 0.109928 | -0.09629 |
| Pz-F10 | -1.51751 | 0.065907 | 0.276809 | 65535 | 0.000639 | 0.815758 | -0.27706 |
| F9-F10 | 1.254124 | 0.893862 | 0.993257 | 65535 | 0.098797 | 0.193108 | 0.228971 |

Table S4. Functional connectivity (wPLI)

Table S4a: alpha_wpli_HC_PT

| channel | tvalue | pvalue | fdr_pvalue | CI_low | CI_high | statistical power | effect size |
| --- | --- | --- | --- | --- | --- | --- | --- |
| Fp1-Fp2 | 3.137238 | 0.001077 | 0.001488 | 0.018621 | 65535 | 0.79851 | 0.572779 |
| Fp1-F3 | 4.124791 | 3.47E-05 | 0.000118 | 0.031499 | 65535 | 0.906256 | 0.75308 |
| Fp1-F4 | 4.834106 | 2.03E-06 | 3.29E-05 | 0.032164 | 65535 | 0.987931 | 0.882583 |
| Fp1-C3 | 3.267432 | 0.000711 | 0.001066 | 0.020045 | 65535 | 0.798188 | 0.596549 |
| Fp1-C4 | 3.522854 | 0.000304 | 0.000537 | 0.020291 | 65535 | 0.886318 | 0.643182 |
| Fp1-P3 | 3.509073 | 0.000319 | 0.000558 | 0.0312 | 65535 | 0.878434 | 0.640666 |
| Fp1-P4 | 3.192074 | 0.000905 | 0.00132 | 0.025984 | 65535 | 0.761721 | 0.58279 |
| Fp1-O1 | 3.013683 | 0.00158 | 0.002061 | 0.019762 | 65535 | 0.850784 | 0.550221 |
| Fp1-O2 | 3.30145 | 0.000636 | 0.000962 | 0.026469 | 65535 | 0.823131 | 0.60276 |
| Fp1-F7 | 4.682686 | 3.82E-06 | 4.22E-05 | 0.038063 | 65535 | 0.980278 | 0.854938 |
| Fp1-F8 | 3.566375 | 0.000262 | 0.000487 | 0.02389 | 65535 | 0.88864 | 0.651128 |
| Fp1-T3 | 2.94349 | 0.001954 | 0.0024 | 0.018083 | 65535 | 0.781728 | 0.537405 |
| Fp1-T4 | 3.842568 | 9.88E-05 | 0.000228 | 0.026504 | 65535 | 0.918231 | 0.701554 |
| Fp1-T5 | 4.683044 | 3.81E-06 | 4.22E-05 | 0.041852 | 65535 | 0.983789 | 0.855003 |
| Fp1-T6 | 4.33341 | 1.55E-05 | 7.41E-05 | 0.038071 | 65535 | 0.968029 | 0.791169 |
| Fp1-Fz | 3.989842 | 5.76E-05 | 0.000149 | 0.028682 | 65535 | 0.923738 | 0.728442 |
| Fp1-Cz | 3.333839 | 0.000572 | 0.000889 | 0.025817 | 65535 | 0.780708 | 0.608673 |
| Fp1-Pz | 2.969103 | 0.001809 | 0.002248 | 0.020602 | 65535 | 0.759011 | 0.542082 |
| Fp1-F9 | 3.865417 | 9.09E-05 | 0.000217 | 0.024554 | 65535 | 0.905194 | 0.705725 |
| Fp1-F10 | 3.820036 | 0.000107 | 0.000237 | 0.02746 | 65535 | 0.914925 | 0.69744 |
| Fp2-F3 | 3.891249 | 8.28E-05 | 0.000201 | 0.026119 | 65535 | 0.898361 | 0.710442 |
| Fp2-F4 | 4.664113 | 4.12E-06 | 4.32E-05 | 0.028472 | 65535 | 0.99108 | 0.851547 |
| Fp2-C3 | 3.499432 | 0.000329 | 0.000572 | 0.021485 | 65535 | 0.820753 | 0.638906 |
| Fp2-C4 | 3.831779 | 0.000103 | 0.000234 | 0.026406 | 65535 | 0.918202 | 0.699584 |
| Fp2-P3 | 2.747017 | 0.003479 | 0.004008 | 0.019452 | 65535 | 0.728935 | 0.501534 |
| Fp2-P4 | 3.319761 | 0.000599 | 0.000919 | 0.0276 | 65535 | 0.811069 | 0.606103 |
| Fp2-O1 | 3.527529 | 0.000299 | 0.000534 | 0.026031 | 65535 | 0.933875 | 0.644036 |
| Fp2-O2 | 3.429141 | 0.000417 | 0.00069 | 0.029523 | 65535 | 0.86243 | 0.626073 |
| Fp2-F7 | 4.307705 | 1.72E-05 | 7.69E-05 | 0.029048 | 65535 | 0.929072 | 0.786476 |
| Fp2-F8 | 4.192794 | 2.68E-05 | 9.88E-05 | 0.033818 | 65535 | 0.988586 | 0.765496 |
| Fp2-T3 | 3.665621 | 0.000186 | 0.000371 | 0.02453 | 65535 | 0.908489 | 0.669248 |
| Fp2-T4 | 3.481602 | 0.00035 | 0.000602 | 0.024575 | 65535 | 0.874291 | 0.635651 |
| Fp2-T5 | 4.170022 | 2.92E-05 | 0.000102 | 0.034949 | 65535 | 0.952321 | 0.761338 |
| Fp2-T6 | 3.86004 | 9.27E-05 | 0.000219 | 0.034648 | 65535 | 0.929645 | 0.704744 |
| Fp2-Fz | 3.195597 | 0.000895 | 0.001315 | 0.018245 | 65535 | 0.839796 | 0.583434 |
| Fp2-Cz | 2.426548 | 0.008378 | 0.008976 | 0.012908 | 65535 | 0.652099 | 0.443025 |
| Fp2-Pz | 3.038321 | 0.001465 | 0.001935 | 0.021281 | 65535 | 0.807551 | 0.554719 |
| Fp2-F9 | 4.244597 | 2.19E-05 | 8.87E-05 | 0.029131 | 65535 | 0.948918 | 0.774954 |
| Fp2-F10 | 3.741304 | 0.000142 | 0.000301 | 0.025965 | 65535 | 0.898674 | 0.683066 |
| F3-F4 | 4.280659 | 1.91E-05 | 8.01E-05 | 0.020109 | 65535 | 0.894432 | 0.781538 |
| F3-C3 | 3.332033 | 0.000576 | 0.000889 | 0.018358 | 65535 | 0.739776 | 0.608343 |
| F3-C4 | 4.105989 | 3.73E-05 | 0.000122 | 0.028918 | 65535 | 0.85457 | 0.749648 |
| F3-P3 | 4.016999 | 5.2E-05 | 0.00014 | 0.032927 | 65535 | 0.891211 | 0.7334 |
| F3-P4 | 4.078237 | 4.14E-05 | 0.000124 | 0.025271 | 65535 | 0.933475 | 0.744581 |
| F3-O1 | 3.632293 | 0.000209 | 0.000402 | 0.027194 | 65535 | 0.89981 | 0.663163 |
| F3-O2 | 3.260645 | 0.000727 | 0.001077 | 0.021811 | 65535 | 0.850699 | 0.59531 |
| F3-F7 | 2.752669 | 0.003423 | 0.003971 | 0.011737 | 65535 | 0.635018 | 0.502566 |
| F3-F8 | 4.090451 | 3.95E-05 | 0.000122 | 0.02762 | 65535 | 0.924612 | 0.746811 |
| F3-T3 | 2.009384 | 0.023389 | 0.024077 | 0.004833 | 65535 | 0.401254 | 0.366862 |
| F3-T4 | 4.426352 | 1.08E-05 | 6.64E-05 | 0.031032 | 65535 | 0.951787 | 0.808138 |
| F3-T5 | 3.753782 | 0.000136 | 0.000291 | 0.025067 | 65535 | 0.882186 | 0.685344 |
| F3-T6 | 3.769449 | 0.000129 | 0.000278 | 0.023815 | 65535 | 0.876128 | 0.688204 |
| F3-Fz | 3.017481 | 0.001562 | 0.00205 | 0.011735 | 65535 | 0.773715 | 0.550914 |
| F3-Cz | 2.920699 | 0.002093 | 0.002547 | 0.019211 | 65535 | 0.686622 | 0.533244 |
| F3-Pz | 4.09454 | 3.89E-05 | 0.000122 | 0.026852 | 65535 | 0.932214 | 0.747557 |
| F3-F9 | 4.108706 | 3.69E-05 | 0.000122 | 0.022621 | 65535 | 0.917917 | 0.750144 |
| F3-F10 | 2.979961 | 0.001751 | 0.002215 | 0.013804 | 65535 | 0.704348 | 0.544064 |
| F4-C3 | 3.35375 | 0.000536 | 0.00084 | 0.016608 | 65535 | 0.786193 | 0.612308 |
| F4-C4 | 4.316554 | 1.66E-05 | 7.69E-05 | 0.030851 | 65535 | 0.919688 | 0.788091 |
| F4-P3 | 4.301537 | 1.76E-05 | 7.69E-05 | 0.035954 | 65535 | 0.945805 | 0.78535 |
| F4-P4 | 3.820036 | 0.000107 | 0.000237 | 0.030537 | 65535 | 0.902076 | 0.69744 |
| F4-O1 | 3.962673 | 6.37E-05 | 0.000163 | 0.025643 | 65535 | 0.947102 | 0.723482 |
| F4-O2 | 4.202613 | 2.58E-05 | 9.88E-05 | 0.032287 | 65535 | 0.9626 | 0.767289 |
| F4-F7 | 4.038454 | 4.8E-05 | 0.000135 | 0.019495 | 65535 | 0.955797 | 0.737318 |
| F4-F8 | 4.714135 | 3.35E-06 | 4.2E-05 | 0.034052 | 65535 | 0.972358 | 0.860679 |
| F4-T3 | 2.988529 | 0.001706 | 0.002171 | 0.014411 | 65535 | 0.746027 | 0.545628 |
| F4-T4 | 3.672922 | 0.000181 | 0.000365 | 0.021966 | 65535 | 0.923052 | 0.670581 |
| F4-T5 | 4.90131 | 1.53E-06 | 2.93E-05 | 0.032505 | 65535 | 0.981789 | 0.894853 |
| F4-T6 | 3.727068 | 0.000149 | 0.000308 | 0.029009 | 65535 | 0.935408 | 0.680466 |
| F4-Fz | 3.259911 | 0.000728 | 0.001077 | 0.017509 | 65535 | 0.803383 | 0.595176 |
| F4-Cz | 2.585449 | 0.005471 | 0.006047 | 0.012438 | 65535 | 0.580493 | 0.472036 |
| F4-Pz | 3.110579 | 0.001171 | 0.001597 | 0.015501 | 65535 | 0.829256 | 0.567911 |
| F4-F9 | 4.877965 | 1.69E-06 | 2.96E-05 | 0.023125 | 65535 | 0.991235 | 0.890591 |
| F4-F10 | 2.97044 | 0.001802 | 0.002248 | 0.014118 | 65535 | 0.786909 | 0.542326 |
| C3-C4 | 3.120378 | 0.001135 | 0.001558 | 0.014434 | 65535 | 0.738785 | 0.569701 |
| C3-P3 | 2.570076 | 0.005706 | 0.006274 | 0.015086 | 65535 | 0.587633 | 0.46923 |
| C3-P4 | 3.581173 | 0.000249 | 0.000474 | 0.02113 | 65535 | 0.888909 | 0.65383 |
| C3-O1 | 2.9691 | 0.001809 | 0.002248 | 0.019876 | 65535 | 0.786093 | 0.542081 |
| C3-O2 | 2.412922 | 0.008682 | 0.009208 | 0.009456 | 65535 | 0.641877 | 0.440537 |
| C3-F7 | 3.404195 | 0.000454 | 0.00074 | 0.018338 | 65535 | 0.811834 | 0.621518 |
| C3-F8 | 4.138748 | 3.29E-05 | 0.000113 | 0.026301 | 65535 | 0.903974 | 0.755628 |
| C3-T3 | 2.244041 | 0.013347 | 0.014015 | 0.008119 | 65535 | 0.539951 | 0.409704 |
| C3-T4 | 3.178819 | 0.000944 | 0.001368 | 0.018349 | 65535 | 0.697682 | 0.58037 |
| C3-T5 | 2.822935 | 0.002793 | 0.003333 | 0.013357 | 65535 | 0.756831 | 0.515395 |
| C3-T6 | 2.54366 | 0.006131 | 0.006706 | 0.009441 | 65535 | 0.605754 | 0.464407 |
| C3-Fz | 3.142586 | 0.001059 | 0.001473 | 0.01396 | 65535 | 0.732492 | 0.573755 |
| C3-Cz | 1.516409 | 0.066045 | 0.066045 | -0.00186 | 65535 | 0.338226 | 0.276857 |
| C3-Pz | 1.953882 | 0.026541 | 0.027188 | 0.003571 | 65535 | 0.472401 | 0.356728 |
| C3-F9 | 3.359687 | 0.000526 | 0.000836 | 0.019674 | 65535 | 0.779535 | 0.613392 |
| C3-F10 | 2.743759 | 0.003511 | 0.004008 | 0.011226 | 65535 | 0.731423 | 0.50094 |
| C4-P3 | 3.57608 | 0.000253 | 0.000475 | 0.028345 | 65535 | 0.827584 | 0.6529 |
| C4-P4 | 4.368743 | 1.35E-05 | 7.27E-05 | 0.043204 | 65535 | 0.929584 | 0.79762 |
| C4-O1 | 4.006813 | 5.41E-05 | 0.000142 | 0.029339 | 65535 | 0.950171 | 0.731541 |
| C4-O2 | 4.710691 | 3.4E-06 | 4.2E-05 | 0.040363 | 65535 | 0.961291 | 0.860051 |
| C4-F7 | 4.556914 | 6.37E-06 | 4.96E-05 | 0.029484 | 65535 | 0.96839 | 0.831975 |
| C4-F8 | 4.228975 | 2.33E-05 | 9.23E-05 | 0.027899 | 65535 | 0.943857 | 0.772102 |
| C4-T3 | 3.437373 | 0.000406 | 0.000677 | 0.017208 | 65535 | 0.850998 | 0.627576 |
| C4-T4 | 4.080429 | 4.1E-05 | 0.000124 | 0.026169 | 65535 | 0.94884 | 0.744981 |
| C4-T5 | 4.102443 | 3.78E-05 | 0.000122 | 0.027896 | 65535 | 0.951539 | 0.749 |
| C4-T6 | 3.579361 | 0.000251 | 0.000474 | 0.028836 | 65535 | 0.907749 | 0.653499 |
| C4-Fz | 3.924898 | 7.32E-05 | 0.000185 | 0.025001 | 65535 | 0.868178 | 0.716585 |
| C4-Cz | 3.154847 | 0.001019 | 0.001446 | 0.020002 | 65535 | 0.794777 | 0.575994 |
| C4-Pz | 4.006163 | 5.42E-05 | 0.000142 | 0.030022 | 65535 | 0.938391 | 0.731422 |
| C4-F9 | 4.729819 | 3.14E-06 | 4.2E-05 | 0.028052 | 65535 | 0.964323 | 0.863543 |
| C4-F10 | 4.023437 | 5.08E-05 | 0.000139 | 0.026639 | 65535 | 0.917647 | 0.734576 |
| P3-P4 | 4.760089 | 2.77E-06 | 4.16E-05 | 0.039231 | 65535 | 0.977865 | 0.869069 |
| P3-O1 | 2.519788 | 0.00654 | 0.007116 | 0.016456 | 65535 | 0.679683 | 0.460048 |
| P3-O2 | 4.617068 | 4.99E-06 | 4.56E-05 | 0.043927 | 65535 | 0.971533 | 0.842957 |
| P3-F7 | 3.00325 | 0.001631 | 0.002101 | 0.022483 | 65535 | 0.755149 | 0.548316 |
| P3-F8 | 4.475816 | 8.83E-06 | 6.06E-05 | 0.047546 | 65535 | 0.969751 | 0.817169 |
| P3-T3 | 2.893862 | 0.002267 | 0.00272 | 0.021065 | 65535 | 0.743263 | 0.528344 |
| P3-T4 | 3.640953 | 0.000202 | 0.000401 | 0.030999 | 65535 | 0.882232 | 0.664744 |
| P3-T5 | 4.281149 | 1.9E-05 | 8.01E-05 | 0.03803 | 65535 | 0.986745 | 0.781627 |
| P3-T6 | 5.288823 | 2.87E-07 | 1E-05 | 0.04089 | 65535 | 0.996117 | 0.965602 |
| P3-Fz | 2.789458 | 0.003079 | 0.003612 | 0.014881 | 65535 | 0.658251 | 0.509283 |
| P3-Cz | 2.62038 | 0.004969 | 0.005551 | 0.016798 | 65535 | 0.637622 | 0.478414 |
| P3-Pz | 3.73635 | 0.000145 | 0.000304 | 0.032796 | 65535 | 0.890472 | 0.682161 |
| P3-F9 | 3.895358 | 8.15E-05 | 0.000201 | 0.03676 | 65535 | 0.919483 | 0.711192 |
| P3-F10 | 5.188379 | 4.46E-07 | 1.34E-05 | 0.046794 | 65535 | 0.989314 | 0.947264 |
| P4-O1 | 4.574002 | 5.95E-06 | 4.8E-05 | 0.040122 | 65535 | 0.986996 | 0.835095 |
| P4-O2 | 5.341568 | 2.27E-07 | 9.54E-06 | 0.066167 | 65535 | 0.984314 | 0.975232 |
| P4-F7 | 3.555661 | 0.000272 | 0.000496 | 0.027783 | 65535 | 0.849366 | 0.649172 |
| P4-F8 | 4.192302 | 2.68E-05 | 9.88E-05 | 0.042765 | 65535 | 0.935404 | 0.765406 |
| P4-T3 | 2.789616 | 0.003077 | 0.003612 | 0.017942 | 65535 | 0.691004 | 0.509312 |
| P4-T4 | 2.693725 | 0.004048 | 0.00457 | 0.017241 | 65535 | 0.617393 | 0.491805 |
| P4-T5 | 4.965551 | 1.17E-06 | 2.73E-05 | 0.038338 | 65535 | 0.995041 | 0.906582 |
| P4-T6 | 3.314156 | 0.000611 | 0.000929 | 0.031156 | 65535 | 0.836262 | 0.605079 |
| P4-Fz | 2.744959 | 0.003499 | 0.004008 | 0.014154 | 65535 | 0.669909 | 0.501159 |
| P4-Cz | 3.547816 | 0.000279 | 0.000505 | 0.031221 | 65535 | 0.812639 | 0.64774 |
| P4-Pz | 4.067541 | 4.31E-05 | 0.000127 | 0.034075 | 65535 | 0.922633 | 0.742628 |
| P4-F9 | 4.381679 | 1.28E-05 | 7.09E-05 | 0.034111 | 65535 | 0.963758 | 0.799981 |
| P4-F10 | 4.645498 | 4.44E-06 | 4.44E-05 | 0.050467 | 65535 | 0.97115 | 0.848148 |
| O1-O2 | 4.396066 | 1.21E-05 | 6.88E-05 | 0.042563 | 65535 | 0.973172 | 0.802608 |
| O1-F7 | 2.643215 | 0.004664 | 0.005237 | 0.015184 | 65535 | 0.730484 | 0.482583 |
| O1-F8 | 4.940476 | 1.3E-06 | 2.73E-05 | 0.0444 | 65535 | 0.989662 | 0.902003 |
| O1-T3 | 3.560036 | 0.000268 | 0.000493 | 0.025813 | 65535 | 0.849794 | 0.649971 |
| O1-T4 | 4.398856 | 1.2E-05 | 6.88E-05 | 0.036744 | 65535 | 0.960676 | 0.803118 |
| O1-T5 | 3.453023 | 0.000385 | 0.000648 | 0.027247 | 65535 | 0.919779 | 0.630433 |
| O1-T6 | 4.596314 | 5.43E-06 | 4.74E-05 | 0.035603 | 65535 | 0.987268 | 0.839168 |
| O1-Fz | 3.094496 | 0.001231 | 0.001665 | 0.016948 | 65535 | 0.838822 | 0.564975 |
| O1-Cz | 2.167635 | 0.016097 | 0.016818 | 0.007237 | 65535 | 0.525347 | 0.395754 |
| O1-Pz | 3.728758 | 0.000149 | 0.000308 | 0.030441 | 65535 | 0.952384 | 0.680775 |
| O1-F9 | 4.030288 | 4.95E-05 | 0.000137 | 0.029394 | 65535 | 0.940347 | 0.735827 |
| O1-F10 | 4.45424 | 9.63E-06 | 6.32E-05 | 0.039047 | 65535 | 0.95982 | 0.813229 |
| O2-F7 | 3.174471 | 0.000957 | 0.001377 | 0.023134 | 65535 | 0.817085 | 0.579576 |
| O2-F8 | 4.482896 | 8.58E-06 | 6.06E-05 | 0.041586 | 65535 | 0.962556 | 0.818461 |
| O2-T3 | 3.843771 | 9.84E-05 | 0.000228 | 0.030997 | 65535 | 0.906734 | 0.701773 |
| O2-T4 | 4.050648 | 4.59E-05 | 0.000132 | 0.032255 | 65535 | 0.930463 | 0.739544 |
| O2-T5 | 5.45377 | 1.38E-07 | 9.54E-06 | 0.04559 | 65535 | 0.999148 | 0.995718 |
| O2-T6 | 3.526715 | 0.0003 | 0.000534 | 0.024085 | 65535 | 0.900978 | 0.643887 |
| O2-Fz | 2.49398 | 0.007008 | 0.007586 | 0.009892 | 65535 | 0.687444 | 0.455336 |
| O2-Cz | 2.480527 | 0.007264 | 0.007823 | 0.011999 | 65535 | 0.614454 | 0.45288 |
| O2-Pz | 4.406323 | 1.16E-05 | 6.88E-05 | 0.045467 | 65535 | 0.945585 | 0.804481 |
| O2-F9 | 4.347057 | 1.47E-05 | 7.38E-05 | 0.030691 | 65535 | 0.965912 | 0.79366 |
| O2-F10 | 4.091799 | 3.93E-05 | 0.000122 | 0.030072 | 65535 | 0.947674 | 0.747057 |
| F7-F8 | 4.169662 | 2.92E-05 | 0.000102 | 0.023707 | 65535 | 0.960462 | 0.761273 |
| F7-T3 | 2.029159 | 0.022346 | 0.023117 | 0.005383 | 65535 | 0.427179 | 0.370472 |
| F7-T4 | 4.361922 | 1.39E-05 | 7.28E-05 | 0.032775 | 65535 | 0.960215 | 0.796374 |
| F7-T5 | 3.63466 | 0.000207 | 0.000402 | 0.029338 | 65535 | 0.920227 | 0.663595 |
| F7-T6 | 3.824289 | 0.000106 | 0.000237 | 0.030384 | 65535 | 0.924425 | 0.698216 |
| F7-Fz | 4.444241 | 1E-05 | 6.37E-05 | 0.029179 | 65535 | 0.973898 | 0.811404 |
| F7-Cz | 1.920215 | 0.028622 | 0.029037 | 0.003711 | 65535 | 0.426105 | 0.350582 |
| F7-Pz | 2.762762 | 0.003325 | 0.003879 | 0.015951 | 65535 | 0.703053 | 0.504409 |
| F7-F9 | 4.053336 | 4.54E-05 | 0.000132 | 0.022228 | 65535 | 0.928667 | 0.740034 |
| F7-F10 | 3.912329 | 7.66E-05 | 0.000192 | 0.026845 | 65535 | 0.935811 | 0.71429 |
| F8-T3 | 2.818206 | 0.002832 | 0.00336 | 0.013933 | 65535 | 0.823177 | 0.514532 |
| F8-T4 | 4.472388 | 8.95E-06 | 6.06E-05 | 0.037178 | 65535 | 0.954725 | 0.816543 |
| F8-T5 | 5.675343 | 5.03E-08 | 5.29E-06 | 0.047828 | 65535 | 0.996796 | 1.036171 |
| F8-T6 | 4.340099 | 1.51E-05 | 7.38E-05 | 0.041547 | 65535 | 0.972575 | 0.79239 |
| F8-Fz | 4.941512 | 1.29E-06 | 2.73E-05 | 0.03885 | 65535 | 0.983603 | 0.902192 |
| F8-Cz | 2.990535 | 0.001695 | 0.002171 | 0.020543 | 65535 | 0.783701 | 0.545994 |
| F8-Pz | 3.889976 | 8.32E-05 | 0.000201 | 0.032658 | 65535 | 0.927432 | 0.710209 |
| F8-F9 | 5.765308 | 3.33E-08 | 5.29E-06 | 0.035756 | 65535 | 0.997514 | 1.052596 |
| F8-F10 | 4.244285 | 2.2E-05 | 8.87E-05 | 0.028907 | 65535 | 0.966158 | 0.774897 |
| T3-T4 | 2.156015 | 0.016556 | 0.017212 | 0.00451 | 65535 | 0.530481 | 0.393633 |
| T3-T5 | 2.272978 | 0.012418 | 0.013105 | 0.007512 | 65535 | 0.559522 | 0.414987 |
| T3-T6 | 3.087765 | 0.001257 | 0.001682 | 0.017679 | 65535 | 0.78072 | 0.563746 |
| T3-Fz | 2.614732 | 0.005047 | 0.005608 | 0.012055 | 65535 | 0.633163 | 0.477382 |
| T3-Cz | 1.896643 | 0.03016 | 0.03045 | 0.003079 | 65535 | 0.366418 | 0.346278 |
| T3-Pz | 2.963492 | 0.00184 | 0.002273 | 0.021571 | 65535 | 0.747017 | 0.541057 |
| T3-F9 | 1.944583 | 0.027102 | 0.027629 | 0.002555 | 65535 | 0.377412 | 0.355031 |
| T3-F10 | 2.69919 | 0.003986 | 0.004524 | 0.012622 | 65535 | 0.679805 | 0.492802 |
| T4-T5 | 3.157399 | 0.001011 | 0.001444 | 0.01745 | 65535 | 0.885465 | 0.57646 |
| T4-T6 | 3.003482 | 0.00163 | 0.002101 | 0.017829 | 65535 | 0.785132 | 0.548358 |
| T4-Fz | 3.081092 | 0.001283 | 0.001706 | 0.014427 | 65535 | 0.770519 | 0.562528 |
| T4-Cz | 3.092916 | 0.001237 | 0.001665 | 0.021406 | 65535 | 0.741762 | 0.564687 |
| T4-Pz | 4.200967 | 2.59E-05 | 9.88E-05 | 0.03821 | 65535 | 0.941698 | 0.766988 |
| T4-F9 | 3.635542 | 0.000206 | 0.000402 | 0.018999 | 65535 | 0.896736 | 0.663756 |
| T4-F10 | 4.533466 | 7.01E-06 | 5.25E-05 | 0.030859 | 65535 | 0.966263 | 0.827694 |
| T5-T6 | 3.144466 | 0.001053 | 0.001473 | 0.016171 | 65535 | 0.9064 | 0.574098 |
| T5-Fz | 3.370913 | 0.000507 | 0.000812 | 0.018378 | 65535 | 0.837498 | 0.615442 |
| T5-Cz | 3.379774 | 0.000492 | 0.000795 | 0.021579 | 65535 | 0.875171 | 0.617059 |
| T5-Pz | 4.341335 | 1.5E-05 | 7.38E-05 | 0.032519 | 65535 | 0.972974 | 0.792616 |
| T5-F9 | 3.474013 | 0.000359 | 0.000613 | 0.018918 | 65535 | 0.874373 | 0.634265 |
| T5-F10 | 4.306508 | 1.72E-05 | 7.69E-05 | 0.030532 | 65535 | 0.973803 | 0.786257 |
| T6-Fz | 2.421971 | 0.008479 | 0.009039 | 0.008908 | 65535 | 0.661399 | 0.442189 |
| T6-Cz | 2.919798 | 0.002098 | 0.002547 | 0.018844 | 65535 | 0.733998 | 0.53308 |
| T6-Pz | 4.5867 | 5.65E-06 | 4.74E-05 | 0.043104 | 65535 | 0.959872 | 0.837413 |
| T6-F9 | 3.144991 | 0.001051 | 0.001473 | 0.016751 | 65535 | 0.842255 | 0.574194 |
| T6-F10 | 5.361455 | 2.08E-07 | 9.54E-06 | 0.044636 | 65535 | 0.986463 | 0.978863 |
| Fz-Cz | 3.403608 | 0.000455 | 0.00074 | 0.023655 | 65535 | 0.774053 | 0.621411 |
| Fz-Pz | 4.174593 | 2.87E-05 | 0.000102 | 0.028171 | 65535 | 0.942842 | 0.762173 |
| Fz-F9 | 3.774329 | 0.000126 | 0.000276 | 0.019326 | 65535 | 0.903648 | 0.689095 |
| Fz-F10 | 3.356992 | 0.00053 | 0.000838 | 0.019111 | 65535 | 0.81575 | 0.6129 |
| Cz-Pz | 1.758196 | 0.040654 | 0.040848 | 0.001363 | 65535 | 0.468669 | 0.321001 |
| Cz-F9 | 3.452639 | 0.000386 | 0.000648 | 0.019416 | 65535 | 0.832535 | 0.630363 |
| Cz-F10 | 2.899318 | 0.00223 | 0.002692 | 0.015692 | 65535 | 0.675622 | 0.529341 |
| Pz-F9 | 3.678437 | 0.000177 | 0.000362 | 0.025365 | 65535 | 0.875563 | 0.671588 |
| Pz-F10 | 4.621333 | 4.91E-06 | 4.56E-05 | 0.040561 | 65535 | 0.960795 | 0.843736 |
| F9-F10 | 4.039229 | 4.79E-05 | 0.000135 | 0.020469 | 65535 | 0.935849 | 0.737459 |

Table S4b: alpha_wpli_PT_HC

| channel | tvalue | pvalue | fdr_pvalue | CI_low | CI_high | statistical power | effect size |
| --- | --- | --- | --- | --- | --- | --- | --- |
| Fp1-Fp2 | 3.137238 | 0.998923 | 1 | 65535 | 0.060358 | 0.79851 | 0.572779 |
| Fp1-F3 | 4.124791 | 0.999965 | 1 | 65535 | 0.073836 | 0.906256 | 0.75308 |
| Fp1-F4 | 4.834106 | 0.999998 | 1 | 65535 | 0.065741 | 0.987931 | 0.882583 |
| Fp1-C3 | 3.267432 | 0.999289 | 1 | 65535 | 0.061339 | 0.798188 | 0.596549 |
| Fp1-C4 | 3.522854 | 0.999696 | 1 | 65535 | 0.056366 | 0.886318 | 0.643182 |
| Fp1-P3 | 3.509073 | 0.999681 | 1 | 65535 | 0.087084 | 0.878434 | 0.640666 |
| Fp1-P4 | 3.192074 | 0.999095 | 1 | 65535 | 0.08214 | 0.761721 | 0.58279 |
| Fp1-O1 | 3.013683 | 0.99842 | 1 | 65535 | 0.068091 | 0.850784 | 0.550221 |
| Fp1-O2 | 3.30145 | 0.999364 | 1 | 65535 | 0.079866 | 0.823131 | 0.60276 |
| Fp1-F7 | 4.682686 | 0.999996 | 1 | 65535 | 0.079788 | 0.980278 | 0.854938 |
| Fp1-F8 | 3.566375 | 0.999738 | 1 | 65535 | 0.065394 | 0.88864 | 0.651128 |
| Fp1-T3 | 2.94349 | 0.998046 | 1 | 65535 | 0.06472 | 0.781728 | 0.537405 |
| Fp1-T4 | 3.842568 | 0.999901 | 1 | 65535 | 0.066729 | 0.918231 | 0.701554 |
| Fp1-T5 | 4.683044 | 0.999996 | 1 | 65535 | 0.087723 | 0.983789 | 0.855003 |
| Fp1-T6 | 4.33341 | 0.999984 | 1 | 65535 | 0.085251 | 0.968029 | 0.791169 |
| Fp1-Fz | 3.989842 | 0.999942 | 1 | 65535 | 0.069465 | 0.923738 | 0.728442 |
| Fp1-Cz | 3.333839 | 0.999428 | 1 | 65535 | 0.076894 | 0.780708 | 0.608673 |
| Fp1-Pz | 2.969103 | 0.998191 | 1 | 65535 | 0.072698 | 0.759011 | 0.542082 |
| Fp1-F9 | 3.865417 | 0.999909 | 1 | 65535 | 0.061435 | 0.905194 | 0.705725 |
| Fp1-F10 | 3.820036 | 0.999893 | 1 | 65535 | 0.069571 | 0.914925 | 0.69744 |
| Fp2-F3 | 3.891249 | 0.999917 | 1 | 65535 | 0.064897 | 0.898361 | 0.710442 |
| Fp2-F4 | 4.664113 | 0.999996 | 1 | 65535 | 0.059876 | 0.99108 | 0.851547 |
| Fp2-C3 | 3.499432 | 0.999671 | 1 | 65535 | 0.060168 | 0.820753 | 0.638906 |
| Fp2-C4 | 3.831779 | 0.999897 | 1 | 65535 | 0.066681 | 0.918202 | 0.699584 |
| Fp2-P3 | 2.747017 | 0.996521 | 1 | 65535 | 0.078671 | 0.728935 | 0.501534 |
| Fp2-P4 | 3.319761 | 0.999401 | 1 | 65535 | 0.082665 | 0.811069 | 0.606103 |
| Fp2-O1 | 3.527529 | 0.999701 | 1 | 65535 | 0.072195 | 0.933875 | 0.644036 |
| Fp2-O2 | 3.429141 | 0.999583 | 1 | 65535 | 0.084789 | 0.86243 | 0.626073 |
| Fp2-F7 | 4.307705 | 0.999983 | 1 | 65535 | 0.065397 | 0.929072 | 0.786476 |
| Fp2-F8 | 4.192794 | 0.999973 | 1 | 65535 | 0.078052 | 0.988586 | 0.765496 |
| Fp2-T3 | 3.665621 | 0.999814 | 1 | 65535 | 0.06504 | 0.908489 | 0.669248 |
| Fp2-T4 | 3.481602 | 0.99965 | 1 | 65535 | 0.069255 | 0.874291 | 0.635651 |
| Fp2-T5 | 4.170022 | 0.999971 | 1 | 65535 | 0.081077 | 0.952321 | 0.761338 |
| Fp2-T6 | 3.86004 | 0.999907 | 1 | 65535 | 0.086817 | 0.929645 | 0.704744 |
| Fp2-Fz | 3.195597 | 0.999105 | 1 | 65535 | 0.057586 | 0.839796 | 0.583434 |
| Fp2-Cz | 2.426548 | 0.991622 | 1 | 65535 | 0.068588 | 0.652099 | 0.443025 |
| Fp2-Pz | 3.038321 | 0.998535 | 1 | 65535 | 0.072395 | 0.807551 | 0.554719 |
| Fp2-F9 | 4.244597 | 0.999978 | 1 | 65535 | 0.066472 | 0.948918 | 0.774954 |
| Fp2-F10 | 3.741304 | 0.999858 | 1 | 65535 | 0.067288 | 0.898674 | 0.683066 |
| F3-F4 | 4.280659 | 0.999981 | 1 | 65535 | 0.04553 | 0.894432 | 0.781538 |
| F3-C3 | 3.332033 | 0.999424 | 1 | 65535 | 0.054717 | 0.739776 | 0.608343 |
| F3-C4 | 4.105989 | 0.999963 | 1 | 65535 | 0.068084 | 0.85457 | 0.749648 |
| F3-P3 | 4.016999 | 0.999948 | 1 | 65535 | 0.079205 | 0.891211 | 0.7334 |
| F3-P4 | 4.078237 | 0.999959 | 1 | 65535 | 0.05989 | 0.933475 | 0.744581 |
| F3-O1 | 3.632293 | 0.999791 | 1 | 65535 | 0.072862 | 0.89981 | 0.663163 |
| F3-O2 | 3.260645 | 0.999273 | 1 | 65535 | 0.066933 | 0.850699 | 0.59531 |
| F3-F7 | 2.752669 | 0.996577 | 1 | 65535 | 0.047283 | 0.635018 | 0.502566 |
| F3-F8 | 4.090451 | 0.99996 | 1 | 65535 | 0.065268 | 0.924612 | 0.746811 |
| F3-T3 | 2.009384 | 0.976611 | 1 | 65535 | 0.050419 | 0.401254 | 0.366862 |
| F3-T4 | 4.426352 | 0.999989 | 1 | 65535 | 0.068198 | 0.951787 | 0.808138 |
| F3-T5 | 3.753782 | 0.999864 | 1 | 65535 | 0.064724 | 0.882186 | 0.685344 |
| F3-T6 | 3.769449 | 0.999871 | 1 | 65535 | 0.061211 | 0.876128 | 0.688204 |
| F3-Fz | 3.017481 | 0.998438 | 1 | 65535 | 0.040354 | 0.773715 | 0.550914 |
| F3-Cz | 2.920699 | 0.997907 | 1 | 65535 | 0.069653 | 0.686622 | 0.533244 |
| F3-Pz | 4.09454 | 0.999961 | 1 | 65535 | 0.063392 | 0.932214 | 0.747557 |
| F3-F9 | 4.108706 | 0.999963 | 1 | 65535 | 0.053226 | 0.917917 | 0.750144 |
| F3-F10 | 2.979961 | 0.998249 | 1 | 65535 | 0.048424 | 0.704348 | 0.544064 |
| F4-C3 | 3.35375 | 0.999464 | 1 | 65535 | 0.049081 | 0.786193 | 0.612308 |
| F4-C4 | 4.316554 | 0.999983 | 1 | 65535 | 0.069326 | 0.919688 | 0.788091 |
| F4-P3 | 4.301537 | 0.999982 | 1 | 65535 | 0.081047 | 0.945805 | 0.78535 |
| F4-P4 | 3.820036 | 0.999893 | 1 | 65535 | 0.077366 | 0.902076 | 0.69744 |
| F4-O1 | 3.962673 | 0.999936 | 1 | 65535 | 0.062534 | 0.947102 | 0.723482 |
| F4-O2 | 4.202613 | 0.999974 | 1 | 65535 | 0.074357 | 0.9626 | 0.767289 |
| F4-F7 | 4.038454 | 0.999952 | 1 | 65535 | 0.046648 | 0.955797 | 0.737318 |
| F4-F8 | 4.714135 | 0.999997 | 1 | 65535 | 0.070996 | 0.972358 | 0.860679 |
| F4-T3 | 2.988529 | 0.998294 | 1 | 65535 | 0.05032 | 0.746027 | 0.545628 |
| F4-T4 | 3.672922 | 0.999819 | 1 | 65535 | 0.058112 | 0.923052 | 0.670581 |
| F4-T5 | 4.90131 | 0.999998 | 1 | 65535 | 0.065734 | 0.981789 | 0.894853 |
| F4-T6 | 3.727068 | 0.999851 | 1 | 65535 | 0.075494 | 0.935408 | 0.680466 |
| F4-Fz | 3.259911 | 0.999272 | 1 | 65535 | 0.053747 | 0.803383 | 0.595176 |
| F4-Cz | 2.585449 | 0.994529 | 1 | 65535 | 0.056901 | 0.580493 | 0.472036 |
| F4-Pz | 3.110579 | 0.998829 | 1 | 65535 | 0.050881 | 0.829256 | 0.567911 |
| F4-F9 | 4.877965 | 0.999998 | 1 | 65535 | 0.046936 | 0.991235 | 0.890591 |
| F4-F10 | 2.97044 | 0.998198 | 1 | 65535 | 0.049781 | 0.786909 | 0.542326 |
| C3-C4 | 3.120378 | 0.998865 | 1 | 65535 | 0.047158 | 0.738785 | 0.569701 |
| C3-P3 | 2.570076 | 0.994294 | 1 | 65535 | 0.06992 | 0.587633 | 0.46923 |
| C3-P4 | 3.581173 | 0.999751 | 1 | 65535 | 0.057557 | 0.888909 | 0.65383 |
| C3-O1 | 2.9691 | 0.998191 | 1 | 65535 | 0.070136 | 0.786093 | 0.542081 |
| C3-O2 | 2.412922 | 0.991318 | 1 | 65535 | 0.050983 | 0.641877 | 0.440537 |
| C3-F7 | 3.404195 | 0.999546 | 1 | 65535 | 0.053158 | 0.811834 | 0.621518 |
| C3-F8 | 4.138748 | 0.999967 | 1 | 65535 | 0.061452 | 0.903974 | 0.755628 |
| C3-T3 | 2.244041 | 0.986653 | 1 | 65535 | 0.054048 | 0.539951 | 0.409704 |
| C3-T4 | 3.178819 | 0.999056 | 1 | 65535 | 0.058351 | 0.697682 | 0.58037 |
| C3-T5 | 2.822935 | 0.997207 | 1 | 65535 | 0.05137 | 0.756831 | 0.515395 |
| C3-T6 | 2.54366 | 0.993869 | 1 | 65535 | 0.044783 | 0.605754 | 0.464407 |
| C3-Fz | 3.142586 | 0.998941 | 1 | 65535 | 0.045136 | 0.732492 | 0.573755 |
| C3-Cz | 1.516409 | 0.933955 | 1 | 65535 | 0.041823 | 0.338226 | 0.276857 |
| C3-Pz | 1.953882 | 0.973459 | 1 | 65535 | 0.043569 | 0.472401 | 0.356728 |
| C3-F9 | 3.359687 | 0.999474 | 1 | 65535 | 0.058007 | 0.779535 | 0.613392 |
| C3-F10 | 2.743759 | 0.996489 | 1 | 65535 | 0.045505 | 0.731423 | 0.50094 |
| C4-P3 | 3.57608 | 0.999747 | 1 | 65535 | 0.07734 | 0.827584 | 0.6529 |
| C4-P4 | 4.368743 | 0.999986 | 1 | 65535 | 0.096048 | 0.929584 | 0.79762 |
| C4-O1 | 4.006813 | 0.999946 | 1 | 65535 | 0.070755 | 0.950171 | 0.731541 |
| C4-O2 | 4.710691 | 0.999997 | 1 | 65535 | 0.084202 | 0.961291 | 0.860051 |
| C4-F7 | 4.556914 | 0.999994 | 1 | 65535 | 0.063205 | 0.96839 | 0.831975 |
| C4-F8 | 4.228975 | 0.999977 | 1 | 65535 | 0.063879 | 0.943857 | 0.772102 |
| C4-T3 | 3.437373 | 0.999594 | 1 | 65535 | 0.04927 | 0.850998 | 0.627576 |
| C4-T4 | 4.080429 | 0.999959 | 1 | 65535 | 0.061987 | 0.94884 | 0.744981 |
| C4-T5 | 4.102443 | 0.999962 | 1 | 65535 | 0.065734 | 0.951539 | 0.749 |
| C4-T6 | 3.579361 | 0.999749 | 1 | 65535 | 0.078596 | 0.907749 | 0.653499 |
| C4-Fz | 3.924898 | 0.999927 | 1 | 65535 | 0.061567 | 0.868178 | 0.716585 |
| C4-Cz | 3.154847 | 0.998981 | 1 | 65535 | 0.064304 | 0.794777 | 0.575994 |
| C4-Pz | 4.006163 | 0.999946 | 1 | 65535 | 0.072412 | 0.938391 | 0.731422 |
| C4-F9 | 4.729819 | 0.999997 | 1 | 65535 | 0.05833 | 0.964323 | 0.863543 |
| C4-F10 | 4.023437 | 0.999949 | 1 | 65535 | 0.063978 | 0.917647 | 0.734576 |
| P3-P4 | 4.760089 | 0.999997 | 1 | 65535 | 0.081163 | 0.977865 | 0.869069 |
| P3-O1 | 2.519788 | 0.99346 | 1 | 65535 | 0.07976 | 0.679683 | 0.460048 |
| P3-O2 | 4.617068 | 0.999995 | 1 | 65535 | 0.093147 | 0.971533 | 0.842957 |
| P3-F7 | 3.00325 | 0.998369 | 1 | 65535 | 0.077895 | 0.755149 | 0.548316 |
| P3-F8 | 4.475816 | 0.999991 | 1 | 65535 | 0.103492 | 0.969751 | 0.817169 |
| P3-T3 | 2.893862 | 0.997733 | 1 | 65535 | 0.077574 | 0.743263 | 0.528344 |
| P3-T4 | 3.640953 | 0.999798 | 1 | 65535 | 0.082829 | 0.882232 | 0.664744 |
| P3-T5 | 4.281149 | 0.999981 | 1 | 65535 | 0.086098 | 0.986745 | 0.781627 |
| P3-T6 | 5.288823 | 1 | 1 | 65535 | 0.078229 | 0.996117 | 0.965602 |
| P3-Fz | 2.789458 | 0.996921 | 1 | 65535 | 0.058486 | 0.658251 | 0.509283 |
| P3-Cz | 2.62038 | 0.995031 | 1 | 65535 | 0.074663 | 0.637622 | 0.478414 |
| P3-Pz | 3.73635 | 0.999855 | 1 | 65535 | 0.085115 | 0.890472 | 0.682161 |
| P3-F9 | 3.895358 | 0.999918 | 1 | 65535 | 0.091235 | 0.919483 | 0.711192 |
| P3-F10 | 5.188379 | 1 | 1 | 65535 | 0.090742 | 0.989314 | 0.947264 |
| P4-O1 | 4.574002 | 0.999994 | 1 | 65535 | 0.085741 | 0.986996 | 0.835095 |
| P4-O2 | 5.341568 | 1 | 1 | 65535 | 0.125725 | 0.984314 | 0.975232 |
| P4-F7 | 3.555661 | 0.999728 | 1 | 65535 | 0.076325 | 0.849366 | 0.649172 |
| P4-F8 | 4.192302 | 0.999973 | 1 | 65535 | 0.098714 | 0.935404 | 0.765406 |
| P4-T3 | 2.789616 | 0.996923 | 1 | 65535 | 0.070508 | 0.691004 | 0.509312 |
| P4-T4 | 2.693725 | 0.995952 | 1 | 65535 | 0.072429 | 0.617393 | 0.491805 |
| P4-T5 | 4.965551 | 0.999999 | 1 | 65535 | 0.076769 | 0.995041 | 0.906582 |
| P4-T6 | 3.314156 | 0.999389 | 1 | 65535 | 0.093529 | 0.836262 | 0.605079 |
| P4-Fz | 2.744959 | 0.996501 | 1 | 65535 | 0.057324 | 0.669909 | 0.501159 |
| P4-Cz | 3.547816 | 0.999721 | 1 | 65535 | 0.085994 | 0.812639 | 0.64774 |
| P4-Pz | 4.067541 | 0.999957 | 1 | 65535 | 0.080962 | 0.922633 | 0.742628 |
| P4-F9 | 4.381679 | 0.999987 | 1 | 65535 | 0.075636 | 0.963758 | 0.799981 |
| P4-F10 | 4.645498 | 0.999996 | 1 | 65535 | 0.106476 | 0.97115 | 0.848148 |
| O1-O2 | 4.396066 | 0.999988 | 1 | 65535 | 0.094103 | 0.973172 | 0.802608 |
| O1-F7 | 2.643215 | 0.995336 | 1 | 65535 | 0.06628 | 0.730484 | 0.482583 |
| O1-F8 | 4.940476 | 0.999999 | 1 | 65535 | 0.089248 | 0.989662 | 0.902003 |
| O1-T3 | 3.560036 | 0.999732 | 1 | 65535 | 0.070808 | 0.849794 | 0.649971 |
| O1-T4 | 4.398856 | 0.999988 | 1 | 65535 | 0.081193 | 0.960676 | 0.803118 |
| O1-T5 | 3.453023 | 0.999615 | 1 | 65535 | 0.077572 | 0.919779 | 0.630433 |
| O1-T6 | 4.596314 | 0.999995 | 1 | 65535 | 0.075778 | 0.987268 | 0.839168 |
| O1-Fz | 3.094496 | 0.998769 | 1 | 65535 | 0.056063 | 0.838822 | 0.564975 |
| O1-Cz | 2.167635 | 0.983903 | 1 | 65535 | 0.054313 | 0.525347 | 0.395754 |
| O1-Pz | 3.728758 | 0.999851 | 1 | 65535 | 0.07918 | 0.952384 | 0.680775 |
| O1-F9 | 4.030288 | 0.99995 | 1 | 65535 | 0.070476 | 0.940347 | 0.735827 |
| O1-F10 | 4.45424 | 0.99999 | 1 | 65535 | 0.085345 | 0.95982 | 0.813229 |
| O2-F7 | 3.174471 | 0.999043 | 1 | 65535 | 0.073711 | 0.817085 | 0.579576 |
| O2-F8 | 4.482896 | 0.999991 | 1 | 65535 | 0.090395 | 0.962556 | 0.818461 |
| O2-T3 | 3.843771 | 0.999902 | 1 | 65535 | 0.078017 | 0.906734 | 0.701773 |
| O2-T4 | 4.050648 | 0.999954 | 1 | 65535 | 0.076952 | 0.930463 | 0.739544 |
| O2-T5 | 5.45377 | 1 | 1 | 65535 | 0.085413 | 0.999148 | 0.995718 |
| O2-T6 | 3.526715 | 0.9997 | 1 | 65535 | 0.066816 | 0.900978 | 0.643887 |
| O2-Fz | 2.49398 | 0.992992 | 1 | 65535 | 0.049122 | 0.687444 | 0.455336 |
| O2-Cz | 2.480527 | 0.992736 | 1 | 65535 | 0.060363 | 0.614454 | 0.45288 |
| O2-Pz | 4.406323 | 0.999988 | 1 | 65535 | 0.100319 | 0.945585 | 0.804481 |
| O2-F9 | 4.347057 | 0.999985 | 1 | 65535 | 0.068532 | 0.965912 | 0.79366 |
| O2-F10 | 4.091799 | 0.999961 | 1 | 65535 | 0.07104 | 0.947674 | 0.747057 |
| F7-F8 | 4.169662 | 0.999971 | 1 | 65535 | 0.055002 | 0.960462 | 0.761273 |
| F7-T3 | 2.029159 | 0.977654 | 1 | 65535 | 0.053456 | 0.427179 | 0.370472 |
| F7-T4 | 4.361922 | 0.999986 | 1 | 65535 | 0.072964 | 0.960215 | 0.796374 |
| F7-T5 | 3.63466 | 0.999793 | 1 | 65535 | 0.078547 | 0.920227 | 0.663595 |
| F7-T6 | 3.824289 | 0.999894 | 1 | 65535 | 0.076887 | 0.924425 | 0.698216 |
| F7-Fz | 4.444241 | 0.99999 | 1 | 65535 | 0.0639 | 0.973898 | 0.811404 |
| F7-Cz | 1.920215 | 0.971378 | 1 | 65535 | 0.050609 | 0.426105 | 0.350582 |
| F7-Pz | 2.762762 | 0.996675 | 1 | 65535 | 0.063819 | 0.703053 | 0.504409 |
| F7-F9 | 4.053336 | 0.999955 | 1 | 65535 | 0.052996 | 0.928667 | 0.740034 |
| F7-F10 | 3.912329 | 0.999923 | 1 | 65535 | 0.066327 | 0.935811 | 0.71429 |
| F8-T3 | 2.818206 | 0.997168 | 1 | 65535 | 0.053747 | 0.823177 | 0.514532 |
| F8-T4 | 4.472388 | 0.999991 | 1 | 65535 | 0.080976 | 0.954725 | 0.816543 |
| F8-T5 | 5.675343 | 1 | 1 | 65535 | 0.087301 | 0.996796 | 1.036171 |
| F8-T6 | 4.340099 | 0.999985 | 1 | 65535 | 0.092906 | 0.972575 | 0.79239 |
| F8-Fz | 4.941512 | 0.999999 | 1 | 65535 | 0.078079 | 0.983603 | 0.902192 |
| F8-Cz | 2.990535 | 0.998305 | 1 | 65535 | 0.071656 | 0.783701 | 0.545994 |
| F8-Pz | 3.889976 | 0.999917 | 1 | 65535 | 0.08117 | 0.927432 | 0.710209 |
| F8-F9 | 5.765308 | 1 | 1 | 65535 | 0.06462 | 0.997514 | 1.052596 |
| F8-F10 | 4.244285 | 0.999978 | 1 | 65535 | 0.065966 | 0.966158 | 0.774897 |
| T3-T4 | 2.156015 | 0.983444 | 1 | 65535 | 0.034531 | 0.530481 | 0.393633 |
| T3-T5 | 2.272978 | 0.987582 | 1 | 65535 | 0.048006 | 0.559522 | 0.414987 |
| T3-T6 | 3.087765 | 0.998743 | 1 | 65535 | 0.058673 | 0.78072 | 0.563746 |
| T3-Fz | 2.614732 | 0.994953 | 1 | 65535 | 0.053829 | 0.633163 | 0.477382 |
| T3-Cz | 1.896643 | 0.96984 | 1 | 65535 | 0.04583 | 0.366418 | 0.346278 |
| T3-Pz | 2.963492 | 0.99816 | 1 | 65535 | 0.076351 | 0.747017 | 0.541057 |
| T3-F9 | 1.944583 | 0.972898 | 1 | 65535 | 0.032107 | 0.377412 | 0.355031 |
| T3-F10 | 2.69919 | 0.996014 | 1 | 65535 | 0.052813 | 0.679805 | 0.492802 |
| T4-T5 | 3.157399 | 0.998989 | 1 | 65535 | 0.056035 | 0.885465 | 0.57646 |
| T4-T6 | 3.003482 | 0.99837 | 1 | 65535 | 0.06176 | 0.785132 | 0.548358 |
| T4-Fz | 3.081092 | 0.998717 | 1 | 65535 | 0.048037 | 0.770519 | 0.562528 |
| T4-Cz | 3.092916 | 0.998763 | 1 | 65535 | 0.070865 | 0.741762 | 0.564687 |
| T4-Pz | 4.200967 | 0.999974 | 1 | 65535 | 0.088028 | 0.941698 | 0.766988 |
| T4-F9 | 3.635542 | 0.999794 | 1 | 65535 | 0.050853 | 0.896736 | 0.663756 |
| T4-F10 | 4.533466 | 0.999993 | 1 | 65535 | 0.066441 | 0.966263 | 0.827694 |
| T5-T6 | 3.144466 | 0.998947 | 1 | 65535 | 0.052238 | 0.9064 | 0.574098 |
| T5-Fz | 3.370913 | 0.999493 | 1 | 65535 | 0.053949 | 0.837498 | 0.615442 |
| T5-Cz | 3.379774 | 0.999508 | 1 | 65535 | 0.063131 | 0.875171 | 0.617059 |
| T5-Pz | 4.341335 | 0.999985 | 1 | 65535 | 0.072701 | 0.972974 | 0.792616 |
| T5-F9 | 3.474013 | 0.999641 | 1 | 65535 | 0.053457 | 0.874373 | 0.634265 |
| T5-F10 | 4.306508 | 0.999983 | 1 | 65535 | 0.068755 | 0.973803 | 0.786257 |
| T6-Fz | 2.421971 | 0.991521 | 1 | 65535 | 0.047565 | 0.661399 | 0.442189 |
| T6-Cz | 2.919798 | 0.997902 | 1 | 65535 | 0.068358 | 0.733998 | 0.53308 |
| T6-Pz | 4.5867 | 0.999994 | 1 | 65535 | 0.091902 | 0.959872 | 0.837413 |
| T6-F9 | 3.144991 | 0.998949 | 1 | 65535 | 0.0541 | 0.842255 | 0.574194 |
| T6-F10 | 5.361455 | 1 | 1 | 65535 | 0.084598 | 0.986463 | 0.978863 |
| Fz-Cz | 3.403608 | 0.999545 | 1 | 65535 | 0.068585 | 0.774053 | 0.621411 |
| Fz-Pz | 4.174593 | 0.999971 | 1 | 65535 | 0.065287 | 0.942842 | 0.762173 |
| Fz-F9 | 3.774329 | 0.999874 | 1 | 65535 | 0.049603 | 0.903648 | 0.689095 |
| Fz-F10 | 3.356992 | 0.99947 | 1 | 65535 | 0.056406 | 0.81575 | 0.6129 |
| Cz-Pz | 1.758196 | 0.959346 | 1 | 65535 | 0.046423 | 0.468669 | 0.321001 |
| Cz-F9 | 3.452639 | 0.999614 | 1 | 65535 | 0.055285 | 0.832535 | 0.630363 |
| Cz-F10 | 2.899318 | 0.99777 | 1 | 65535 | 0.057604 | 0.675622 | 0.529341 |
| Pz-F9 | 3.678437 | 0.999823 | 1 | 65535 | 0.06699 | 0.875563 | 0.671588 |
| Pz-F10 | 4.621333 | 0.999995 | 1 | 65535 | 0.085943 | 0.960795 | 0.843736 |
| F9-F10 | 4.039229 | 0.999952 | 1 | 65535 | 0.048969 | 0.935849 | 0.737459 |

Table S4c: beta_wpli_HC_PT

| channel | tvalue | pvalue | fdr_pvalue | CI_low | CI_high | statistical power | effect size |
| --- | --- | --- | --- | --- | --- | --- | --- |
| Fp1-Fp2 | 2.644812 | 0.004643 | 0.009222 | 0.002939 | 65535 | 0.660736 | 0.482874 |
| Fp1-F3 | 1.300235 | 0.098027 | 0.111879 | -0.00114 | 65535 | 0.244873 | 0.237389 |
| Fp1-F4 | 1.944744 | 0.027093 | 0.037186 | 0.000901 | 65535 | 0.506132 | 0.35506 |
| Fp1-C3 | 4.009975 | 5.34E-05 | 0.000449 | 0.009712 | 65535 | 0.913025 | 0.732118 |
| Fp1-C4 | 3.269478 | 0.000706 | 0.002317 | 0.006584 | 65535 | 0.822585 | 0.596922 |
| Fp1-P3 | 2.180105 | 0.015618 | 0.023766 | 0.002205 | 65535 | 0.540896 | 0.398031 |
| Fp1-P4 | 3.513265 | 0.000314 | 0.001294 | 0.009213 | 65535 | 0.88767 | 0.641431 |
| Fp1-O1 | 2.795978 | 0.003021 | 0.006409 | 0.004533 | 65535 | 0.672841 | 0.510473 |
| Fp1-O2 | 4.023407 | 5.08E-05 | 0.000449 | 0.009134 | 65535 | 0.916839 | 0.73457 |
| Fp1-F7 | 2.540657 | 0.006181 | 0.01119 | 0.002787 | 65535 | 0.579366 | 0.463858 |
| Fp1-F8 | 1.8802 | 0.031273 | 0.041566 | 0.000843 | 65535 | 0.546916 | 0.343276 |
| Fp1-T3 | 2.362171 | 0.009903 | 0.016636 | 0.002069 | 65535 | 0.511137 | 0.431271 |
| Fp1-T4 | 3.242037 | 0.000772 | 0.002383 | 0.004333 | 65535 | 0.836942 | 0.591912 |
| Fp1-T5 | 3.031004 | 0.001498 | 0.003933 | 0.004747 | 65535 | 0.755351 | 0.553383 |
| Fp1-T6 | 3.970029 | 6.2E-05 | 0.000482 | 0.012762 | 65535 | 0.868274 | 0.724825 |
| Fp1-Fz | 2.265082 | 0.012666 | 0.020304 | 0.00203 | 65535 | 0.669867 | 0.413545 |
| Fp1-Cz | 1.632202 | 0.052651 | 0.064284 | -0.00012 | 65535 | 0.3746 | 0.297998 |
| Fp1-Pz | 3.803057 | 0.000114 | 0.000658 | 0.007361 | 65535 | 0.873294 | 0.69434 |
| Fp1-F9 | 1.536263 | 0.063575 | 0.075429 | -0.00038 | 65535 | 0.309488 | 0.280482 |
| Fp1-F10 | 2.898908 | 0.002233 | 0.005329 | 0.004489 | 65535 | 0.683383 | 0.529266 |
| Fp2-F3 | 0.304123 | 0.380785 | 0.382607 | -0.00453 | 65535 | 0.061333 | 0.055525 |
| Fp2-F4 | 1.639295 | 0.051908 | 0.063746 | -5.3E-05 | 65535 | 0.392937 | 0.299293 |
| Fp2-C3 | 3.616573 | 0.00022 | 0.001017 | 0.008908 | 65535 | 0.87752 | 0.660293 |
| Fp2-C4 | 3.470212 | 0.000364 | 0.001424 | 0.007374 | 65535 | 0.881865 | 0.633571 |
| Fp2-P3 | 2.754154 | 0.003408 | 0.007087 | 0.00533 | 65535 | 0.785358 | 0.502837 |
| Fp2-P4 | 2.791897 | 0.003057 | 0.00642 | 0.004903 | 65535 | 0.795102 | 0.509728 |
| Fp2-O1 | 3.37051 | 0.000507 | 0.001776 | 0.006933 | 65535 | 0.813219 | 0.615368 |
| Fp2-O2 | 3.275487 | 0.000693 | 0.002308 | 0.007164 | 65535 | 0.754205 | 0.598019 |
| Fp2-F7 | 1.028177 | 0.152985 | 0.164753 | -0.0022 | 65535 | 0.143671 | 0.187719 |
| Fp2-F8 | 1.40669 | 0.081074 | 0.095114 | -0.0009 | 65535 | 0.30674 | 0.256825 |
| Fp2-T3 | 2.403543 | 0.008897 | 0.015068 | 0.001923 | 65535 | 0.690477 | 0.438825 |
| Fp2-T4 | 2.81722 | 0.00284 | 0.006345 | 0.003708 | 65535 | 0.68025 | 0.514352 |
| Fp2-T5 | 2.796564 | 0.003016 | 0.006409 | 0.004313 | 65535 | 0.751455 | 0.51058 |
| Fp2-T6 | 3.900498 | 8E-05 | 0.000542 | 0.011651 | 65535 | 0.863516 | 0.71213 |
| Fp2-Fz | 0.531525 | 0.298027 | 0.303814 | -0.00381 | 65535 | 0.099627 | 0.097043 |
| Fp2-Cz | 0.822196 | 0.206312 | 0.216628 | -0.00392 | 65535 | 0.159359 | 0.150112 |
| Fp2-Pz | 2.442966 | 0.008024 | 0.013831 | 0.002756 | 65535 | 0.569038 | 0.446022 |
| Fp2-F9 | 1.982124 | 0.024895 | 0.034853 | 0.000842 | 65535 | 0.459391 | 0.361885 |
| Fp2-F10 | 2.67371 | 0.004283 | 0.008817 | 0.003269 | 65535 | 0.609282 | 0.48815 |
| F3-F4 | 0.164337 | 0.434873 | 0.434873 | -0.0053 | 65535 | 0.053304 | 0.030004 |
| F3-C3 | 1.763148 | 0.040232 | 0.051517 | 0.000683 | 65535 | 0.329153 | 0.321905 |
| F3-C4 | 1.046626 | 0.148706 | 0.16097 | -0.0021 | 65535 | 0.183433 | 0.191087 |
| F3-P3 | 1.921373 | 0.028548 | 0.038678 | 0.001306 | 65535 | 0.527986 | 0.350793 |
| F3-P4 | 1.814433 | 0.036076 | 0.047647 | 0.000621 | 65535 | 0.455391 | 0.331269 |
| F3-O1 | 1.376859 | 0.085581 | 0.099293 | -0.00104 | 65535 | 0.283775 | 0.251379 |
| F3-O2 | 1.211786 | 0.114008 | 0.126675 | -0.00144 | 65535 | 0.189901 | 0.221241 |
| F3-F7 | 1.019286 | 0.155076 | 0.166153 | -0.00189 | 65535 | 0.174781 | 0.186095 |
| F3-F8 | 1.241556 | 0.108431 | 0.121768 | -0.00128 | 65535 | 0.237207 | 0.226676 |
| F3-T3 | 2.442438 | 0.008035 | 0.013831 | 0.00234 | 65535 | 0.551958 | 0.445926 |
| F3-T4 | 1.701778 | 0.045715 | 0.056472 | 0.000102 | 65535 | 0.344486 | 0.310701 |
| F3-T5 | 2.280724 | 0.01218 | 0.019675 | 0.002393 | 65535 | 0.544966 | 0.416401 |
| F3-T6 | 0.779072 | 0.218749 | 0.227412 | -0.0032 | 65535 | 0.157134 | 0.142238 |
| F3-Fz | 0.674259 | 0.250733 | 0.256848 | -0.00434 | 65535 | 0.090715 | 0.123102 |
| F3-Cz | 0.883197 | 0.189463 | 0.200318 | -0.00317 | 65535 | 0.147226 | 0.161249 |
| F3-Pz | 1.735346 | 0.042645 | 0.053626 | 0.000255 | 65535 | 0.350063 | 0.316829 |
| F3-F9 | 0.49557 | 0.31056 | 0.315061 | -0.00385 | 65535 | 0.079333 | 0.090478 |
| F3-F10 | 2.566065 | 0.005769 | 0.010627 | 0.002379 | 65535 | 0.675692 | 0.468497 |
| F4-C3 | 2.186743 | 0.015367 | 0.023704 | 0.00172 | 65535 | 0.543916 | 0.399243 |
| F4-C4 | 2.841475 | 0.002646 | 0.005975 | 0.005874 | 65535 | 0.761285 | 0.51878 |
| F4-P3 | 3.645118 | 0.000199 | 0.000997 | 0.005501 | 65535 | 0.958963 | 0.665505 |
| F4-P4 | 1.897356 | 0.030112 | 0.040278 | 0.001105 | 65535 | 0.611349 | 0.346408 |
| F4-O1 | 2.256452 | 0.012942 | 0.020589 | 0.001738 | 65535 | 0.506605 | 0.41197 |
| F4-O2 | 1.719632 | 0.044061 | 0.05475 | 0.000177 | 65535 | 0.369925 | 0.31396 |
| F4-F7 | 0.683632 | 0.247774 | 0.255061 | -0.00322 | 65535 | 0.113454 | 0.124814 |
| F4-F8 | 1.724948 | 0.043578 | 0.054472 | 0.000242 | 65535 | 0.422141 | 0.314931 |
| F4-T3 | 1.106878 | 0.135299 | 0.147983 | -0.00166 | 65535 | 0.169426 | 0.202087 |
| F4-T4 | 2.651922 | 0.004552 | 0.009191 | 0.003478 | 65535 | 0.601099 | 0.484172 |
| F4-T5 | 3.600942 | 0.000233 | 0.001017 | 0.005172 | 65535 | 0.845711 | 0.657439 |
| F4-T6 | 1.80221 | 0.037033 | 0.048605 | 0.000574 | 65535 | 0.495745 | 0.329037 |
| F4-Fz | 1.284004 | 0.100828 | 0.114453 | -0.00129 | 65535 | 0.256832 | 0.234426 |
| F4-Cz | 1.740239 | 0.042212 | 0.053401 | 0.000335 | 65535 | 0.417147 | 0.317723 |
| F4-Pz | 2.476902 | 0.007335 | 0.012836 | 0.002869 | 65535 | 0.584286 | 0.452218 |
| F4-F9 | 1.169063 | 0.122367 | 0.13454 | -0.00124 | 65535 | 0.204042 | 0.213441 |
| F4-F10 | 1.608608 | 0.055187 | 0.066605 | -0.00016 | 65535 | 0.354751 | 0.29369 |
| C3-C4 | 2.126759 | 0.017761 | 0.026083 | 0.001745 | 65535 | 0.562262 | 0.388291 |
| C3-P3 | 3.474492 | 0.000358 | 0.001424 | 0.014623 | 65535 | 0.816603 | 0.634353 |
| C3-P4 | 2.19738 | 0.014973 | 0.02338 | 0.002246 | 65535 | 0.51344 | 0.401185 |
| C3-O1 | 2.130074 | 0.017621 | 0.026083 | 0.00222 | 65535 | 0.52941 | 0.388896 |
| C3-O2 | 1.593526 | 0.056859 | 0.06823 | -0.00025 | 65535 | 0.28108 | 0.290937 |
| C3-F7 | 3.143775 | 0.001055 | 0.003077 | 0.00597 | 65535 | 0.831531 | 0.573972 |
| C3-F8 | 3.529539 | 0.000297 | 0.001274 | 0.006867 | 65535 | 0.859707 | 0.644403 |
| C3-T3 | 2.809589 | 0.002904 | 0.006375 | 0.00347 | 65535 | 0.756904 | 0.512959 |
| C3-T4 | 2.482588 | 0.007225 | 0.012749 | 0.00276 | 65535 | 0.56828 | 0.453257 |
| C3-T5 | 3.337623 | 0.000565 | 0.001946 | 0.012823 | 65535 | 0.774061 | 0.609364 |
| C3-T6 | 3.005887 | 0.001618 | 0.004143 | 0.004444 | 65535 | 0.823338 | 0.548797 |
| C3-Fz | 1.251497 | 0.106614 | 0.120371 | -0.002 | 65535 | 0.202264 | 0.228491 |
| C3-Cz | 3.128289 | 0.001108 | 0.003101 | 0.008309 | 65535 | 0.749395 | 0.571145 |
| C3-Pz | 2.88194 | 0.002348 | 0.00548 | 0.005022 | 65535 | 0.73895 | 0.526168 |
| C3-F9 | 3.407486 | 0.000449 | 0.001653 | 0.007068 | 65535 | 0.826468 | 0.622119 |
| C3-F10 | 2.127782 | 0.017718 | 0.026083 | 0.001288 | 65535 | 0.714567 | 0.388478 |
| C4-P3 | 3.184692 | 0.000927 | 0.002741 | 0.006148 | 65535 | 0.811416 | 0.581443 |
| C4-P4 | 2.58113 | 0.005536 | 0.010288 | 0.005691 | 65535 | 0.628385 | 0.471248 |
| C4-O1 | 1.946062 | 0.027012 | 0.037186 | 0.001133 | 65535 | 0.355349 | 0.355301 |
| C4-O2 | 3.397575 | 0.000464 | 0.001679 | 0.008071 | 65535 | 0.794134 | 0.620309 |
| C4-F7 | 3.251363 | 0.000749 | 0.002383 | 0.004832 | 65535 | 0.81395 | 0.593615 |
| C4-F8 | 2.983428 | 0.001732 | 0.004383 | 0.005485 | 65535 | 0.775451 | 0.544697 |
| C4-T3 | 2.807796 | 0.002919 | 0.006375 | 0.003194 | 65535 | 0.656801 | 0.512631 |
| C4-T4 | 2.874492 | 0.002401 | 0.00554 | 0.005119 | 65535 | 0.691883 | 0.524808 |
| C4-T5 | 3.51539 | 0.000312 | 0.001294 | 0.006871 | 65535 | 0.841893 | 0.64182 |
| C4-T6 | 3.684704 | 0.000174 | 0.000911 | 0.011084 | 65535 | 0.926886 | 0.672732 |
| C4-Fz | 0.881852 | 0.189825 | 0.200318 | -0.0037 | 65535 | 0.126757 | 0.161003 |
| C4-Cz | 2.320344 | 0.01102 | 0.018367 | 0.003373 | 65535 | 0.58585 | 0.423635 |
| C4-Pz | 2.643362 | 0.004662 | 0.009222 | 0.00427 | 65535 | 0.625158 | 0.48261 |
| C4-F9 | 3.218717 | 0.000832 | 0.002531 | 0.004878 | 65535 | 0.82334 | 0.587655 |
| C4-F10 | 4.122151 | 3.5E-05 | 0.000409 | 0.010213 | 65535 | 0.932725 | 0.752598 |
| P3-P4 | 2.622131 | 0.004945 | 0.009355 | 0.005798 | 65535 | 0.700607 | 0.478733 |
| P3-O1 | 3.633871 | 0.000207 | 0.001013 | 0.011127 | 65535 | 0.842063 | 0.663451 |
| P3-O2 | 3.608707 | 0.000226 | 0.001017 | 0.008374 | 65535 | 0.927826 | 0.658857 |
| P3-F7 | 2.302473 | 0.011531 | 0.018865 | 0.003119 | 65535 | 0.623135 | 0.420372 |
| P3-F8 | 4.299855 | 1.77E-05 | 0.000286 | 0.009096 | 65535 | 0.985103 | 0.785042 |
| P3-T3 | 2.071567 | 0.020243 | 0.029317 | 0.002116 | 65535 | 0.373613 | 0.378215 |
| P3-T4 | 3.874526 | 8.8E-05 | 0.000552 | 0.007044 | 65535 | 0.936074 | 0.707388 |
| P3-T5 | 4.168503 | 2.94E-05 | 0.000385 | 0.020401 | 65535 | 0.92939 | 0.761061 |
| P3-T6 | 2.552341 | 0.005988 | 0.010935 | 0.004254 | 65535 | 0.831575 | 0.465991 |
| P3-Fz | 2.403762 | 0.008892 | 0.015068 | 0.00301 | 65535 | 0.506713 | 0.438865 |
| P3-Cz | 2.127238 | 0.017741 | 0.026083 | 0.002911 | 65535 | 0.500342 | 0.388379 |
| P3-Pz | 3.119426 | 0.001139 | 0.003147 | 0.009051 | 65535 | 0.753381 | 0.569527 |
| P3-F9 | 3.870038 | 8.94E-05 | 0.000552 | 0.010939 | 65535 | 0.909787 | 0.706569 |
| P3-F10 | 3.461638 | 0.000374 | 0.001429 | 0.005595 | 65535 | 0.922047 | 0.632006 |
| P4-O1 | 4.153037 | 3.12E-05 | 0.000385 | 0.012984 | 65535 | 0.914234 | 0.758237 |
| P4-O2 | 4.219048 | 2.42E-05 | 0.000339 | 0.018456 | 65535 | 0.898745 | 0.770289 |
| P4-F7 | 3.798341 | 0.000116 | 0.000658 | 0.010156 | 65535 | 0.869511 | 0.693479 |
| P4-F8 | 4.041638 | 4.75E-05 | 0.000449 | 0.012063 | 65535 | 0.973387 | 0.737899 |
| P4-T3 | 2.851991 | 0.002565 | 0.005856 | 0.003848 | 65535 | 0.703763 | 0.5207 |
| P4-T4 | 2.652863 | 0.00454 | 0.009191 | 0.004698 | 65535 | 0.574301 | 0.484344 |
| P4-T5 | 3.881757 | 8.57E-05 | 0.000552 | 0.009165 | 65535 | 0.93258 | 0.708709 |
| P4-T6 | 4.073896 | 4.21E-05 | 0.000449 | 0.023621 | 65535 | 0.914185 | 0.743788 |
| P4-Fz | 1.182458 | 0.119701 | 0.132301 | -0.00204 | 65535 | 0.284423 | 0.215886 |
| P4-Cz | 3.112186 | 0.001165 | 0.003177 | 0.008803 | 65535 | 0.754743 | 0.568205 |
| P4-Pz | 3.993334 | 5.68E-05 | 0.000459 | 0.014902 | 65535 | 0.898876 | 0.72908 |
| P4-F9 | 3.052345 | 0.001403 | 0.003777 | 0.004472 | 65535 | 0.843787 | 0.557279 |
| P4-F10 | 4.467201 | 9.14E-06 | 0.000246 | 0.016418 | 65535 | 0.954052 | 0.815596 |
| O1-O2 | 3.955303 | 6.54E-05 | 0.000482 | 0.009816 | 65535 | 0.939569 | 0.722136 |
| O1-F7 | 2.300485 | 0.011589 | 0.018865 | 0.002077 | 65535 | 0.603766 | 0.420009 |
| O1-F8 | 4.697715 | 3.59E-06 | 0.000172 | 0.009728 | 65535 | 0.975611 | 0.857681 |
| O1-T3 | 3.245999 | 0.000762 | 0.002383 | 0.004769 | 65535 | 0.76799 | 0.592636 |
| O1-T4 | 4.329356 | 1.58E-05 | 0.000276 | 0.007884 | 65535 | 0.951418 | 0.790429 |
| O1-T5 | 4.016383 | 5.22E-05 | 0.000449 | 0.013198 | 65535 | 0.921285 | 0.733288 |
| O1-T6 | 4.633162 | 4.67E-06 | 0.000172 | 0.013365 | 65535 | 0.954935 | 0.845896 |
| O1-Fz | 1.393336 | 0.083069 | 0.096913 | -0.00075 | 65535 | 0.234052 | 0.254387 |
| O1-Cz | 2.006654 | 0.023536 | 0.033396 | 0.001527 | 65535 | 0.41369 | 0.366363 |
| O1-Pz | 3.810061 | 0.000111 | 0.000658 | 0.009575 | 65535 | 0.91337 | 0.695619 |
| O1-F9 | 3.601868 | 0.000232 | 0.001017 | 0.007336 | 65535 | 0.839452 | 0.657608 |
| O1-F10 | 3.702068 | 0.000163 | 0.000879 | 0.00627 | 65535 | 0.919179 | 0.675902 |
| O2-F7 | 2.940737 | 0.001971 | 0.004757 | 0.00372 | 65535 | 0.808447 | 0.536903 |
| O2-F8 | 5.062486 | 7.71E-07 | 0.000159 | 0.011635 | 65535 | 0.994688 | 0.924279 |
| O2-T3 | 2.961452 | 0.001851 | 0.004629 | 0.005345 | 65535 | 0.645413 | 0.540685 |
| O2-T4 | 2.636245 | 0.004755 | 0.009243 | 0.003754 | 65535 | 0.612416 | 0.48131 |
| O2-T5 | 3.244738 | 0.000765 | 0.002383 | 0.007096 | 65535 | 0.793001 | 0.592405 |
| O2-T6 | 3.289523 | 0.000662 | 0.002241 | 0.011401 | 65535 | 0.740725 | 0.600582 |
| O2-Fz | 0.793737 | 0.214471 | 0.224074 | -0.00284 | 65535 | 0.112811 | 0.144916 |
| O2-Cz | 1.998709 | 0.023969 | 0.033782 | 0.001715 | 65535 | 0.420199 | 0.364913 |
| O2-Pz | 3.196367 | 0.000893 | 0.002679 | 0.0076 | 65535 | 0.754694 | 0.583574 |
| O2-F9 | 2.509642 | 0.00672 | 0.012062 | 0.002685 | 65535 | 0.570846 | 0.458196 |
| O2-F10 | 4.251896 | 2.13E-05 | 0.00032 | 0.010032 | 65535 | 0.918236 | 0.776286 |
| F7-F8 | 1.977673 | 0.025148 | 0.034974 | 0.001003 | 65535 | 0.606724 | 0.361072 |
| F7-T3 | 3.024586 | 0.001528 | 0.003961 | 0.004427 | 65535 | 0.705861 | 0.552211 |
| F7-T4 | 3.941451 | 6.89E-05 | 0.000482 | 0.006852 | 65535 | 0.915005 | 0.719607 |
| F7-T5 | 3.430121 | 0.000416 | 0.00156 | 0.006866 | 65535 | 0.876146 | 0.626251 |
| F7-T6 | 3.042216 | 0.001447 | 0.003848 | 0.006946 | 65535 | 0.671352 | 0.55543 |
| F7-Fz | 1.789287 | 0.038067 | 0.049346 | 0.000438 | 65535 | 0.377152 | 0.326678 |
| F7-Cz | 1.610351 | 0.054997 | 0.066605 | -0.00017 | 65535 | 0.342114 | 0.294008 |
| F7-Pz | 2.313774 | 0.011205 | 0.018529 | 0.001937 | 65535 | 0.526886 | 0.422435 |
| F7-F9 | 2.195832 | 0.01503 | 0.02338 | 0.001595 | 65535 | 0.568394 | 0.400902 |
| F7-F10 | 2.950829 | 0.001912 | 0.004723 | 0.003827 | 65535 | 0.825968 | 0.538745 |
| F8-T3 | 2.804827 | 0.002945 | 0.006375 | 0.003186 | 65535 | 0.714658 | 0.512089 |
| F8-T4 | 3.4681 | 0.000366 | 0.001424 | 0.006712 | 65535 | 0.840442 | 0.633186 |
| F8-T5 | 3.944851 | 6.8E-05 | 0.000482 | 0.007782 | 65535 | 0.959467 | 0.720228 |
| F8-T6 | 4.36323 | 1.38E-05 | 0.000264 | 0.01296 | 65535 | 0.964662 | 0.796613 |
| F8-Fz | 2.496785 | 0.006956 | 0.012379 | 0.00252 | 65535 | 0.643968 | 0.455848 |
| F8-Cz | 1.902262 | 0.029787 | 0.040098 | 0.000825 | 65535 | 0.524399 | 0.347304 |
| F8-Pz | 1.792821 | 0.037782 | 0.049281 | 0.000416 | 65535 | 0.391521 | 0.327323 |
| F8-F9 | 4.052912 | 4.55E-05 | 0.000449 | 0.006107 | 65535 | 0.942182 | 0.739957 |
| F8-F10 | 2.633059 | 0.004797 | 0.009243 | 0.002908 | 65535 | 0.762195 | 0.480729 |
| T3-T4 | 2.891014 | 0.002286 | 0.005394 | 0.003422 | 65535 | 0.78254 | 0.527825 |
| T3-T5 | 4.461368 | 9.36E-06 | 0.000246 | 0.013835 | 65535 | 0.947256 | 0.814531 |
| T3-T6 | 3.385734 | 0.000482 | 0.001717 | 0.006912 | 65535 | 0.774292 | 0.618148 |
| T3-Fz | 0.690698 | 0.245556 | 0.254023 | -0.00286 | 65535 | 0.097044 | 0.126104 |
| T3-Cz | 2.166168 | 0.016155 | 0.024406 | 0.00182 | 65535 | 0.461892 | 0.395486 |
| T3-Pz | 2.114284 | 0.018298 | 0.026685 | 0.001276 | 65535 | 0.42584 | 0.386014 |
| T3-F9 | 2.940748 | 0.001971 | 0.004757 | 0.004286 | 65535 | 0.652115 | 0.536905 |
| T3-F10 | 4.807949 | 2.27E-06 | 0.000159 | 0.008724 | 65535 | 0.981759 | 0.877807 |
| T4-T5 | 2.625225 | 0.004903 | 0.009355 | 0.00284 | 65535 | 0.752307 | 0.479298 |
| T4-T6 | 4.023021 | 5.09E-05 | 0.000449 | 0.011992 | 65535 | 0.937161 | 0.7345 |
| T4-Fz | 1.550736 | 0.061822 | 0.073764 | -0.00036 | 65535 | 0.25934 | 0.283124 |
| T4-Cz | 1.779818 | 0.03884 | 0.050039 | 0.000404 | 65535 | 0.403236 | 0.324949 |
| T4-Pz | 3.611859 | 0.000224 | 0.001017 | 0.005469 | 65535 | 0.891555 | 0.659432 |
| T4-F9 | 1.941826 | 0.027271 | 0.037187 | 0.000701 | 65535 | 0.464295 | 0.354527 |
| T4-F10 | 3.139335 | 0.00107 | 0.003078 | 0.005427 | 65535 | 0.816647 | 0.573162 |
| T5-T6 | 2.582761 | 0.005511 | 0.010288 | 0.004145 | 65535 | 0.8503 | 0.471545 |
| T5-Fz | 2.063183 | 0.020644 | 0.029694 | 0.001094 | 65535 | 0.460867 | 0.376684 |
| T5-Cz | 1.218369 | 0.112757 | 0.125952 | -0.00215 | 65535 | 0.214425 | 0.222443 |
| T5-Pz | 4.832057 | 2.05E-06 | 0.000159 | 0.015314 | 65535 | 0.967155 | 0.882209 |
| T5-F9 | 3.777711 | 0.000125 | 0.00069 | 0.00724 | 65535 | 0.89736 | 0.689713 |
| T5-F10 | 4.375307 | 1.32E-05 | 0.000264 | 0.008995 | 65535 | 0.983689 | 0.798818 |
| T6-Fz | 1.359389 | 0.088308 | 0.101894 | -0.00105 | 65535 | 0.356785 | 0.248189 |
| T6-Cz | 2.184156 | 0.015464 | 0.023704 | 0.002441 | 65535 | 0.516393 | 0.39877 |
| T6-Pz | 4.426027 | 1.08E-05 | 0.000251 | 0.014075 | 65535 | 0.94111 | 0.808078 |
| T6-F9 | 3.64745 | 0.000198 | 0.000997 | 0.007707 | 65535 | 0.927168 | 0.66593 |
| T6-F10 | 4.621029 | 4.91E-06 | 0.000172 | 0.013377 | 65535 | 0.943999 | 0.843681 |
| Fz-Cz | 0.34477 | 0.365441 | 0.368954 | -0.00659 | 65535 | 0.070231 | 0.062946 |
| Fz-Pz | 1.75859 | 0.04062 | 0.051698 | 0.000433 | 65535 | 0.326767 | 0.321073 |
| Fz-F9 | 1.529786 | 0.064373 | 0.075946 | -0.00038 | 65535 | 0.30524 | 0.279299 |
| Fz-F10 | 1.076087 | 0.142042 | 0.154553 | -0.00154 | 65535 | 0.169975 | 0.196466 |
| Cz-Pz | 1.312161 | 0.096006 | 0.110171 | -0.00179 | 65535 | 0.204928 | 0.239567 |
| Cz-F9 | 0.918803 | 0.180036 | 0.191917 | -0.00223 | 65535 | 0.150642 | 0.16775 |
| Cz-F10 | 2.204408 | 0.014718 | 0.023239 | 0.001659 | 65535 | 0.543184 | 0.402468 |
| Pz-F9 | 2.640536 | 0.004699 | 0.009222 | 0.002669 | 65535 | 0.673224 | 0.482094 |
| Pz-F10 | 2.054812 | 0.021053 | 0.030075 | 0.001155 | 65535 | 0.407088 | 0.375156 |
| F9-F10 | 3.133216 | 0.001091 | 0.003095 | 0.004301 | 65535 | 0.856613 | 0.572044 |

Table S4d: beta_wpli_PT_HC

| channel | tvalue | pvalue | fdr_pvalue | CI_low | CI_high | statistical power | effect size |
| --- | --- | --- | --- | --- | --- | --- | --- |
| Fp1-Fp2 | 2.644812 | 0.995357 | 0.999999 | 65535 | 0.012813 | 0.660736 | 0.482874 |
| Fp1-F3 | 1.300235 | 0.901973 | 0.999999 | 65535 | 0.009462 | 0.244873 | 0.237389 |
| Fp1-F4 | 1.944744 | 0.972907 | 0.999999 | 65535 | 0.011317 | 0.506132 | 0.35506 |
| Fp1-C3 | 4.009975 | 0.999947 | 0.999999 | 65535 | 0.023403 | 0.913025 | 0.732118 |
| Fp1-C4 | 3.269478 | 0.999294 | 0.999999 | 65535 | 0.02013 | 0.822585 | 0.596922 |
| Fp1-P3 | 2.180105 | 0.984382 | 0.999999 | 65535 | 0.016207 | 0.540896 | 0.398031 |
| Fp1-P4 | 3.513265 | 0.999686 | 0.999999 | 65535 | 0.025677 | 0.88767 | 0.641431 |
| Fp1-O1 | 2.795978 | 0.996979 | 0.999999 | 65535 | 0.01774 | 0.672841 | 0.510473 |
| Fp1-O2 | 4.023407 | 0.999949 | 0.999999 | 65535 | 0.021938 | 0.916839 | 0.73457 |
| Fp1-F7 | 2.540657 | 0.993819 | 0.999999 | 65535 | 0.013254 | 0.579366 | 0.463858 |
| Fp1-F8 | 1.8802 | 0.968727 | 0.999999 | 65535 | 0.013411 | 0.546916 | 0.343276 |
| Fp1-T3 | 2.362171 | 0.990097 | 0.999999 | 65535 | 0.011808 | 0.511137 | 0.431271 |
| Fp1-T4 | 3.242037 | 0.999228 | 0.999999 | 65535 | 0.013403 | 0.836942 | 0.591912 |
| Fp1-T5 | 3.031004 | 0.998502 | 0.999999 | 65535 | 0.016209 | 0.755351 | 0.553383 |
| Fp1-T6 | 3.970029 | 0.999938 | 0.999999 | 65535 | 0.031064 | 0.868274 | 0.724825 |
| Fp1-Fz | 2.265082 | 0.987334 | 0.999999 | 65535 | 0.013116 | 0.669867 | 0.413545 |
| Fp1-Cz | 1.632202 | 0.947349 | 0.999999 | 65535 | 0.015426 | 0.3746 | 0.297998 |
| Fp1-Pz | 3.803057 | 0.999886 | 0.999999 | 65535 | 0.018739 | 0.873294 | 0.69434 |
| Fp1-F9 | 1.536263 | 0.936425 | 0.999999 | 65535 | 0.010068 | 0.309488 | 0.280482 |
| Fp1-F10 | 2.898908 | 0.997767 | 0.999999 | 65535 | 0.016483 | 0.683383 | 0.529266 |
| Fp2-F3 | 0.304123 | 0.619215 | 0.999999 | 65535 | 0.006568 | 0.061333 | 0.055525 |
| Fp2-F4 | 1.639295 | 0.948092 | 0.999999 | 65535 | 0.009471 | 0.392937 | 0.299293 |
| Fp2-C3 | 3.616573 | 0.99978 | 0.999999 | 65535 | 0.023989 | 0.87752 | 0.660293 |
| Fp2-C4 | 3.470212 | 0.999636 | 0.999999 | 65535 | 0.020864 | 0.881865 | 0.633571 |
| Fp2-P3 | 2.754154 | 0.996592 | 0.999999 | 65535 | 0.021451 | 0.785358 | 0.502837 |
| Fp2-P4 | 2.791897 | 0.996943 | 0.999999 | 65535 | 0.019239 | 0.795102 | 0.509728 |
| Fp2-O1 | 3.37051 | 0.999493 | 0.999999 | 65535 | 0.020354 | 0.813219 | 0.615368 |
| Fp2-O2 | 3.275487 | 0.999307 | 0.999999 | 65535 | 0.02185 | 0.754205 | 0.598019 |
| Fp2-F7 | 1.028177 | 0.847015 | 0.999999 | 65535 | 0.009379 | 0.143671 | 0.187719 |
| Fp2-F8 | 1.40669 | 0.918926 | 0.999999 | 65535 | 0.010969 | 0.30674 | 0.256825 |
| Fp2-T3 | 2.403543 | 0.991103 | 0.999999 | 65535 | 0.010476 | 0.690477 | 0.438825 |
| Fp2-T4 | 2.81722 | 0.99716 | 0.999999 | 65535 | 0.014313 | 0.68025 | 0.514352 |
| Fp2-T5 | 2.796564 | 0.996984 | 0.999999 | 65535 | 0.016871 | 0.751455 | 0.51058 |
| Fp2-T6 | 3.900498 | 0.99992 | 0.999999 | 65535 | 0.028877 | 0.863516 | 0.71213 |
| Fp2-Fz | 0.531525 | 0.701973 | 0.999999 | 65535 | 0.0074 | 0.099627 | 0.097043 |
| Fp2-Cz | 0.822196 | 0.793688 | 0.999999 | 65535 | 0.011623 | 0.159359 | 0.150112 |
| Fp2-Pz | 2.442966 | 0.991976 | 0.999999 | 65535 | 0.014395 | 0.569038 | 0.446022 |
| Fp2-F9 | 1.982124 | 0.975105 | 0.999999 | 65535 | 0.009448 | 0.459391 | 0.361885 |
| Fp2-F10 | 2.67371 | 0.995717 | 0.999999 | 65535 | 0.01394 | 0.609282 | 0.48815 |
| F3-F4 | 0.164337 | 0.565127 | 0.999999 | 65535 | 0.006468 | 0.053304 | 0.030004 |
| F3-C3 | 1.763148 | 0.959768 | 0.999999 | 65535 | 0.022208 | 0.329153 | 0.321905 |
| F3-C4 | 1.046626 | 0.851294 | 0.999999 | 65535 | 0.00931 | 0.183433 | 0.191087 |
| F3-P3 | 1.921373 | 0.971452 | 0.999999 | 65535 | 0.01774 | 0.527986 | 0.350793 |
| F3-P4 | 1.814433 | 0.963924 | 0.999999 | 65535 | 0.013773 | 0.455391 | 0.331269 |
| F3-O1 | 1.376859 | 0.914419 | 0.999999 | 65535 | 0.011219 | 0.283775 | 0.251379 |
| F3-O2 | 1.211786 | 0.885992 | 0.999999 | 65535 | 0.009287 | 0.189901 | 0.221241 |
| F3-F7 | 1.019286 | 0.844924 | 0.999999 | 65535 | 0.007904 | 0.174781 | 0.186095 |
| F3-F8 | 1.241556 | 0.891569 | 0.999999 | 65535 | 0.008897 | 0.237207 | 0.226676 |
| F3-T3 | 2.442438 | 0.991965 | 0.999999 | 65535 | 0.012229 | 0.551958 | 0.445926 |
| F3-T4 | 1.701778 | 0.954285 | 0.999999 | 65535 | 0.00781 | 0.344486 | 0.310701 |
| F3-T5 | 2.280724 | 0.98782 | 0.999999 | 65535 | 0.015131 | 0.544966 | 0.416401 |
| F3-T6 | 0.779072 | 0.781251 | 0.999999 | 65535 | 0.008885 | 0.157134 | 0.142238 |
| F3-Fz | 0.674259 | 0.749267 | 0.999999 | 65535 | 0.010292 | 0.090715 | 0.123102 |
| F3-Cz | 0.883197 | 0.810537 | 0.999999 | 65535 | 0.010406 | 0.147226 | 0.161249 |
| F3-Pz | 1.735346 | 0.957355 | 0.999999 | 65535 | 0.011172 | 0.350063 | 0.316829 |
| F3-F9 | 0.49557 | 0.68944 | 0.999999 | 65535 | 0.007125 | 0.079333 | 0.090478 |
| F3-F10 | 2.566065 | 0.994231 | 0.999999 | 65535 | 0.011063 | 0.675692 | 0.468497 |
| F4-C3 | 2.186743 | 0.984633 | 0.999999 | 65535 | 0.012506 | 0.543916 | 0.399243 |
| F4-C4 | 2.841475 | 0.997354 | 0.999999 | 65535 | 0.022328 | 0.761285 | 0.51878 |
| F4-P3 | 3.645118 | 0.999801 | 0.999999 | 65535 | 0.014679 | 0.958963 | 0.665505 |
| F4-P4 | 1.897356 | 0.969888 | 0.999999 | 65535 | 0.016405 | 0.611349 | 0.346408 |
| F4-O1 | 2.256452 | 0.987058 | 0.999999 | 65535 | 0.011363 | 0.506605 | 0.41197 |
| F4-O2 | 1.719632 | 0.955939 | 0.999999 | 65535 | 0.009676 | 0.369925 | 0.31396 |
| F4-F7 | 0.683632 | 0.752226 | 0.999999 | 65535 | 0.00774 | 0.113454 | 0.124814 |
| F4-F8 | 1.724948 | 0.956422 | 0.999999 | 65535 | 0.012223 | 0.422141 | 0.314931 |
| F4-T3 | 1.106878 | 0.864701 | 0.999999 | 65535 | 0.008349 | 0.169426 | 0.202087 |
| F4-T4 | 2.651922 | 0.995448 | 0.999999 | 65535 | 0.015081 | 0.601099 | 0.484172 |
| F4-T5 | 3.600942 | 0.999767 | 0.999999 | 65535 | 0.013998 | 0.845711 | 0.657439 |
| F4-T6 | 1.80221 | 0.962967 | 0.999999 | 65535 | 0.013761 | 0.495745 | 0.329037 |
| F4-Fz | 1.284004 | 0.899172 | 0.999999 | 65535 | 0.010116 | 0.256832 | 0.234426 |
| F4-Cz | 1.740239 | 0.957788 | 0.999999 | 65535 | 0.013816 | 0.417147 | 0.317723 |
| F4-Pz | 2.476902 | 0.992665 | 0.999999 | 65535 | 0.014484 | 0.584286 | 0.452218 |
| F4-F9 | 1.169063 | 0.877633 | 0.999999 | 65535 | 0.007176 | 0.204042 | 0.213441 |
| F4-F10 | 1.608608 | 0.944813 | 0.999999 | 65535 | 0.010467 | 0.354751 | 0.29369 |
| C3-C4 | 2.126759 | 0.982239 | 0.999999 | 65535 | 0.014086 | 0.562262 | 0.388291 |
| C3-P3 | 3.474492 | 0.999642 | 0.999999 | 65535 | 0.041312 | 0.816603 | 0.634353 |
| C3-P4 | 2.19738 | 0.985027 | 0.999999 | 65535 | 0.016049 | 0.51344 | 0.401185 |
| C3-O1 | 2.130074 | 0.982379 | 0.999999 | 65535 | 0.017805 | 0.52941 | 0.388896 |
| C3-O2 | 1.593526 | 0.943141 | 0.999999 | 65535 | 0.012634 | 0.28108 | 0.290937 |
| C3-F7 | 3.143775 | 0.998945 | 0.999999 | 65535 | 0.019291 | 0.831531 | 0.573972 |
| C3-F8 | 3.529539 | 0.999703 | 0.999999 | 65535 | 0.019033 | 0.859707 | 0.644403 |
| C3-T3 | 2.809589 | 0.997096 | 0.999999 | 65535 | 0.013458 | 0.756904 | 0.512959 |
| C3-T4 | 2.482588 | 0.992775 | 0.999999 | 65535 | 0.013858 | 0.56828 | 0.453257 |
| C3-T5 | 3.337623 | 0.999435 | 0.999999 | 65535 | 0.038135 | 0.774061 | 0.609364 |
| C3-T6 | 3.005887 | 0.998382 | 0.999999 | 65535 | 0.015375 | 0.823338 | 0.548797 |
| C3-Fz | 1.251497 | 0.893386 | 0.999999 | 65535 | 0.014302 | 0.202264 | 0.228491 |
| C3-Cz | 3.128289 | 0.998892 | 0.999999 | 65535 | 0.027046 | 0.749395 | 0.571145 |
| C3-Pz | 2.88194 | 0.997652 | 0.999999 | 65535 | 0.018624 | 0.73895 | 0.526168 |
| C3-F9 | 3.407486 | 0.999551 | 0.999999 | 65535 | 0.020464 | 0.826468 | 0.622119 |
| C3-F10 | 2.127782 | 0.982282 | 0.999999 | 65535 | 0.010372 | 0.714567 | 0.388478 |
| C4-P3 | 3.184692 | 0.999073 | 0.999999 | 65535 | 0.019501 | 0.811416 | 0.581443 |
| C4-P4 | 2.58113 | 0.994464 | 0.999999 | 65535 | 0.026128 | 0.628385 | 0.471248 |
| C4-O1 | 1.946062 | 0.972988 | 0.999999 | 65535 | 0.014165 | 0.355349 | 0.355301 |
| C4-O2 | 3.397575 | 0.999536 | 0.999999 | 65535 | 0.023453 | 0.794134 | 0.620309 |
| C4-F7 | 3.251363 | 0.999251 | 0.999999 | 65535 | 0.014886 | 0.81395 | 0.593615 |
| C4-F8 | 2.983428 | 0.998268 | 0.999999 | 65535 | 0.019204 | 0.775451 | 0.544697 |
| C4-T3 | 2.807796 | 0.997081 | 0.999999 | 65535 | 0.012403 | 0.656801 | 0.512631 |
| C4-T4 | 2.874492 | 0.997599 | 0.999999 | 65535 | 0.019069 | 0.691883 | 0.524808 |
| C4-T5 | 3.51539 | 0.999688 | 0.999999 | 65535 | 0.019136 | 0.841893 | 0.64182 |
| C4-T6 | 3.684704 | 0.999826 | 0.999999 | 65535 | 0.029216 | 0.926886 | 0.672732 |
| C4-Fz | 0.881852 | 0.810175 | 0.999999 | 65535 | 0.012095 | 0.126757 | 0.161003 |
| C4-Cz | 2.320344 | 0.98898 | 0.999999 | 65535 | 0.020255 | 0.58585 | 0.423635 |
| C4-Pz | 2.643362 | 0.995338 | 0.999999 | 65535 | 0.018636 | 0.625158 | 0.48261 |
| C4-F9 | 3.218717 | 0.999168 | 0.999999 | 65535 | 0.01524 | 0.82334 | 0.587655 |
| C4-F10 | 4.122151 | 0.999965 | 0.999999 | 65535 | 0.023954 | 0.932725 | 0.752598 |
| P3-P4 | 2.622131 | 0.995055 | 0.999999 | 65535 | 0.025737 | 0.700607 | 0.478733 |
| P3-O1 | 3.633871 | 0.999793 | 0.999999 | 65535 | 0.029797 | 0.842063 | 0.663451 |
| P3-O2 | 3.608707 | 0.999774 | 0.999999 | 65535 | 0.022607 | 0.927826 | 0.658857 |
| P3-F7 | 2.302473 | 0.988469 | 0.999999 | 65535 | 0.019161 | 0.623135 | 0.420372 |
| P3-F8 | 4.299855 | 0.999982 | 0.999999 | 65535 | 0.020511 | 0.985103 | 0.785042 |
| P3-T3 | 2.071567 | 0.979757 | 0.999999 | 65535 | 0.019079 | 0.373613 | 0.378215 |
| P3-T4 | 3.874526 | 0.999912 | 0.999999 | 65535 | 0.017579 | 0.936074 | 0.707388 |
| P3-T5 | 4.168503 | 0.999971 | 0.999999 | 65535 | 0.047343 | 0.92939 | 0.761061 |
| P3-T6 | 2.552341 | 0.994012 | 0.999999 | 65535 | 0.020024 | 0.831575 | 0.465991 |
| P3-Fz | 2.403762 | 0.991108 | 0.999999 | 65535 | 0.01639 | 0.506713 | 0.438865 |
| P3-Cz | 2.127238 | 0.982259 | 0.999999 | 65535 | 0.023475 | 0.500342 | 0.388379 |
| P3-Pz | 3.119426 | 0.998861 | 0.999999 | 65535 | 0.029583 | 0.753381 | 0.569527 |
| P3-F9 | 3.870038 | 0.999911 | 0.999999 | 65535 | 0.027334 | 0.909787 | 0.706569 |
| P3-F10 | 3.461638 | 0.999626 | 0.999999 | 65535 | 0.01588 | 0.922047 | 0.632006 |
| P4-O1 | 4.153037 | 0.999969 | 0.999999 | 65535 | 0.030238 | 0.914234 | 0.758237 |
| P4-O2 | 4.219048 | 0.999976 | 0.999999 | 65535 | 0.042349 | 0.898745 | 0.770289 |
| P4-F7 | 3.798341 | 0.999884 | 0.999999 | 65535 | 0.025889 | 0.869511 | 0.693479 |
| P4-F8 | 4.041638 | 0.999953 | 0.999999 | 65535 | 0.028843 | 0.973387 | 0.737899 |
| P4-T3 | 2.851991 | 0.997435 | 0.999999 | 65535 | 0.014533 | 0.703763 | 0.5207 |
| P4-T4 | 2.652863 | 0.99546 | 0.999999 | 65535 | 0.020355 | 0.574301 | 0.484344 |
| P4-T5 | 3.881757 | 0.999914 | 0.999999 | 65535 | 0.02283 | 0.93258 | 0.708709 |
| P4-T6 | 4.073896 | 0.999958 | 0.999999 | 65535 | 0.056038 | 0.914185 | 0.743788 |
| P4-Fz | 1.182458 | 0.880299 | 0.999999 | 65535 | 0.012174 | 0.284423 | 0.215886 |
| P4-Cz | 3.112186 | 0.998835 | 0.999999 | 65535 | 0.028874 | 0.754743 | 0.568205 |
| P4-Pz | 3.993334 | 0.999943 | 0.999999 | 65535 | 0.03606 | 0.898876 | 0.72908 |
| P4-F9 | 3.052345 | 0.998597 | 0.999999 | 65535 | 0.015104 | 0.843787 | 0.557279 |
| P4-F10 | 4.467201 | 0.999991 | 0.999999 | 65535 | 0.035795 | 0.954052 | 0.815596 |
| O1-O2 | 3.955303 | 0.999935 | 0.999999 | 65535 | 0.023983 | 0.939569 | 0.722136 |
| O1-F7 | 2.300485 | 0.988411 | 0.999999 | 65535 | 0.012793 | 0.603766 | 0.420009 |
| O1-F8 | 4.697715 | 0.999996 | 0.999999 | 65535 | 0.020339 | 0.975611 | 0.857681 |
| O1-T3 | 3.245999 | 0.999238 | 0.999999 | 65535 | 0.014725 | 0.76799 | 0.592636 |
| O1-T4 | 4.329356 | 0.999984 | 0.999999 | 65535 | 0.01767 | 0.951418 | 0.790429 |
| O1-T5 | 4.016383 | 0.999948 | 0.999999 | 65535 | 0.031752 | 0.921285 | 0.733288 |
| O1-T6 | 4.633162 | 0.999995 | 0.999999 | 65535 | 0.028259 | 0.954935 | 0.845896 |
| O1-Fz | 1.393336 | 0.916931 | 0.999999 | 65535 | 0.008685 | 0.234052 | 0.254387 |
| O1-Cz | 2.006654 | 0.976464 | 0.999999 | 65535 | 0.016041 | 0.41369 | 0.366363 |
| O1-Pz | 3.810061 | 0.999889 | 0.999999 | 65535 | 0.024327 | 0.91337 | 0.695619 |
| O1-F9 | 3.601868 | 0.999768 | 0.999999 | 65535 | 0.019848 | 0.839452 | 0.657608 |
| O1-F10 | 3.702068 | 0.999837 | 0.999999 | 65535 | 0.01644 | 0.919179 | 0.675902 |
| O2-F7 | 2.940737 | 0.998029 | 0.999999 | 65535 | 0.013333 | 0.808447 | 0.536903 |
| O2-F8 | 5.062486 | 0.999999 | 0.999999 | 65535 | 0.022965 | 0.994688 | 0.924279 |
| O2-T3 | 2.961452 | 0.998149 | 0.999999 | 65535 | 0.018939 | 0.645413 | 0.540685 |
| O2-T4 | 2.636245 | 0.995245 | 0.999999 | 65535 | 0.016478 | 0.612416 | 0.48131 |
| O2-T5 | 3.244738 | 0.999235 | 0.999999 | 65535 | 0.021923 | 0.793001 | 0.592405 |
| O2-T6 | 3.289523 | 0.999338 | 0.999999 | 65535 | 0.034569 | 0.740725 | 0.600582 |
| O2-Fz | 0.793737 | 0.785529 | 0.999999 | 65535 | 0.008049 | 0.112811 | 0.144916 |
| O2-Cz | 1.998709 | 0.976031 | 0.999999 | 65535 | 0.018397 | 0.420199 | 0.364913 |
| O2-Pz | 3.196367 | 0.999107 | 0.999999 | 65535 | 0.023979 | 0.754694 | 0.583574 |
| O2-F9 | 2.509642 | 0.99328 | 0.999999 | 65535 | 0.013139 | 0.570846 | 0.458196 |
| O2-F10 | 4.251896 | 0.999979 | 0.999999 | 65535 | 0.022855 | 0.918236 | 0.776286 |
| F7-F8 | 1.977673 | 0.974852 | 0.999999 | 65535 | 0.0114 | 0.606724 | 0.361072 |
| F7-T3 | 3.024586 | 0.998472 | 0.999999 | 65535 | 0.015166 | 0.705861 | 0.552211 |
| F7-T4 | 3.941451 | 0.999931 | 0.999999 | 65535 | 0.016801 | 0.915005 | 0.719607 |
| F7-T5 | 3.430121 | 0.999584 | 0.999999 | 65535 | 0.019713 | 0.876146 | 0.626251 |
| F7-T6 | 3.042216 | 0.998553 | 0.999999 | 65535 | 0.023582 | 0.671352 | 0.55543 |
| F7-Fz | 1.789287 | 0.961933 | 0.999999 | 65535 | 0.011479 | 0.377152 | 0.326678 |
| F7-Cz | 1.610351 | 0.945003 | 0.999999 | 65535 | 0.011474 | 0.342114 | 0.294008 |
| F7-Pz | 2.313774 | 0.988795 | 0.999999 | 65535 | 0.01173 | 0.526886 | 0.422435 |
| F7-F9 | 2.195832 | 0.98497 | 0.999999 | 65535 | 0.011429 | 0.568394 | 0.400902 |
| F7-F10 | 2.950829 | 0.998088 | 0.999999 | 65535 | 0.013641 | 0.825968 | 0.538745 |
| F8-T3 | 2.804827 | 0.997055 | 0.999999 | 65535 | 0.012398 | 0.714658 | 0.512089 |
| F8-T4 | 3.4681 | 0.999634 | 0.999999 | 65535 | 0.019005 | 0.840442 | 0.633186 |
| F8-T5 | 3.944851 | 0.999932 | 0.999999 | 65535 | 0.019065 | 0.959467 | 0.720228 |
| F8-T6 | 4.36323 | 0.999986 | 0.999999 | 65535 | 0.028844 | 0.964662 | 0.796613 |
| F8-Fz | 2.496785 | 0.993044 | 0.999999 | 65535 | 0.012478 | 0.643968 | 0.455848 |
| F8-Cz | 1.902262 | 0.970213 | 0.999999 | 65535 | 0.012018 | 0.524399 | 0.347304 |
| F8-Pz | 1.792821 | 0.962218 | 0.999999 | 65535 | 0.010627 | 0.391521 | 0.327323 |
| F8-F9 | 4.052912 | 0.999954 | 0.999999 | 65535 | 0.01456 | 0.942182 | 0.739957 |
| F8-F10 | 2.633059 | 0.995203 | 0.999999 | 65535 | 0.012797 | 0.762195 | 0.480729 |
| T3-T4 | 2.891014 | 0.997714 | 0.999999 | 65535 | 0.012623 | 0.78254 | 0.527825 |
| T3-T5 | 4.461368 | 0.999991 | 0.999999 | 65535 | 0.030198 | 0.947256 | 0.814531 |
| T3-T6 | 3.385734 | 0.999518 | 0.999999 | 65535 | 0.020176 | 0.774292 | 0.618148 |
| T3-Fz | 0.690698 | 0.754444 | 0.999999 | 65535 | 0.006943 | 0.097044 | 0.126104 |
| T3-Cz | 2.166168 | 0.983845 | 0.999999 | 65535 | 0.013689 | 0.461892 | 0.395486 |
| T3-Pz | 2.114284 | 0.981702 | 0.999999 | 65535 | 0.010547 | 0.42584 | 0.386014 |
| T3-F9 | 2.940748 | 0.998029 | 0.999999 | 65535 | 0.015364 | 0.652115 | 0.536905 |
| T3-F10 | 4.807949 | 0.999998 | 0.999999 | 65535 | 0.017906 | 0.981759 | 0.877807 |
| T4-T5 | 2.625225 | 0.995097 | 0.999999 | 65535 | 0.012574 | 0.752307 | 0.479298 |
| T4-T6 | 4.023021 | 0.999949 | 0.999999 | 65535 | 0.028804 | 0.937161 | 0.7345 |
| T4-Fz | 1.550736 | 0.938178 | 0.999999 | 65535 | 0.010818 | 0.25934 | 0.283124 |
| T4-Cz | 1.779818 | 0.96116 | 0.999999 | 65535 | 0.011402 | 0.403236 | 0.324949 |
| T4-Pz | 3.611859 | 0.999776 | 0.999999 | 65535 | 0.01475 | 0.891555 | 0.659432 |
| T4-F9 | 1.941826 | 0.972729 | 0.999999 | 65535 | 0.00889 | 0.464295 | 0.354527 |
| T4-F10 | 3.139335 | 0.99893 | 0.999999 | 65535 | 0.017574 | 0.816647 | 0.573162 |
| T5-T6 | 2.582761 | 0.994489 | 0.999999 | 65535 | 0.019006 | 0.8503 | 0.471545 |
| T5-Fz | 2.063183 | 0.979356 | 0.999999 | 65535 | 0.01004 | 0.460867 | 0.376684 |
| T5-Cz | 1.218369 | 0.887243 | 0.999999 | 65535 | 0.014076 | 0.214425 | 0.222443 |
| T5-Pz | 4.832057 | 0.999998 | 0.999999 | 65535 | 0.031311 | 0.967155 | 0.882209 |
| T5-F9 | 3.777711 | 0.999875 | 0.999999 | 65535 | 0.018564 | 0.89736 | 0.689713 |
| T5-F10 | 4.375307 | 0.999987 | 0.999999 | 65535 | 0.01997 | 0.983689 | 0.798818 |
| T6-Fz | 1.359389 | 0.911692 | 0.999999 | 65535 | 0.01057 | 0.356785 | 0.248189 |
| T6-Cz | 2.184156 | 0.984536 | 0.999999 | 65535 | 0.017816 | 0.516393 | 0.39877 |
| T6-Pz | 4.426027 | 0.999989 | 0.999999 | 65535 | 0.030934 | 0.94111 | 0.808078 |
| T6-F9 | 3.64745 | 0.999802 | 0.999999 | 65535 | 0.020552 | 0.927168 | 0.66593 |
| T6-F10 | 4.621029 | 0.999995 | 0.999999 | 65535 | 0.028346 | 0.943999 | 0.843681 |
| Fz-Cz | 0.34477 | 0.634559 | 0.999999 | 65535 | 0.010056 | 0.070231 | 0.062946 |
| Fz-Pz | 1.75859 | 0.95938 | 0.999999 | 65535 | 0.014688 | 0.326767 | 0.321073 |
| Fz-F9 | 1.529786 | 0.935627 | 0.999999 | 65535 | 0.009493 | 0.30524 | 0.279299 |
| Fz-F10 | 1.076087 | 0.857958 | 0.999999 | 65535 | 0.007215 | 0.169975 | 0.196466 |
| Cz-Pz | 1.312161 | 0.903994 | 0.999999 | 65535 | 0.015364 | 0.204928 | 0.239567 |
| Cz-F9 | 0.918803 | 0.819964 | 0.999999 | 65535 | 0.007763 | 0.150642 | 0.16775 |
| Cz-F10 | 2.204408 | 0.985282 | 0.999999 | 65535 | 0.011725 | 0.543184 | 0.402468 |
| Pz-F9 | 2.640536 | 0.995301 | 0.999999 | 65535 | 0.011673 | 0.673224 | 0.482094 |
| Pz-F10 | 2.054812 | 0.978947 | 0.999999 | 65535 | 0.010799 | 0.407088 | 0.375156 |
| F9-F10 | 3.133216 | 0.998909 | 0.999999 | 65535 | 0.013967 | 0.856613 | 0.572044 |

Table S4e: delta_wpli_HC_PT

| channel | tvalue | pvalue | fdr_pvalue | CI_low | CI_high | statistical power | effect size |
| --- | --- | --- | --- | --- | --- | --- | --- |
| Fp1-Fp2 | -1.00866 | 0.8424 | 0.99938 | -0.00831 | 65535 | 0.171975 | -0.18416 |
| Fp1-F3 | 0.982042 | 0.164044 | 0.99938 | -0.00192 | 65535 | 0.139872 | 0.179296 |
| Fp1-F4 | -0.46177 | 0.677453 | 0.99938 | -0.00693 | 65535 | 0.072687 | -0.08431 |
| Fp1-C3 | -0.11587 | 0.546025 | 0.99938 | -0.00558 | 65535 | 0.051232 | -0.02116 |
| Fp1-C4 | -1.24799 | 0.892747 | 0.99938 | -0.00907 | 65535 | 0.209857 | -0.22785 |
| Fp1-P3 | -0.4516 | 0.673809 | 0.99938 | -0.00771 | 65535 | 0.067078 | -0.08245 |
| Fp1-P4 | -0.0143 | 0.505693 | 0.99938 | -0.00575 | 65535 | 0.050021 | -0.00261 |
| Fp1-O1 | -0.22507 | 0.588844 | 0.99938 | -0.00599 | 65535 | 0.056058 | -0.04109 |
| Fp1-O2 | -1.22381 | 0.888269 | 0.99938 | -0.00923 | 65535 | 0.229625 | -0.22344 |
| Fp1-F7 | -0.63974 | 0.738208 | 0.99938 | -0.00741 | 65535 | 0.091738 | -0.1168 |
| Fp1-F8 | -0.2312 | 0.591221 | 0.99938 | -0.00666 | 65535 | 0.055028 | -0.04221 |
| Fp1-T3 | -2.15442 | 0.98338 | 0.99938 | -0.01165 | 65535 | 0.631541 | -0.39334 |
| Fp1-T4 | -1.24434 | 0.89208 | 0.99938 | -0.00873 | 65535 | 0.196418 | -0.22718 |
| Fp1-T5 | -0.62594 | 0.733718 | 0.99938 | -0.00699 | 65535 | 0.09275 | -0.11428 |
| Fp1-T6 | 0.474267 | 0.318093 | 0.99938 | -0.00331 | 65535 | 0.075694 | 0.086589 |
| Fp1-Fz | -0.89078 | 0.812569 | 0.99938 | -0.00847 | 65535 | 0.135728 | -0.16263 |
| Fp1-Cz | -0.31234 | 0.622333 | 0.99938 | -0.00684 | 65535 | 0.05873 | -0.05702 |
| Fp1-Pz | 1.327663 | 0.093426 | 0.99938 | -0.00123 | 65535 | 0.216391 | 0.242397 |
| Fp1-F9 | -1.83439 | 0.965442 | 0.99938 | -0.01127 | 65535 | 0.410502 | -0.33491 |
| Fp1-F10 | -1.19149 | 0.882075 | 0.99938 | -0.01024 | 65535 | 0.187634 | -0.21754 |
| Fp2-F3 | -0.01501 | 0.505973 | 0.99938 | -0.00516 | 65535 | 0.050026 | -0.00274 |
| Fp2-F4 | -0.64782 | 0.740821 | 0.99938 | -0.00658 | 65535 | 0.090653 | -0.11828 |
| Fp2-C3 | -0.35207 | 0.637294 | 0.99938 | -0.00572 | 65535 | 0.066706 | -0.06428 |
| Fp2-C4 | -1.32466 | 0.906079 | 0.99938 | -0.00985 | 65535 | 0.230417 | -0.24185 |
| Fp2-P3 | 0.022423 | 0.491074 | 0.99938 | -0.00652 | 65535 | 0.050039 | 0.004094 |
| Fp2-P4 | -1.00805 | 0.842252 | 0.99938 | -0.00919 | 65535 | 0.161671 | -0.18404 |
| Fp2-O1 | -0.47714 | 0.682927 | 0.99938 | -0.00699 | 65535 | 0.072331 | -0.08711 |
| Fp2-O2 | -2.6535 | 0.995468 | 0.99938 | -0.01235 | 65535 | 0.75608 | -0.48446 |
| Fp2-F7 | -0.35263 | 0.637502 | 0.99938 | -0.00704 | 65535 | 0.062876 | -0.06438 |
| Fp2-F8 | -0.10308 | 0.540961 | 0.99938 | -0.00601 | 65535 | 0.051011 | -0.01882 |
| Fp2-T3 | -2.1739 | 0.984145 | 0.99938 | -0.01087 | 65535 | 0.556759 | -0.3969 |
| Fp2-T4 | -0.72591 | 0.765334 | 0.99938 | -0.00737 | 65535 | 0.112901 | -0.13253 |
| Fp2-T5 | -1.48598 | 0.930024 | 0.99938 | -0.00941 | 65535 | 0.297527 | -0.2713 |
| Fp2-T6 | 0.581326 | 0.281065 | 0.99938 | -0.0031 | 65535 | 0.081298 | 0.106135 |
| Fp2-Fz | -0.45439 | 0.674808 | 0.99938 | -0.00587 | 65535 | 0.067208 | -0.08296 |
| Fp2-Cz | -0.07702 | 0.53063 | 0.99938 | -0.00574 | 65535 | 0.050587 | -0.01406 |
| Fp2-Pz | 0.596349 | 0.276042 | 0.99938 | -0.00453 | 65535 | 0.074983 | 0.108878 |
| Fp2-F9 | -2.48725 | 0.992865 | 0.99938 | -0.01198 | 65535 | 0.694041 | -0.45411 |
| Fp2-F10 | -1.37055 | 0.913442 | 0.99938 | -0.00963 | 65535 | 0.229137 | -0.25023 |
| F3-F4 | 0.262078 | 0.396859 | 0.99938 | -0.00424 | 65535 | 0.055364 | 0.047849 |
| F3-C3 | -1.47437 | 0.928477 | 0.99938 | -0.0089 | 65535 | 0.311051 | -0.26918 |
| F3-C4 | -0.63074 | 0.735285 | 0.99938 | -0.00755 | 65535 | 0.080538 | -0.11516 |
| F3-P3 | 0.110354 | 0.456158 | 0.99938 | -0.00591 | 65535 | 0.05085 | 0.020148 |
| F3-P4 | -0.67021 | 0.747983 | 0.99938 | -0.0077 | 65535 | 0.088612 | -0.12236 |
| F3-O1 | -0.42257 | 0.663309 | 0.99938 | -0.00587 | 65535 | 0.06842 | -0.07715 |
| F3-O2 | -0.16394 | 0.564971 | 0.99938 | -0.0052 | 65535 | 0.05219 | -0.02993 |
| F3-F7 | -0.6286 | 0.734588 | 0.99938 | -0.00696 | 65535 | 0.087799 | -0.11477 |
| F3-F8 | 0.098447 | 0.460872 | 0.99938 | -0.0051 | 65535 | 0.05097 | 0.017974 |
| F3-T3 | -0.02622 | 0.510437 | 0.99938 | -0.00512 | 65535 | 0.050059 | -0.00479 |
| F3-T4 | 0.647287 | 0.259352 | 0.99938 | -0.00338 | 65535 | 0.087412 | 0.118178 |
| F3-T5 | -1.53008 | 0.935664 | 0.99938 | -0.00918 | 65535 | 0.273811 | -0.27935 |
| F3-T6 | -0.44081 | 0.669923 | 0.99938 | -0.00606 | 65535 | 0.080377 | -0.08048 |
| F3-Fz | -1.01341 | 0.843531 | 0.99938 | -0.00841 | 65535 | 0.142649 | -0.18502 |
| F3-Cz | 0.076218 | 0.469687 | 0.99938 | -0.00453 | 65535 | 0.050653 | 0.013915 |
| F3-Pz | 1.137745 | 0.128766 | 0.99938 | -0.00205 | 65535 | 0.152844 | 0.207723 |
| F3-F9 | -0.01775 | 0.507067 | 0.99938 | -0.00564 | 65535 | 0.050026 | -0.00324 |
| F3-F10 | -0.785 | 0.782988 | 0.99938 | -0.00742 | 65535 | 0.107131 | -0.14332 |
| F4-C3 | 0.754246 | 0.226102 | 0.99938 | -0.00272 | 65535 | 0.101664 | 0.137706 |
| F4-C4 | -3.02638 | 0.99848 | 0.99938 | -0.01382 | 65535 | 0.821465 | -0.55254 |
| F4-P3 | -1.60874 | 0.944827 | 0.99938 | -0.01013 | 65535 | 0.292106 | -0.29371 |
| F4-P4 | -2.01647 | 0.976989 | 0.99938 | -0.01115 | 65535 | 0.463547 | -0.36816 |
| F4-O1 | -1.0234 | 0.845893 | 0.99938 | -0.00884 | 65535 | 0.240901 | -0.18685 |
| F4-O2 | 0.036807 | 0.485351 | 0.99938 | -0.00683 | 65535 | 0.050116 | 0.00672 |
| F4-F7 | -0.26017 | 0.602408 | 0.99938 | -0.00596 | 65535 | 0.055869 | -0.0475 |
| F4-F8 | -1.09778 | 0.862733 | 0.99938 | -0.00898 | 65535 | 0.19251 | -0.20043 |
| F4-T3 | -1.81048 | 0.963617 | 0.99938 | -0.00965 | 65535 | 0.380047 | -0.33055 |
| F4-T4 | -0.41243 | 0.659612 | 0.99938 | -0.0054 | 65535 | 0.071931 | -0.0753 |
| F4-T5 | -0.26642 | 0.604808 | 0.99938 | -0.00605 | 65535 | 0.056753 | -0.04864 |
| F4-T6 | -2.92335 | 0.997924 | 0.99938 | -0.01396 | 65535 | 0.798157 | -0.53373 |
| F4-Fz | -1.79965 | 0.962764 | 0.99938 | -0.01241 | 65535 | 0.554297 | -0.32857 |
| F4-Cz | -2.58116 | 0.994464 | 0.99938 | -0.01213 | 65535 | 0.873119 | -0.47125 |
| F4-Pz | -1.22629 | 0.888734 | 0.99938 | -0.01 | 65535 | 0.188347 | -0.22389 |
| F4-F9 | 0.353608 | 0.362132 | 0.99938 | -0.00332 | 65535 | 0.063587 | 0.06456 |
| F4-F10 | -1.86619 | 0.967751 | 0.99938 | -0.0124 | 65535 | 0.417827 | -0.34072 |
| C3-C4 | -0.93056 | 0.823008 | 0.99938 | -0.00845 | 65535 | 0.132801 | -0.1699 |
| C3-P3 | 1.242281 | 0.108298 | 0.99938 | -0.00148 | 65535 | 0.182743 | 0.226809 |
| C3-P4 | 0.075374 | 0.470022 | 0.99938 | -0.00572 | 65535 | 0.050534 | 0.013761 |
| C3-O1 | -1.24613 | 0.892408 | 0.99938 | -0.00965 | 65535 | 0.201771 | -0.22751 |
| C3-O2 | -0.89803 | 0.814502 | 0.99938 | -0.00859 | 65535 | 0.12272 | -0.16396 |
| C3-F7 | -1.32273 | 0.905758 | 0.99938 | -0.00951 | 65535 | 0.226885 | -0.2415 |
| C3-F8 | 1.479769 | 0.0708 | 0.99938 | -0.00058 | 65535 | 0.275025 | 0.270168 |
| C3-T3 | -0.60275 | 0.726083 | 0.99938 | -0.00654 | 65535 | 0.085515 | -0.11005 |
| C3-T4 | 0.013984 | 0.494433 | 0.99938 | -0.00482 | 65535 | 0.050016 | 0.002553 |
| C3-T5 | -2.29868 | 0.988358 | 0.99938 | -0.01209 | 65535 | 0.591703 | -0.41968 |
| C3-T6 | -0.09974 | 0.539642 | 0.99938 | -0.0055 | 65535 | 0.05112 | -0.01821 |
| C3-Fz | 0.021588 | 0.491406 | 0.99938 | -0.00606 | 65535 | 0.05004 | 0.003941 |
| C3-Cz | 0.662158 | 0.254581 | 0.99938 | -0.00313 | 65535 | 0.102003 | 0.120893 |
| C3-Pz | 0.962272 | 0.16894 | 0.99938 | -0.00328 | 65535 | 0.117335 | 0.175686 |
| C3-F9 | -0.70159 | 0.757842 | 0.99938 | -0.00727 | 65535 | 0.096961 | -0.12809 |
| C3-F10 | -1.79329 | 0.962256 | 0.99938 | -0.0115 | 65535 | 0.360995 | -0.32741 |
| C4-P3 | -1.51404 | 0.933655 | 0.99938 | -0.01123 | 65535 | 0.238741 | -0.27642 |
| C4-P4 | -1.20993 | 0.885639 | 0.99938 | -0.01015 | 65535 | 0.247787 | -0.2209 |
| C4-O1 | -1.59016 | 0.942763 | 0.99938 | -0.01101 | 65535 | 0.287465 | -0.29032 |
| C4-O2 | -1.43488 | 0.923017 | 0.99938 | -0.01066 | 65535 | 0.368728 | -0.26197 |
| C4-F7 | -0.99151 | 0.838267 | 0.99938 | -0.00866 | 65535 | 0.135861 | -0.18102 |
| C4-F8 | -1.04261 | 0.850369 | 0.99938 | -0.00909 | 65535 | 0.159126 | -0.19035 |
| C4-T3 | -3.06508 | 0.998651 | 0.99938 | -0.01477 | 65535 | 0.787462 | -0.55961 |
| C4-T4 | -0.04154 | 0.516534 | 0.99938 | -0.00567 | 65535 | 0.05016 | -0.00758 |
| C4-T5 | -2.88696 | 0.997686 | 0.99938 | -0.01266 | 65535 | 0.75211 | -0.52708 |
| C4-T6 | -0.99863 | 0.839991 | 0.99938 | -0.0077 | 65535 | 0.205557 | -0.18232 |
| C4-Fz | -0.81174 | 0.790713 | 0.99938 | -0.00651 | 65535 | 0.133855 | -0.1482 |
| C4-Cz | -1.83674 | 0.965618 | 0.99938 | -0.00915 | 65535 | 0.474484 | -0.33534 |
| C4-Pz | -1.28482 | 0.899314 | 0.99938 | -0.01022 | 65535 | 0.210157 | -0.23457 |
| C4-F9 | -0.3483 | 0.635883 | 0.99938 | -0.0069 | 65535 | 0.059152 | -0.06359 |
| C4-F10 | -2.54366 | 0.993869 | 0.99938 | -0.01355 | 65535 | 0.566794 | -0.46441 |
| P3-P4 | -0.14328 | 0.556843 | 0.99938 | -0.00759 | 65535 | 0.051731 | -0.02616 |
| P3-O1 | -1.61944 | 0.945989 | 0.99938 | -0.01175 | 65535 | 0.294857 | -0.29567 |
| P3-O2 | 0.41386 | 0.339864 | 0.99938 | -0.00624 | 65535 | 0.06421 | 0.07556 |
| P3-F7 | 0.764736 | 0.222978 | 0.99938 | -0.00319 | 65535 | 0.098471 | 0.139621 |
| P3-F8 | -0.34887 | 0.636095 | 0.99938 | -0.00738 | 65535 | 0.060889 | -0.06369 |
| P3-T3 | -0.57337 | 0.716256 | 0.99938 | -0.00762 | 65535 | 0.077159 | -0.10468 |
| P3-T4 | 0.585676 | 0.279606 | 0.99938 | -0.0033 | 65535 | 0.082111 | 0.106929 |
| P3-T5 | -1.82172 | 0.964485 | 0.99938 | -0.01566 | 65535 | 0.467943 | -0.3326 |
| P3-T6 | 0.433534 | 0.332709 | 0.99938 | -0.00439 | 65535 | 0.064889 | 0.079152 |
| P3-Fz | 0.804403 | 0.211391 | 0.99938 | -0.00246 | 65535 | 0.105284 | 0.146863 |
| P3-Cz | 0.426917 | 0.335109 | 0.99938 | -0.00377 | 65535 | 0.064712 | 0.077944 |
| P3-Pz | 0.044901 | 0.482131 | 0.99938 | -0.00689 | 65535 | 0.050172 | 0.008198 |
| P3-F9 | -0.64085 | 0.73857 | 0.99938 | -0.0081 | 65535 | 0.086252 | -0.117 |
| P3-F10 | 1.183099 | 0.119574 | 0.99938 | -0.00181 | 65535 | 0.166012 | 0.216003 |
| P4-O1 | -1.67961 | 0.952161 | 0.99938 | -0.01182 | 65535 | 0.406332 | -0.30665 |
| P4-O2 | -0.76673 | 0.777614 | 0.99938 | -0.01156 | 65535 | 0.118295 | -0.13999 |
| P4-F7 | -0.99053 | 0.838029 | 0.99938 | -0.00965 | 65535 | 0.154373 | -0.18084 |
| P4-F8 | -0.61006 | 0.728503 | 0.99938 | -0.00885 | 65535 | 0.093682 | -0.11138 |
| P4-T3 | -0.45319 | 0.674377 | 0.99938 | -0.00743 | 65535 | 0.069248 | -0.08274 |
| P4-T4 | 0.47929 | 0.31631 | 0.99938 | -0.00437 | 65535 | 0.068971 | 0.087506 |
| P4-T5 | -1.05285 | 0.85272 | 0.99938 | -0.00987 | 65535 | 0.159169 | -0.19222 |
| P4-T6 | -1.96441 | 0.974083 | 0.99938 | -0.01025 | 65535 | 0.484174 | -0.35865 |
| P4-Fz | 0.176688 | 0.430028 | 0.99938 | -0.00433 | 65535 | 0.053612 | 0.032259 |
| P4-Cz | 0.512662 | 0.304573 | 0.99938 | -0.00385 | 65535 | 0.080123 | 0.093599 |
| P4-Pz | 0.215859 | 0.414735 | 0.99938 | -0.00588 | 65535 | 0.053464 | 0.03941 |
| P4-F9 | -0.97139 | 0.833329 | 0.99938 | -0.00922 | 65535 | 0.143138 | -0.17735 |
| P4-F10 | -0.34633 | 0.635146 | 0.99938 | -0.00912 | 65535 | 0.058961 | -0.06323 |
| O1-O2 | -0.21433 | 0.584668 | 0.99938 | -0.00826 | 65535 | 0.054089 | -0.03913 |
| O1-F7 | -1.0168 | 0.844335 | 0.99938 | -0.008 | 65535 | 0.146205 | -0.18564 |
| O1-F8 | -0.6285 | 0.734554 | 0.99938 | -0.00734 | 65535 | 0.101125 | -0.11475 |
| O1-T3 | -1.88672 | 0.969172 | 0.99938 | -0.01168 | 65535 | 0.3803 | -0.34447 |
| O1-T4 | -1.16013 | 0.875831 | 0.99938 | -0.00973 | 65535 | 0.194751 | -0.21181 |
| O1-T5 | -2.78842 | 0.996912 | 0.99938 | -0.01401 | 65535 | 0.867143 | -0.50909 |
| O1-T6 | -0.3154 | 0.623493 | 0.99938 | -0.00614 | 65535 | 0.058677 | -0.05758 |
| O1-Fz | 1.3069 | 0.096894 | 0.99938 | -0.001 | 65535 | 0.219462 | 0.238606 |
| O1-Cz | -0.83321 | 0.796795 | 0.99938 | -0.00757 | 65535 | 0.125983 | -0.15212 |
| O1-Pz | 0.047059 | 0.481273 | 0.99938 | -0.00618 | 65535 | 0.050207 | 0.008592 |
| O1-F9 | -1.20853 | 0.88537 | 0.99938 | -0.00903 | 65535 | 0.178631 | -0.22065 |
| O1-F10 | -1.41615 | 0.920317 | 0.99938 | -0.01093 | 65535 | 0.226892 | -0.25855 |
| O2-F7 | -1.59331 | 0.943117 | 0.99938 | -0.01007 | 65535 | 0.289191 | -0.2909 |
| O2-F8 | -0.46526 | 0.6787 | 0.99938 | -0.01005 | 65535 | 0.073403 | -0.08495 |
| O2-T3 | -2.67969 | 0.995789 | 0.99938 | -0.01249 | 65535 | 0.693306 | -0.48924 |
| O2-T4 | 0.401701 | 0.344315 | 0.99938 | -0.00402 | 65535 | 0.065488 | 0.07334 |
| O2-T5 | -1.40038 | 0.917989 | 0.99938 | -0.00951 | 65535 | 0.291929 | -0.25567 |
| O2-T6 | -1.17441 | 0.878702 | 0.99938 | -0.00983 | 65535 | 0.178786 | -0.21442 |
| O2-Fz | 0.076836 | 0.469442 | 0.99938 | -0.00653 | 65535 | 0.050433 | 0.014028 |
| O2-Cz | -0.04247 | 0.516904 | 0.99938 | -0.00596 | 65535 | 0.05019 | -0.00775 |
| O2-Pz | 1.066389 | 0.144213 | 0.99938 | -0.00263 | 65535 | 0.148272 | 0.194695 |
| O2-F9 | -0.423 | 0.663467 | 0.99938 | -0.00604 | 65535 | 0.066634 | -0.07723 |
| O2-F10 | -1.13304 | 0.870252 | 0.99938 | -0.01092 | 65535 | 0.15776 | -0.20686 |
| F7-F8 | -0.62251 | 0.732596 | 0.99938 | -0.00753 | 65535 | 0.093338 | -0.11365 |
| F7-T3 | -0.52847 | 0.700918 | 0.99938 | -0.0067 | 65535 | 0.074853 | -0.09649 |
| F7-T4 | -0.47344 | 0.681615 | 0.99938 | -0.00659 | 65535 | 0.072285 | -0.08644 |
| F7-T5 | -1.50609 | 0.932642 | 0.99938 | -0.00943 | 65535 | 0.319065 | -0.27497 |
| F7-T6 | -0.87884 | 0.809364 | 0.99938 | -0.00733 | 65535 | 0.147933 | -0.16045 |
| F7-Fz | -1.35235 | 0.910576 | 0.99938 | -0.00921 | 65535 | 0.272744 | -0.2469 |
| F7-Cz | -0.71726 | 0.762685 | 0.99938 | -0.00715 | 65535 | 0.115305 | -0.13095 |
| F7-Pz | 0.836377 | 0.202317 | 0.99938 | -0.00369 | 65535 | 0.109861 | 0.152701 |
| F7-F9 | -0.79887 | 0.787014 | 0.99938 | -0.0078 | 65535 | 0.113927 | -0.14585 |
| F7-F10 | -1.04465 | 0.850839 | 0.99938 | -0.0075 | 65535 | 0.206696 | -0.19073 |
| F8-T3 | -1.16249 | 0.87631 | 0.99938 | -0.0088 | 65535 | 0.201128 | -0.21224 |
| F8-T4 | -0.51658 | 0.696791 | 0.99938 | -0.00629 | 65535 | 0.076114 | -0.09431 |
| F8-T5 | -0.4267 | 0.664811 | 0.99938 | -0.00739 | 65535 | 0.07653 | -0.0779 |
| F8-T6 | -1.54436 | 0.93741 | 0.99938 | -0.00917 | 65535 | 0.310209 | -0.28196 |
| F8-Fz | -0.92669 | 0.82201 | 0.99938 | -0.00872 | 65535 | 0.154051 | -0.16919 |
| F8-Cz | -0.27123 | 0.606657 | 0.99938 | -0.00615 | 65535 | 0.057603 | -0.04952 |
| F8-Pz | 0.22126 | 0.412636 | 0.99938 | -0.00501 | 65535 | 0.054225 | 0.040396 |
| F8-F9 | -1.57786 | 0.941363 | 0.99938 | -0.00943 | 65535 | 0.334477 | -0.28808 |
| F8-F10 | -3.30938 | 0.99938 | 0.99938 | -0.01342 | 65535 | 0.969143 | -0.60421 |
| T3-T4 | -2.44478 | 0.992014 | 0.99938 | -0.01187 | 65535 | 0.687375 | -0.44635 |
| T3-T5 | -1.61202 | 0.945185 | 0.99938 | -0.01054 | 65535 | 0.353489 | -0.29431 |
| T3-T6 | -1.7496 | 0.958606 | 0.99938 | -0.00966 | 65535 | 0.374722 | -0.31943 |
| T3-Fz | -1.16004 | 0.875814 | 0.99938 | -0.00732 | 65535 | 0.179179 | -0.21179 |
| T3-Cz | -1.80054 | 0.962835 | 0.99938 | -0.01077 | 65535 | 0.431224 | -0.32873 |
| T3-Pz | -0.61107 | 0.728837 | 0.99938 | -0.00791 | 65535 | 0.087742 | -0.11157 |
| T3-F9 | -2.05132 | 0.978775 | 0.99938 | -0.01152 | 65535 | 0.468429 | -0.37452 |
| T3-F10 | -1.20967 | 0.885589 | 0.99938 | -0.00876 | 65535 | 0.253807 | -0.22085 |
| T4-T5 | 0.41522 | 0.339367 | 0.99938 | -0.00353 | 65535 | 0.069762 | 0.075808 |
| T4-T6 | -1.24193 | 0.891638 | 0.99938 | -0.0085 | 65535 | 0.208185 | -0.22674 |
| T4-Fz | -1.27771 | 0.89807 | 0.99938 | -0.00874 | 65535 | 0.18868 | -0.23328 |
| T4-Cz | -0.18504 | 0.57324 | 0.99938 | -0.00522 | 65535 | 0.054229 | -0.03378 |
| T4-Pz | 1.805177 | 0.036798 | 0.99938 | 0.000608 | 65535 | 0.299501 | 0.329579 |
| T4-F9 | -0.91032 | 0.817745 | 0.99938 | -0.00797 | 65535 | 0.137109 | -0.1662 |
| T4-F10 | -0.64881 | 0.741138 | 0.99938 | -0.00777 | 65535 | 0.095318 | -0.11846 |
| T5-T6 | -1.16676 | 0.877169 | 0.99938 | -0.00929 | 65535 | 0.213835 | -0.21302 |
| T5-Fz | -0.71399 | 0.76168 | 0.99938 | -0.00716 | 65535 | 0.107851 | -0.13036 |
| T5-Cz | -1.42468 | 0.921556 | 0.99938 | -0.01032 | 65535 | 0.39442 | -0.26011 |
| T5-Pz | 0.324852 | 0.372934 | 0.99938 | -0.00571 | 65535 | 0.060228 | 0.05931 |
| T5-F9 | -1.24655 | 0.892484 | 0.99938 | -0.00971 | 65535 | 0.21306 | -0.22759 |
| T5-F10 | -1.68713 | 0.95289 | 0.99938 | -0.01162 | 65535 | 0.385958 | -0.30803 |
| T6-Fz | -0.12272 | 0.54873 | 0.99938 | -0.00468 | 65535 | 0.051479 | -0.0224 |
| T6-Cz | 0.416714 | 0.338822 | 0.99938 | -0.00369 | 65535 | 0.065104 | 0.076081 |
| T6-Pz | 0.256299 | 0.399083 | 0.99938 | -0.00469 | 65535 | 0.056407 | 0.046794 |
| T6-F9 | 0.201161 | 0.42046 | 0.99938 | -0.00471 | 65535 | 0.053411 | 0.036727 |
| T6-F10 | -1.74939 | 0.958588 | 0.99938 | -0.01079 | 65535 | 0.380329 | -0.31939 |
| Fz-Cz | 0.721985 | 0.235866 | 0.99938 | -0.00251 | 65535 | 0.126396 | 0.131816 |
| Fz-Pz | 2.036748 | 0.021957 | 0.99938 | 0.001413 | 65535 | 0.393352 | 0.371858 |
| Fz-F9 | -0.6605 | 0.74489 | 0.99938 | -0.00574 | 65535 | 0.089615 | -0.12059 |
| Fz-F10 | -1.61817 | 0.945852 | 0.99938 | -0.01009 | 65535 | 0.366104 | -0.29544 |
| Cz-Pz | 0.509703 | 0.305606 | 0.99938 | -0.00424 | 65535 | 0.074581 | 0.093059 |
| Cz-F9 | -1.41708 | 0.920452 | 0.99938 | -0.00869 | 65535 | 0.241894 | -0.25872 |
| Cz-F10 | -0.55478 | 0.709951 | 0.99938 | -0.00666 | 65535 | 0.091758 | -0.10129 |
| Pz-F9 | 0.052028 | 0.479297 | 0.99938 | -0.00587 | 65535 | 0.050294 | 0.009499 |
| Pz-F10 | -0.88619 | 0.81134 | 0.99938 | -0.00873 | 65535 | 0.122793 | -0.16179 |
| F9-F10 | -1.80616 | 0.963279 | 0.99938 | -0.01114 | 65535 | 0.454571 | -0.32976 |

Table S4f: delta_wpli_PT_HC

| channel | tvalue | pvalue | fdr_pvalue | CI_low | CI_high | statistical power | effect size |
| --- | --- | --- | --- | --- | --- | --- | --- |
| Fp1-Fp2 | -1.00866 | 0.1576 | 0.400164 | 65535 | 0.002024 | 0.171975 | -0.18416 |
| Fp1-F3 | 0.982042 | 0.835956 | 0.873387 | 65535 | 0.0075 | 0.139872 | 0.179296 |
| Fp1-F4 | -0.46177 | 0.322547 | 0.539372 | 65535 | 0.00391 | 0.072687 | -0.08431 |
| Fp1-C3 | -0.11587 | 0.453975 | 0.635566 | 65535 | 0.004852 | 0.051232 | -0.02116 |
| Fp1-C4 | -1.24799 | 0.107253 | 0.36221 | 65535 | 0.00128 | 0.209857 | -0.22785 |
| Fp1-P3 | -0.4516 | 0.326191 | 0.539372 | 65535 | 0.004407 | 0.067078 | -0.08245 |
| Fp1-P4 | -0.0143 | 0.494307 | 0.652858 | 65535 | 0.005654 | 0.050021 | -0.00261 |
| Fp1-O1 | -0.22507 | 0.411156 | 0.599603 | 65535 | 0.004557 | 0.056058 | -0.04109 |
| Fp1-O2 | -1.22381 | 0.111731 | 0.36221 | 65535 | 0.001391 | 0.229625 | -0.22344 |
| Fp1-F7 | -0.63974 | 0.261792 | 0.495777 | 65535 | 0.003285 | 0.091738 | -0.1168 |
| Fp1-F8 | -0.2312 | 0.408779 | 0.599603 | 65535 | 0.005032 | 0.055028 | -0.04221 |
| Fp1-T3 | -2.15442 | 0.01662 | 0.23268 | 65535 | -0.00152 | 0.631541 | -0.39334 |
| Fp1-T4 | -1.24434 | 0.10792 | 0.36221 | 65535 | 0.001244 | 0.196418 | -0.22718 |
| Fp1-T5 | -0.62594 | 0.266282 | 0.495777 | 65535 | 0.00316 | 0.09275 | -0.11428 |
| Fp1-T6 | 0.474267 | 0.681907 | 0.76778 | 65535 | 0.005961 | 0.075694 | 0.086589 |
| Fp1-Fz | -0.89078 | 0.187431 | 0.430469 | 65535 | 0.00255 | 0.135728 | -0.16263 |
| Fp1-Cz | -0.31234 | 0.377667 | 0.570576 | 65535 | 0.004672 | 0.05873 | -0.05702 |
| Fp1-Pz | 1.327663 | 0.906574 | 0.919713 | 65535 | 0.011162 | 0.216391 | 0.242397 |
| Fp1-F9 | -1.83439 | 0.034558 | 0.283079 | 65535 | -0.00057 | 0.410502 | -0.33491 |
| Fp1-F10 | -1.19149 | 0.117925 | 0.36221 | 65535 | 0.001676 | 0.187634 | -0.21754 |
| Fp2-F3 | -0.01501 | 0.494027 | 0.652858 | 65535 | 0.005063 | 0.050026 | -0.00274 |
| Fp2-F4 | -0.64782 | 0.259179 | 0.495777 | 65535 | 0.002881 | 0.090653 | -0.11828 |
| Fp2-C3 | -0.35207 | 0.362706 | 0.559265 | 65535 | 0.003716 | 0.066706 | -0.06428 |
| Fp2-C4 | -1.32466 | 0.093921 | 0.36221 | 65535 | 0.0011 | 0.230417 | -0.24185 |
| Fp2-P3 | 0.022423 | 0.508926 | 0.65872 | 65535 | 0.006698 | 0.050039 | 0.004094 |
| Fp2-P4 | -1.00805 | 0.157748 | 0.400164 | 65535 | 0.00224 | 0.161671 | -0.18404 |
| Fp2-O1 | -0.47714 | 0.317073 | 0.539372 | 65535 | 0.003864 | 0.072331 | -0.08711 |
| Fp2-O2 | -2.6535 | 0.004532 | 0.118961 | 65535 | -0.00285 | 0.75608 | -0.48446 |
| Fp2-F7 | -0.35263 | 0.362498 | 0.559265 | 65535 | 0.004572 | 0.062876 | -0.06438 |
| Fp2-F8 | -0.10308 | 0.459039 | 0.636021 | 65535 | 0.005305 | 0.051011 | -0.01882 |
| Fp2-T3 | -2.1739 | 0.015855 | 0.23268 | 65535 | -0.00146 | 0.556759 | -0.3969 |
| Fp2-T4 | -0.72591 | 0.234666 | 0.495517 | 65535 | 0.002882 | 0.112901 | -0.13253 |
| Fp2-T5 | -1.48598 | 0.069976 | 0.333774 | 65535 | 0.000514 | 0.297527 | -0.2713 |
| Fp2-T6 | 0.581326 | 0.718935 | 0.791829 | 65535 | 0.006455 | 0.081298 | 0.106135 |
| Fp2-Fz | -0.45439 | 0.325192 | 0.539372 | 65535 | 0.003346 | 0.067208 | -0.08296 |
| Fp2-Cz | -0.07702 | 0.46937 | 0.644233 | 65535 | 0.005234 | 0.050587 | -0.01406 |
| Fp2-Pz | 0.596349 | 0.723958 | 0.791829 | 65535 | 0.009612 | 0.074983 | 0.108878 |
| Fp2-F9 | -2.48725 | 0.007135 | 0.136221 | 65535 | -0.0024 | 0.694041 | -0.45411 |
| Fp2-F10 | -1.37055 | 0.086558 | 0.356414 | 65535 | 0.000913 | 0.229137 | -0.25023 |
| F3-F4 | 0.262078 | 0.603141 | 0.715591 | 65535 | 0.005835 | 0.055364 | 0.047849 |
| F3-C3 | -1.47437 | 0.071523 | 0.333774 | 65535 | 0.000521 | 0.311051 | -0.26918 |
| F3-C4 | -0.63074 | 0.264715 | 0.495777 | 65535 | 0.003387 | 0.080538 | -0.11516 |
| F3-P3 | 0.110354 | 0.543842 | 0.667876 | 65535 | 0.006751 | 0.05085 | 0.020148 |
| F3-P4 | -0.67021 | 0.252017 | 0.495777 | 65535 | 0.003268 | 0.088612 | -0.12236 |
| F3-O1 | -0.42257 | 0.336691 | 0.539733 | 65535 | 0.003483 | 0.06842 | -0.07715 |
| F3-O2 | -0.16394 | 0.435029 | 0.621471 | 65535 | 0.004268 | 0.05219 | -0.02993 |
| F3-F7 | -0.6286 | 0.265412 | 0.495777 | 65535 | 0.003132 | 0.087799 | -0.11477 |
| F3-F8 | 0.098447 | 0.539128 | 0.665981 | 65535 | 0.005746 | 0.05097 | 0.017974 |
| F3-T3 | -0.02622 | 0.489563 | 0.652858 | 65535 | 0.004964 | 0.050059 | -0.00479 |
| F3-T4 | 0.647287 | 0.740648 | 0.805887 | 65535 | 0.00772 | 0.087412 | 0.118178 |
| F3-T5 | -1.53008 | 0.064336 | 0.328959 | 65535 | 0.000368 | 0.273811 | -0.27935 |
| F3-T6 | -0.44081 | 0.330077 | 0.539733 | 65535 | 0.003515 | 0.080377 | -0.08048 |
| F3-Fz | -1.01341 | 0.156469 | 0.400164 | 65535 | 0.002028 | 0.142649 | -0.18502 |
| F3-Cz | 0.076218 | 0.530313 | 0.659273 | 65535 | 0.004965 | 0.050653 | 0.013915 |
| F3-Pz | 1.137745 | 0.871234 | 0.901277 | 65535 | 0.011042 | 0.152844 | 0.207723 |
| F3-F9 | -0.01775 | 0.492933 | 0.652858 | 65535 | 0.005517 | 0.050026 | -0.00324 |
| F3-F10 | -0.785 | 0.217012 | 0.46982 | 65535 | 0.00265 | 0.107131 | -0.14332 |
| F4-C3 | 0.754246 | 0.773898 | 0.828298 | 65535 | 0.007255 | 0.101664 | 0.137706 |
| F4-C4 | -3.02638 | 0.00152 | 0.097182 | 65535 | -0.00404 | 0.821465 | -0.55254 |
| F4-P3 | -1.60874 | 0.055173 | 0.31574 | 65535 | 0.000152 | 0.292106 | -0.29371 |
| F4-P4 | -2.01647 | 0.023011 | 0.283079 | 65535 | -0.00109 | 0.463547 | -0.36816 |
| F4-O1 | -1.0234 | 0.154107 | 0.400164 | 65535 | 0.002093 | 0.240901 | -0.18685 |
| F4-O2 | 0.036807 | 0.514649 | 0.65872 | 65535 | 0.007136 | 0.050116 | 0.00672 |
| F4-F7 | -0.26017 | 0.397592 | 0.587988 | 65535 | 0.004344 | 0.055869 | -0.0475 |
| F4-F8 | -1.09778 | 0.137267 | 0.389541 | 65535 | 0.001825 | 0.19251 | -0.20043 |
| F4-T3 | -1.81048 | 0.036383 | 0.283079 | 65535 | -0.00042 | 0.380047 | -0.33055 |
| F4-T4 | -0.41243 | 0.340388 | 0.541526 | 65535 | 0.003248 | 0.071931 | -0.0753 |
| F4-T5 | -0.26642 | 0.395192 | 0.587988 | 65535 | 0.004374 | 0.056753 | -0.04864 |
| F4-T6 | -2.92335 | 0.002076 | 0.097182 | 65535 | -0.00386 | 0.798157 | -0.53373 |
| F4-Fz | -1.79965 | 0.037236 | 0.283079 | 65535 | -0.00051 | 0.554297 | -0.32857 |
| F4-Cz | -2.58116 | 0.005536 | 0.128754 | 65535 | -0.00264 | 0.873119 | -0.47125 |
| F4-Pz | -1.22629 | 0.111266 | 0.36221 | 65535 | 0.001497 | 0.188347 | -0.22389 |
| F4-F9 | 0.353608 | 0.637868 | 0.748337 | 65535 | 0.005121 | 0.063587 | 0.06456 |
| F4-F10 | -1.86619 | 0.032249 | 0.283079 | 65535 | -0.00073 | 0.417827 | -0.34072 |
| C3-C4 | -0.93056 | 0.176992 | 0.424748 | 65535 | 0.002375 | 0.132801 | -0.1699 |
| C3-P3 | 1.242281 | 0.891702 | 0.913451 | 65535 | 0.010355 | 0.182743 | 0.226809 |
| C3-P4 | 0.075374 | 0.529978 | 0.659273 | 65535 | 0.006262 | 0.050534 | 0.013761 |
| C3-O1 | -1.24613 | 0.107592 | 0.36221 | 65535 | 0.001369 | 0.201771 | -0.22751 |
| C3-O2 | -0.89803 | 0.185498 | 0.430469 | 65535 | 0.002552 | 0.12272 | -0.16396 |
| C3-F7 | -1.32273 | 0.094242 | 0.36221 | 65535 | 0.001069 | 0.226885 | -0.2415 |
| C3-F8 | 1.479769 | 0.9292 | 0.938135 | 65535 | 0.010302 | 0.275025 | 0.270168 |
| C3-T3 | -0.60275 | 0.273917 | 0.495885 | 65535 | 0.003054 | 0.085515 | -0.11005 |
| C3-T4 | 0.013984 | 0.505567 | 0.65872 | 65535 | 0.004899 | 0.050016 | 0.002553 |
| C3-T5 | -2.29868 | 0.011642 | 0.188057 | 65535 | -0.00196 | 0.591703 | -0.41968 |
| C3-T6 | -0.09974 | 0.460358 | 0.636021 | 65535 | 0.004872 | 0.05112 | -0.01821 |
| C3-Fz | 0.021588 | 0.508594 | 0.65872 | 65535 | 0.006217 | 0.05004 | 0.003941 |
| C3-Cz | 0.662158 | 0.745419 | 0.806897 | 65535 | 0.0073 | 0.102003 | 0.120893 |
| C3-Pz | 0.962272 | 0.83106 | 0.872613 | 65535 | 0.012346 | 0.117335 | 0.175686 |
| C3-F9 | -0.70159 | 0.242158 | 0.495777 | 65535 | 0.002947 | 0.096961 | -0.12809 |
| C3-F10 | -1.79329 | 0.037744 | 0.283079 | 65535 | -0.00045 | 0.360995 | -0.32741 |
| C4-P3 | -1.51404 | 0.066345 | 0.328959 | 65535 | 0.000509 | 0.238741 | -0.27642 |
| C4-P4 | -1.20993 | 0.114361 | 0.36221 | 65535 | 0.001585 | 0.247787 | -0.2209 |
| C4-O1 | -1.59016 | 0.057237 | 0.31574 | 65535 | 0.000229 | 0.287465 | -0.29032 |
| C4-O2 | -1.43488 | 0.076983 | 0.341498 | 65535 | 0.000768 | 0.368728 | -0.26197 |
| C4-F7 | -0.99151 | 0.161733 | 0.400164 | 65535 | 0.002179 | 0.135861 | -0.18102 |
| C4-F8 | -1.04261 | 0.149631 | 0.400164 | 65535 | 0.002072 | 0.159126 | -0.19035 |
| C4-T3 | -3.06508 | 0.001349 | 0.097182 | 65535 | -0.0044 | 0.787462 | -0.55961 |
| C4-T4 | -0.04154 | 0.483466 | 0.652858 | 65535 | 0.005396 | 0.05016 | -0.00758 |
| C4-T5 | -2.88696 | 0.002314 | 0.097182 | 65535 | -0.00342 | 0.75211 | -0.52708 |
| C4-T6 | -0.99863 | 0.160009 | 0.400164 | 65535 | 0.00191 | 0.205557 | -0.18232 |
| C4-Fz | -0.81174 | 0.209287 | 0.462634 | 65535 | 0.002231 | 0.133855 | -0.1482 |
| C4-Cz | -1.83674 | 0.034382 | 0.283079 | 65535 | -0.00047 | 0.474484 | -0.33534 |
| C4-Pz | -1.28482 | 0.100686 | 0.36221 | 65535 | 0.001296 | 0.210157 | -0.23457 |
| C4-F9 | -0.3483 | 0.364117 | 0.559265 | 65535 | 0.004506 | 0.059152 | -0.06359 |
| C4-F10 | -2.54366 | 0.006131 | 0.128754 | 65535 | -0.00286 | 0.566794 | -0.46441 |
| P3-P4 | -0.14328 | 0.443157 | 0.628803 | 65535 | 0.00638 | 0.051731 | -0.02616 |
| P3-O1 | -1.61944 | 0.054011 | 0.31574 | 65535 | 0.000138 | 0.294857 | -0.29567 |
| P3-O2 | 0.41386 | 0.660136 | 0.757465 | 65535 | 0.010385 | 0.06421 | 0.07556 |
| P3-F7 | 0.764736 | 0.777022 | 0.828298 | 65535 | 0.008656 | 0.098471 | 0.139621 |
| P3-F8 | -0.34887 | 0.363905 | 0.559265 | 65535 | 0.004815 | 0.060889 | -0.06369 |
| P3-T3 | -0.57337 | 0.283744 | 0.509284 | 65535 | 0.003705 | 0.077159 | -0.10468 |
| P3-T4 | 0.585676 | 0.720394 | 0.791829 | 65535 | 0.006898 | 0.082111 | 0.106929 |
| P3-T5 | -1.82172 | 0.035515 | 0.283079 | 65535 | -0.00074 | 0.467943 | -0.3326 |
| P3-T6 | 0.433534 | 0.667291 | 0.757465 | 65535 | 0.007501 | 0.064889 | 0.079152 |
| P3-Fz | 0.804403 | 0.788609 | 0.836404 | 65535 | 0.007088 | 0.105284 | 0.146863 |
| P3-Cz | 0.426917 | 0.664891 | 0.757465 | 65535 | 0.006383 | 0.064712 | 0.077944 |
| P3-Pz | 0.044901 | 0.517869 | 0.65872 | 65535 | 0.007271 | 0.050172 | 0.008198 |
| P3-F9 | -0.64085 | 0.26143 | 0.495777 | 65535 | 0.003584 | 0.086252 | -0.117 |
| P3-F10 | 1.183099 | 0.880426 | 0.906321 | 65535 | 0.010823 | 0.166012 | 0.216003 |
| P4-O1 | -1.67961 | 0.047839 | 0.313946 | 65535 | -7.7E-05 | 0.406332 | -0.30665 |
| P4-O2 | -0.76673 | 0.222386 | 0.476541 | 65535 | 0.00425 | 0.118295 | -0.13999 |
| P4-F7 | -0.99053 | 0.161971 | 0.400164 | 65535 | 0.002431 | 0.154373 | -0.18084 |
| P4-F8 | -0.61006 | 0.271497 | 0.495777 | 65535 | 0.004089 | 0.093682 | -0.11138 |
| P4-T3 | -0.45319 | 0.325623 | 0.539372 | 65535 | 0.004242 | 0.069248 | -0.08274 |
| P4-T4 | 0.47929 | 0.68369 | 0.76778 | 65535 | 0.007928 | 0.068971 | 0.087506 |
| P4-T5 | -1.05285 | 0.14728 | 0.400164 | 65535 | 0.002204 | 0.159169 | -0.19222 |
| P4-T6 | -1.96441 | 0.025917 | 0.283079 | 65535 | -0.00087 | 0.484174 | -0.35865 |
| P4-Fz | 0.176688 | 0.569972 | 0.695896 | 65535 | 0.005361 | 0.053612 | 0.032259 |
| P4-Cz | 0.512662 | 0.695427 | 0.772697 | 65535 | 0.007302 | 0.080123 | 0.093599 |
| P4-Pz | 0.215859 | 0.585265 | 0.704836 | 65535 | 0.007641 | 0.053464 | 0.03941 |
| P4-F9 | -0.97139 | 0.166671 | 0.406988 | 65535 | 0.002407 | 0.143138 | -0.17735 |
| P4-F10 | -0.34633 | 0.364854 | 0.559265 | 65535 | 0.005966 | 0.058961 | -0.06323 |
| O1-O2 | -0.21433 | 0.415332 | 0.601515 | 65535 | 0.006366 | 0.054089 | -0.03913 |
| O1-F7 | -1.0168 | 0.155665 | 0.400164 | 65535 | 0.001917 | 0.146205 | -0.18564 |
| O1-F8 | -0.6285 | 0.265446 | 0.495777 | 65535 | 0.003302 | 0.101125 | -0.11475 |
| O1-T3 | -1.88672 | 0.030828 | 0.283079 | 65535 | -0.00075 | 0.3803 | -0.34447 |
| O1-T4 | -1.16013 | 0.124169 | 0.36221 | 65535 | 0.001718 | 0.194751 | -0.21181 |
| O1-T5 | -2.78842 | 0.003088 | 0.108083 | 65535 | -0.00356 | 0.867143 | -0.50909 |
| O1-T6 | -0.3154 | 0.376507 | 0.570576 | 65535 | 0.004178 | 0.058677 | -0.05758 |
| O1-Fz | 1.3069 | 0.903106 | 0.919713 | 65535 | 0.008446 | 0.219462 | 0.238606 |
| O1-Cz | -0.83321 | 0.203205 | 0.453968 | 65535 | 0.002507 | 0.125983 | -0.15212 |
| O1-Pz | 0.047059 | 0.518727 | 0.65872 | 65535 | 0.006543 | 0.050207 | 0.008592 |
| O1-F9 | -1.20853 | 0.11463 | 0.36221 | 65535 | 0.001416 | 0.178631 | -0.22065 |
| O1-F10 | -1.41615 | 0.079683 | 0.341498 | 65535 | 0.000859 | 0.226892 | -0.25855 |
| O2-F7 | -1.59331 | 0.056883 | 0.31574 | 65535 | 0.0002 | 0.289191 | -0.2909 |
| O2-F8 | -0.46526 | 0.3213 | 0.539372 | 65535 | 0.005646 | 0.073403 | -0.08495 |
| O2-T3 | -2.67969 | 0.004211 | 0.118961 | 65535 | -0.00294 | 0.693306 | -0.48924 |
| O2-T4 | 0.401701 | 0.655685 | 0.757465 | 65535 | 0.00659 | 0.065488 | 0.07334 |
| O2-T5 | -1.40038 | 0.082011 | 0.344447 | 65535 | 0.0008 | 0.291929 | -0.25567 |
| O2-T6 | -1.17441 | 0.121298 | 0.36221 | 65535 | 0.001678 | 0.178786 | -0.21442 |
| O2-Fz | 0.076836 | 0.530558 | 0.659273 | 65535 | 0.007169 | 0.050433 | 0.014028 |
| O2-Cz | -0.04247 | 0.483096 | 0.652858 | 65535 | 0.005663 | 0.05019 | -0.00775 |
| O2-Pz | 1.066389 | 0.855787 | 0.88968 | 65535 | 0.012103 | 0.148272 | 0.194695 |
| O2-F9 | -0.423 | 0.336533 | 0.539733 | 65535 | 0.003587 | 0.066634 | -0.07723 |
| O2-F10 | -1.13304 | 0.129748 | 0.373246 | 65535 | 0.002053 | 0.15776 | -0.20686 |
| F7-F8 | -0.62251 | 0.267404 | 0.495777 | 65535 | 0.003418 | 0.093338 | -0.11365 |
| F7-T3 | -0.52847 | 0.299082 | 0.527791 | 65535 | 0.00346 | 0.074853 | -0.09649 |
| F7-T4 | -0.47344 | 0.318385 | 0.539372 | 65535 | 0.003662 | 0.072285 | -0.08644 |
| F7-T5 | -1.50609 | 0.067358 | 0.328959 | 65535 | 0.000452 | 0.319065 | -0.27497 |
| F7-T6 | -0.87884 | 0.190636 | 0.430469 | 65535 | 0.002251 | 0.147933 | -0.16045 |
| F7-Fz | -1.35235 | 0.089424 | 0.361136 | 65535 | 0.000935 | 0.272744 | -0.2469 |
| F7-Cz | -0.71726 | 0.237315 | 0.495517 | 65535 | 0.002833 | 0.115305 | -0.13095 |
| F7-Pz | 0.836377 | 0.797683 | 0.841776 | 65535 | 0.011201 | 0.109861 | 0.152701 |
| F7-F9 | -0.79887 | 0.212986 | 0.465906 | 65535 | 0.002728 | 0.113927 | -0.14585 |
| F7-F10 | -1.04465 | 0.149161 | 0.400164 | 65535 | 0.001702 | 0.206696 | -0.19073 |
| F8-T3 | -1.16249 | 0.12369 | 0.36221 | 65535 | 0.001545 | 0.201128 | -0.21224 |
| F8-T4 | -0.51658 | 0.303209 | 0.530615 | 65535 | 0.003303 | 0.076114 | -0.09431 |
| F8-T5 | -0.4267 | 0.335189 | 0.539733 | 65535 | 0.004368 | 0.07653 | -0.0779 |
| F8-T6 | -1.54436 | 0.06259 | 0.328597 | 65535 | 0.000325 | 0.310209 | -0.28196 |
| F8-Fz | -0.92669 | 0.17799 | 0.424748 | 65535 | 0.002468 | 0.154051 | -0.16919 |
| F8-Cz | -0.27123 | 0.393343 | 0.587988 | 65535 | 0.004418 | 0.057603 | -0.04952 |
| F8-Pz | 0.22126 | 0.587364 | 0.704836 | 65535 | 0.006551 | 0.054225 | 0.040396 |
| F8-F9 | -1.57786 | 0.058637 | 0.31574 | 65535 | 0.000233 | 0.334477 | -0.28808 |
| F8-F10 | -3.30938 | 0.00062 | 0.097182 | 65535 | -0.00446 | 0.969143 | -0.60421 |
| T3-T4 | -2.44478 | 0.007986 | 0.139756 | 65535 | -0.00228 | 0.687375 | -0.44635 |
| T3-T5 | -1.61202 | 0.054815 | 0.31574 | 65535 | 0.000148 | 0.353489 | -0.29431 |
| T3-T6 | -1.7496 | 0.041394 | 0.289882 | 65535 | -0.00026 | 0.374722 | -0.31943 |
| T3-Fz | -1.16004 | 0.124186 | 0.36221 | 65535 | 0.001293 | 0.179179 | -0.21179 |
| T3-Cz | -1.80054 | 0.037165 | 0.283079 | 65535 | -0.00044 | 0.431224 | -0.32873 |
| T3-Pz | -0.61107 | 0.271163 | 0.495777 | 65535 | 0.00365 | 0.087742 | -0.11157 |
| T3-F9 | -2.05132 | 0.021225 | 0.278574 | 65535 | -0.00122 | 0.468429 | -0.37452 |
| T3-F10 | -1.20967 | 0.114411 | 0.36221 | 65535 | 0.00137 | 0.253807 | -0.22085 |
| T4-T5 | 0.41522 | 0.660633 | 0.757465 | 65535 | 0.005887 | 0.069762 | 0.075808 |
| T4-T6 | -1.24193 | 0.108362 | 0.36221 | 65535 | 0.00122 | 0.208185 | -0.22674 |
| T4-Fz | -1.27771 | 0.10193 | 0.36221 | 65535 | 0.001132 | 0.18868 | -0.23328 |
| T4-Cz | -0.18504 | 0.42676 | 0.613832 | 65535 | 0.004173 | 0.054229 | -0.03378 |
| T4-Pz | 1.805177 | 0.963202 | 0.96781 | 65535 | 0.014287 | 0.299501 | 0.329579 |
| T4-F9 | -0.91032 | 0.182255 | 0.430039 | 65535 | 0.00232 | 0.137109 | -0.1662 |
| T4-F10 | -0.64881 | 0.258862 | 0.495777 | 65535 | 0.003398 | 0.095318 | -0.11846 |
| T5-T6 | -1.16676 | 0.122831 | 0.36221 | 65535 | 0.001615 | 0.213835 | -0.21302 |
| T5-Fz | -0.71399 | 0.23832 | 0.495517 | 65535 | 0.002848 | 0.107851 | -0.13036 |
| T5-Cz | -1.42468 | 0.078444 | 0.341498 | 65535 | 0.000781 | 0.39442 | -0.26011 |
| T5-Pz | 0.324852 | 0.627066 | 0.739797 | 65535 | 0.008497 | 0.060228 | 0.05931 |
| T5-F9 | -1.24655 | 0.107516 | 0.36221 | 65535 | 0.001375 | 0.21306 | -0.22759 |
| T5-F10 | -1.68713 | 0.04711 | 0.313946 | 65535 | -0.0001 | 0.385958 | -0.30803 |
| T6-Fz | -0.12272 | 0.45127 | 0.635566 | 65535 | 0.004037 | 0.051479 | -0.0224 |
| T6-Cz | 0.416714 | 0.661178 | 0.757465 | 65535 | 0.006172 | 0.065104 | 0.076081 |
| T6-Pz | 0.256299 | 0.600917 | 0.715591 | 65535 | 0.006409 | 0.056407 | 0.046794 |
| T6-F9 | 0.201161 | 0.57954 | 0.703488 | 65535 | 0.006012 | 0.053411 | 0.036727 |
| T6-F10 | -1.74939 | 0.041412 | 0.289882 | 65535 | -0.00029 | 0.380329 | -0.31939 |
| Fz-Cz | 0.721985 | 0.764134 | 0.822913 | 65535 | 0.006377 | 0.126396 | 0.131816 |
| Fz-Pz | 2.036748 | 0.978043 | 0.978043 | 65535 | 0.013782 | 0.393352 | 0.371858 |
| Fz-F9 | -0.6605 | 0.25511 | 0.495777 | 65535 | 0.002468 | 0.089615 | -0.12059 |
| Fz-F10 | -1.61817 | 0.054148 | 0.31574 | 65535 | 0.000122 | 0.366104 | -0.29544 |
| Cz-Pz | 0.509703 | 0.694394 | 0.772697 | 65535 | 0.007999 | 0.074581 | 0.093059 |
| Cz-F9 | -1.41708 | 0.079548 | 0.341498 | 65535 | 0.00068 | 0.241894 | -0.25872 |
| Cz-F10 | -0.55478 | 0.290049 | 0.516189 | 65535 | 0.003319 | 0.091758 | -0.10129 |
| Pz-F9 | 0.052028 | 0.520703 | 0.65872 | 65535 | 0.006247 | 0.050294 | 0.009499 |
| Pz-F10 | -0.88619 | 0.18866 | 0.430469 | 65535 | 0.002647 | 0.122793 | -0.16179 |
| F9-F10 | -1.80616 | 0.036721 | 0.283079 | 65535 | -0.00048 | 0.454571 | -0.32976 |

Table S4g: theta_wpli_HC_PT

| channel | tvalue | pvalue | fdr_pvalue | CI_low | CI_high | statistical power | effect size |
| --- | --- | --- | --- | --- | --- | --- | --- |
| Fp1-Fp2 | -0.58417 | 0.719888 | 0.998966 | -0.00833 | 65535 | 0.131346 | -0.10665 |
| Fp1-F3 | -0.33265 | 0.630004 | 0.998966 | -0.00842 | 65535 | 0.070476 | -0.06073 |
| Fp1-F4 | 0.138099 | 0.445199 | 0.998966 | -0.00629 | 65535 | 0.053336 | 0.025213 |
| Fp1-C3 | -0.22734 | 0.589724 | 0.998966 | -0.00649 | 65535 | 0.059394 | -0.04151 |
| Fp1-C4 | -0.41885 | 0.661957 | 0.998966 | -0.008 | 65535 | 0.078998 | -0.07647 |
| Fp1-P3 | -2.08202 | 0.980249 | 0.998966 | -0.01992 | 65535 | 0.869271 | -0.38012 |
| Fp1-P4 | -1.27577 | 0.897729 | 0.998966 | -0.01518 | 65535 | 0.521314 | -0.23292 |
| Fp1-O1 | -2.965 | 0.998168 | 0.998966 | -0.02877 | 65535 | 0.999999 | -0.54133 |
| Fp1-O2 | -1.98931 | 0.97551 | 0.998966 | -0.01769 | 65535 | 0.741632 | -0.3632 |
| Fp1-F7 | 0.232782 | 0.408167 | 0.998966 | -0.00489 | 65535 | 0.056047 | 0.0425 |
| Fp1-F8 | 0.102586 | 0.459233 | 0.998966 | -0.00534 | 65535 | 0.05132 | 0.018729 |
| Fp1-T3 | -2.21194 | 0.985552 | 0.998966 | -0.01633 | 65535 | 0.899739 | -0.40384 |
| Fp1-T4 | -0.42657 | 0.664763 | 0.998966 | -0.00631 | 65535 | 0.075126 | -0.07788 |
| Fp1-T5 | -1.33035 | 0.907016 | 0.998966 | -0.01316 | 65535 | 0.331476 | -0.24289 |
| Fp1-T6 | -1.27427 | 0.897464 | 0.998966 | -0.01407 | 65535 | 0.400683 | -0.23265 |
| Fp1-Fz | 0.583176 | 0.280444 | 0.998966 | -0.00412 | 65535 | 0.093811 | 0.106473 |
| Fp1-Cz | -1.37502 | 0.914134 | 0.998966 | -0.01543 | 65535 | 0.69188 | -0.25104 |
| Fp1-Pz | -1.54868 | 0.937932 | 0.998966 | -0.01375 | 65535 | 0.637267 | -0.28275 |
| Fp1-F9 | -0.4139 | 0.660151 | 0.998966 | -0.0069 | 65535 | 0.078793 | -0.07557 |
| Fp1-F10 | -1.57646 | 0.941202 | 0.998966 | -0.01157 | 65535 | 0.506682 | -0.28782 |
| Fp2-F3 | 0.522425 | 0.301177 | 0.998966 | -0.00381 | 65535 | 0.085349 | 0.095381 |
| Fp2-F4 | 0.736444 | 0.231461 | 0.998966 | -0.00396 | 65535 | 0.146825 | 0.134456 |
| Fp2-C3 | -1.33675 | 0.908063 | 0.998966 | -0.011 | 65535 | 0.407073 | -0.24406 |
| Fp2-C4 | -0.83805 | 0.798151 | 0.998966 | -0.00977 | 65535 | 0.18694 | -0.15301 |
| Fp2-P3 | -1.8588 | 0.967227 | 0.998966 | -0.0186 | 65535 | 0.71384 | -0.33937 |
| Fp2-P4 | -1.27532 | 0.897649 | 0.998966 | -0.01421 | 65535 | 0.498309 | -0.23284 |
| Fp2-O1 | -2.55109 | 0.993991 | 0.998966 | -0.02536 | 65535 | 0.998005 | -0.46576 |
| Fp2-O2 | -1.95282 | 0.973395 | 0.998966 | -0.01512 | 65535 | 0.837815 | -0.35653 |
| Fp2-F7 | -0.11436 | 0.545428 | 0.998966 | -0.00617 | 65535 | 0.051929 | -0.02088 |
| Fp2-F8 | 1.147355 | 0.126778 | 0.998966 | -0.00171 | 65535 | 0.226893 | 0.209477 |
| Fp2-T3 | -1.70831 | 0.954896 | 0.998966 | -0.01473 | 65535 | 0.654704 | -0.31189 |
| Fp2-T4 | -0.64955 | 0.741378 | 0.998966 | -0.00875 | 65535 | 0.148042 | -0.11859 |
| Fp2-T5 | -0.78718 | 0.783623 | 0.998966 | -0.01072 | 65535 | 0.146857 | -0.14372 |
| Fp2-T6 | -1.04308 | 0.850477 | 0.998966 | -0.01364 | 65535 | 0.277801 | -0.19044 |
| Fp2-Fz | -0.18522 | 0.573312 | 0.998966 | -0.00673 | 65535 | 0.054594 | -0.03382 |
| Fp2-Cz | -0.77504 | 0.780066 | 0.998966 | -0.01147 | 65535 | 0.209205 | -0.1415 |
| Fp2-Pz | -1.05644 | 0.853538 | 0.998966 | -0.01181 | 65535 | 0.311451 | -0.19288 |
| Fp2-F9 | -0.31392 | 0.622933 | 0.998966 | -0.00561 | 65535 | 0.06462 | -0.05731 |
| Fp2-F10 | -0.69493 | 0.755767 | 0.998966 | -0.00804 | 65535 | 0.13466 | -0.12688 |
| F3-F4 | -0.5814 | 0.718962 | 0.998966 | -0.00715 | 65535 | 0.085122 | -0.10615 |
| F3-C3 | 0.079341 | 0.468448 | 0.998966 | -0.00845 | 65535 | 0.051482 | 0.014486 |
| F3-C4 | 0.109546 | 0.456478 | 0.998966 | -0.00523 | 65535 | 0.051548 | 0.02 |
| F3-P3 | -1.38254 | 0.915292 | 0.998966 | -0.01293 | 65535 | 0.485767 | -0.25242 |
| F3-P4 | -0.13308 | 0.552822 | 0.998966 | -0.00744 | 65535 | 0.053772 | -0.0243 |
| F3-O1 | -0.4817 | 0.684545 | 0.998966 | -0.0088 | 65535 | 0.12499 | -0.08795 |
| F3-O2 | -1.15475 | 0.874737 | 0.998966 | -0.0086 | 65535 | 0.274381 | -0.21083 |
| F3-F7 | 0.24458 | 0.403603 | 0.998966 | -0.00624 | 65535 | 0.066294 | 0.044654 |
| F3-F8 | 1.272045 | 0.102929 | 0.998966 | -0.00129 | 65535 | 0.247893 | 0.232243 |
| F3-T3 | 0.181489 | 0.428148 | 0.998966 | -0.00712 | 65535 | 0.061189 | 0.033135 |
| F3-T4 | 1.557499 | 0.061015 | 0.998966 | -0.00033 | 65535 | 0.455574 | 0.284359 |
| F3-T5 | -0.97054 | 0.833118 | 0.998966 | -0.01304 | 65535 | 0.326571 | -0.17719 |
| F3-T6 | 0.688307 | 0.246305 | 0.998966 | -0.00464 | 65535 | 0.248018 | 0.125667 |
| F3-Fz | -0.32448 | 0.626926 | 0.998966 | -0.0077 | 65535 | 0.066755 | -0.05924 |
| F3-Cz | -0.53485 | 0.70312 | 0.998966 | -0.01037 | 65535 | 0.101092 | -0.09765 |
| F3-Pz | -0.72685 | 0.76562 | 0.998966 | -0.01076 | 65535 | 0.237517 | -0.1327 |
| F3-F9 | 1.398085 | 0.082355 | 0.998966 | -0.00093 | 65535 | 0.305927 | 0.255254 |
| F3-F10 | 0.233703 | 0.40781 | 0.998966 | -0.00683 | 65535 | 0.069752 | 0.042668 |
| F4-C3 | -0.59662 | 0.724048 | 0.998966 | -0.00776 | 65535 | 0.103015 | -0.10893 |
| F4-C4 | -0.57231 | 0.715899 | 0.998966 | -0.00913 | 65535 | 0.101505 | -0.10449 |
| F4-P3 | -0.73656 | 0.768574 | 0.998966 | -0.00754 | 65535 | 0.109251 | -0.13448 |
| F4-P4 | -0.66349 | 0.745844 | 0.998966 | -0.00986 | 65535 | 0.139228 | -0.12114 |
| F4-O1 | 0.026876 | 0.489302 | 0.998966 | -0.00616 | 65535 | 0.050203 | 0.004907 |
| F4-O2 | -0.72777 | 0.765902 | 0.998966 | -0.00781 | 65535 | 0.133613 | -0.13287 |
| F4-F7 | -0.74899 | 0.772324 | 0.998966 | -0.00915 | 65535 | 0.13181 | -0.13675 |
| F4-F8 | -0.15686 | 0.56219 | 0.998966 | -0.00899 | 65535 | 0.056853 | -0.02864 |
| F4-T3 | -1.38736 | 0.916026 | 0.998966 | -0.01242 | 65535 | 0.560702 | -0.2533 |
| F4-T4 | 0.020339 | 0.491904 | 0.998966 | -0.00936 | 65535 | 0.050154 | 0.003713 |
| F4-T5 | 0.246503 | 0.402861 | 0.998966 | -0.00545 | 65535 | 0.066825 | 0.045005 |
| F4-T6 | -1.08678 | 0.860326 | 0.998966 | -0.01093 | 65535 | 0.320939 | -0.19842 |
| F4-Fz | 0.205238 | 0.41887 | 0.998966 | -0.00463 | 65535 | 0.055281 | 0.037471 |
| F4-Cz | 0.324654 | 0.373009 | 0.998966 | -0.00476 | 65535 | 0.061941 | 0.059273 |
| F4-Pz | -0.36698 | 0.642856 | 0.998966 | -0.00819 | 65535 | 0.083638 | -0.067 |
| F4-F9 | 0.294342 | 0.384507 | 0.998966 | -0.0062 | 65535 | 0.076342 | 0.053739 |
| F4-F10 | -1.39446 | 0.917101 | 0.998966 | -0.01106 | 65535 | 0.367293 | -0.25459 |
| C3-C4 | 0.358599 | 0.360268 | 0.998966 | -0.00484 | 65535 | 0.070091 | 0.065471 |
| C3-P3 | -1.8563 | 0.967047 | 0.998966 | -0.01288 | 65535 | 0.608561 | -0.33891 |
| C3-P4 | -0.92933 | 0.822692 | 0.998966 | -0.01086 | 65535 | 0.205076 | -0.16967 |
| C3-O1 | -1.52286 | 0.934765 | 0.998966 | -0.022 | 65535 | 0.915283 | -0.27803 |
| C3-O2 | -2.04976 | 0.978698 | 0.998966 | -0.01393 | 65535 | 0.827258 | -0.37423 |
| C3-F7 | -1.40855 | 0.919201 | 0.998966 | -0.01302 | 65535 | 0.560248 | -0.25716 |
| C3-F8 | 0.504259 | 0.30751 | 0.998966 | -0.00385 | 65535 | 0.088784 | 0.092065 |
| C3-T3 | -2.0549 | 0.978952 | 0.998966 | -0.02275 | 65535 | 0.992907 | -0.37517 |
| C3-T4 | 0.365305 | 0.357769 | 0.998966 | -0.00394 | 65535 | 0.076757 | 0.066695 |
| C3-T5 | -0.8377 | 0.798055 | 0.998966 | -0.00991 | 65535 | 0.151839 | -0.15294 |
| C3-T6 | -1.40066 | 0.91803 | 0.998966 | -0.01286 | 65535 | 0.542958 | -0.25572 |
| C3-Fz | 0.170364 | 0.432508 | 0.998966 | -0.0052 | 65535 | 0.053813 | 0.031104 |
| C3-Cz | 0.100218 | 0.460171 | 0.998966 | -0.00486 | 65535 | 0.051203 | 0.018297 |
| C3-Pz | -1.96023 | 0.973837 | 0.998966 | -0.0156 | 65535 | 0.778242 | -0.35789 |
| C3-F9 | -0.16673 | 0.566066 | 0.998966 | -0.00655 | 65535 | 0.054475 | -0.03044 |
| C3-F10 | -1.5103 | 0.93318 | 0.998966 | -0.01519 | 65535 | 0.751128 | -0.27574 |
| C4-P3 | -1.90862 | 0.97063 | 0.998966 | -0.01357 | 65535 | 0.563383 | -0.34847 |
| C4-P4 | -0.75086 | 0.772885 | 0.998966 | -0.01062 | 65535 | 0.182586 | -0.13709 |
| C4-O1 | -0.32055 | 0.625442 | 0.998966 | -0.00986 | 65535 | 0.090051 | -0.05852 |
| C4-O2 | 0.237913 | 0.406181 | 0.998966 | -0.0053 | 65535 | 0.060442 | 0.043437 |
| C4-F7 | 0.38058 | 0.3521 | 0.998966 | -0.0046 | 65535 | 0.080415 | 0.069484 |
| C4-F8 | 1.042157 | 0.149735 | 0.998966 | -0.0026 | 65535 | 0.277935 | 0.190271 |
| C4-T3 | -2.21027 | 0.985492 | 0.998966 | -0.01546 | 65535 | 0.910254 | -0.40354 |
| C4-T4 | -0.68907 | 0.753935 | 0.998966 | -0.01043 | 65535 | 0.216245 | -0.12581 |
| C4-T5 | -0.5715 | 0.715627 | 0.998966 | -0.00737 | 65535 | 0.114685 | -0.10434 |
| C4-T6 | -0.14519 | 0.557596 | 0.998966 | -0.00788 | 65535 | 0.052897 | -0.02651 |
| C4-Fz | -0.50979 | 0.694424 | 0.998966 | -0.00738 | 65535 | 0.096704 | -0.09307 |
| C4-Cz | -0.83591 | 0.797552 | 0.998966 | -0.00909 | 65535 | 0.185389 | -0.15262 |
| C4-Pz | -1.02245 | 0.84567 | 0.998966 | -0.01175 | 65535 | 0.334865 | -0.18667 |
| C4-F9 | -0.3793 | 0.647427 | 0.998966 | -0.00677 | 65535 | 0.073556 | -0.06925 |
| C4-F10 | -0.93346 | 0.823756 | 0.998966 | -0.00961 | 65535 | 0.277442 | -0.17043 |
| P3-P4 | -2.0597 | 0.979187 | 0.998966 | -0.0142 | 65535 | 0.729858 | -0.37605 |
| P3-O1 | -1.70702 | 0.954776 | 0.998966 | -0.01961 | 65535 | 0.550513 | -0.31166 |
| P3-O2 | -1.55731 | 0.938962 | 0.998966 | -0.01559 | 65535 | 0.336484 | -0.28432 |
| P3-F7 | -1.93751 | 0.972464 | 0.998966 | -0.02004 | 65535 | 0.986587 | -0.35374 |
| P3-F8 | -0.9174 | 0.819597 | 0.998966 | -0.0122 | 65535 | 0.158084 | -0.16749 |
| P3-T3 | -2.63133 | 0.99518 | 0.998966 | -0.02532 | 65535 | 0.978506 | -0.48041 |
| P3-T4 | -0.19551 | 0.577335 | 0.998966 | -0.00759 | 65535 | 0.055313 | -0.03569 |
| P3-T5 | -1.80903 | 0.963504 | 0.998966 | -0.01338 | 65535 | 0.539683 | -0.33028 |
| P3-T6 | -2.4407 | 0.991928 | 0.998966 | -0.0147 | 65535 | 0.916481 | -0.44561 |
| P3-Fz | -2.5597 | 0.99413 | 0.998966 | -0.01362 | 65535 | 0.784331 | -0.46733 |
| P3-Cz | -2.48307 | 0.992785 | 0.998966 | -0.01558 | 65535 | 0.97162 | -0.45335 |
| P3-Pz | -1.45738 | 0.926166 | 0.998966 | -0.01329 | 65535 | 0.443934 | -0.26608 |
| P3-F9 | -0.5922 | 0.722575 | 0.998966 | -0.01036 | 65535 | 0.084779 | -0.10812 |
| P3-F10 | -1.84979 | 0.966577 | 0.998966 | -0.01617 | 65535 | 0.888677 | -0.33772 |
| P4-O1 | -1.87152 | 0.968125 | 0.998966 | -0.01545 | 65535 | 0.863918 | -0.34169 |
| P4-O2 | -1.18126 | 0.880064 | 0.998966 | -0.01175 | 65535 | 0.262846 | -0.21567 |
| P4-F7 | -1.6842 | 0.952607 | 0.998966 | -0.01504 | 65535 | 0.666989 | -0.30749 |
| P4-F8 | -1.07409 | 0.857513 | 0.998966 | -0.01335 | 65535 | 0.316455 | -0.1961 |
| P4-T3 | -1.3735 | 0.9139 | 0.998966 | -0.01531 | 65535 | 0.556424 | -0.25077 |
| P4-T4 | -1.21615 | 0.886822 | 0.998966 | -0.01497 | 65535 | 0.431549 | -0.22204 |
| P4-T5 | -0.96811 | 0.832516 | 0.998966 | -0.00871 | 65535 | 0.211314 | -0.17675 |
| P4-T6 | -1.23591 | 0.890527 | 0.998966 | -0.01596 | 65535 | 0.344039 | -0.22565 |
| P4-Fz | -1.2633 | 0.895515 | 0.998966 | -0.01119 | 65535 | 0.576151 | -0.23065 |
| P4-Cz | -1.32129 | 0.90552 | 0.998966 | -0.01102 | 65535 | 0.4249 | -0.24123 |
| P4-Pz | -1.79592 | 0.962467 | 0.998966 | -0.01503 | 65535 | 0.787666 | -0.32789 |
| P4-F9 | 0.215042 | 0.415053 | 0.998966 | -0.00563 | 65535 | 0.057256 | 0.039261 |
| P4-F10 | -1.43745 | 0.923381 | 0.998966 | -0.01496 | 65535 | 0.652217 | -0.26244 |
| O1-O2 | -1.19109 | 0.881996 | 0.998966 | -0.013 | 65535 | 0.463515 | -0.21746 |
| O1-F7 | -2.6809 | 0.995803 | 0.998966 | -0.02761 | 65535 | 1 | -0.48946 |
| O1-F8 | -1.34392 | 0.909224 | 0.998966 | -0.01709 | 65535 | 0.679888 | -0.24537 |
| O1-T3 | -2.58184 | 0.994475 | 0.998966 | -0.02515 | 65535 | 0.999809 | -0.47138 |
| O1-T4 | -2.48094 | 0.992743 | 0.998966 | -0.02349 | 65535 | 0.999451 | -0.45295 |
| O1-T5 | -2.13399 | 0.982543 | 0.998966 | -0.02104 | 65535 | 0.749457 | -0.38961 |
| O1-T6 | -1.56192 | 0.939507 | 0.998966 | -0.01306 | 65535 | 0.565887 | -0.28517 |
| O1-Fz | -1.28001 | 0.898474 | 0.998966 | -0.01173 | 65535 | 0.526853 | -0.2337 |
| O1-Cz | -1.63564 | 0.94771 | 0.998966 | -0.02058 | 65535 | 0.889014 | -0.29863 |
| O1-Pz | -1.0074 | 0.842098 | 0.998966 | -0.01435 | 65535 | 0.410254 | -0.18393 |
| O1-F9 | -2.16533 | 0.983812 | 0.998966 | -0.02113 | 65535 | 0.98239 | -0.39533 |
| O1-F10 | -1.36157 | 0.912036 | 0.998966 | -0.01662 | 65535 | 0.788206 | -0.24859 |
| O2-F7 | -3.14915 | 0.998963 | 0.998966 | -0.01785 | 65535 | 0.995486 | -0.57495 |
| O2-F8 | -1.02784 | 0.846936 | 0.998966 | -0.01229 | 65535 | 0.182346 | -0.18766 |
| O2-T3 | -3.13582 | 0.998918 | 0.998966 | -0.02076 | 65535 | 0.999189 | -0.57252 |
| O2-T4 | -1.83277 | 0.965321 | 0.998966 | -0.01431 | 65535 | 0.750002 | -0.33462 |
| O2-T5 | -0.82642 | 0.794884 | 0.998966 | -0.00998 | 65535 | 0.13311 | -0.15088 |
| O2-T6 | -2.16287 | 0.983716 | 0.998966 | -0.01694 | 65535 | 0.855024 | -0.39488 |
| O2-Fz | -0.5466 | 0.707158 | 0.998966 | -0.00757 | 65535 | 0.114831 | -0.0998 |
| O2-Cz | -2.37856 | 0.990507 | 0.998966 | -0.0188 | 65535 | 0.997331 | -0.43426 |
| O2-Pz | -1.70263 | 0.954365 | 0.998966 | -0.01358 | 65535 | 0.614752 | -0.31086 |
| O2-F9 | -1.54308 | 0.937256 | 0.998966 | -0.01105 | 65535 | 0.484385 | -0.28173 |
| O2-F10 | -2.47675 | 0.992662 | 0.998966 | -0.01825 | 65535 | 0.98452 | -0.45219 |
| F7-F8 | 0.826536 | 0.205084 | 0.998966 | -0.00267 | 65535 | 0.154499 | 0.150904 |
| F7-T3 | -1.59221 | 0.942994 | 0.998966 | -0.01858 | 65535 | 0.84512 | -0.2907 |
| F7-T4 | -0.09657 | 0.538383 | 0.998966 | -0.00483 | 65535 | 0.051298 | -0.01763 |
| F7-T5 | -0.85303 | 0.802315 | 0.998966 | -0.01261 | 65535 | 0.248963 | -0.15574 |
| F7-T6 | -1.53201 | 0.935901 | 0.998966 | -0.01585 | 65535 | 0.796048 | -0.2797 |
| F7-Fz | 0.005743 | 0.497714 | 0.998966 | -0.00613 | 65535 | 0.050006 | 0.001049 |
| F7-Cz | -1.11737 | 0.866948 | 0.998966 | -0.01562 | 65535 | 0.353956 | -0.204 |
| F7-Pz | -1.04235 | 0.850309 | 0.998966 | -0.01485 | 65535 | 0.385582 | -0.19031 |
| F7-F9 | -0.25934 | 0.602087 | 0.998966 | -0.00639 | 65535 | 0.061625 | -0.04735 |
| F7-F10 | -1.84031 | 0.965882 | 0.998966 | -0.01802 | 65535 | 0.951937 | -0.33599 |
| F8-T3 | -2.52293 | 0.993515 | 0.998966 | -0.01529 | 65535 | 0.966742 | -0.46062 |
| F8-T4 | -0.16561 | 0.565626 | 0.998966 | -0.01004 | 65535 | 0.060476 | -0.03024 |
| F8-T5 | 0.44455 | 0.328729 | 0.998966 | -0.00606 | 65535 | 0.074809 | 0.081163 |
| F8-T6 | -0.61122 | 0.728886 | 0.998966 | -0.01161 | 65535 | 0.110517 | -0.11159 |
| F8-Fz | 0.748136 | 0.227933 | 0.998966 | -0.00291 | 65535 | 0.140352 | 0.13659 |
| F8-Cz | -0.11249 | 0.544686 | 0.998966 | -0.00704 | 65535 | 0.051681 | -0.02054 |
| F8-Pz | -0.49449 | 0.689062 | 0.998966 | -0.0086 | 65535 | 0.100493 | -0.09028 |
| F8-F9 | 0.624025 | 0.266908 | 0.998966 | -0.00345 | 65535 | 0.114025 | 0.113931 |
| F8-F10 | -0.05017 | 0.519963 | 0.998966 | -0.00576 | 65535 | 0.050443 | -0.00916 |
| T3-T4 | -2.95036 | 0.998086 | 0.998966 | -0.0144 | 65535 | 0.91533 | -0.53866 |
| T3-T5 | -1.86031 | 0.967334 | 0.998966 | -0.01965 | 65535 | 0.689813 | -0.33964 |
| T3-T6 | -1.62322 | 0.946395 | 0.998966 | -0.01296 | 65535 | 0.651696 | -0.29636 |
| T3-Fz | -0.99721 | 0.83965 | 0.998966 | -0.00921 | 65535 | 0.23443 | -0.18207 |
| T3-Cz | -2.00649 | 0.976455 | 0.998966 | -0.01747 | 65535 | 0.933762 | -0.36633 |
| T3-Pz | -1.60649 | 0.944581 | 0.998966 | -0.0159 | 65535 | 0.701717 | -0.2933 |
| T3-F9 | -1.97066 | 0.974448 | 0.998966 | -0.01475 | 65535 | 0.850482 | -0.35979 |
| T3-F10 | -2.47564 | 0.99264 | 0.998966 | -0.01252 | 65535 | 0.907088 | -0.45199 |
| T4-T5 | -1.91919 | 0.971312 | 0.998966 | -0.01516 | 65535 | 0.666629 | -0.35039 |
| T4-T6 | -1.33177 | 0.907248 | 0.998966 | -0.0153 | 65535 | 0.378019 | -0.24315 |
| T4-Fz | 0.11182 | 0.455578 | 0.998966 | -0.00519 | 65535 | 0.05218 | 0.020416 |
| T4-Cz | -1.42338 | 0.921368 | 0.998966 | -0.01175 | 65535 | 0.441237 | -0.25987 |
| T4-Pz | -0.36128 | 0.640732 | 0.998966 | -0.00857 | 65535 | 0.080868 | -0.06596 |
| T4-F9 | -2.06583 | 0.979483 | 0.998966 | -0.01472 | 65535 | 0.848567 | -0.37717 |
| T4-F10 | -0.67212 | 0.74859 | 0.998966 | -0.00755 | 65535 | 0.156377 | -0.12271 |
| T5-T6 | -2.1888 | 0.98471 | 0.998966 | -0.01419 | 65535 | 0.915612 | -0.39962 |
| T5-Fz | 0.603441 | 0.273687 | 0.998966 | -0.00364 | 65535 | 0.09919 | 0.110173 |
| T5-Cz | -1.04253 | 0.850352 | 0.998966 | -0.00961 | 65535 | 0.257058 | -0.19034 |
| T5-Pz | -1.41585 | 0.920273 | 0.998966 | -0.01178 | 65535 | 0.415408 | -0.2585 |
| T5-F9 | -1.04128 | 0.850062 | 0.998966 | -0.01145 | 65535 | 0.196368 | -0.19011 |
| T5-F10 | -1.97256 | 0.974558 | 0.998966 | -0.01578 | 65535 | 0.829292 | -0.36014 |
| T6-Fz | -0.44676 | 0.672067 | 0.998966 | -0.0071 | 65535 | 0.08726 | -0.08157 |
| T6-Cz | -1.68749 | 0.952925 | 0.998966 | -0.01409 | 65535 | 0.66205 | -0.30809 |
| T6-Pz | -1.73271 | 0.95712 | 0.998966 | -0.01433 | 65535 | 0.687467 | -0.31635 |
| T6-F9 | -1.49891 | 0.931717 | 0.998966 | -0.01424 | 65535 | 0.574566 | -0.27366 |
| T6-F10 | -1.61329 | 0.945323 | 0.998966 | -0.01344 | 65535 | 0.666289 | -0.29454 |
| Fz-Cz | 0.366815 | 0.357207 | 0.998966 | -0.0046 | 65535 | 0.067288 | 0.066971 |
| Fz-Pz | -2.37181 | 0.99034 | 0.998966 | -0.01434 | 65535 | 0.937879 | -0.43303 |
| Fz-F9 | 1.307093 | 0.096861 | 0.998966 | -0.00107 | 65535 | 0.335167 | 0.238641 |
| Fz-F10 | -0.14514 | 0.557576 | 0.998966 | -0.00604 | 65535 | 0.054054 | -0.0265 |
| Cz-Pz | -3.15006 | 0.998966 | 0.998966 | -0.02184 | 65535 | 0.999985 | -0.57512 |
| Cz-F9 | -1.03209 | 0.84793 | 0.998966 | -0.00956 | 65535 | 0.217231 | -0.18843 |
| Cz-F10 | -2.36139 | 0.990077 | 0.998966 | -0.01567 | 65535 | 0.866394 | -0.43113 |
| Pz-F9 | -0.02132 | 0.508486 | 0.998966 | -0.00712 | 65535 | 0.050098 | -0.00389 |
| Pz-F10 | -1.04975 | 0.852012 | 0.998966 | -0.01087 | 65535 | 0.273161 | -0.19166 |
| F9-F10 | -1.04497 | 0.850913 | 0.998966 | -0.00891 | 65535 | 0.278138 | -0.19078 |

Table S4h: theta_wpli_PT_HC

| channel | tvalue | pvalue | fdr_pvalue | CI_low | CI_high | statistical power | effect size |
| --- | --- | --- | --- | --- | --- | --- | --- |
| Fp1-Fp2 | -0.58417 | 0.280112 | 0.429629 | 65535 | 0.003991 | 0.131346 | -0.10665 |
| Fp1-F3 | -0.33265 | 0.369996 | 0.510761 | 65535 | 0.005605 | 0.070476 | -0.06073 |
| Fp1-F4 | 0.138099 | 0.554801 | 0.650884 | 65535 | 0.007428 | 0.053336 | 0.025213 |
| Fp1-C3 | -0.22734 | 0.410276 | 0.548777 | 65535 | 0.004922 | 0.059394 | -0.04151 |
| Fp1-C4 | -0.41885 | 0.338043 | 0.482219 | 65535 | 0.004773 | 0.078998 | -0.07647 |
| Fp1-P3 | -2.08202 | 0.019751 | 0.149114 | 65535 | -0.00226 | 0.869271 | -0.38012 |
| Fp1-P4 | -1.27577 | 0.102271 | 0.241939 | 65535 | 0.001977 | 0.521314 | -0.23292 |
| Fp1-O1 | -2.965 | 0.001832 | 0.080407 | 65535 | -0.00814 | 0.999999 | -0.54133 |
| Fp1-O2 | -1.98931 | 0.02449 | 0.155194 | 65535 | -0.00161 | 0.741632 | -0.3632 |
| Fp1-F7 | 0.232782 | 0.591833 | 0.667017 | 65535 | 0.006492 | 0.056047 | 0.0425 |
| Fp1-F8 | 0.102586 | 0.540767 | 0.642296 | 65535 | 0.006042 | 0.05132 | 0.018729 |
| Fp1-T3 | -2.21194 | 0.014448 | 0.142486 | 65535 | -0.00234 | 0.899739 | -0.40384 |
| Fp1-T4 | -0.42657 | 0.335237 | 0.48219 | 65535 | 0.003727 | 0.075126 | -0.07788 |
| Fp1-T5 | -1.33035 | 0.092984 | 0.232461 | 65535 | 0.001443 | 0.331476 | -0.24289 |
| Fp1-T6 | -1.27427 | 0.102536 | 0.241939 | 65535 | 0.001841 | 0.400683 | -0.23265 |
| Fp1-Fz | 0.583176 | 0.719556 | 0.763166 | 65535 | 0.008582 | 0.093811 | 0.106473 |
| Fp1-Cz | -1.37502 | 0.085866 | 0.228873 | 65535 | 0.001439 | 0.69188 | -0.25104 |
| Fp1-Pz | -1.54868 | 0.062068 | 0.205879 | 65535 | 0.000468 | 0.637267 | -0.28275 |
| Fp1-F9 | -0.4139 | 0.339849 | 0.482219 | 65535 | 0.004145 | 0.078793 | -0.07557 |
| Fp1-F10 | -1.57646 | 0.058798 | 0.205793 | 65535 | 0.000291 | 0.506682 | -0.28782 |
| Fp2-F3 | 0.522425 | 0.698823 | 0.744938 | 65535 | 0.007322 | 0.085349 | 0.095381 |
| Fp2-F4 | 0.736444 | 0.768539 | 0.79869 | 65535 | 0.010288 | 0.146825 | 0.134456 |
| Fp2-C3 | -1.33675 | 0.091937 | 0.232461 | 65535 | 0.001179 | 0.407073 | -0.24406 |
| Fp2-C4 | -0.83805 | 0.201849 | 0.357261 | 65535 | 0.00321 | 0.18694 | -0.15301 |
| Fp2-P3 | -1.8588 | 0.032773 | 0.158315 | 65535 | -0.00106 | 0.71384 | -0.33937 |
| Fp2-P4 | -1.27532 | 0.102351 | 0.241939 | 65535 | 0.001853 | 0.498309 | -0.23284 |
| Fp2-O1 | -2.55109 | 0.006009 | 0.103034 | 65535 | -0.00538 | 0.998005 | -0.46576 |
| Fp2-O2 | -1.95282 | 0.026605 | 0.155194 | 65535 | -0.00123 | 0.837815 | -0.35653 |
| Fp2-F7 | -0.11436 | 0.454572 | 0.572551 | 65535 | 0.005376 | 0.051929 | -0.02088 |
| Fp2-F8 | 1.147355 | 0.873222 | 0.890178 | 65535 | 0.009388 | 0.226893 | 0.209477 |
| Fp2-T3 | -1.70831 | 0.045104 | 0.184294 | 65535 | -0.00022 | 0.654704 | -0.31189 |
| Fp2-T4 | -0.64955 | 0.258622 | 0.411445 | 65535 | 0.003822 | 0.148042 | -0.11859 |
| Fp2-T5 | -0.78718 | 0.216377 | 0.37553 | 65535 | 0.003819 | 0.146857 | -0.14372 |
| Fp2-T6 | -1.04308 | 0.149523 | 0.299875 | 65535 | 0.003104 | 0.277801 | -0.19044 |
| Fp2-Fz | -0.18522 | 0.426688 | 0.56355 | 65535 | 0.005377 | 0.054594 | -0.03382 |
| Fp2-Cz | -0.77504 | 0.219934 | 0.378574 | 65535 | 0.004163 | 0.209205 | -0.1415 |
| Fp2-Pz | -1.05644 | 0.146462 | 0.299875 | 65535 | 0.002618 | 0.311451 | -0.19288 |
| Fp2-F9 | -0.31392 | 0.377067 | 0.510866 | 65535 | 0.003824 | 0.06462 | -0.05731 |
| Fp2-F10 | -0.69493 | 0.244233 | 0.400571 | 65535 | 0.003291 | 0.13466 | -0.12688 |
| F3-F4 | -0.5814 | 0.281038 | 0.429629 | 65535 | 0.003435 | 0.085122 | -0.10615 |
| F3-C3 | 0.079341 | 0.531552 | 0.641528 | 65535 | 0.009298 | 0.051482 | 0.014486 |
| F3-C4 | 0.109546 | 0.543522 | 0.642296 | 65535 | 0.005969 | 0.051548 | 0.02 |
| F3-P3 | -1.38254 | 0.084708 | 0.228873 | 65535 | 0.001171 | 0.485767 | -0.25242 |
| F3-P4 | -0.13308 | 0.447178 | 0.569136 | 65535 | 0.006331 | 0.053772 | -0.0243 |
| F3-O1 | -0.4817 | 0.315455 | 0.460039 | 65535 | 0.004837 | 0.12499 | -0.08795 |
| F3-O2 | -1.15475 | 0.125263 | 0.276898 | 65535 | 0.001538 | 0.274381 | -0.21083 |
| F3-F7 | 0.24458 | 0.596397 | 0.667017 | 65535 | 0.008405 | 0.066294 | 0.044654 |
| F3-F8 | 1.272045 | 0.897071 | 0.910072 | 65535 | 0.009821 | 0.247893 | 0.232243 |
| F3-T3 | 0.181489 | 0.571852 | 0.663475 | 65535 | 0.008867 | 0.061189 | 0.033135 |
| F3-T4 | 1.557499 | 0.938985 | 0.938985 | 65535 | 0.010486 | 0.455574 | 0.284359 |
| F3-T5 | -0.97054 | 0.166882 | 0.314032 | 65535 | 0.00341 | 0.326571 | -0.17719 |
| F3-T6 | 0.688307 | 0.753695 | 0.787442 | 65535 | 0.011238 | 0.248018 | 0.125667 |
| F3-Fz | -0.32448 | 0.373074 | 0.510761 | 65535 | 0.005176 | 0.066755 | -0.05924 |
| F3-Cz | -0.53485 | 0.29688 | 0.442162 | 65535 | 0.005309 | 0.101092 | -0.09765 |
| F3-Pz | -0.72685 | 0.23438 | 0.387557 | 65535 | 0.0042 | 0.237517 | -0.1327 |
| F3-F9 | 1.398085 | 0.917645 | 0.922036 | 65535 | 0.010969 | 0.305927 | 0.255254 |
| F3-F10 | 0.233703 | 0.59219 | 0.667017 | 65535 | 0.009075 | 0.069752 | 0.042668 |
| F4-C3 | -0.59662 | 0.275952 | 0.429629 | 65535 | 0.003652 | 0.103015 | -0.10893 |
| F4-C4 | -0.57231 | 0.284101 | 0.429629 | 65535 | 0.004442 | 0.101505 | -0.10449 |
| F4-P3 | -0.73656 | 0.231426 | 0.387557 | 65535 | 0.002901 | 0.109251 | -0.13448 |
| F4-P4 | -0.66349 | 0.254156 | 0.407426 | 65535 | 0.004224 | 0.139228 | -0.12114 |
| F4-O1 | 0.026876 | 0.510698 | 0.619923 | 65535 | 0.006368 | 0.050203 | 0.004907 |
| F4-O2 | -0.72777 | 0.234098 | 0.387557 | 65535 | 0.003044 | 0.133613 | -0.13287 |
| F4-F7 | -0.74899 | 0.227676 | 0.38558 | 65535 | 0.003455 | 0.13181 | -0.13675 |
| F4-F8 | -0.15686 | 0.43781 | 0.566518 | 65535 | 0.007438 | 0.056853 | -0.02864 |
| F4-T3 | -1.38736 | 0.083974 | 0.228873 | 65535 | 0.001103 | 0.560702 | -0.2533 |
| F4-T4 | 0.020339 | 0.508096 | 0.619923 | 65535 | 0.00959 | 0.050154 | 0.003713 |
| F4-T5 | 0.246503 | 0.597139 | 0.667017 | 65535 | 0.007355 | 0.066825 | 0.045005 |
| F4-T6 | -1.08678 | 0.139674 | 0.299875 | 65535 | 0.002275 | 0.320939 | -0.19842 |
| F4-Fz | 0.205238 | 0.58113 | 0.667017 | 65535 | 0.005944 | 0.055281 | 0.037471 |
| F4-Cz | 0.324654 | 0.626991 | 0.69299 | 65535 | 0.007083 | 0.061941 | 0.059273 |
| F4-Pz | -0.36698 | 0.357144 | 0.499645 | 65535 | 0.005221 | 0.083638 | -0.067 |
| F4-F9 | 0.294342 | 0.615493 | 0.683881 | 65535 | 0.008876 | 0.076342 | 0.053739 |
| F4-F10 | -1.39446 | 0.082899 | 0.228873 | 65535 | 0.000954 | 0.367293 | -0.25459 |
| C3-C4 | 0.358599 | 0.639732 | 0.699412 | 65535 | 0.007515 | 0.070091 | 0.065471 |
| C3-P3 | -1.8563 | 0.032953 | 0.158315 | 65535 | -0.00073 | 0.608561 | -0.33891 |
| C3-P4 | -0.92933 | 0.177308 | 0.32662 | 65535 | 0.003058 | 0.205076 | -0.16967 |
| C3-O1 | -1.52286 | 0.065235 | 0.207565 | 65535 | 0.000934 | 0.915283 | -0.27803 |
| C3-O2 | -2.04976 | 0.021302 | 0.149114 | 65535 | -0.00147 | 0.827258 | -0.37423 |
| C3-F7 | -1.40855 | 0.080799 | 0.228873 | 65535 | 0.001059 | 0.560248 | -0.25716 |
| C3-F8 | 0.504259 | 0.69249 | 0.741954 | 65535 | 0.007214 | 0.088784 | 0.092065 |
| C3-T3 | -2.0549 | 0.021048 | 0.149114 | 65535 | -0.00243 | 0.992907 | -0.37517 |
| C3-T4 | 0.365305 | 0.642231 | 0.699412 | 65535 | 0.00617 | 0.076757 | 0.066695 |
| C3-T5 | -0.8377 | 0.201945 | 0.357261 | 65535 | 0.003256 | 0.151839 | -0.15294 |
| C3-T6 | -1.40066 | 0.08197 | 0.228873 | 65535 | 0.001082 | 0.542958 | -0.25572 |
| C3-Fz | 0.170364 | 0.567492 | 0.662074 | 65535 | 0.006395 | 0.053813 | 0.031104 |
| C3-Cz | 0.100218 | 0.539829 | 0.642296 | 65535 | 0.005491 | 0.051203 | 0.018297 |
| C3-Pz | -1.96023 | 0.026163 | 0.155194 | 65535 | -0.0013 | 0.778242 | -0.35789 |
| C3-F9 | -0.16673 | 0.433934 | 0.566518 | 65535 | 0.005354 | 0.054475 | -0.03044 |
| C3-F10 | -1.5103 | 0.06682 | 0.209437 | 65535 | 0.000708 | 0.751128 | -0.27574 |
| C4-P3 | -1.90862 | 0.02937 | 0.158146 | 65535 | -0.00095 | 0.563383 | -0.34847 |
| C4-P4 | -0.75086 | 0.227115 | 0.38558 | 65535 | 0.003999 | 0.182586 | -0.13709 |
| C4-O1 | -0.32055 | 0.374558 | 0.510761 | 65535 | 0.006667 | 0.090051 | -0.05852 |
| C4-O2 | 0.237913 | 0.593819 | 0.667017 | 65535 | 0.007081 | 0.060442 | 0.043437 |
| C4-F7 | 0.38058 | 0.6479 | 0.701335 | 65535 | 0.007347 | 0.080415 | 0.069484 |
| C4-F8 | 1.042157 | 0.850265 | 0.871003 | 65535 | 0.011385 | 0.277935 | 0.190271 |
| C4-T3 | -2.21027 | 0.014508 | 0.142486 | 65535 | -0.00221 | 0.910254 | -0.40354 |
| C4-T4 | -0.68907 | 0.246065 | 0.400571 | 65535 | 0.004305 | 0.216245 | -0.12581 |
| C4-T5 | -0.5715 | 0.284373 | 0.429629 | 65535 | 0.003594 | 0.114685 | -0.10434 |
| C4-T6 | -0.14519 | 0.442404 | 0.566518 | 65535 | 0.006609 | 0.052897 | -0.02651 |
| C4-Fz | -0.50979 | 0.305576 | 0.451908 | 65535 | 0.003908 | 0.096704 | -0.09307 |
| C4-Cz | -0.83591 | 0.202448 | 0.357261 | 65535 | 0.002997 | 0.185389 | -0.15262 |
| C4-Pz | -1.02245 | 0.15433 | 0.300087 | 65535 | 0.002785 | 0.334865 | -0.18667 |
| C4-F9 | -0.3793 | 0.352573 | 0.496915 | 65535 | 0.004246 | 0.073556 | -0.06925 |
| C4-F10 | -0.93346 | 0.176244 | 0.32662 | 65535 | 0.002685 | 0.277442 | -0.17043 |
| P3-P4 | -2.0597 | 0.020813 | 0.149114 | 65535 | -0.00153 | 0.729858 | -0.37605 |
| P3-O1 | -1.70702 | 0.045224 | 0.184294 | 65535 | -0.00029 | 0.550513 | -0.31166 |
| P3-O2 | -1.55731 | 0.061038 | 0.205879 | 65535 | 0.000488 | 0.336484 | -0.28432 |
| P3-F7 | -1.93751 | 0.027536 | 0.156287 | 65535 | -0.00156 | 0.986587 | -0.35374 |
| P3-F8 | -0.9174 | 0.180403 | 0.329432 | 65535 | 0.003507 | 0.158084 | -0.16749 |
| P3-T3 | -2.63133 | 0.00482 | 0.103034 | 65535 | -0.00575 | 0.978506 | -0.48041 |
| P3-T4 | -0.19551 | 0.422665 | 0.56177 | 65535 | 0.005992 | 0.055313 | -0.03569 |
| P3-T5 | -1.80903 | 0.036496 | 0.163069 | 65535 | -0.00058 | 0.539683 | -0.33028 |
| P3-T6 | -2.4407 | 0.008072 | 0.105948 | 65535 | -0.00281 | 0.916481 | -0.44561 |
| P3-Fz | -2.5597 | 0.00587 | 0.103034 | 65535 | -0.00291 | 0.784331 | -0.46733 |
| P3-Cz | -2.48307 | 0.007215 | 0.103034 | 65535 | -0.00311 | 0.97162 | -0.45335 |
| P3-Pz | -1.45738 | 0.073834 | 0.224713 | 65535 | 0.000855 | 0.443934 | -0.26608 |
| P3-F9 | -0.5922 | 0.277425 | 0.429629 | 65535 | 0.004905 | 0.084779 | -0.10812 |
| P3-F10 | -1.84979 | 0.033423 | 0.158315 | 65535 | -0.00088 | 0.888677 | -0.33772 |
| P4-O1 | -1.87152 | 0.031875 | 0.158315 | 65535 | -0.00094 | 0.863918 | -0.34169 |
| P4-O2 | -1.18126 | 0.119936 | 0.267943 | 65535 | 0.001973 | 0.262846 | -0.21567 |
| P4-F7 | -1.6842 | 0.047393 | 0.184307 | 65535 | -0.00012 | 0.666989 | -0.30749 |
| P4-F8 | -1.07409 | 0.142487 | 0.299875 | 65535 | 0.002853 | 0.316455 | -0.1961 |
| P4-T3 | -1.3735 | 0.0861 | 0.228873 | 65535 | 0.001437 | 0.556424 | -0.25077 |
| P4-T4 | -1.21615 | 0.113178 | 0.258342 | 65535 | 0.002301 | 0.431549 | -0.22204 |
| P4-T5 | -0.96811 | 0.167484 | 0.314032 | 65535 | 0.002288 | 0.211314 | -0.17675 |
| P4-T6 | -1.23591 | 0.109473 | 0.25263 | 65535 | 0.002328 | 0.344039 | -0.22565 |
| P4-Fz | -1.2633 | 0.104485 | 0.243799 | 65535 | 0.001511 | 0.576151 | -0.23065 |
| P4-Cz | -1.32129 | 0.09448 | 0.233422 | 65535 | 0.001245 | 0.4249 | -0.24123 |
| P4-Pz | -1.79592 | 0.037533 | 0.164208 | 65535 | -0.0006 | 0.787666 | -0.32789 |
| P4-F9 | 0.215042 | 0.584947 | 0.667017 | 65535 | 0.007311 | 0.057256 | 0.039261 |
| P4-F10 | -1.43745 | 0.076619 | 0.228873 | 65535 | 0.001065 | 0.652217 | -0.26244 |
| O1-O2 | -1.19109 | 0.118004 | 0.266461 | 65535 | 0.00213 | 0.463515 | -0.21746 |
| O1-F7 | -2.6809 | 0.004197 | 0.103034 | 65535 | -0.00651 | 1 | -0.48946 |
| O1-F8 | -1.34392 | 0.090776 | 0.232461 | 65535 | 0.001787 | 0.679888 | -0.24537 |
| O1-T3 | -2.58184 | 0.005525 | 0.103034 | 65535 | -0.00548 | 0.999809 | -0.47138 |
| O1-T4 | -2.48094 | 0.007257 | 0.103034 | 65535 | -0.00467 | 0.999451 | -0.45295 |
| O1-T5 | -2.13399 | 0.017457 | 0.146636 | 65535 | -0.00264 | 0.749457 | -0.38961 |
| O1-T6 | -1.56192 | 0.060493 | 0.205879 | 65535 | 0.000389 | 0.565887 | -0.28517 |
| O1-Fz | -1.28001 | 0.101526 | 0.241939 | 65535 | 0.001508 | 0.526853 | -0.2337 |
| O1-Cz | -1.63564 | 0.05229 | 0.199651 | 65535 | 0.000139 | 0.889014 | -0.29863 |
| O1-Pz | -1.0074 | 0.157902 | 0.304215 | 65535 | 0.003503 | 0.410254 | -0.18393 |
| O1-F9 | -2.16533 | 0.016188 | 0.142486 | 65535 | -0.0028 | 0.98239 | -0.39533 |
| O1-F10 | -1.36157 | 0.087964 | 0.230906 | 65535 | 0.001631 | 0.788206 | -0.24859 |
| O2-F7 | -3.14915 | 0.001037 | 0.075722 | 65535 | -0.00554 | 0.995486 | -0.57495 |
| O2-F8 | -1.02784 | 0.153064 | 0.300087 | 65535 | 0.002884 | 0.182346 | -0.18766 |
| O2-T3 | -3.13582 | 0.001082 | 0.075722 | 65535 | -0.0064 | 0.999189 | -0.57252 |
| O2-T4 | -1.83277 | 0.034679 | 0.158315 | 65535 | -0.00072 | 0.750002 | -0.33462 |
| O2-T5 | -0.82642 | 0.205116 | 0.358953 | 65535 | 0.003339 | 0.13311 | -0.15088 |
| O2-T6 | -2.16287 | 0.016284 | 0.142486 | 65535 | -0.00224 | 0.855024 | -0.39488 |
| O2-Fz | -0.5466 | 0.292842 | 0.439263 | 65535 | 0.003817 | 0.114831 | -0.0998 |
| O2-Cz | -2.37856 | 0.009493 | 0.10967 | 65535 | -0.00336 | 0.997331 | -0.43426 |
| O2-Pz | -1.70263 | 0.045635 | 0.184294 | 65535 | -0.00018 | 0.614752 | -0.31086 |
| O2-F9 | -1.54308 | 0.062744 | 0.205879 | 65535 | 0.000396 | 0.484385 | -0.28173 |
| O2-F10 | -2.47675 | 0.007338 | 0.103034 | 65535 | -0.00362 | 0.98452 | -0.45219 |
| F7-F8 | 0.826536 | 0.794916 | 0.818296 | 65535 | 0.007986 | 0.154499 | 0.150904 |
| F7-T3 | -1.59221 | 0.057006 | 0.202904 | 65535 | 0.000375 | 0.84512 | -0.2907 |
| F7-T4 | -0.09657 | 0.461617 | 0.577021 | 65535 | 0.0043 | 0.051298 | -0.01763 |
| F7-T5 | -0.85303 | 0.197685 | 0.357261 | 65535 | 0.004041 | 0.248963 | -0.15574 |
| F7-T6 | -1.53201 | 0.064099 | 0.207088 | 65535 | 0.000625 | 0.796048 | -0.2797 |
| F7-Fz | 0.005743 | 0.502286 | 0.616843 | 65535 | 0.006174 | 0.050006 | 0.001049 |
| F7-Cz | -1.11737 | 0.133052 | 0.291051 | 65535 | 0.003041 | 0.353956 | -0.204 |
| F7-Pz | -1.04235 | 0.149691 | 0.299875 | 65535 | 0.003386 | 0.385582 | -0.19031 |
| F7-F9 | -0.25934 | 0.397913 | 0.535652 | 65535 | 0.004663 | 0.061625 | -0.04735 |
| F7-F10 | -1.84031 | 0.034118 | 0.158315 | 65535 | -0.00094 | 0.951937 | -0.33599 |
| F8-T3 | -2.52293 | 0.006485 | 0.103034 | 65535 | -0.00316 | 0.966742 | -0.46062 |
| F8-T4 | -0.16561 | 0.434374 | 0.566518 | 65535 | 0.008218 | 0.060476 | -0.03024 |
| F8-T5 | 0.44455 | 0.671271 | 0.722907 | 65535 | 0.010506 | 0.074809 | 0.081163 |
| F8-T6 | -0.61122 | 0.271114 | 0.428075 | 65535 | 0.005355 | 0.110517 | -0.11159 |
| F8-Fz | 0.748136 | 0.772067 | 0.79869 | 65535 | 0.007693 | 0.140352 | 0.13659 |
| F8-Cz | -0.11249 | 0.455314 | 0.572551 | 65535 | 0.006144 | 0.051681 | -0.02054 |
| F8-Pz | -0.49449 | 0.310938 | 0.456623 | 65535 | 0.00465 | 0.100493 | -0.09028 |
| F8-F9 | 0.624025 | 0.733092 | 0.769747 | 65535 | 0.007625 | 0.114025 | 0.113931 |
| F8-F10 | -0.05017 | 0.480037 | 0.596495 | 65535 | 0.005419 | 0.050443 | -0.00916 |
| T3-T4 | -2.95036 | 0.001914 | 0.080407 | 65535 | -0.00404 | 0.91533 | -0.53866 |
| T3-T5 | -1.86031 | 0.032666 | 0.158315 | 65535 | -0.00113 | 0.689813 | -0.33964 |
| T3-T6 | -1.62322 | 0.053605 | 0.200655 | 65535 | 0.000137 | 0.651696 | -0.29636 |
| T3-Fz | -0.99721 | 0.16035 | 0.306124 | 65535 | 0.002292 | 0.23443 | -0.18207 |
| T3-Cz | -2.00649 | 0.023545 | 0.155194 | 65535 | -0.00166 | 0.933762 | -0.36633 |
| T3-Pz | -1.60649 | 0.055419 | 0.200655 | 65535 | 0.00025 | 0.701717 | -0.2933 |
| T3-F9 | -1.97066 | 0.025552 | 0.155194 | 65535 | -0.00127 | 0.850482 | -0.35979 |
| T3-F10 | -2.47564 | 0.00736 | 0.103034 | 65535 | -0.00248 | 0.907088 | -0.45199 |
| T4-T5 | -1.91919 | 0.028688 | 0.158146 | 65535 | -0.00111 | 0.666629 | -0.35039 |
| T4-T6 | -1.33177 | 0.092752 | 0.232461 | 65535 | 0.001669 | 0.378019 | -0.24315 |
| T4-Fz | 0.11182 | 0.544422 | 0.642296 | 65535 | 0.00594 | 0.05218 | 0.020416 |
| T4-Cz | -1.42338 | 0.078632 | 0.228873 | 65535 | 0.000894 | 0.441237 | -0.25987 |
| T4-Pz | -0.36128 | 0.359268 | 0.499645 | 65535 | 0.005503 | 0.080868 | -0.06596 |
| T4-F9 | -2.06583 | 0.020517 | 0.149114 | 65535 | -0.00161 | 0.848567 | -0.37717 |
| T4-F10 | -0.67212 | 0.25141 | 0.406124 | 65535 | 0.003196 | 0.156377 | -0.12271 |
| T5-T6 | -2.1888 | 0.01529 | 0.142486 | 65535 | -0.00196 | 0.915612 | -0.39962 |
| T5-Fz | 0.603441 | 0.726313 | 0.766461 | 65535 | 0.007816 | 0.09919 | 0.110173 |
| T5-Cz | -1.04253 | 0.149648 | 0.299875 | 65535 | 0.002191 | 0.257058 | -0.19034 |
| T5-Pz | -1.41585 | 0.079727 | 0.228873 | 65535 | 0.000927 | 0.415408 | -0.2585 |
| T5-F9 | -1.04128 | 0.149938 | 0.299875 | 65535 | 0.002615 | 0.196368 | -0.19011 |
| T5-F10 | -1.97256 | 0.025442 | 0.155194 | 65535 | -0.00137 | 0.829292 | -0.36014 |
| T6-Fz | -0.44676 | 0.327933 | 0.474937 | 65535 | 0.004084 | 0.08726 | -0.08157 |
| T6-Cz | -1.68749 | 0.047075 | 0.184307 | 65535 | -0.00012 | 0.66205 | -0.30809 |
| T6-Pz | -1.73271 | 0.04288 | 0.183771 | 65535 | -0.00032 | 0.687467 | -0.31635 |
| T6-F9 | -1.49891 | 0.068283 | 0.210875 | 65535 | 0.000717 | 0.574566 | -0.27366 |
| T6-F10 | -1.61329 | 0.054677 | 0.200655 | 65535 | 0.000183 | 0.666289 | -0.29454 |
| Fz-Cz | 0.366815 | 0.642793 | 0.699412 | 65535 | 0.007215 | 0.067288 | 0.066971 |
| Fz-Pz | -2.37181 | 0.00966 | 0.10967 | 65535 | -0.00254 | 0.937879 | -0.43303 |
| Fz-F9 | 1.307093 | 0.903139 | 0.911823 | 65535 | 0.009067 | 0.335167 | 0.238641 |
| Fz-F10 | -0.14514 | 0.442424 | 0.566518 | 65535 | 0.00507 | 0.054054 | -0.0265 |
| Cz-Pz | -3.15006 | 0.001034 | 0.075722 | 65535 | -0.00678 | 0.999985 | -0.57512 |
| Cz-F9 | -1.03209 | 0.15207 | 0.300087 | 65535 | 0.002224 | 0.217231 | -0.18843 |
| Cz-F10 | -2.36139 | 0.009923 | 0.10967 | 65535 | -0.00274 | 0.866394 | -0.43113 |
| Pz-F9 | -0.02132 | 0.491514 | 0.607165 | 65535 | 0.006942 | 0.050098 | -0.00389 |
| Pz-F10 | -1.04975 | 0.147988 | 0.299875 | 65535 | 0.002442 | 0.273161 | -0.19166 |
| F9-F10 | -1.04497 | 0.149087 | 0.299875 | 65535 | 0.002021 | 0.278138 | -0.19078 |

Table S4i: gamma_wpli_HC_PT

| channel | tvalue | pvalue | fdr_pvalue | CI_low | CI_high | statistical power | effect size |
| --- | --- | --- | --- | --- | --- | --- | --- |
| Fp1-Fp2 | -0.43337 | 0.667232 | 0.980416 | -0.00572 | 65535 | 0.077448 | -0.07912 |
| Fp1-F3 | 0.309444 | 0.378765 | 0.980416 | -0.00706 | 65535 | 0.076418 | 0.056496 |
| Fp1-F4 | -0.1668 | 0.566092 | 0.980416 | -0.00988 | 65535 | 0.053611 | -0.03045 |
| Fp1-C3 | 0.316552 | 0.376072 | 0.980416 | -0.00555 | 65535 | 0.059716 | 0.057794 |
| Fp1-C4 | -1.13477 | 0.870615 | 0.980416 | -0.00952 | 65535 | 0.376197 | -0.20718 |
| Fp1-P3 | 0.38128 | 0.35184 | 0.980416 | -0.00591 | 65535 | 0.076901 | 0.069612 |
| Fp1-P4 | 0.307168 | 0.379628 | 0.980416 | -0.0039 | 65535 | 0.058071 | 0.056081 |
| Fp1-O1 | 0.556213 | 0.289559 | 0.980416 | -0.0029 | 65535 | 0.080986 | 0.10155 |
| Fp1-O2 | 0.579906 | 0.281542 | 0.980416 | -0.00253 | 65535 | 0.077229 | 0.105876 |
| Fp1-F7 | 0.25242 | 0.400578 | 0.980416 | -0.00648 | 65535 | 0.056095 | 0.046085 |
| Fp1-F8 | 0.570853 | 0.284592 | 0.980416 | -0.00545 | 65535 | 0.076419 | 0.104223 |
| Fp1-T3 | -0.52631 | 0.700171 | 0.980416 | -0.00604 | 65535 | 0.075264 | -0.09609 |
| Fp1-T4 | -0.01419 | 0.50565 | 0.980416 | -0.0047 | 65535 | 0.050022 | -0.00259 |
| Fp1-T5 | -0.05056 | 0.520121 | 0.980416 | -0.00524 | 65535 | 0.050325 | -0.00923 |
| Fp1-T6 | 1.118318 | 0.132851 | 0.980416 | -0.0019 | 65535 | 0.147481 | 0.204176 |
| Fp1-Fz | 0.637822 | 0.262413 | 0.980416 | -0.00502 | 65535 | 0.083805 | 0.11645 |
| Fp1-Cz | -0.78094 | 0.7818 | 0.980416 | -0.01335 | 65535 | 0.279819 | -0.14258 |
| Fp1-Pz | -1.88029 | 0.968733 | 0.988286 | -0.00763 | 65535 | 0.681508 | -0.34329 |
| Fp1-F9 | -0.61619 | 0.730523 | 0.980416 | -0.00631 | 65535 | 0.117794 | -0.1125 |
| Fp1-F10 | -0.77913 | 0.781268 | 0.980416 | -0.00577 | 65535 | 0.117052 | -0.14225 |
| Fp2-F3 | -0.65915 | 0.74446 | 0.980416 | -0.01252 | 65535 | 0.219586 | -0.12034 |
| Fp2-F4 | -0.34865 | 0.636014 | 0.980416 | -0.01063 | 65535 | 0.067795 | -0.06365 |
| Fp2-C3 | -1.40251 | 0.918306 | 0.980416 | -0.01177 | 65535 | 0.523518 | -0.25606 |
| Fp2-C4 | -1.34663 | 0.90966 | 0.980416 | -0.01192 | 65535 | 0.731518 | -0.24586 |
| Fp2-P3 | -0.16049 | 0.563614 | 0.980416 | -0.00811 | 65535 | 0.054679 | -0.0293 |
| Fp2-P4 | -1.41838 | 0.920642 | 0.980416 | -0.01007 | 65535 | 0.359031 | -0.25896 |
| Fp2-O1 | 0.427931 | 0.334741 | 0.980416 | -0.00314 | 65535 | 0.069883 | 0.078129 |
| Fp2-O2 | -0.42408 | 0.66386 | 0.980416 | -0.00605 | 65535 | 0.073285 | -0.07743 |
| Fp2-F7 | -0.63685 | 0.737273 | 0.980416 | -0.00878 | 65535 | 0.092259 | -0.11627 |
| Fp2-F8 | 0.170194 | 0.432575 | 0.980416 | -0.0078 | 65535 | 0.052371 | 0.031073 |
| Fp2-T3 | -1.27835 | 0.898183 | 0.980416 | -0.00708 | 65535 | 0.233582 | -0.23339 |
| Fp2-T4 | -0.99603 | 0.839363 | 0.980416 | -0.0078 | 65535 | 0.260105 | -0.18185 |
| Fp2-T5 | -0.22886 | 0.590312 | 0.980416 | -0.00604 | 65535 | 0.057447 | -0.04178 |
| Fp2-T6 | 0.705893 | 0.240823 | 0.980416 | -0.00359 | 65535 | 0.097303 | 0.128878 |
| Fp2-Fz | -0.38262 | 0.648654 | 0.980416 | -0.01195 | 65535 | 0.069159 | -0.06986 |
| Fp2-Cz | -1.28383 | 0.899141 | 0.980416 | -0.01941 | 65535 | 0.888091 | -0.23439 |
| Fp2-Pz | -1.92667 | 0.971787 | 0.988286 | -0.00896 | 65535 | 0.758831 | -0.35176 |
| Fp2-F9 | -1.35968 | 0.911738 | 0.980416 | -0.00825 | 65535 | 0.385607 | -0.24824 |
| Fp2-F10 | -1.22621 | 0.888719 | 0.980416 | -0.00718 | 65535 | 0.266232 | -0.22387 |
| F3-F4 | -0.57507 | 0.71683 | 0.980416 | -0.01263 | 65535 | 0.115297 | -0.10499 |
| F3-C3 | 0.044869 | 0.482144 | 0.980416 | -0.00752 | 65535 | 0.050328 | 0.008192 |
| F3-C4 | -0.22699 | 0.589586 | 0.980416 | -0.00587 | 65535 | 0.060693 | -0.04144 |
| F3-P3 | 0.362817 | 0.358695 | 0.980416 | -0.00572 | 65535 | 0.071921 | 0.066241 |
| F3-P4 | -0.82057 | 0.793226 | 0.980416 | -0.00644 | 65535 | 0.216212 | -0.14981 |
| F3-O1 | 0.809905 | 0.209812 | 0.980416 | -0.0021 | 65535 | 0.119997 | 0.147868 |
| F3-O2 | -1.11522 | 0.866488 | 0.980416 | -0.00594 | 65535 | 0.226636 | -0.20361 |
| F3-F7 | 0.775832 | 0.2197 | 0.980416 | -0.00343 | 65535 | 0.120788 | 0.141647 |
| F3-F8 | 0.213167 | 0.415782 | 0.980416 | -0.00675 | 65535 | 0.05846 | 0.038919 |
| F3-T3 | 0.003821 | 0.498479 | 0.980416 | -0.00622 | 65535 | 0.050003 | 0.000698 |
| F3-T4 | -0.12248 | 0.548637 | 0.980416 | -0.00434 | 65535 | 0.051769 | -0.02236 |
| F3-T5 | 0.235236 | 0.407217 | 0.980416 | -0.00483 | 65535 | 0.055386 | 0.042948 |
| F3-T6 | -0.4654 | 0.678747 | 0.980416 | -0.00578 | 65535 | 0.084347 | -0.08497 |
| F3-Fz | -0.37971 | 0.64758 | 0.980416 | -0.01159 | 65535 | 0.083133 | -0.06933 |
| F3-Cz | -0.3198 | 0.625159 | 0.980416 | -0.00995 | 65535 | 0.078816 | -0.05839 |
| F3-Pz | -1.60049 | 0.943918 | 0.980677 | -0.00977 | 65535 | 0.756426 | -0.29221 |
| F3-F9 | 0.794094 | 0.214367 | 0.980416 | -0.00255 | 65535 | 0.11862 | 0.144981 |
| F3-F10 | -0.15871 | 0.562915 | 0.980416 | -0.00502 | 65535 | 0.054658 | -0.02898 |
| F4-C3 | -0.92507 | 0.821591 | 0.980416 | -0.0113 | 65535 | 0.407701 | -0.16889 |
| F4-C4 | -1.3263 | 0.906348 | 0.980416 | -0.0135 | 65535 | 0.532073 | -0.24215 |
| F4-P3 | 0.409247 | 0.34155 | 0.980416 | -0.00478 | 65535 | 0.067315 | 0.074718 |
| F4-P4 | -0.7284 | 0.766093 | 0.980416 | -0.00974 | 65535 | 0.136004 | -0.13299 |
| F4-O1 | 0.350267 | 0.363381 | 0.980416 | -0.00348 | 65535 | 0.063991 | 0.06395 |
| F4-O2 | -0.76996 | 0.77857 | 0.980416 | -0.00636 | 65535 | 0.131564 | -0.14058 |
| F4-F7 | -0.15058 | 0.559719 | 0.980416 | -0.00679 | 65535 | 0.052344 | -0.02749 |
| F4-F8 | -0.22463 | 0.588672 | 0.980416 | -0.0081 | 65535 | 0.056841 | -0.04101 |
| F4-T3 | -0.608 | 0.72782 | 0.980416 | -0.00658 | 65535 | 0.099754 | -0.111 |
| F4-T4 | -0.39283 | 0.652423 | 0.980416 | -0.0076 | 65535 | 0.077162 | -0.07172 |
| F4-T5 | 0.294128 | 0.384588 | 0.980416 | -0.00352 | 65535 | 0.061034 | 0.0537 |
| F4-T6 | 0.402225 | 0.344123 | 0.980416 | -0.00487 | 65535 | 0.064093 | 0.073436 |
| F4-Fz | -0.41734 | 0.661407 | 0.980416 | -0.01315 | 65535 | 0.076799 | -0.0762 |
| F4-Cz | -0.27817 | 0.609316 | 0.980416 | -0.01252 | 65535 | 0.065657 | -0.05079 |
| F4-Pz | -0.62157 | 0.732289 | 0.980416 | -0.00708 | 65535 | 0.114259 | -0.11348 |
| F4-F9 | -0.23338 | 0.592063 | 0.980416 | -0.00594 | 65535 | 0.058577 | -0.04261 |
| F4-F10 | -0.4479 | 0.672478 | 0.980416 | -0.0062 | 65535 | 0.069699 | -0.08178 |
| C3-C4 | -0.95782 | 0.829945 | 0.980416 | -0.00763 | 65535 | 0.32227 | -0.17487 |
| C3-P3 | -0.45972 | 0.67672 | 0.980416 | -0.01219 | 65535 | 0.08562 | -0.08393 |
| C3-P4 | -1.33955 | 0.908517 | 0.980416 | -0.00766 | 65535 | 0.381986 | -0.24457 |
| C3-O1 | -0.15349 | 0.560864 | 0.980416 | -0.00495 | 65535 | 0.052503 | -0.02802 |
| C3-O2 | -0.73358 | 0.767671 | 0.980416 | -0.00508 | 65535 | 0.131186 | -0.13393 |
| C3-F7 | -0.215 | 0.584932 | 0.980416 | -0.00712 | 65535 | 0.05724 | -0.03925 |
| C3-F8 | -0.22598 | 0.589197 | 0.980416 | -0.00557 | 65535 | 0.058347 | -0.04126 |
| C3-T3 | -0.52748 | 0.700574 | 0.980416 | -0.01036 | 65535 | 0.073375 | -0.0963 |
| C3-T4 | -0.70981 | 0.760389 | 0.980416 | -0.00457 | 65535 | 0.121452 | -0.12959 |
| C3-T5 | -1.51539 | 0.933826 | 0.980677 | -0.01018 | 65535 | 0.474478 | -0.27667 |
| C3-T6 | -0.39799 | 0.654321 | 0.980416 | -0.00465 | 65535 | 0.068721 | -0.07266 |
| C3-Fz | -1.03433 | 0.848452 | 0.980416 | -0.01394 | 65535 | 0.50371 | -0.18884 |
| C3-Cz | -0.2813 | 0.610514 | 0.980416 | -0.00743 | 65535 | 0.067037 | -0.05136 |
| C3-Pz | -1.98835 | 0.975456 | 0.988286 | -0.01253 | 65535 | 0.935347 | -0.36302 |
| C3-F9 | -0.79158 | 0.784904 | 0.980416 | -0.00758 | 65535 | 0.121858 | -0.14452 |
| C3-F10 | -0.42564 | 0.664428 | 0.980416 | -0.00501 | 65535 | 0.074922 | -0.07771 |
| C4-P3 | -0.32525 | 0.627215 | 0.980416 | -0.00621 | 65535 | 0.08259 | -0.05938 |
| C4-P4 | -1.00717 | 0.842043 | 0.980416 | -0.00761 | 65535 | 0.28084 | -0.18388 |
| C4-O1 | -1.13107 | 0.86984 | 0.980416 | -0.00514 | 65535 | 0.211366 | -0.2065 |
| C4-O2 | -0.35652 | 0.638955 | 0.980416 | -0.0038 | 65535 | 0.063345 | -0.06509 |
| C4-F7 | 0.067692 | 0.473073 | 0.980416 | -0.00406 | 65535 | 0.050695 | 0.012359 |
| C4-F8 | -0.62724 | 0.734142 | 0.980416 | -0.00748 | 65535 | 0.134755 | -0.11452 |
| C4-T3 | -1.22224 | 0.887975 | 0.980416 | -0.00623 | 65535 | 0.336998 | -0.22315 |
| C4-T4 | -1.63829 | 0.947988 | 0.980677 | -0.01694 | 65535 | 0.777766 | -0.29911 |
| C4-T5 | -0.51845 | 0.697444 | 0.980416 | -0.00423 | 65535 | 0.100206 | -0.09466 |
| C4-T6 | -0.52844 | 0.700907 | 0.980416 | -0.00549 | 65535 | 0.094716 | -0.09648 |
| C4-Fz | -0.91724 | 0.819556 | 0.980416 | -0.01141 | 65535 | 0.384798 | -0.16746 |
| C4-Cz | -0.28791 | 0.613039 | 0.980416 | -0.00736 | 65535 | 0.071315 | -0.05256 |
| C4-Pz | -1.30523 | 0.902824 | 0.980416 | -0.0081 | 65535 | 0.421204 | -0.2383 |
| C4-F9 | -0.03285 | 0.513075 | 0.980416 | -0.00379 | 65535 | 0.050232 | -0.006 |
| C4-F10 | -0.58129 | 0.718923 | 0.980416 | -0.00432 | 65535 | 0.100741 | -0.10613 |
| P3-P4 | -0.13066 | 0.551866 | 0.980416 | -0.00516 | 65535 | 0.052479 | -0.02385 |
| P3-O1 | 0.082038 | 0.467378 | 0.980416 | -0.00534 | 65535 | 0.050813 | 0.014978 |
| P3-O2 | -0.95311 | 0.828758 | 0.980416 | -0.008 | 65535 | 0.188385 | -0.17401 |
| P3-F7 | 1.277935 | 0.10189 | 0.980416 | -0.00135 | 65535 | 0.210683 | 0.233318 |
| P3-F8 | 0.938746 | 0.174889 | 0.980416 | -0.00261 | 65535 | 0.127523 | 0.171391 |
| P3-T3 | 1.084148 | 0.140255 | 0.980416 | -0.00141 | 65535 | 0.174061 | 0.197937 |
| P3-T4 | 0.587734 | 0.278917 | 0.980416 | -0.00169 | 65535 | 0.084413 | 0.107305 |
| P3-T5 | -0.1395 | 0.555354 | 0.980416 | -0.0073 | 65535 | 0.053989 | -0.02547 |
| P3-T6 | 0.084023 | 0.46659 | 0.980416 | -0.00343 | 65535 | 0.050869 | 0.01534 |
| P3-Fz | -0.30152 | 0.618226 | 0.980416 | -0.00655 | 65535 | 0.063385 | -0.05505 |
| P3-Cz | -0.09907 | 0.539374 | 0.980416 | -0.0076 | 65535 | 0.052674 | -0.01809 |
| P3-Pz | -1.13316 | 0.870277 | 0.980416 | -0.01213 | 65535 | 0.266779 | -0.20688 |
| P3-F9 | 0.538433 | 0.295646 | 0.980416 | -0.00222 | 65535 | 0.076685 | 0.098304 |
| P3-F10 | 0.018766 | 0.49253 | 0.980416 | -0.00369 | 65535 | 0.050032 | 0.003426 |
| P4-O1 | -0.55834 | 0.711165 | 0.980416 | -0.00487 | 65535 | 0.089164 | -0.10194 |
| P4-O2 | -0.76864 | 0.778179 | 0.980416 | -0.0077 | 65535 | 0.128214 | -0.14033 |
| P4-F7 | 0.296719 | 0.383601 | 0.980416 | -0.00351 | 65535 | 0.05741 | 0.054173 |
| P4-F8 | -0.08223 | 0.532697 | 0.980416 | -0.0056 | 65535 | 0.050635 | -0.01501 |
| P4-T3 | -0.33975 | 0.632674 | 0.980416 | -0.00356 | 65535 | 0.069587 | -0.06203 |
| P4-T4 | -0.84572 | 0.800291 | 0.980416 | -0.00457 | 65535 | 0.144104 | -0.15441 |
| P4-T5 | -0.89886 | 0.814721 | 0.980416 | -0.00453 | 65535 | 0.170132 | -0.16411 |
| P4-T6 | 0.496931 | 0.310081 | 0.980416 | -0.00407 | 65535 | 0.083797 | 0.090727 |
| P4-Fz | -1.84471 | 0.966206 | 0.988286 | -0.01073 | 65535 | 0.827445 | -0.3368 |
| P4-Cz | -1.36503 | 0.91258 | 0.980416 | -0.0096 | 65535 | 0.48745 | -0.24922 |
| P4-Pz | -1.27198 | 0.897059 | 0.980416 | -0.01231 | 65535 | 0.579832 | -0.23223 |
| P4-F9 | -0.94622 | 0.827014 | 0.980416 | -0.00578 | 65535 | 0.183061 | -0.17275 |
| P4-F10 | 0.079438 | 0.468409 | 0.980416 | -0.00316 | 65535 | 0.05099 | 0.014503 |
| O1-O2 | -0.6462 | 0.740298 | 0.980416 | -0.00729 | 65535 | 0.117375 | -0.11798 |
| O1-F7 | 0.095172 | 0.46217 | 0.980416 | -0.00331 | 65535 | 0.050925 | 0.017376 |
| O1-F8 | 0.253364 | 0.400214 | 0.980416 | -0.00292 | 65535 | 0.057291 | 0.046258 |
| O1-T3 | 0.329066 | 0.371344 | 0.980416 | -0.00289 | 65535 | 0.061196 | 0.060079 |
| O1-T4 | 0.206799 | 0.418262 | 0.980416 | -0.00245 | 65535 | 0.054375 | 0.037756 |
| O1-T5 | 1.544138 | 0.062616 | 0.980416 | -0.00034 | 65535 | 0.369994 | 0.28192 |
| O1-T6 | 1.302377 | 0.097662 | 0.980416 | -0.00063 | 65535 | 0.251751 | 0.23778 |
| O1-Fz | -0.95286 | 0.828696 | 0.980416 | -0.00669 | 65535 | 0.167752 | -0.17397 |
| O1-Cz | -0.63104 | 0.735381 | 0.980416 | -0.00521 | 65535 | 0.105088 | -0.11521 |
| O1-Pz | -0.9321 | 0.823407 | 0.980416 | -0.0098 | 65535 | 0.381469 | -0.17018 |
| O1-F9 | -0.28877 | 0.613366 | 0.980416 | -0.00384 | 65535 | 0.059134 | -0.05272 |
| O1-F10 | 0.315829 | 0.376345 | 0.980416 | -0.00226 | 65535 | 0.058468 | 0.057662 |
| O2-F7 | -0.15221 | 0.56036 | 0.980416 | -0.00331 | 65535 | 0.052049 | -0.02779 |
| O2-F8 | -0.15652 | 0.562053 | 0.980416 | -0.00389 | 65535 | 0.052534 | -0.02858 |
| O2-T3 | -0.92143 | 0.820646 | 0.980416 | -0.00461 | 65535 | 0.149501 | -0.16823 |
| O2-T4 | 0.080339 | 0.468052 | 0.980416 | -0.00296 | 65535 | 0.050671 | 0.014668 |
| O2-T5 | 0.132379 | 0.447455 | 0.980416 | -0.00276 | 65535 | 0.051879 | 0.024169 |
| O2-T6 | -0.524 | 0.699368 | 0.980416 | -0.00729 | 65535 | 0.104036 | -0.09567 |
| O2-Fz | -0.44265 | 0.670587 | 0.980416 | -0.00512 | 65535 | 0.069985 | -0.08082 |
| O2-Cz | -0.3887 | 0.650902 | 0.980416 | -0.00599 | 65535 | 0.06081 | -0.07097 |
| O2-Pz | -1.08767 | 0.860521 | 0.980416 | -0.00719 | 65535 | 0.185896 | -0.19858 |
| O2-F9 | -0.22164 | 0.587511 | 0.980416 | -0.00353 | 65535 | 0.055815 | -0.04047 |
| O2-F10 | -0.94406 | 0.826465 | 0.980416 | -0.00516 | 65535 | 0.133734 | -0.17236 |
| F7-F8 | 0.341474 | 0.366677 | 0.980416 | -0.0036 | 65535 | 0.06379 | 0.062344 |
| F7-T3 | -1.10785 | 0.86491 | 0.980416 | -0.00852 | 65535 | 0.208341 | -0.20226 |
| F7-T4 | 0.701781 | 0.242099 | 0.980416 | -0.00203 | 65535 | 0.088173 | 0.128127 |
| F7-T5 | 0.15481 | 0.438618 | 0.980416 | -0.00365 | 65535 | 0.052591 | 0.028264 |
| F7-T6 | 0.591883 | 0.27753 | 0.980416 | -0.00327 | 65535 | 0.073769 | 0.108063 |
| F7-Fz | 0.602091 | 0.274134 | 0.980416 | -0.00568 | 65535 | 0.079919 | 0.109926 |
| F7-Cz | -0.46049 | 0.676994 | 0.980416 | -0.00846 | 65535 | 0.083181 | -0.08407 |
| F7-Pz | -1.44462 | 0.924392 | 0.980416 | -0.0063 | 65535 | 0.322237 | -0.26375 |
| F7-F9 | -0.56134 | 0.712184 | 0.980416 | -0.00529 | 65535 | 0.087921 | -0.10249 |
| F7-F10 | 0.500771 | 0.308732 | 0.980416 | -0.00277 | 65535 | 0.070918 | 0.091428 |
| F8-T3 | -0.02792 | 0.511114 | 0.980416 | -0.00471 | 65535 | 0.050076 | -0.0051 |
| F8-T4 | 1.226814 | 0.111167 | 0.980416 | -0.00114 | 65535 | 0.20908 | 0.223985 |
| F8-T5 | 0.171758 | 0.431961 | 0.980416 | -0.00365 | 65535 | 0.054078 | 0.031358 |
| F8-T6 | 1.508163 | 0.067093 | 0.980416 | -0.00051 | 65535 | 0.235119 | 0.275352 |
| F8-Fz | 0.39635 | 0.346281 | 0.980416 | -0.00694 | 65535 | 0.0683 | 0.072363 |
| F8-Cz | -0.08266 | 0.532869 | 0.980416 | -0.00831 | 65535 | 0.05204 | -0.01509 |
| F8-Pz | -1.03567 | 0.848763 | 0.980416 | -0.00612 | 65535 | 0.210202 | -0.18909 |
| F8-F9 | 0.54273 | 0.29417 | 0.980416 | -0.00217 | 65535 | 0.082317 | 0.099088 |
| F8-F10 | 0.883818 | 0.189296 | 0.980416 | -0.00213 | 65535 | 0.120093 | 0.161362 |
| T3-T4 | 0.48994 | 0.312543 | 0.980416 | -0.0022 | 65535 | 0.07381 | 0.08945 |
| T3-T5 | 0.346748 | 0.364699 | 0.980416 | -0.00277 | 65535 | 0.06228 | 0.063307 |
| T3-T6 | -0.3874 | 0.650422 | 0.980416 | -0.00406 | 65535 | 0.066229 | -0.07073 |
| T3-Fz | -0.67204 | 0.748564 | 0.980416 | -0.00822 | 65535 | 0.119845 | -0.1227 |
| T3-Cz | -0.72883 | 0.766224 | 0.980416 | -0.00699 | 65535 | 0.125116 | -0.13306 |
| T3-Pz | -2.1545 | 0.983383 | 0.988286 | -0.00743 | 65535 | 0.73266 | -0.39336 |
| T3-F9 | -1.5589 | 0.939151 | 0.980677 | -0.00763 | 65535 | 0.41699 | -0.28461 |
| T3-F10 | 0.871011 | 0.192758 | 0.980416 | -0.0018 | 65535 | 0.121643 | 0.159024 |
| T4-T5 | -0.85975 | 0.804166 | 0.980416 | -0.00502 | 65535 | 0.155125 | -0.15697 |
| T4-T6 | -0.61472 | 0.730038 | 0.980416 | -0.00501 | 65535 | 0.089714 | -0.11223 |
| T4-Fz | -1.17332 | 0.878486 | 0.980416 | -0.00978 | 65535 | 0.434688 | -0.21422 |
| T4-Cz | -0.9886 | 0.83756 | 0.980416 | -0.00676 | 65535 | 0.219471 | -0.18049 |
| T4-Pz | -2.26627 | 0.987371 | 0.988286 | -0.0078 | 65535 | 0.517635 | -0.41376 |
| T4-F9 | -1.1147 | 0.866379 | 0.980416 | -0.00464 | 65535 | 0.170225 | -0.20352 |
| T4-F10 | 0.809112 | 0.21004 | 0.980416 | -0.00177 | 65535 | 0.116482 | 0.147723 |
| T5-T6 | -0.06055 | 0.524092 | 0.980416 | -0.00354 | 65535 | 0.050367 | -0.01106 |
| T5-Fz | -0.06059 | 0.524107 | 0.980416 | -0.00431 | 65535 | 0.050512 | -0.01106 |
| T5-Cz | -0.91482 | 0.818925 | 0.980416 | -0.00598 | 65535 | 0.177832 | -0.16702 |
| T5-Pz | -1.41623 | 0.920328 | 0.980416 | -0.00745 | 65535 | 0.551911 | -0.25857 |
| T5-F9 | -0.73813 | 0.769048 | 0.980416 | -0.00503 | 65535 | 0.13868 | -0.13476 |
| T5-F10 | -0.09132 | 0.536302 | 0.980416 | -0.00447 | 65535 | 0.051207 | -0.01667 |
| T6-Fz | -0.71902 | 0.763225 | 0.980416 | -0.00711 | 65535 | 0.125871 | -0.13127 |
| T6-Cz | -1.39324 | 0.916917 | 0.980416 | -0.00801 | 65535 | 0.36589 | -0.25437 |
| T6-Pz | -0.45718 | 0.675808 | 0.980416 | -0.00544 | 65535 | 0.083578 | -0.08347 |
| T6-F9 | -1.04473 | 0.850859 | 0.980416 | -0.00525 | 65535 | 0.171759 | -0.19074 |
| T6-F10 | 0.117084 | 0.453496 | 0.980416 | -0.00313 | 65535 | 0.051175 | 0.021376 |
| Fz-Cz | -0.2764 | 0.608637 | 0.980416 | -0.01066 | 65535 | 0.068617 | -0.05046 |
| Fz-Pz | -0.76533 | 0.777198 | 0.980416 | -0.00843 | 65535 | 0.172153 | -0.13973 |
| Fz-F9 | -1.15113 | 0.873998 | 0.980416 | -0.00983 | 65535 | 0.289292 | -0.21017 |
| Fz-F10 | -0.44953 | 0.673063 | 0.980416 | -0.00786 | 65535 | 0.066928 | -0.08207 |
| Cz-Pz | -1.28233 | 0.89888 | 0.980416 | -0.01047 | 65535 | 0.451816 | -0.23412 |
| Cz-F9 | -1.61043 | 0.945012 | 0.980677 | -0.0101 | 65535 | 0.744613 | -0.29402 |
| Cz-F10 | -1.24157 | 0.891572 | 0.980416 | -0.00728 | 65535 | 0.329591 | -0.22668 |
| Pz-F9 | -2.29623 | 0.988286 | 0.988286 | -0.00768 | 65535 | 0.810308 | -0.41923 |
| Pz-F10 | -0.99242 | 0.838488 | 0.980416 | -0.00466 | 65535 | 0.227849 | -0.18119 |
| F9-F10 | -0.42512 | 0.664238 | 0.980416 | -0.00411 | 65535 | 0.07014 | -0.07762 |

Table S4j: gamma_wpli_PT_HC

| channel | tvalue | pvalue | fdr_pvalue | CI_low | CI_high | statistical power | effect size |
| --- | --- | --- | --- | --- | --- | --- | --- |
| Fp1-Fp2 | -0.43337 | 0.332768 | 0.678975 | 65535 | 0.003351 | 0.077448 | -0.07912 |
| Fp1-F3 | 0.309444 | 0.621235 | 0.759602 | 65535 | 0.010302 | 0.076418 | 0.056496 |
| Fp1-F4 | -0.1668 | 0.433908 | 0.689992 | 65535 | 0.008074 | 0.053611 | -0.03045 |
| Fp1-C3 | 0.316552 | 0.623928 | 0.759602 | 65535 | 0.008166 | 0.059716 | 0.057794 |
| Fp1-C4 | -1.13477 | 0.129385 | 0.678975 | 65535 | 0.001783 | 0.376197 | -0.20718 |
| Fp1-P3 | 0.38128 | 0.64816 | 0.763947 | 65535 | 0.009445 | 0.076901 | 0.069612 |
| Fp1-P4 | 0.307168 | 0.620372 | 0.759602 | 65535 | 0.005677 | 0.058071 | 0.056081 |
| Fp1-O1 | 0.556213 | 0.710441 | 0.789802 | 65535 | 0.005827 | 0.080986 | 0.10155 |
| Fp1-O2 | 0.579906 | 0.718458 | 0.789802 | 65535 | 0.005242 | 0.077229 | 0.105876 |
| Fp1-F7 | 0.25242 | 0.599422 | 0.758765 | 65535 | 0.008805 | 0.056095 | 0.046085 |
| Fp1-F8 | 0.570853 | 0.715408 | 0.789802 | 65535 | 0.011184 | 0.076419 | 0.104223 |
| Fp1-T3 | -0.52631 | 0.299829 | 0.678975 | 65535 | 0.003131 | 0.075264 | -0.09609 |
| Fp1-T4 | -0.01419 | 0.49435 | 0.706214 | 65535 | 0.004621 | 0.050022 | -0.00259 |
| Fp1-T5 | -0.05056 | 0.479879 | 0.699823 | 65535 | 0.004932 | 0.050325 | -0.00923 |
| Fp1-T6 | 1.118318 | 0.867149 | 0.888299 | 65535 | 0.009757 | 0.147481 | 0.204176 |
| Fp1-Fz | 0.637822 | 0.737587 | 0.798419 | 65535 | 0.011302 | 0.083805 | 0.11645 |
| Fp1-Cz | -0.78094 | 0.2182 | 0.678975 | 65535 | 0.004801 | 0.279819 | -0.14258 |
| Fp1-Pz | -1.88029 | 0.031267 | 0.678975 | 65535 | -0.00048 | 0.681508 | -0.34329 |
| Fp1-F9 | -0.61619 | 0.269477 | 0.678975 | 65535 | 0.002888 | 0.117794 | -0.1125 |
| Fp1-F10 | -0.77913 | 0.218732 | 0.678975 | 65535 | 0.002079 | 0.117052 | -0.14225 |
| Fp2-F3 | -0.65915 | 0.25554 | 0.678975 | 65535 | 0.005398 | 0.219586 | -0.12034 |
| Fp2-F4 | -0.34865 | 0.363986 | 0.684885 | 65535 | 0.006935 | 0.067795 | -0.06365 |
| Fp2-C3 | -1.40251 | 0.081694 | 0.678975 | 65535 | 0.000982 | 0.523518 | -0.25606 |
| Fp2-C4 | -1.34663 | 0.09034 | 0.678975 | 65535 | 0.001235 | 0.731518 | -0.24586 |
| Fp2-P3 | -0.16049 | 0.436386 | 0.689992 | 65535 | 0.006675 | 0.054679 | -0.0293 |
| Fp2-P4 | -1.41838 | 0.079358 | 0.678975 | 65535 | 0.000784 | 0.359031 | -0.25896 |
| Fp2-O1 | 0.427931 | 0.665259 | 0.767607 | 65535 | 0.005329 | 0.069883 | 0.078129 |
| Fp2-O2 | -0.42408 | 0.33614 | 0.678975 | 65535 | 0.003586 | 0.073285 | -0.07743 |
| Fp2-F7 | -0.63685 | 0.262727 | 0.678975 | 65535 | 0.003906 | 0.092259 | -0.11627 |
| Fp2-F8 | 0.170194 | 0.567425 | 0.74092 | 65535 | 0.009585 | 0.052371 | 0.031073 |
| Fp2-T3 | -1.27835 | 0.101817 | 0.678975 | 65535 | 0.000915 | 0.233582 | -0.23339 |
| Fp2-T4 | -0.99603 | 0.160637 | 0.678975 | 65535 | 0.001946 | 0.260105 | -0.18185 |
| Fp2-T5 | -0.22886 | 0.409688 | 0.686333 | 65535 | 0.004573 | 0.057447 | -0.04178 |
| Fp2-T6 | 0.705893 | 0.759177 | 0.813404 | 65535 | 0.008909 | 0.097303 | 0.128878 |
| Fp2-Fz | -0.38262 | 0.351346 | 0.678975 | 65535 | 0.007467 | 0.069159 | -0.06986 |
| Fp2-Cz | -1.28383 | 0.100859 | 0.678975 | 65535 | 0.002467 | 0.888091 | -0.23439 |
| Fp2-Pz | -1.92667 | 0.028213 | 0.678975 | 65535 | -0.00067 | 0.758831 | -0.35176 |
| Fp2-F9 | -1.35968 | 0.088262 | 0.678975 | 65535 | 0.000815 | 0.385607 | -0.24824 |
| Fp2-F10 | -1.22621 | 0.111281 | 0.678975 | 65535 | 0.001074 | 0.266232 | -0.22387 |
| F3-F4 | -0.57507 | 0.28317 | 0.678975 | 65535 | 0.006125 | 0.115297 | -0.10499 |
| F3-C3 | 0.044869 | 0.517856 | 0.722684 | 65535 | 0.007939 | 0.050328 | 0.008192 |
| F3-C4 | -0.22699 | 0.410414 | 0.686333 | 65535 | 0.004457 | 0.060693 | -0.04144 |
| F3-P3 | 0.362817 | 0.641305 | 0.76087 | 65535 | 0.00893 | 0.071921 | 0.066241 |
| F3-P4 | -0.82057 | 0.206774 | 0.678975 | 65535 | 0.002177 | 0.216212 | -0.14981 |
| F3-O1 | 0.809905 | 0.790188 | 0.829697 | 65535 | 0.006111 | 0.119997 | 0.147868 |
| F3-O2 | -1.11522 | 0.133512 | 0.678975 | 65535 | 0.001162 | 0.226636 | -0.20361 |
| F3-F7 | 0.775832 | 0.7803 | 0.829697 | 65535 | 0.009469 | 0.120788 | 0.141647 |
| F3-F8 | 0.213167 | 0.584218 | 0.752673 | 65535 | 0.008748 | 0.05846 | 0.038919 |
| F3-T3 | 0.003821 | 0.501521 | 0.711618 | 65535 | 0.006247 | 0.050003 | 0.000698 |
| F3-T4 | -0.12248 | 0.451363 | 0.69187 | 65535 | 0.003745 | 0.051769 | -0.02236 |
| F3-T5 | 0.235236 | 0.592783 | 0.758765 | 65535 | 0.00643 | 0.055386 | 0.042948 |
| F3-T6 | -0.4654 | 0.321253 | 0.678975 | 65535 | 0.003245 | 0.084347 | -0.08497 |
| F3-Fz | -0.37971 | 0.35242 | 0.678975 | 65535 | 0.00727 | 0.083133 | -0.06933 |
| F3-Cz | -0.3198 | 0.374841 | 0.684885 | 65535 | 0.006731 | 0.078816 | -0.05839 |
| F3-Pz | -1.60049 | 0.056082 | 0.678975 | 65535 | 0.000172 | 0.756426 | -0.29221 |
| F3-F9 | 0.794094 | 0.785633 | 0.829697 | 65535 | 0.007237 | 0.11862 | 0.144981 |
| F3-F10 | -0.15871 | 0.437085 | 0.689992 | 65535 | 0.00414 | 0.054658 | -0.02898 |
| F4-C3 | -0.92507 | 0.178409 | 0.678975 | 65535 | 0.003206 | 0.407701 | -0.16889 |
| F4-C4 | -1.3263 | 0.093652 | 0.678975 | 65535 | 0.0015 | 0.532073 | -0.24215 |
| F4-P3 | 0.409247 | 0.65845 | 0.763947 | 65535 | 0.007908 | 0.067315 | 0.074718 |
| F4-P4 | -0.7284 | 0.233907 | 0.678975 | 65535 | 0.003795 | 0.136004 | -0.13299 |
| F4-O1 | 0.350267 | 0.636619 | 0.759602 | 65535 | 0.005338 | 0.063991 | 0.06395 |
| F4-O2 | -0.76996 | 0.22143 | 0.678975 | 65535 | 0.002326 | 0.131564 | -0.14058 |
| F4-F7 | -0.15058 | 0.440281 | 0.689992 | 65535 | 0.005663 | 0.052344 | -0.02749 |
| F4-F8 | -0.22463 | 0.411328 | 0.686333 | 65535 | 0.006164 | 0.056841 | -0.04101 |
| F4-T3 | -0.608 | 0.27218 | 0.678975 | 65535 | 0.003047 | 0.099754 | -0.111 |
| F4-T4 | -0.39283 | 0.347577 | 0.678975 | 65535 | 0.004686 | 0.077162 | -0.07172 |
| F4-T5 | 0.294128 | 0.615412 | 0.759602 | 65535 | 0.00504 | 0.061034 | 0.0537 |
| F4-T6 | 0.402225 | 0.655877 | 0.763947 | 65535 | 0.007995 | 0.064093 | 0.073436 |
| F4-Fz | -0.41734 | 0.338593 | 0.678975 | 65535 | 0.007861 | 0.076799 | -0.0762 |
| F4-Cz | -0.27817 | 0.390684 | 0.684885 | 65535 | 0.008926 | 0.065657 | -0.05079 |
| F4-Pz | -0.62157 | 0.267711 | 0.678975 | 65535 | 0.003218 | 0.114259 | -0.11348 |
| F4-F9 | -0.23338 | 0.407937 | 0.686333 | 65535 | 0.004472 | 0.058577 | -0.04261 |
| F4-F10 | -0.4479 | 0.327522 | 0.678975 | 65535 | 0.003564 | 0.069699 | -0.08178 |
| C3-C4 | -0.95782 | 0.170055 | 0.678975 | 65535 | 0.002043 | 0.32227 | -0.17487 |
| C3-P3 | -0.45972 | 0.32328 | 0.678975 | 65535 | 0.006895 | 0.08562 | -0.08393 |
| C3-P4 | -1.33955 | 0.091483 | 0.678975 | 65535 | 0.000814 | 0.381986 | -0.24457 |
| C3-O1 | -0.15349 | 0.439136 | 0.689992 | 65535 | 0.004112 | 0.052503 | -0.02802 |
| C3-O2 | -0.73358 | 0.232329 | 0.678975 | 65535 | 0.001965 | 0.131186 | -0.13393 |
| C3-F7 | -0.215 | 0.415068 | 0.686333 | 65535 | 0.005482 | 0.05724 | -0.03925 |
| C3-F8 | -0.22598 | 0.410803 | 0.686333 | 65535 | 0.00423 | 0.058347 | -0.04126 |
| C3-T3 | -0.52748 | 0.299426 | 0.678975 | 65535 | 0.005358 | 0.073375 | -0.0963 |
| C3-T4 | -0.70981 | 0.239611 | 0.678975 | 65535 | 0.00183 | 0.121452 | -0.12959 |
| C3-T5 | -1.51539 | 0.066174 | 0.678975 | 65535 | 0.000457 | 0.474478 | -0.27667 |
| C3-T6 | -0.39799 | 0.345679 | 0.678975 | 65535 | 0.002851 | 0.068721 | -0.07266 |
| C3-Fz | -1.03433 | 0.151548 | 0.678975 | 65535 | 0.003229 | 0.50371 | -0.18884 |
| C3-Cz | -0.2813 | 0.389486 | 0.684885 | 65535 | 0.005277 | 0.067037 | -0.05136 |
| C3-Pz | -1.98835 | 0.024544 | 0.678975 | 65535 | -0.00114 | 0.935347 | -0.36302 |
| C3-F9 | -0.79158 | 0.215096 | 0.678975 | 65535 | 0.00268 | 0.121858 | -0.14452 |
| C3-F10 | -0.42564 | 0.335572 | 0.678975 | 65535 | 0.002961 | 0.074922 | -0.07771 |
| C4-P3 | -0.32525 | 0.372785 | 0.684885 | 65535 | 0.004174 | 0.08259 | -0.05938 |
| C4-P4 | -1.00717 | 0.157957 | 0.678975 | 65535 | 0.001858 | 0.28084 | -0.18388 |
| C4-O1 | -1.13107 | 0.13016 | 0.678975 | 65535 | 0.00097 | 0.211366 | -0.2065 |
| C4-O2 | -0.35652 | 0.361045 | 0.684885 | 65535 | 0.002457 | 0.063345 | -0.06509 |
| C4-F7 | 0.067692 | 0.526927 | 0.722684 | 65535 | 0.004404 | 0.050695 | 0.012359 |
| C4-F8 | -0.62724 | 0.265858 | 0.678975 | 65535 | 0.003374 | 0.134755 | -0.11452 |
| C4-T3 | -1.22224 | 0.112025 | 0.678975 | 65535 | 0.000942 | 0.336998 | -0.22315 |
| C4-T4 | -1.63829 | 0.052012 | 0.678975 | 65535 | 0.000101 | 0.777766 | -0.29911 |
| C4-T5 | -0.51845 | 0.302556 | 0.678975 | 65535 | 0.002213 | 0.100206 | -0.09466 |
| C4-T6 | -0.52844 | 0.299093 | 0.678975 | 65535 | 0.002837 | 0.094716 | -0.09648 |
| C4-Fz | -0.91724 | 0.180444 | 0.678975 | 65535 | 0.003281 | 0.384798 | -0.16746 |
| C4-Cz | -0.28791 | 0.386961 | 0.684885 | 65535 | 0.005184 | 0.071315 | -0.05256 |
| C4-Pz | -1.30523 | 0.097176 | 0.678975 | 65535 | 0.000964 | 0.421204 | -0.2383 |
| C4-F9 | -0.03285 | 0.486925 | 0.703192 | 65535 | 0.003638 | 0.050232 | -0.006 |
| C4-F10 | -0.58129 | 0.281077 | 0.678975 | 65535 | 0.002076 | 0.100741 | -0.10613 |
| P3-P4 | -0.13066 | 0.448134 | 0.69187 | 65535 | 0.004406 | 0.052479 | -0.02385 |
| P3-O1 | 0.082038 | 0.532622 | 0.722684 | 65535 | 0.0059 | 0.050813 | 0.014978 |
| P3-O2 | -0.95311 | 0.171242 | 0.678975 | 65535 | 0.00216 | 0.188385 | -0.17401 |
| P3-F7 | 1.277935 | 0.89811 | 0.911014 | 65535 | 0.010458 | 0.210683 | 0.233318 |
| P3-F8 | 0.938746 | 0.825111 | 0.853563 | 65535 | 0.009419 | 0.127523 | 0.171391 |
| P3-T3 | 1.084148 | 0.859745 | 0.885032 | 65535 | 0.006727 | 0.174061 | 0.197937 |
| P3-T4 | 0.587734 | 0.721083 | 0.789802 | 65535 | 0.003539 | 0.084413 | 0.107305 |
| P3-T5 | -0.1395 | 0.444646 | 0.691671 | 65535 | 0.006163 | 0.053989 | -0.02547 |
| P3-T6 | 0.084023 | 0.53341 | 0.722684 | 65535 | 0.003801 | 0.050869 | 0.01534 |
| P3-Fz | -0.30152 | 0.381774 | 0.684885 | 65535 | 0.004537 | 0.063385 | -0.05505 |
| P3-Cz | -0.09907 | 0.460626 | 0.695983 | 65535 | 0.006745 | 0.052674 | -0.01809 |
| P3-Pz | -1.13316 | 0.129723 | 0.678975 | 65535 | 0.00228 | 0.266779 | -0.20688 |
| P3-F9 | 0.538433 | 0.704354 | 0.789802 | 65535 | 0.004362 | 0.076685 | 0.098304 |
| P3-F10 | 0.018766 | 0.50747 | 0.715227 | 65535 | 0.003772 | 0.050032 | 0.003426 |
| P4-O1 | -0.55834 | 0.288835 | 0.678975 | 65535 | 0.002417 | 0.089164 | -0.10194 |
| P4-O2 | -0.76864 | 0.221821 | 0.678975 | 65535 | 0.002823 | 0.128214 | -0.14033 |
| P4-F7 | 0.296719 | 0.616399 | 0.759602 | 65535 | 0.005042 | 0.05741 | 0.054173 |
| P4-F8 | -0.08223 | 0.467303 | 0.695983 | 65535 | 0.005069 | 0.050635 | -0.01501 |
| P4-T3 | -0.33975 | 0.367326 | 0.684885 | 65535 | 0.002352 | 0.069587 | -0.06203 |
| P4-T4 | -0.84572 | 0.199709 | 0.678975 | 65535 | 0.001483 | 0.144104 | -0.15441 |
| P4-T5 | -0.89886 | 0.185279 | 0.678975 | 65535 | 0.001345 | 0.170132 | -0.16411 |
| P4-T6 | 0.496931 | 0.689919 | 0.784682 | 65535 | 0.007554 | 0.083797 | 0.090727 |
| P4-Fz | -1.84471 | 0.033794 | 0.678975 | 65535 | -0.00057 | 0.827445 | -0.3368 |
| P4-Cz | -1.36503 | 0.08742 | 0.678975 | 65535 | 0.00093 | 0.48745 | -0.24922 |
| P4-Pz | -1.27198 | 0.102941 | 0.678975 | 65535 | 0.001621 | 0.579832 | -0.23223 |
| P4-F9 | -0.94622 | 0.172986 | 0.678975 | 65535 | 0.00158 | 0.183061 | -0.17275 |
| P4-F10 | 0.079438 | 0.531591 | 0.722684 | 65535 | 0.003477 | 0.05099 | 0.014503 |
| O1-O2 | -0.6462 | 0.259702 | 0.678975 | 65535 | 0.003203 | 0.117375 | -0.11798 |
| O1-F7 | 0.095172 | 0.53783 | 0.724002 | 65535 | 0.003717 | 0.050925 | 0.017376 |
| O1-F8 | 0.253364 | 0.599786 | 0.758765 | 65535 | 0.003973 | 0.057291 | 0.046258 |
| O1-T3 | 0.329066 | 0.628656 | 0.759602 | 65535 | 0.004321 | 0.061196 | 0.060079 |
| O1-T4 | 0.206799 | 0.581738 | 0.752673 | 65535 | 0.003145 | 0.054375 | 0.037756 |
| O1-T5 | 1.544138 | 0.937384 | 0.937384 | 65535 | 0.00967 | 0.369994 | 0.28192 |
| O1-T6 | 1.302377 | 0.902338 | 0.911014 | 65535 | 0.005266 | 0.251751 | 0.23778 |
| O1-Fz | -0.95286 | 0.171304 | 0.678975 | 65535 | 0.001806 | 0.167752 | -0.17397 |
| O1-Cz | -0.63104 | 0.264619 | 0.678975 | 65535 | 0.002339 | 0.105088 | -0.11521 |
| O1-Pz | -0.9321 | 0.176593 | 0.678975 | 65535 | 0.002745 | 0.381469 | -0.17018 |
| O1-F9 | -0.28877 | 0.386634 | 0.684885 | 65535 | 0.002699 | 0.059134 | -0.05272 |
| O1-F10 | 0.315829 | 0.623655 | 0.759602 | 65535 | 0.003325 | 0.058468 | 0.057662 |
| O2-F7 | -0.15221 | 0.43964 | 0.689992 | 65535 | 0.002753 | 0.052049 | -0.02779 |
| O2-F8 | -0.15652 | 0.437947 | 0.689992 | 65535 | 0.003221 | 0.052534 | -0.02858 |
| O2-T3 | -0.92143 | 0.179354 | 0.678975 | 65535 | 0.001316 | 0.149501 | -0.16823 |
| O2-T4 | 0.080339 | 0.531948 | 0.722684 | 65535 | 0.003266 | 0.050671 | 0.014668 |
| O2-T5 | 0.132379 | 0.552545 | 0.734395 | 65535 | 0.003242 | 0.051879 | 0.024169 |
| O2-T6 | -0.524 | 0.300632 | 0.678975 | 65535 | 0.00379 | 0.104036 | -0.09567 |
| O2-Fz | -0.44265 | 0.329413 | 0.678975 | 65535 | 0.002964 | 0.069985 | -0.08082 |
| O2-Cz | -0.3887 | 0.349098 | 0.678975 | 65535 | 0.003718 | 0.06081 | -0.07097 |
| O2-Pz | -1.08767 | 0.139479 | 0.678975 | 65535 | 0.001494 | 0.185896 | -0.19858 |
| O2-F9 | -0.22164 | 0.412489 | 0.686333 | 65535 | 0.002697 | 0.055815 | -0.04047 |
| O2-F10 | -0.94406 | 0.173535 | 0.678975 | 65535 | 0.001416 | 0.133734 | -0.17236 |
| F7-F8 | 0.341474 | 0.633323 | 0.759602 | 65535 | 0.005469 | 0.06379 | 0.062344 |
| F7-T3 | -1.10785 | 0.13509 | 0.678975 | 65535 | 0.001694 | 0.208341 | -0.20226 |
| F7-T4 | 0.701781 | 0.757901 | 0.813404 | 65535 | 0.00501 | 0.088173 | 0.128127 |
| F7-T5 | 0.15481 | 0.561382 | 0.74092 | 65535 | 0.004398 | 0.052591 | 0.028264 |
| F7-T6 | 0.591883 | 0.72247 | 0.789802 | 65535 | 0.006908 | 0.073769 | 0.108063 |
| F7-Fz | 0.602091 | 0.725866 | 0.789802 | 65535 | 0.012155 | 0.079919 | 0.109926 |
| F7-Cz | -0.46049 | 0.323006 | 0.678975 | 65535 | 0.004784 | 0.083181 | -0.08407 |
| F7-Pz | -1.44462 | 0.075608 | 0.678975 | 65535 | 0.000433 | 0.322237 | -0.26375 |
| F7-F9 | -0.56134 | 0.287816 | 0.678975 | 65535 | 0.002614 | 0.087921 | -0.10249 |
| F7-F10 | 0.500771 | 0.691268 | 0.784682 | 65535 | 0.005171 | 0.070918 | 0.091428 |
| F8-T3 | -0.02792 | 0.488886 | 0.703192 | 65535 | 0.00455 | 0.050076 | -0.0051 |
| F8-T4 | 1.226814 | 0.888833 | 0.906092 | 65535 | 0.007633 | 0.20908 | 0.223985 |
| F8-T5 | 0.171758 | 0.568039 | 0.74092 | 65535 | 0.004492 | 0.054078 | 0.031358 |
| F8-T6 | 1.508163 | 0.932907 | 0.937371 | 65535 | 0.010879 | 0.235119 | 0.275352 |
| F8-Fz | 0.39635 | 0.653719 | 0.763947 | 65535 | 0.011308 | 0.0683 | 0.072363 |
| F8-Cz | -0.08266 | 0.467131 | 0.695983 | 65535 | 0.007522 | 0.05204 | -0.01509 |
| F8-Pz | -1.03567 | 0.151237 | 0.678975 | 65535 | 0.001413 | 0.210202 | -0.18909 |
| F8-F9 | 0.54273 | 0.70583 | 0.789802 | 65535 | 0.004273 | 0.082317 | 0.099088 |
| F8-F10 | 0.883818 | 0.810704 | 0.842811 | 65535 | 0.006979 | 0.120093 | 0.161362 |
| T3-T4 | 0.48994 | 0.687457 | 0.784682 | 65535 | 0.00405 | 0.07381 | 0.08945 |
| T3-T5 | 0.346748 | 0.635301 | 0.759602 | 65535 | 0.004239 | 0.06228 | 0.063307 |
| T3-T6 | -0.3874 | 0.349578 | 0.678975 | 65535 | 0.002519 | 0.066229 | -0.07073 |
| T3-Fz | -0.67204 | 0.251436 | 0.678975 | 65535 | 0.003478 | 0.119845 | -0.1227 |
| T3-Cz | -0.72883 | 0.233776 | 0.678975 | 65535 | 0.002723 | 0.125116 | -0.13306 |
| T3-Pz | -2.1545 | 0.016617 | 0.678975 | 65535 | -0.00097 | 0.73266 | -0.39336 |
| T3-F9 | -1.5589 | 0.060849 | 0.678975 | 65535 | 0.000235 | 0.41699 | -0.28461 |
| T3-F10 | 0.871011 | 0.807242 | 0.842811 | 65535 | 0.005798 | 0.121643 | 0.159024 |
| T4-T5 | -0.85975 | 0.195834 | 0.678975 | 65535 | 0.001592 | 0.155125 | -0.15697 |
| T4-T6 | -0.61472 | 0.269962 | 0.678975 | 65535 | 0.002297 | 0.089714 | -0.11223 |
| T4-Fz | -1.17332 | 0.121514 | 0.678975 | 65535 | 0.001674 | 0.434688 | -0.21422 |
| T4-Cz | -0.9886 | 0.16244 | 0.678975 | 65535 | 0.00171 | 0.219471 | -0.18049 |
| T4-Pz | -2.26627 | 0.012629 | 0.678975 | 65535 | -0.00121 | 0.517635 | -0.41376 |
| T4-F9 | -1.1147 | 0.133621 | 0.678975 | 65535 | 0.00091 | 0.170225 | -0.20352 |
| T4-F10 | 0.809112 | 0.78996 | 0.829697 | 65535 | 0.005148 | 0.116482 | 0.147723 |
| T5-T6 | -0.06055 | 0.475908 | 0.698886 | 65535 | 0.003288 | 0.050367 | -0.01106 |
| T5-Fz | -0.06059 | 0.475893 | 0.698886 | 65535 | 0.004008 | 0.050512 | -0.01106 |
| T5-Cz | -0.91482 | 0.181075 | 0.678975 | 65535 | 0.001727 | 0.177832 | -0.16702 |
| T5-Pz | -1.41623 | 0.079672 | 0.678975 | 65535 | 0.000585 | 0.551911 | -0.25857 |
| T5-F9 | -0.73813 | 0.230952 | 0.678975 | 65535 | 0.001931 | 0.13868 | -0.13476 |
| T5-F10 | -0.09132 | 0.463698 | 0.695983 | 65535 | 0.004004 | 0.051207 | -0.01667 |
| T6-Fz | -0.71902 | 0.236775 | 0.678975 | 65535 | 0.002809 | 0.125871 | -0.13127 |
| T6-Cz | -1.39324 | 0.083083 | 0.678975 | 65535 | 0.000695 | 0.36589 | -0.25437 |
| T6-Pz | -0.45718 | 0.324192 | 0.678975 | 65535 | 0.00309 | 0.083578 | -0.08347 |
| T6-F9 | -1.04473 | 0.149141 | 0.678975 | 65535 | 0.001192 | 0.171759 | -0.19074 |
| T6-F10 | 0.117084 | 0.546504 | 0.730992 | 65535 | 0.00361 | 0.051175 | 0.021376 |
| Fz-Cz | -0.2764 | 0.391363 | 0.684885 | 65535 | 0.007615 | 0.068617 | -0.05046 |
| Fz-Pz | -0.76533 | 0.222802 | 0.678975 | 65535 | 0.003106 | 0.172153 | -0.13973 |
| Fz-F9 | -1.15113 | 0.126002 | 0.678975 | 65535 | 0.001773 | 0.289292 | -0.21017 |
| Fz-F10 | -0.44953 | 0.326937 | 0.678975 | 65535 | 0.004505 | 0.066928 | -0.08207 |
| Cz-Pz | -1.28233 | 0.10112 | 0.678975 | 65535 | 0.001337 | 0.451816 | -0.23412 |
| Cz-F9 | -1.61043 | 0.054988 | 0.678975 | 65535 | 0.000147 | 0.744613 | -0.29402 |
| Cz-F10 | -1.24157 | 0.108428 | 0.678975 | 65535 | 0.001046 | 0.329591 | -0.22668 |
| Pz-F9 | -2.29623 | 0.011714 | 0.678975 | 65535 | -0.00124 | 0.810308 | -0.41923 |
| Pz-F10 | -0.99242 | 0.161512 | 0.678975 | 65535 | 0.001169 | 0.227849 | -0.18119 |
| F9-F10 | -0.42512 | 0.335762 | 0.678975 | 65535 | 0.002432 | 0.07014 | -0.07762 |

Table S5. Cross-frequency coupling (PAC)

Table S5a: theta_beta PAC

| channel | tvalue | pvalue | fdr_pvalue | CI_low | CI_high | statistical power | effect size |
| --- | --- | --- | --- | --- | --- | --- | --- |
| Fp1 | 3.558227 | 0.000539 | 0.011315 | 1.278801 | 4.488491 | 1 | -0.64964 |
| Fp2 | 1.614477 | 0.109095 | 0.254555 | -0.6432 | 6.320819 | 0.631825 | -0.29476 |
| F3 | 0.849356 | 0.397403 | 0.556738 | -0.31533 | 0.788989 | 0.122702 | -0.15507 |
| F4 | 1.663376 | 0.098891 | 0.254555 | -0.16613 | 1.910152 | 0.873878 | -0.30369 |
| C3 | -0.32376 | 0.746691 | 0.82529 | -1.97336 | 1.418764 | 0.060887 | 0.059111 |
| C4 | 0.060806 | 0.951617 | 0.951617 | -2.10771 | 2.241252 | 0.050322 | -0.0111 |
| P3 | 0.477829 | 0.633656 | 0.771516 | -2.33987 | 3.828187 | 0.081944 | -0.08724 |
| P4 | 0.848875 | 0.39767 | 0.556738 | -1.62144 | 4.054542 | 0.19413 | -0.15498 |
| O1 | 1.145403 | 0.25436 | 0.44513 | -0.9795 | 3.667148 | 0.394551 | -0.20912 |
| O2 | 0.157814 | 0.874873 | 0.918617 | -1.75896 | 2.063596 | 0.053593 | -0.02881 |
| F7 | 0.439228 | 0.6613 | 0.771516 | -2.03896 | 3.201249 | 0.067636 | -0.08019 |
| F8 | 1.989109 | 0.049003 | 0.205811 | 0.00996 | 4.473753 | 0.983128 | -0.36316 |
| T3 | 1.066151 | 0.288532 | 0.46609 | -3.05266 | 10.17336 | 0.466502 | -0.19465 |
| T4 | 1.78603 | 0.076663 | 0.254555 | -0.97487 | 18.9025 | 0.875502 | -0.32608 |
| T5 | 2.177019 | 0.03147 | 0.165219 | 0.79099 | 16.71371 | 0.996654 | -0.39747 |
| T6 | 1.632807 | 0.105175 | 0.254555 | -0.67694 | 7.039121 | 0.656518 | -0.29811 |
| Fz | 2.405147 | 0.01772 | 0.132351 | 0.22515 | 2.323927 | 1 | -0.43912 |
| Cz | 0.510416 | 0.610713 | 0.771516 | -0.97207 | 1.647183 | 0.073248 | -0.09319 |
| Pz | 1.494441 | 0.13773 | 0.289232 | -0.91101 | 6.515654 | 0.969355 | -0.27285 |
| F9 | 2.38016 | 0.018907 | 0.132351 | 4.431379 | 48.32037 | 0.999444 | -0.43456 |
| F10 | 1.362569 | 0.175613 | 0.335262 | -3.81139 | 20.62617 | 0.649057 | -0.24877 |

Table S5b: theta_gamma PAC

| channel | tvalue | pvalue | fdr_pvalue | CI_low | CI_high | statistical power | effect size |
| --- | --- | --- | --- | --- | --- | --- | --- |
| Fp1 | 3.61983 | 0.000436 | 0.009146 | 1.504174 | 5.137683 | 1 | -0.66089 |
| Fp2 | 1.907436 | 0.058895 | 0.176685 | -0.16922 | 9.032312 | 1 | -0.34825 |
| F3 | -0.63314 | 0.527867 | 0.583432 | -1.32821 | 0.68465 | 0.075563 | 0.115595 |
| F4 | 1.390732 | 0.166924 | 0.274274 | -0.33484 | 1.914635 | 0.783834 | -0.25391 |
| C3 | -0.19116 | 0.848729 | 0.863693 | -0.68385 | 0.563443 | 0.053403 | 0.034901 |
| C4 | -0.92522 | 0.356738 | 0.416194 | -1.75675 | 0.637915 | 0.11054 | 0.168922 |
| P3 | 0.17205 | 0.863693 | 0.863693 | -1.48996 | 1.773494 | 0.05277 | -0.03141 |
| P4 | -1.0812 | 0.281815 | 0.348124 | -1.42417 | 0.418243 | 0.161698 | 0.197398 |
| O1 | 1.953465 | 0.053131 | 0.176685 | -0.01969 | 2.889308 | 0.703091 | -0.35665 |
| O2 | 1.250282 | 0.21367 | 0.289667 | -0.4271 | 1.890134 | 0.506073 | -0.22827 |
| F7 | 1.62703 | 0.106398 | 0.223436 | -0.27311 | 2.789004 | 0.714474 | -0.29705 |
| F8 | 1.231194 | 0.220698 | 0.289667 | -0.68503 | 2.936874 | 0.559465 | -0.22478 |
| T3 | 1.987107 | 0.049227 | 0.176685 | 0.009567 | 5.553878 | 1 | -0.36279 |
| T4 | 1.520108 | 0.131159 | 0.250394 | -0.91737 | 6.97825 | 0.420694 | -0.27753 |
| T5 | 2.138007 | 0.034579 | 0.176685 | 0.406287 | 10.60767 | 1 | -0.39034 |
| T6 | 1.688614 | 0.093935 | 0.223436 | -0.16243 | 2.043217 | 0.599478 | -0.3083 |
| Fz | 1.654701 | 0.100643 | 0.223436 | -0.22144 | 2.472305 | 0.992316 | -0.30211 |
| Cz | 1.33991 | 0.182849 | 0.274274 | -0.63066 | 3.269901 | 0.756986 | -0.24463 |
| Pz | 1.344085 | 0.181499 | 0.274274 | -2.34162 | 12.236 | 1 | -0.2454 |
| F9 | 2.129918 | 0.035256 | 0.176685 | 0.765354 | 21.02127 | 0.993201 | -0.38887 |
| F10 | 1.961599 | 0.052164 | 0.176685 | -0.04564 | 9.634966 | 0.999921 | -0.35814 |

Table S5c: beta_gamma PAC

| channel | tvalue | pvalue | fdr_pvalue | CI_low | CI_high | statistical power | effect size |
| --- | --- | --- | --- | --- | --- | --- | --- |
| Fp1 | -0.49978 | 0.618164 | 0.89517 | -2.55644 | 1.526102 | 0.073331 | 0.091246 |
| Fp2 | -1.74798 | 0.083069 | 0.290741 | -4.81649 | 0.300099 | 0.242087 | 0.319136 |
| F3 | -0.02957 | 0.976459 | 0.976459 | -7.02934 | 6.82249 | 0.050067 | 0.005399 |
| F4 | 1.377604 | 0.170933 | 0.41612 | -3.97948 | 22.17236 | 0.562143 | -0.25151 |
| C3 | 0.22995 | 0.818529 | 0.90469 | -11.9858 | 15.1351 | 0.059454 | -0.04198 |
| C4 | 0.80902 | 0.420132 | 0.73523 | -4.27587 | 10.18283 | 0.148449 | -0.14771 |
| P3 | -1.66792 | 0.097982 | 0.293947 | -26.0858 | 2.23339 | 0.237799 | 0.30452 |
| P4 | -2.27342 | 0.024809 | 0.173666 | -26.4727 | -1.82439 | 0.38915 | 0.415068 |
| O1 | -2.10516 | 0.037398 | 0.196342 | -15.6088 | -0.47716 | 0.361127 | 0.384348 |
| O2 | -3.53522 | 0.000583 | 0.012243 | -10.9102 | -3.07583 | 0.731676 | 0.645439 |
| F7 | 0.982602 | 0.327814 | 0.625826 | -1.2012 | 3.567315 | 0.309478 | -0.1794 |
| F8 | -0.27515 | 0.783682 | 0.90469 | -5.73647 | 4.336831 | 0.054803 | 0.050235 |
| T3 | 0.542313 | 0.588626 | 0.89517 | -34.2634 | 60.10758 | 0.104377 | -0.09901 |
| T4 | 2.530723 | 0.012699 | 0.133341 | 6.423692 | 52.64277 | 0.999901 | -0.46204 |
| T5 | 0.410704 | 0.682035 | 0.89517 | -5.4914 | 8.365234 | 0.076881 | -0.07498 |
| T6 | -0.44149 | 0.659663 | 0.89517 | -6.68367 | 4.246769 | 0.091174 | 0.080605 |
| Fz | -0.2576 | 0.797161 | 0.90469 | -1.91232 | 1.472064 | 0.057287 | 0.047032 |
| Cz | 0.066407 | 0.947166 | 0.976459 | -6.7111 | 7.176822 | 0.050373 | -0.01212 |
| Pz | -1.24791 | 0.214536 | 0.450525 | -5.00049 | 1.134444 | 0.169612 | 0.227836 |
| F9 | 1.767058 | 0.079804 | 0.290741 | -0.6105 | 10.72983 | 1 | -0.32262 |
| F10 | 1.353958 | 0.178337 | 0.41612 | -0.51261 | 2.728894 | 0.365 | -0.2472 |

Table S6. Sample entropy (EnSA)

| channel | tvalue | pvalue | fdr_pvalue | CI_low | CI_high | statistical power | effect size |
| --- | --- | --- | --- | --- | --- | --- | --- |
| Fp1 | -3.49966 | 0.000658 | 0.000921 | -0.18952 | -0.05255 | 0.994012 | -0.63895 |
| Fp2 | -3.26699 | 0.001424 | 0.001573 | -0.18397 | -0.04511 | 0.985147 | -0.59647 |
| F3 | -4.11872 | 7.1E-05 | 0.000213 | -0.1539 | -0.05396 | 0.999965 | -0.75197 |
| F4 | -3.63601 | 0.000412 | 0.000665 | -0.15237 | -0.04492 | 0.997268 | -0.66384 |
| C3 | -4.18904 | 5.43E-05 | 0.000213 | -0.08659 | -0.031 | 0.99865 | -0.76481 |
| C4 | -5.2016 | 8.42E-07 | 1.72E-05 | -0.07704 | -0.03456 | 1 | -0.94968 |
| P3 | -3.29454 | 0.001302 | 0.001519 | -0.16593 | -0.04134 | 0.984426 | -0.6015 |
| P4 | -4.643 | 8.98E-06 | 4.71E-05 | -0.1777 | -0.07144 | 0.999998 | -0.84769 |
| O1 | -3.73292 | 0.000293 | 0.000512 | -0.1783 | -0.0547 | 0.997272 | -0.68154 |
| O2 | -4.12363 | 6.97E-05 | 0.000213 | -0.1853 | -0.06507 | 0.999755 | -0.75287 |
| F7 | -3.85504 | 0.000189 | 0.000417 | -0.16575 | -0.05325 | 0.99904 | -0.70383 |
| F8 | -3.38799 | 0.000958 | 0.001245 | -0.16532 | -0.04335 | 0.985979 | -0.61856 |
| T3 | -4.65862 | 8.42E-06 | 4.71E-05 | -0.0958 | -0.03865 | 0.999788 | -0.85054 |
| T4 | -3.07534 | 0.002613 | 0.002681 | -0.06754 | -0.01463 | 0.894815 | -0.56148 |
| T5 | -3.81479 | 0.000218 | 0.000417 | -0.13283 | -0.04205 | 0.997709 | -0.69648 |
| T6 | -5.04813 | 1.64E-06 | 1.72E-05 | -0.14959 | -0.06529 | 1 | -0.92166 |
| Fz | -3.90093 | 0.00016 | 0.000417 | -0.13479 | -0.04402 | 0.99817 | -0.71221 |
| Cz | -3.828 | 0.000208 | 0.000417 | -0.0809 | -0.02574 | 0.994267 | -0.69889 |
| Pz | -3.37262 | 0.001008 | 0.001245 | -0.1747 | -0.04544 | 0.986286 | -0.61575 |
| F9 | -3.60439 | 0.000459 | 0.000689 | -0.1037 | -0.03016 | 0.993553 | -0.65807 |
| F10 | -3.06702 | 0.002681 | 0.002681 | -0.0959 | -0.02065 | 0.953892 | -0.55996 |
